# Treatment of Follicular Lymphoma With CHOP and Anti-CD20 Therapy: 15-Year Follow-Up of the SWOG S0016 Trial

**DOI:** 10.1001/jamaoncol.2026.0042

**Published:** 2026-02-26

**Authors:** Mazyar Shadman, Michael LeBlanc, Lisa Rimsza, John P. Leonard, Sonali M. Smith, Hongli Li, Jonathan W. Friedberg

**Affiliations:** 1 Fred Hutch Cancer Center, Seattle, Washington; 2 University of Washington, Seattle; 3 SWOG Statistical Center, Seattle, Washington; 4 University of Arizona, Tucson; 5 New York University, New York; 6 University of Chicago Medicine, Chicago, Illinois; 7 Wilmot Cancer Institute, University of Rochester, Rochester, New York

## Abstract

**Importance:**

Follicular lymphoma (FL) has historically been regarded as incurable, with patients experiencing late relapses after initial chemoimmunotherapy treatment.

**Objective:**

To provide 15-year follow-up data from the SWOG S0016 trial that evaluated the potential for long-term remission and cure following chemoimmunotherapy with cyclophosphamide, hydroxydaunorubicin/doxorubicin, oncovin, and prednisone/prednisolone (CHOP)–based regimens.

**Design, Setting, and Participants:**

This multicenter, intergroup study was conducted at academic and community practice locations throughout the US and enrolled patients with untreated, advanced-stage FL. Cure modeling, which involves estimating the proportion of patients cured of the disease, was conducted by incorporating background mortality rates to estimate the proportion of patients cured of FL during the S0016 trial. Patients were enrolled between May 2001 and October 2008 and followed up for a median (IQR) of 15.5 (13.6-16.9) years. The 15-year analysis was conducted in June 2025.

**Interventions:**

Patients were randomized to receive either rituximab plus CHOP (R-CHOP) or CHOP followed by radioimmunotherapy (CHOP-RIT).

**Main Outcomes:**

The main outcomes were 15-year progression free survival (PFS) and overall survival (OS). Secondary outcomes included cure modeling.

**Results:**

A total of 531 eligible patients (242 female patients [46%]; median [IQR] age, 53 [45-61] years) were included in the final analysis (267 [50%] received R-CHOP and 264 [50%] CHOP-RIT). The overall 15-year OS was 70%, with no significant difference between treatment arms, and the 15-year PFS was 40% (95% CI, 36.0%-44.7%). CHOP-RIT demonstrated superior 15-year PFS (47% vs 34%; *P* = .004) compared with R-CHOP. Cure modeling estimated an overall cure rate of 42%, with the highest cure rates observed in patients with low Follicular Lymphoma International Prognostic Index scores and normal β2 microglobulin levels. The rate of relapse declined substantially over time, from 6.8% during the first 5 years to 0.6% between 15 to 20 years.

**Conclusions and Relevance:**

The results of this secondary analysis suggest that a subset of patients with advanced-stage FL can achieve cure with CHOP-based chemoimmunotherapy, as relapse rates decline over time. This finding represents a paradigm shift in the understanding of and approach to FL, with implications for initial patient discussions and future research strategies.

**Trial Registration:**

ClinicalTrials.gov Identifier: NCT00006721

## Key Points

**Question:**

Is advanced-stage follicular lymphoma (FL) curable with standard chemoimmunotherapy treatment?

**Findings:**

In this secondary analysis of the SWOG S0016 trial of 531 individuals with FL, the overall 15-year overall survival was 70%, and cure modeling estimated an overall cure rate of 42%. The median follow-up was 15.5 years, and the rate of relapse declined substantially over time, from 6.8% during the first 5 years to 0.6% between 15 and 20 years.

**Meaning:**

The findings represent a major paradigm shift in the understanding and approach to FL, with broad implications for initial patient discussions, clinical care, and research strategies involving patients with FL.

## Teaser

This secondary analysis examines 15-year follow-up data from a randomized clinical trial to evaluate the potential for long-term remission and cure for follicular lymphoma following chemoimmunotherapy with cyclophosphamide, hydroxydaunorubicin/doxorubicin, oncovin, and prednisone/prednisolone (CHOP)–based regimens.

## Introduction

Historically, patients with advanced-stage follicular lymphoma (FL) were deemed to have incurable disease due to observed late relapses in clinical trials and natural history studies.^1,2^ During the past 20 years, since the introduction of anti-CD20 therapy into treatment paradigms, the outcomes for patients with FL have clearly improved, with several studies now demonstrating progression-free survival (PFS) beyond 8 to 10 years in a substantial minority of patients.^3,4,5^ Despite these improved outcomes, natural history studies have largely failed to show a plateau on event-free survival curves with few exceptions,^6^ and the landscape beyond the 10-year mark remains largely uncharted in the rituximab era.^7^ Therefore, the idea that FL remains an incurable disease with standard treatment approaches persists.

The definition of cure in a disease with indolent behavior is challenging because PFS curves include deaths of other causes. Mathematical cure models account for the possibility that some patients will not experience disease-related progression or death and offer a more accurate estimate of treatment benefits in a disease like FL, for which many long-term events may be unrelated to lymphoma. To address the curability of FL with standard chemoimmunotherapy regimens that use a cure model, we leveraged SWOG S0016, a phase 3 randomized clinical trial for patients with advanced-stage FL that compared rituximab, cyclophosphamide, hydroxydaunorubicin, vincristine sulfate, and prednisone (R-CHOP) with cyclophosphamide, hydroxydaunorubicin, vincristine sulfate, and prednisone (CHOP) followed by ^131^I-tositumomab radioimmunotherapy (RIT); initial and 10-year results were previously reported.^8,9^ In this current analysis, with a median follow-up now exceeding 15 years, we demonstrate achievement of cure for a substantial subset of patients and provide insights into the long-term efficacy estimates and safety profile of standard chemoimmunotherapy.

## Methods

### Patients and Treatment

The details of the SWOG S0016 study were previously published.^9^ All patients provided written informed consent according to institutional and federal guidelines. The trial was reviewed and approved by the central institutional review board in Rockville, Maryland, and was conducted according to the principles of the Declaration of Helsinki. Patients with untreated, CD20–positive FL with advanced-stage or bulky stage 2 disease, a performance status score of 0 to 2, and an indication for treatment were randomized to receive 6 cycles of either regimen (the Trial Protocol is available in Supplement 1). Patients were randomized to receive either R-CHOP or CHOP-RIT. R-CHOP was administered every 21 days (cyclophosphamide, 750 mg/m^2^, intravenously; doxorubicin, 50 mg/m^2^, intravenously; and vincristine, 1.4 mg/m^2^ and prednisone, 100 mg, orally once per day for 5 days). Rituximab was administered on days 1, 6, 48, 90, 134, and 141.^10^ In the CHOP-RIT arm, ^131^I-tositumomab was administered 4 to 8 weeks after the sixth cycle of CHOP.^11^ Maintenance therapy was not given. The trial followed the Consolidated Standards of Reporting Trials (CONSORT) reporting guideline.

### Follow-Up Assessments

Response assessment was performed on days 200 and 365 after treatment was initiated and when disease progression was suspected. Computed tomography was performed every 6 months for 2 years and annually afterward for 7 years or until relapse. Clinical follow-up was continued and performed every 6 months thereafter, including assessment for evidence of progressive disease and development of second cancers. The participating sites were approached for this analysis to provide detailed information on survival, progressive disease, and second cancers. The PFS and overall survival (OS) were the primary end points of the study.

### Statistical Analysis

PFS was defined as the time from random assignment to the first observation of progression or death. Patients last known to be alive and without progression were censored at the date of last contact. OS was defined as the time from random assignment to the date of death or last follow-up. PFS and OS were estimated according to the Kaplan-Meier method. The cumulative incidence of disease progression was calculated using the nonparametric Aalen-Johansen estimator, with death without progression/relapse treated as a competing risk. For comparisons between treatment arms, the Gray test and Fine-Gray model statistics (subdistribution hazard ratios [sHRs] with confidence intervals) were used.^12,13^ Fifteen-year point estimates and confidence intervals were used to describe long-term cumulative incidence. Death of other causes was considered a competing risk for these analyses. Analyses were conducted using SAS, version 9.4 (SAS Institute). Statistical significance was set at .05.

### Cure Modeling

The long-term follow-up of the S0016 trial permitted estimation of the proportion of patients who were cured of the disease (called *cure modeling*); the relative survival cure method was used to incorporate background mortality rates into the analysis.^14,15^ For the cure model component, we used a log-normal model parametric mixture cure model. The log-normal model mixture cure model was chosen among several potential cure models based on maximized Akaike Information Criterion. Model estimates and 95% CIs for cure rates were obtained via the R package flexsurvcure (R Foundation).^16^ Sensitivity analyses were conducted with multiple maximum follow-up times and inflating rates a later points. The cured subpopulation in this setting was assumed to die at the background mortality rate in the US population. For the subpopulation of individuals who were not cured, individuals were assumed to have potentially progressed with disease or died due to any cause. The survival and competing risk analyses assumed that patients lost to follow-up experienced events at the same time-dependent rate as those who remained under observation during the equivalent period. Therefore, the analysis did not assume that censored patients would not experience events. The life table estimates in the model used rates based on incorporating the year of randomization, age, and sex. The life table rates were obtained from the US Mortality DataBase using data from the mortality branch at the National Center for Health Statistics and US Centers for Disease Control and Prevention.^17^

## Results

### Baseline Characteristics

A total of 554 patients who were enrolled at centers participating in the National Clinical Trials Network were randomized between May 2001 and October 2008.^8^ Twenty-three patients did not meet protocol-specified inclusion criteria and were excluded from the final modified intent-to-treat analysis, leaving 531 eligible patients (267 in the R-CHOP arm and 264 in the CHOP-RIT arm) for the final analysis (eFigure 1 in Supplement 2). Baseline characteristics were previously reported and balanced and are shown in Table 1.^8^

**Table 1.** Baseline Characteristics

Characteristic	No. (%)		
	R-CHOP (n = 267)	CHOP-RIT (n = 264)	Missing data
Age, median (IQR), y	54.5 (45.1-61.7)	53.3 (45.9-60.9)	0
Sex			
Female	125 (47)	117 (44)	0
Male	142 (53)	147 (56)	0
Race and ethnicity			
Asian	5 (2)	3 (1)	0
Black	11 (4)	10 (4)	0
Multiracial	1 (0)	0	0
Native American	1 (0)	2 (1)	0
White	241 (90)	237 (90)	0
Unknown	8 (3)	12 (5)	0
Elevated β2M	141 (53)	144 (55)	0
B symptoms	77 (29)	68 (26)	2 (0.4)
Bulky disease (>10 cm)	63 (24)	68 (26)	0
BM involvement	149 (56)	146 (56)	1 (0.2)
Grade 3 histology	20 (8)	22 (9)	38 (7.2)
Stage			
II	11 (4)	5 (2)	0
III/IV	256 (96)	259 (98)	0
FLIPI			
Low	80 (30)	80 (30)	0
Intermediate	127 (48)	114 (43)	0
High	60 (22)	70 (27)	0

Abbreviations: β2M, β 2 microglobulin; BM, bone marrow; CHOP-RIT, cyclophosphamide, doxorubicin, vincristine, and prednisone followed by consolidation with iodine-133–tositumomab radioimmunotherapy; FLIPI, Follicular Lymphoma International Prognostic Index; R-CHOP, rituximab plus cyclophosphamide, doxorubicin, vincristine, and prednisone.

### Long-Term Efficacy

**Figure 1.**
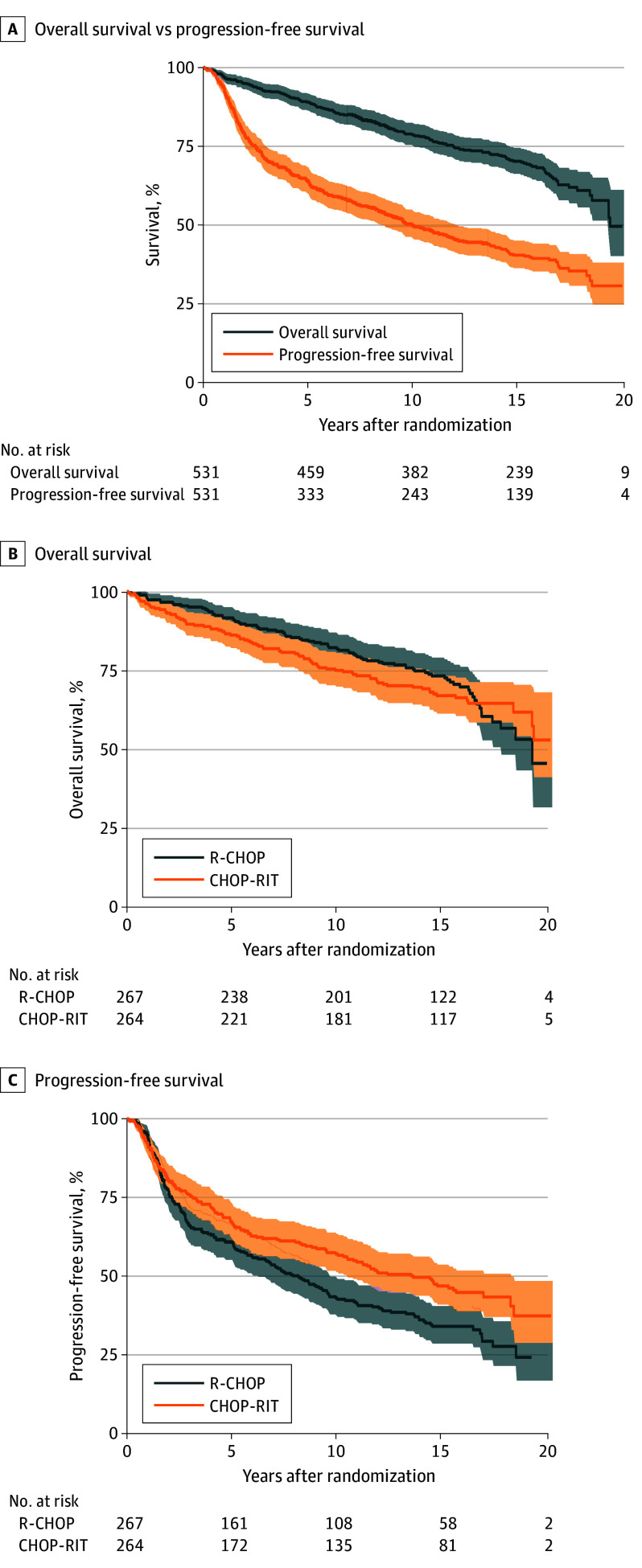
Survival Curves A, Overall survival and progression-free survival of all patients (n = 531) in the S0016 study who were randomly assigned to either rituximab plus cyclophosphamide, doxorubicin, vincristine, and prednisone (R-CHOP) or cyclophosphamide, doxorubicin, vincristine, and prednisone followed by consolidation with iodine-133–tositumomab radioimmunotherapy (CHOP-RIT). Comparison of overall survival (B) and progression-free survival (C) of patients who were randomized to receive 6 cycles of R-CHOP or 6 cycles of CHOP-RIT. 95% Pointwise confidence bands are shown.

The median (IQR) follow-up duration for this analysis was 15.5 (13.6-16.9) years. A total of 322 of 360 living patients (99%) had more than 10 years of follow-up, and 215 of 360 living patients (50%) had more than 15 years of follow-up. The 15-year OS for the entire study population was 70% (95% CI, 65.9%-74.1%) (Figure 1A). There was no difference in the 15-year OS rate between the R-CHOP (73%; 95% CI, 67.2%-78.4%) and CHOP-RIT (67%; 95% CI, 60.7%-72.6%) arms (*P* = .56) (Figure 1B). The 15-year PFS for the overall study participants was 40% (95% CI, 36.0%-44.7%) (Figure 1A). Patients in the R-CHOP arm (34%; 95% CI, 28.2%-40.0%) had a significantly lower 15-year PFS rate compared with patients in the CHOP-RIT arm (47%; 95% CI, 40.4%-53.0%; *P* = .004) (Figure 1C).

### Timing of Progression and Cure Estimate

**Figure 2.**
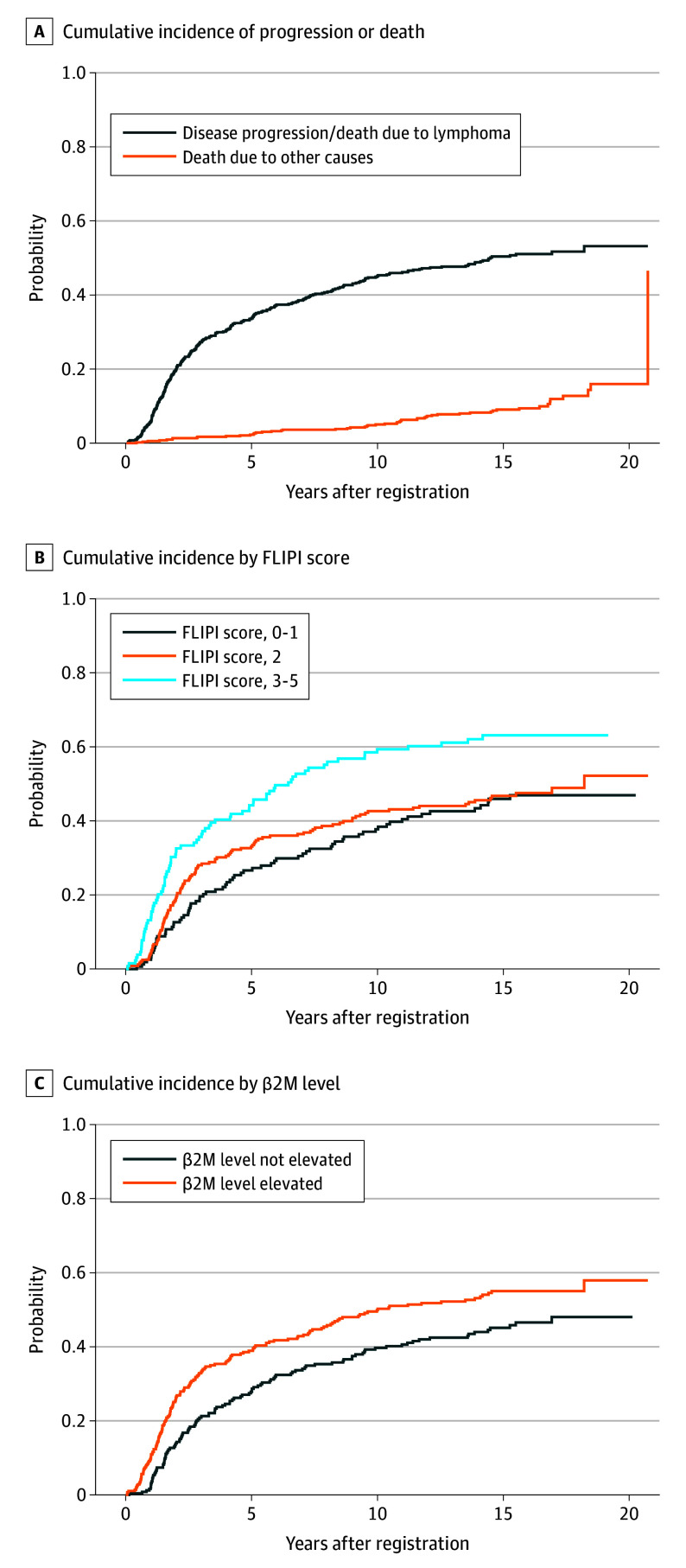
Cumulative Incidence Curves A, Cumulative incidence of lymphoma progression or lymphoma-related death. The dotted line shows deaths due to other causes without progression. The mean progression rates per year in different intervals after registration are depicted in 5-year intervals, showing a decline in the rate of progression of 6.8% (years 0-5), 2.3% (years 5-10), 1.1% (years 10-15), and 0.6% (years 15-20). B and C, Cumulative incidence shown by Follicular Lymphoma International Prognostic Index (FLIPI) scores and β2 microglobulin (β2M) levels.

**Figure 3.**
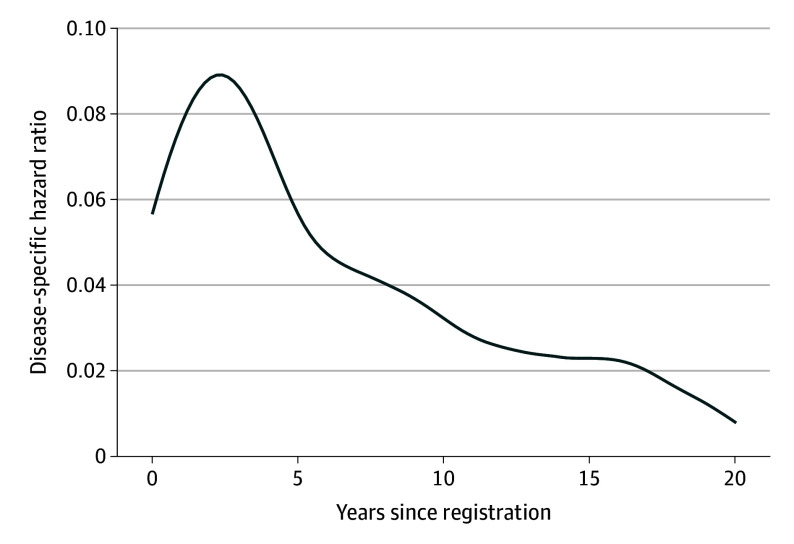
Hazard Curve The cause-specific hazard curve represents the smoothed rate of lymphoma progression or lymphoma-related death as a function of time. The decline suggests that the risk of lymphoma-related events diminishes with prolonged follow-up, approaching the level of the general population by 20 years, supporting the idea of cure in some patients.

Among 531 total patients, there were 316 PFS events (progression or death), and 215 patients (41%) were alive at last follow-up. Of those 316 initial PFS events, 262 (83%) were observed within 10 years, 41 events (8%) were observed between 10 to 15 years, and only 13 (2%) occurred after 15 years postregistration. The rate of mean (SE) progression, including deaths, decreased from 6.8% (0.4%) for the first 5 years after registration to 2.3% (0.1%) from years 5 to 10, 1.1% (0.1%) from years 10 to 15, and 0.6% (0.3%) from years 15 to 20 (Figure 2A). These estimates were consistent with the excess hazard curve, which represents the difference in the risk of death in the study population and age-matched and sex-matched general population over time (Figure 3).

The cure modeling led to an overall cure rate estimate of 42% (95% CI, 33%-52%). The cure rate was highest in patients with Follicular Lymphoma International Prognostic Index (FLIPI) score of 0 to 1 (52%; 95% CI, 40%-65%) and normal β2 microglobulin (β2M) levels (58%; 95% CI, 49%-67%) (Table 2). The cure rate estimate for R-CHOP arm was 35% (95% CI, 24%-47%).

**Table 2.** Cure Rate Estimates

Characteristic	Cure estimate, % (95% CI)
Overall	42 (33-52)
FLIPI score	
Low (0-1)	47 (30-65)
Intermediate (2)	46 (33-60)
High (3-5)	30 (17-48)
β2M	
Low	55 (44-66)
High	31 (20-46)

Abbreviations: β2M, β 2 microglobulin; FLIPI, Follicular Lymphoma International Prognostic Index.

### Sensitivity Analysis

To investigate the effect of reduced follow-up assessments in the long term, particularly beyond 10 years, a sensitivity analysis was conducted. In these analyses, hypothetical lymphoma progressions were added in varying amounts during the 10- to 15-year and 15- to 20-year intervals, and the data were reanalyzed using the same cure modeling methods as in the primary analysis. The estimated cure rate remained robust; for example, within a scenario assuming that 50% of lymphoma progressions were missed beyond 10 years, the resulting cure rate was still 34%. A range of assumptions and results are presented in the eAppendix and eTable 1 in Supplement 2. In separate sensitivity analysis, we also explored a flexible spline-based, nonmixture relative cure model, the cure rate overall was higher than that obtained from the mixture model (48% CI, 42%-56%). However, we believe nonmixture models are more difficult to interpret and thus have limited discussion to the mixture models.

### Prognostic Factors

As in prior reports, adjusting for treatment in a multivariable Cox model, FLIPI scores, and β2M levels were found to be significant prognostic factors for PFS.^18^ The estimated 15-year cumulative incidence of disease progression or death due to lymphoma by FLIPI score levels were low (0-1; 46%; 95% CI, 38%-54%), intermediate (2; 47%; 95% CI, 40%-53%), and high (3-5; 63%; 95% CI, 54%-71%). The estimates for within normal range serum β2M levels were 45% (95% CI, 39%-52%), and elevated β2M levels were 55% (95% CI, 49%-61%) (Figure 2, B and C). In both cases, the curves exhibited a plateau, and the long-term risk of relapse or lymphoma-related mortality was elevated for patients with high-risk baseline characteristics, suggesting that these factors may be associated with differing rates of functional cure in this disease context.

The approximate plateau in the cumulative incidence curves further supports the cure-rate analysis. Consistent with these findings, using the relative survival mixture cure model on the FLIPI score and serum β2M levels led to different estimated cure rates. The cure estimate for low FLIPI scores (0-1) was 47% (95% CI, 30%-65%), intermediate FLIPI score (2) was 46% (95% CI, 33%-60%), and high FLIPI scores (3-5) was 36% (31% CI, 17%-46%). The cure estimates for β2M levels within normal range was 55% (95% CI, 44%-66%) and was 31% for elevated β2M levels (95% CI, 20%-46%) (Table 2).

### Second Cancers

Fifty-nine patients (22.1%) receiving R-CHOP and 52 patients (19.7%) receiving CHOP-RIT developed secondary cancers. The most common nonhematologic cancers were nonmelanoma skin cancers (26 [4.9%]), genitourinary cancer (18 [3.4%]), and gastrointestinal cancers (14 [2.6%]). Six patients (2.2%) from the R-CHOP arm and 14 patients (5.3%) from the CHOP-RIT arm developed myeloid malignant neoplasms (acute myeloid leukemia [AML] or myelodysplastic syndromes [MDS]) (eTable 2 in Supplement 2).

### Cause of Death

There were 171 deaths (84 in the R-CHOP arm and 87 in the CHOP-RIT arm) during follow-up. The most common cause of death was lymphoma for 31 patients (11.6%) receiving R-CHOP and 40 patients (15.2%) receiving CHOP-RIT. However, lymphoma accounted for fewer than half of all deaths after R-CHOP (31 of 84 [36.9%]) and CHOP-RIT (40 of 87 [45.9%]) (eTable 3 in Supplement 2). Fifteen patients (5.6%) in the R-CHOP arm and 19 patients (7.2%) in the CHOP-RIT arm died of secondary cancers, with an estimated 15-year cumulative incidence of death of 5.4% (95% CI, 3.3%-8.9%) in R-CHOP and 7.0% (95% CI, 4.6%-10.8%) in CHOP-RIT (sHR, 1.3; 95 CI%, 0.66–2.55; *P* = .45), (eTable 4 and eFigure 2 in Supplement 2). Estimated 15-year cumulative incidences of death for AML/MDS were 1.2% (95% CI, 0.4%-3.7%) in R-CHOP and 4.4% (95% CI, 2.4%-8.0%) in CHOP-RIT (sHR, 3.8; 95% CI, 1.1-13.5; *P* = .03) (eTable 5 and eFigure 3 in Supplement 2). The few AML/MDS events limited the strength of treatment arm comparisons.

## Discussion

The long-term outcomes of this secondary analysis of a randomized clinical trial challenge the conventional view that FL is incurable, providing what is to our knowledge the strongest evidence to date that a cure is achievable for a subset of patients with advanced-stage disease after standard frontline chemoimmunotherapy. A 42% cure estimate (35% for R-CHOP) that was based on a relative survival model, along with 40% of patients remaining disease free after 15 years, indicated that CHOP-based chemoimmunotherapy resulted in cures. In our analysis, events were defined as lymphoma recurrence or lymphoma-related deaths, and deaths due to other causes without progression were considered as a competing risk; fewer than half of the observed deaths in the trial were related to lymphoma. This explains why the cured fraction of patients was higher than the reported PFS (which included deaths of nonlymphoma causes). These findings support the idea that a cure, defined as having no chance of lymphoma recurrence during a patient’s expected lifespan, is achievable for many patients with FL after only 1 treatment regimen and without maintenance therapy.

The PFS observed in this extended follow-up provides insights into clinical and research strategies when approaching first-line FL. Historically, a few reports suggested the potential for a plateau on the PFS curve after intensive chemoimmunotherapy regimens or high-dose therapy and autologous bone marrow transplant.^19,20^ A long-term analysis with shorter follow-up than our study of more recent trials confirmed the efficacy of chemoimmunotherapy strategies; for example, there was an 8-year PFS of 48% in the FOLL05 study (R-CHOP vs rituximab plus cyclophosphamide, vincristine, and prednisone vs rituximab plus fludarabine and mitoxantrone without rituximab maintenance) and a 7-year PFS of 63% for patients treated with chemotherapy and obinutuzumab in the GALLIUM study.^3,21^ In the PRIMA study, 10-year PFS was 35% for patients who received chemoimmunotherapy without rituximab maintenance.^4^ The StiL NHL1 study, with 9 years of follow-up, reported a 10-year PFS estimate of 71% for patients treated with bendamustine plus rituximab and 66% for those treated with R-CHOP without the use of maintenance therapy.^22^ Watanabe et al^23^ recently reported a 15-year PFS of 28.5% in patients with FL who received R-CHOP, although a cure model was not applied in that analysis. Studies that used nonchemotherapy options have also demonstrated lasting remissions.^24^

The observation of late relapses in our study should not be interpreted as evidence against the likelihood of cure for FL; they were consistent with our estimated cure model, which assumed no events will be seen in the cured subpopulation. However, given a persistent uncured population, there will be a few events at late times in the whole cohort. Also, although diffuse large B-cell lymphoma and classical Hodgkin lymphoma are considered curable, late relapses occur in these histologies.^25,26^ In our study, follow-up imaging was performed annually and reliably for 7 years, with subsequent assessments conducted clinically. This follow-up approach was considerably more aggressive than those used in any of the aforementioned studies, including those in established curable lymphomas.

Our findings potentially have substantial clinical implications. Initial counseling for patients with a new diagnosis of FL may now include the concept that some patients are cured, limiting the negative psychological effect of FL.^27^ Moreover, a FL diagnosis typically involves a lifelong relationship with a lymphoma specialist for clinical and, in some cases, radiologic assessments. Our findings support eliminating the need for indefinite oncology and radiologic follow-up, and transitioning management to primary care teams after 10 years may be a reasonable strategy.^28^

The results of this secondary analysis underscore chemoimmunotherapy as the standard of care for first-line treatment of FL and serve as a benchmark in designing clinical trials within this domain. The absence of maintenance rituximab in our study allowed for an unconfounded evaluation of long-term outcomes following induction chemoimmunotherapy alone. We acknowledge that the degree to which the anthracycline component of CHOP chemotherapy is required is unknown, as studies of bendamustine with shorter follow-up have similar intermediate-term outcomes. With the recent introduction of novel therapeutic classes for FL, there is an interest in considering these agents in the first-line setting.^24,29,30,31,32,33^ While nonchemoimmunotherapy options are appealing for select patients with comorbidities, our results highlight that chemoimmunotherapy remains the standard, particularly for individuals with favorable prognostic markers. Validated surrogates for clinical outcomes (eg, measurable residual disease) are opportunities to accelerate drug development in FL.^34,35^

Our findings suggest a higher likelihood of cure for patients with low-risk disease. The recently reported 15-year follow-up of a UK trial that compared watchful waiting with fixed-duration rituximab maintenance in low–tumor burden FL demonstrated that 65% of patients randomized to rituximab maintenance had not required additional therapy; cure modeling was not part of this analysis.^36^ Ongoing trials in low–tumor burden FL should be mandated to have long-term follow-up to best define curative potential (NCT06337318).^36,37^ Additionally, future studies should continue to explore the genomics and microenvironment of this cured population to further enable a precision approach to therapy in the future.^38,39^

### Strengths and Limitations

Our study had several strengths, including its design as a large, national trial with broad representation of the US population. To our knowledge, it also featured the longest follow-up period among modern-era frontline FL prospective clinical trials. Limitations included that most participants were non-Hispanic and White, and half received radioimmunotherapy, a treatment that is now rarely used.^40^ Also, our study did not address the question of the best chemoimmunotherapy regimen in the first-line setting. Despite our best efforts to maximize data capture and account for patients lost to clinical follow-up or imaging (which was not required after 7 years), loss to follow-up and censoring can limit the interpretation of the data. However, supplementary sensitivity analyses showed that the meaningful cure rate estimates were robust under a range of missed lymphoma event assumptions (eAppendix 1 in Supplement 2). Additionally, central imaging and pathology review at relapse was not conducted, limiting the ability to confirm FL relapse from histological transformation.

Novel therapeutic approaches, including chimeric antigen receptor T-cell therapies and bispecific T-cell–engaging antibodies have shown promising efficacy in patients with previously treated and high-risk FL.^30,41,42,43,44,45,46^ The time-limited use of these therapies, either as monotherapy or in combination with other agents, in earlier lines of treatment may offer the possibility of achieving cures to additional patients with FL.

## Conclusions

In this secondary analysis, the 15-year follow-up of the S0016 study demonstrated that some patients with an FL diagnosis achieved a cure following first-line treatment with chemoimmunotherapy. This finding potentially represents a paradigm shift in our understanding and approach to FL, with implications for initial patient discussions, clinical care, and research strategies involving patients with FL. As the field continues to explore options aimed at identifying effective, safe, and time-limited treatment strategies, chemoimmunotherapy should remain the standard of care for first-line treatment of FL.

## Notes

Supplement 1. Trial protocol

Supplement 2. eFigure 1. CONSORT Diagram eAppendix. Sensitivity Analysis eTable 1. Sensitivity Analysis of Simulated Late Events and Estimated Cure Fraction eTable 2. Secondary Malignancies eTable 3. Cause of Death by Treatment Arm eTable 4. Cause of Death due to Secondary Malignancies eFigure 2. Cause of Death due to Secondary Malignancies eTable 5. Cause of Death due to AML/MDS

Supplement 3. Data sharing statement
