# Supplementary material for: Treatment of Follicular Lymphoma With CHOP and Anti-CD20 Therapy: 15-Year Follow-Up of the SWOG S0016 Trial
Source: JAMA Oncol. 2026 Feb 26;12(4):394–401. doi: 10.1001/jamaoncol.2026.0042 (PMC12947078; doi:10.1001/jamaoncol.2026.0042)
Supplement: Supplement 1. — Trial protocol [file jamaoncol-e260042-s001.pdf]

Protocol Supplement

Table of Contents

Document	Page Number
Original Protocol, including statistical analysis plan	2
Final Protocol, including statistical analysis plan	91
Summary of Protocol Amendments	222

PRIVILEGED COMMUNICATION
FOR INVESTIGATIONAL USE ONLY

S0016
Draft August 16, 2000

SOUTHWEST ONCOLOGY GROUP

A PHASE III TRIAL OF CHOP VS CHOP + RITUXIMAB VS CHOP + IODINE-131-LABELED MONOCLONAL ANTI-B1 ANTIBODY (Tositumomab) FOR TREATMENT OF NEWLY DIAGNOSED FOLLICULAR NON-HODGKIN'S LYMPHOMAS

Page

SCHEMA 2
1.0 OBJECTIVES 3
2.0 BACKGROUND 3
3.0 DRUG INFORMATION 8
4.0 STAGING CRITERIA 17
5.0 ELIGIBILITY CRITERIA 19
6.0 STRATIFICATION FACTORS 22
7.0 TREATMENT PLAN 22
8.0 TOXICITIES TO BE MONITORED AND DOSAGE MODIFICATIONS 30
9.0 STUDY CALENDAR 34
10.0 CRITERIA FOR EVALUATION AND ENDPOINT DEFINITIONS 37
11.0 STATISTICAL CONSIDERATIONS 39
12.0 DISCIPLINE REVIEW 40
13.0 REGISTRATION GUIDELINES 40
14.0 DATA SUBMISSION SCHEDULE 41
15.0 SPECIAL INSTRUCTIONS 43
16.0 ETHICAL AND REGULATORY CONSIDERATIONS 43
17.0 BIBLIOGRAPHY 48
18.0 MASTER FORMS SET 51
19.0 APPENDIX 75

PARTICIPANTS: ALL SOUTHWEST ONCOLOGY GROUP, CCOP AND AFFILIATE MEDICAL ONCOLOGISTS, RADIATION ONCOLOGISTS AND PATHOLOGISTS

STUDY COORDINATORS:

Oliver Press, M.D., Ph.D. (Medical Oncology)
University of Washington Medical Center
Division of Medical Oncology
1959 Pacific St. Rm EE-122-F
P.O. Box 356043
Seattle, WA 98195
Phone: 206/598-4938
Fax: 206/598-4509
E-mail: press@u.washington.edu

David G. Maloney, M.D., Ph.D. (Medical Oncology)
Fred Hutchinson Cancer Research Center
1104 Fairview Avenue North
Seattle, WA 98104
Phone: 206/667-5616
Fax: 206/667-6922
E-mail: dmaloney@fhcrc.org

Rita M. Braziel, M.D. (Hematopathology)
Department of Pathology, L471
Oregon Health Sciences University
3181SW Sam Jackson Park Road
Portland, Oregon 97201
Phone: 503/494-2315
Fax: 503/494-0731
E-mail: braziel@ohsu.edu

AGENTS:

Cyclophosphamide (Cytoxan®) (NSC-26271)
Doxorubicin (NSC-123127)
Prednisone (NSC-10023)
Vincristine (Oncovin) (NSC-67574)
Iodine-131 Anti-B1 Antibody and Iodine-131 Anti-B1 Antibody (Tositumomab + I-131 tositumomab) (BB-IND-8283)
Rituximab Chimeric anti CD-20mab (IDEC-C2B8) (NSC-687451)

Michael LeBlanc, Ph.D. (Biostatistics)
Joseph M. Unger, M.S.
Southwest Oncology Group Statistical Center
Fred Hutchinson Cancer Research Center
1100 Fairview Avenue North, MP-557
P.O. Box 19024
Seattle, WA 98109-1024
Phone: 206/667-4623
Fax: 206/667-4408
E-mail: mikel@swog.fhcrc.org
E-mail: joeu@swog.fhcrc.org

mb

SCHEMA

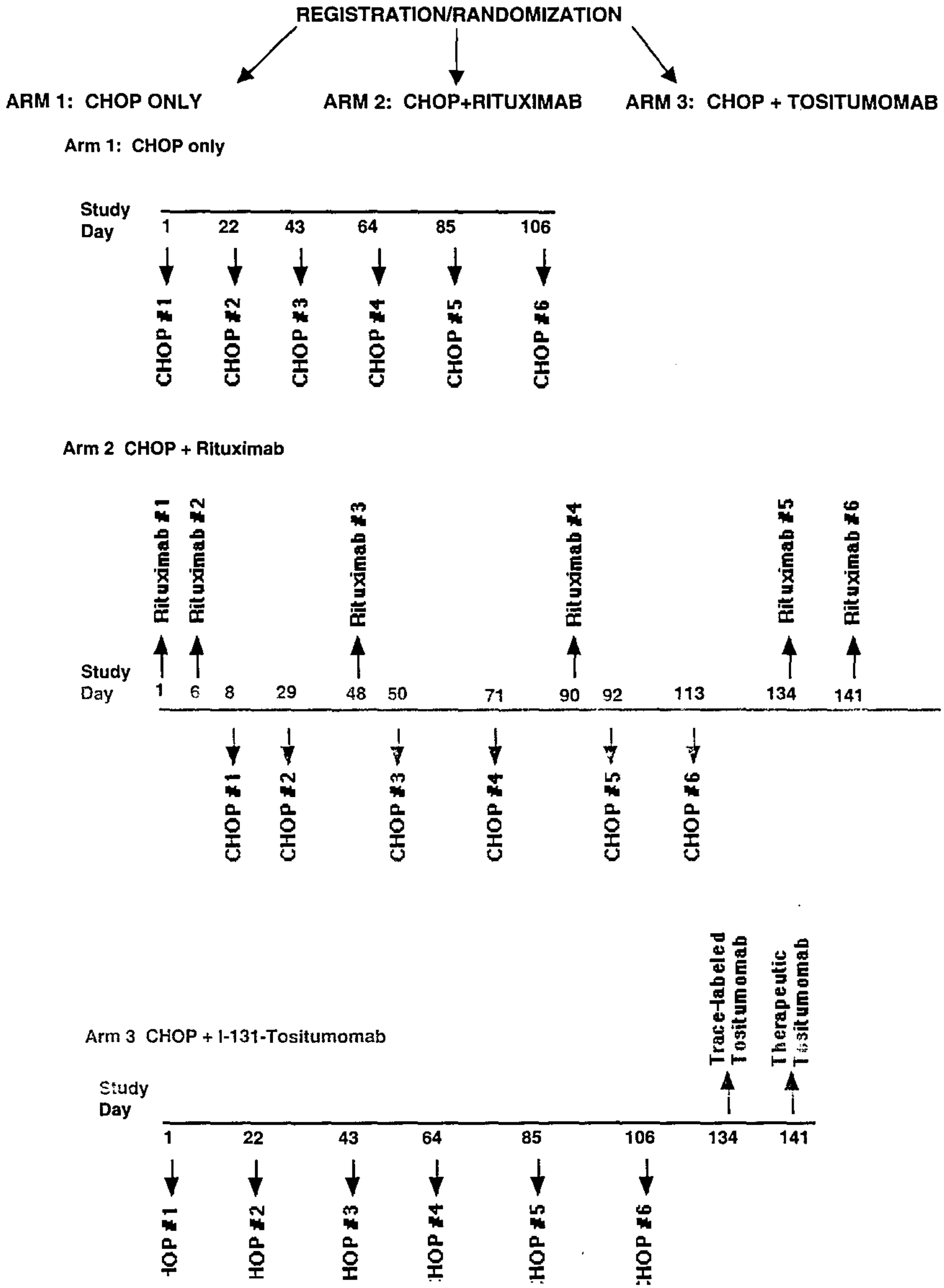
REGISTRATION/RANDOMIZATION
ARM 1: CHOP ONLY
ARM 2: CHOP+RITUXIMAB
ARM 3: CHOP + TOSITUMOMAB
Arm 1: CHOP only
Study Day
1
22
43
64
85
106
CHOP #1
CHOP #2
CHOP #3
CHOP #4
CHOP #5
CHOP #6
Arm 2 CHOP + Rituximab
Rituximab #1
Rituximab #2
Rituximab #3
Rituximab #4
Rituximab #5
Rituximab #6
Study Day
1
6
8
29
48
50
71
90
92
113
134
141
CHOP #1
CHOP #2
CHOP #3
CHOP #4
CHOP #5
CHOP #6
Arm 3 CHOP + I-131-Tositumomab
Trace-labeled Tositumomab
Therapeutic Tositumomab
Study Day
1
22
43
64
85
106
134
141
HOP #1
HOP #2
HOP #3
HOP #4
HOP #5
HOP #6

1.0 OBJECTIVES

1.1 To compare the progression-free survival and overall survival of patients with newly diagnosed follicular lymphoma (CD20+) treated with six cycles of CHOP chemotherapy alone, six cycles of CHOP with rituximab or six cycles of CHOP followed by Anti-B1 Antibody and Iodine-131 Anti-B1 Antibody (tositumomab + I-131 tositumomab).

1.2 To evaluate the response rate for patients with newly diagnosed follicular lymphoma (CD20+) treated with these regimens.

1.3 To evaluate the toxicities of CHOP with or without rituximab or Anti-B1 Antibody and Iodine-131 Anti-B1 Antibody in patients with newly diagnosed follicular lymphomas.

1.4 To assess the molecular remission rates by measuring clonal t(14;18)/bcl2 rearrangements in the bone marrow at baseline and at one year post-treatment.

2.0 BACKGROUND

Follicular lymphomas are characterized by an indolent clinical course with predominant nodal and marrow involvement. With the exception of a small fraction of patients (10 - 15%) with truly localized involvement, the majority of low-grade lymphoma patients do not achieve long-term remissions. *(1, 2)* There is no consensus on the best treatment approach for follicular lymphoma. Many asymptomatic patients are followed with "watchful waiting", because conventional chemotherapy regimens have not been shown to improve survival in this disease compared with a policy of expectant therapy. *(1)* Symptomatic patients are commonly treated with oral alkylating agents (e.g., chlorambucil), or with an alkylating agent (cyclophosphamide) in combination with a vinca alkaloid and corticosteroids ("CVP"), but studies have not demonstrated a survival advantage for patients treated with these regimens. Previous Southwest Oncology Group studies have shown approximately 65% two-year progression-free survival for this patient group. Because of the curative potential of anthracycline-based regimens (e.g., CHOP) for intermediate and high grade Non-Hodgkin's lymphomas, CHOP has been tested extensively by the Southwest Oncology Group in patients with low-grade lymphomas. *(3 - 6)* Four hundred fifteen patients with low grade malignant lymphomas were treated with CHOP between 1972 and 1983 on **SWOG-7204**, **SWOG-7426**, and **SWOG-7713**. Approximately 90% of these patients achieved objective responses, including 61 - 78% with complete remissions. *(3 - 5)* With a median follow-up of 12.8 years, the median survival was 6.9 years. *(6)* Toxicities included myelosuppression, nausea, paresthesias, infection, and cardiomyopathy (< 5%). Unfortunately, these studies failed to demonstrate a plateau on the survival curves of patients treated with CHOP, indicating that by itself CHOP has little curative potential for low grade lymphomas, in contrast to its efficacy for diffuse large cell lymphomas. *(6)* Whether cures with CHOP are possible for patients with follicular large cell lymphoma remains controversial, with conflicting studies from several institutions. *(7 - 10)* Nevertheless, all investigators agree on the necessity of developing better treatment regimens for patients with follicular lymphomas.

New chemotherapeutic agents (fludarabine, 2-chlorodeoxyadenosine) have shown considerable activity in patients with relapsed low grade lymphomas; however, no study has demonstrated that these drugs are curative for follicular lymphomas or that survival is prolonged by their use. **SWOG-9501** investigated the combination of fludarabine plus mitoxantrone in patients with newly diagnosed follicular lymphomas. This regimen was well tolerated and produced a high response rate, but the survival curves do not appear superior to those obtained with other regimens, including CHOP. *(11)*

High-dose chemoradiotherapy with bone marrow or peripheral blood stem cell transplantation is commonly employed for treatment of patients with relapsed lymphomas of all types. Preliminary data from the Dana Farber Cancer Institute suggest the disease-free survival after transplantation for follicular lymphomas relates to the quality of initial morphological remission obtained and the ability to decrease the number of cells bearing clonal rearrangements of the bcl2-oncogene (which is present in 80% of newly diagnosed follicular lymphomas). *(12)* However, the curability of follicular lymphomas with stem cell transplantation and the advisability of performing this procedure are controversial.

Clinical Studies with Rituximab

Rituximab is a chimeric mouse-human anti-CD20 monoclonal antibody which has been extensively evaluated as a single agent for the treatment of patients with multiply -relapsed B cell lymphoma. Data from a Phase I, dose-escalation study in 15 patients with relapsed NHL demonstrated no dose-limiting toxicity, two PRs, and four minor responses to a single infusion of the mAb using doses ranging from 10 to 500 mg/m^2. *(13)* The serum half-life at the higher dose levels ranged from 1.6 to 10.5 days and antibody levels > 10 ug/ml persisted for more than 14 days in 6 of 9 patients. Antibody infusion produced CD20 positive B-cell depletion in peripheral blood at 24 to 72 hours that persisted for 2 to 3 months in most patients. Flow cytometric evaluation of cell suspensions from lymph node biopsy specimens revealed B-cell depletion and tumor cells coated with antibody two weeks post-treatment.

A Phase I/II clinical trial utilized 4 weekly infusions of the antibody in doses ranging from 125 mg/m^2 to 375 mg/m^2. *(14)* There was no dose limiting toxicity observed in this trial and the 375 mg/m^2 dose was selected for Phase II and III clinical trials. In the Phase II portion of the trial, 37 patients with relapsed lymphoma were treated with 4 weekly infusions of rituximab at 375 mg/m^2 dose level. Overall, there was a 46% response rate. The median duration of these responses was 10.2 months. Three patients continue with ongoing response with durations of greater than 25 months. Tumor responses were observed in bulky lymph nodes, spleen, bone marrow, blood and extranodal masses. Toxicity was mild, with the majority of adverse events (67%) observed during the initial antibody infusion with a dramatically decreased incidence of any adverse events occurring with the subsequent antibody infusions. The most common adverse events include Grade 1 or 2 fever (73%), asthenia (16%), chills (38%), nausea (19%), vomiting (11%), rash (14%), and pain at tumor sites (3%). Grade 1 or 2 hypotension occurred in 3 patients (8%). The 37 patients received 142 antibody infusions, and 4 patients had events reported as Grade 3 or 4 (thrombocytopenia or neutropenia). B-cell depletion in the peripheral blood was observed in all patients and lasted approximately 6 months with gradual slow recovery.

A subsequent multi-center trial published by McLaughlin confirmed these findings, with 48% of 166 relapsed lymphoma patients (151 evaluable) achieving remissions with minimal toxicity (15), including 6% CRs. *(15)* The median response duration was approximately one year. Solal-Celigny has recently demonstrated that the response rates are substantially higher if newly diagnosed patients with follicular lymphoma are treated. *(16)* Of 50 previously untreated patients given rituximab, 28% achieved a complete remission and 46% a partial response, for an overall response rate of 74%.

Coiffier evaluated the efficacy of rituximab in 52 patients with aggressive lymphomas and demonstrated 5 complete and 12 partial remissions. *(17)* Approximately 30-35% of patients with diffuse large cell lymphomas and mantle cell lymphomas responded to the standard rituximab regimen in this study. These results have been confirmed in a recent multi-center European study treating 131 patients with newly diagnosed mantle cell lymphoma, immunocytoma, and small lymphocytic lymphoma. *(18)* The overall response rate was 30% (36 of 120 evaluable patients) including 37% responses in mantle cell lymphoma, 28% responses in immunocytoma, and 14% responses in small lymphocytic lymphoma.

Rituximab has been shown to deplete circulating B lymphocytes from the bloodstream for approximately six months after standard doses. Fortunately, this period of B cell depletion has not been associated with a high risk of infections, presumably because serum immunoglobulin levels remain near baseline levels. In addition, patients can be treated repetitively with chimeric anti-CD20 antibody since less than 1% of patients develop anti-chimeric antibody immune responses. Toxicities are usually limited to the infusional period with fever, chills, headache, pruritis, sweats, rash, and transient hypotension being most frequently observed. Classic toxicities associated with chemotherapy such as alopecia, nausea, and myelosuppression are rare with rituximab. However, rare fatal reactions have been observed, including adult respiratory distress syndrome and tumor lysis syndrome. These serious complications appear to be more common in patients with large numbers of circulating malignant B cells and patients with pulmonary infiltration by lymphoma. *(19)*

Despite the encouraging clinical results and success of rituximab, 40-50 per cent of patients with low grade lymphomas and 60 - 70 % of patients with aggressive lymphomas fail to respond to single-agent rituximab, only 5 - 10% of patients attain complete remissions, and the median response duration is only approximately one year. Consequently, many investigators have begun exploring methods of enhancing the potency of antibodies, including combining them with chemotherapy and conjugating them to toxins or radionuclides. Czuczman conducted a pilot trial administering CHOP chemotherapy (cyclophosphamide, doxorubicin, vincristine, and prednisone) with rituximab to forty patients with indolent lymphomas. *(20)* Objective remissions were observed in 38 patients (95%), including 22 CRs (55%), with a median time-to-treatment failure of more than 29 months.

Clinical Studies with Iodine-131 Anti-B1 Antibody

Phase I/II Study at University of Michigan (RIT-I-000)

An alternative approach to enhance the efficacy of anti-CD20 antibodies involves conjugating them to cytotoxic radionuclides which can then be selectively targeted to B lymphoid tumors. Study RIT-I-000 was the initial Phase I/II, open-label study of non-myeloablative doses of Iodine-131 anti-B1 antibody for the treatment of patients with B-cell NHL of all histologic types. *(21 - 23)* Fifty-nine patients were enrolled. Twenty-eight patients had low-grade NHL, 14 had transformed low-grade NHL, 15 had intermediate-grade NHL, and 2 had high-grade NHL. The median time from diagnosis was 45 months, the median number of prior therapies was 4, 88% had Stage III or IV disease, 36% had bulky disease, 51% had an elevated LDH, and 24% had failed bone marrow transplant (BMT). Patients received 1 - 3 dosimetric doses followed by a therapeutic dose. The dosimetric dose(s) involved the IV administration of 5 mCi of Iodine-131 anti-B1 antibody to determine the rate of whole body clearance so that a whole body radiation dose (cGy) could be calculated. Each dosimetric dose was preceded by 0, 95, or 475 mg of unlabeled antibody. Therapeutic dose-escalation was initiated at 25 cGy and adjusted in 10 cGy increments until the maximum tolerated dose (MTD) was reached. Fifty-three of the 59 patients received a therapeutic dose. The MTD was 75 cGy for patients who had not undergone BMT. Based on all data through 7/31/97, a response was observed in 42/59 (71%) patients and a complete response (CR) was observed in 20/59 (34%) patients. The median duration of response was 271 days (95% confidence interval: 140 - 394 days) and median duration of CR was 566 days (95% confidence interval: 385 days to upper limit not reached). Nine of the 59 patients remain in CR. Responses were observed in 50% of post-BMT patients and 52% of bulky disease patients. A response was observed in 24/28 (86%) patients and a CR was observed in 13/28 (46%) patients with low-grade NHL. A response was observed in 11/14 (79%) patients and a CR was observed in 7/14 (50%) patients with transformed low-grade NHL. Dose-dependent pharmacokinetics were observed. The mean tumor dose was 14.5 times the whole body dose. The dose-limiting toxicity was hematologic; 3 patients developed a platelet count < 10,000 cells/mm^3 and 2 patients had an ANC < 100 cells/mm^3. The most prevalent non-hematologic toxicities were transient, mild to moderate fever, nausea, asthenia, and chills. Nine of 59 (15%) patients developed human anti-murine antibodies (HAMA).

Iodine-131 Anti-B1 Antibody Phase II Dosimetry Validation Study (RIT-II-001)

Study RIT-II-001 was a Phase II, multicenter, open-label study of non-myeloablative doses of Iodine-131 anti-B1 antibody for the treatment of patients with low-grade B-cell lymphomas and transformed low-grade lymphomas. Thirty-seven patients had low-grade NHL and 10 had transformed low-grade NHL. The median time from diagnosis was 41 months, the median number of prior therapies was 4, 91% had Stage III or IV disease, 44% had bulky disease, 44% had an elevated LDH. Patients received 1 dosimetric dose followed by a therapeutic dose. The dosimetric dose involved the IV administration of 450 mg of unlabeled antibody and 35 mg (5 mCi) of Iodine-131 anti-B1 antibody to determine the rate of whole body clearance so that a whole body radiation dose (cGy) could be calculated. The therapeutic dose involved IV administration of 450 mg of unlabeled antibody and 35 mg of Iodine-131 anti-B1 antibody with radioactive Iodine-131 titrated to deliver 75 cGy. Forty-five of the 47 patients received a single dosimetric and therapeutic dose of Iodine-131 anti-B1 antibody as described in the protocol. A response was observed in 27/47 (57%) patients and a complete response (CR) was observed in 14/47 (30%) patients. The median duration of response was 248 days (95% confidence interval:

136 days to upper limit not reached) and median duration of CR was not reached (95% confidence interval: 414 days to upper limit not reached). Ten of the 47 patients remain in CR with ongoing responses ranging from 245-606 days. All clinical sites had at least 1 patient who achieved a CR. Responses were observed in 59% of bulky disease patients. A response was observed in 21/37 (57%) patients and a CR was observed in 9/37 (24%) patients with low-grade NHL. A response was observed in 6/10 (60%) patients and a CR was observed in 5/10 (50%) patients with transformed low-grade NHL. The mean tumor dose was 10.6 times the whole body dose. The dose-limiting toxicity was hematologic; 5 patients developed a platelet count <10,000 cells/mm^3 and, mild to moderate asthenia, nausea and fever. Only 1 of 46 (2%) patients developed HAMA following treatment as assessed by centralized validated HAMA assay.

Other groups have also investigated the therapeutic efficacy of radiolabeled anti-CD20 antibodies. Press and colleagues studied myeloablative doses of the same radioiodinated antibody (anti-B1, Coulter Pharmaceuticals) with autologous stem cell support in Phase I and II trials in Seattle. *(24 - 25)* Twenty-nine patients with multiple relapsed B cell lymphomas were treated with single agent I-131-anti-CD20 (B1) antibody (2.5 mg/kg, 280 to 785 mCi) followed by autologous hematopoeitic stem cell rescue between 2/90 and 7/94. Objective responses occurred in 86% of patients, including 79% complete responses. Early toxicities included Grade 4 myelosuppression in all patients, Grade 2 - 3 nausea in 8 of 29, and fatal sepsis in one. Reversible cardiopulmonary failure was the dose-limiting non-hematopoietic toxicity (2 pt.), occurring at an estimated absorbed lung dose of 27 Gy. Overall survival and progression-free survival were 83% and 52%, respectively, after a median follow-up of 38 months. None of the surviving patients had long term objective impairment of performance status or cardiopulmonary function, though one patient with a pre-existing anthracycline-induced cardiomyopathy remained on digoxin (with an ejection fraction of 69%) four years after radioimmunotherapy. No serious delayed cardiopulmonary complications occurred. Late toxicities were minimal, except for elevation of the thyroid stimulating hormone level in 59% of the subjects. Two patients who had been heavily pretreated with alkylating agents and external beam irradiation developed acute leukemia eight years after radioimmunotherapy. Two patients developed secondary solid neoplasms 3 years after treatment (1 noninvasive transitional cell carcinoma of the bladder; 1 metastatic colon cancer).

A similar series of trials have been conducted using the Y-90-labeled 2B8 antibody (Ibritumomab Tiuxetan, Zevalen®). Knox administered Yttrium-90-labeled anti-CD20 antibodies to 18 patients with relapsed B cell lymphomas (4 treated with Y-90-tositumomab antibody and 14 with ibritumomab tiuxetan) in escalating single doses of 13.5 to 50 mCi. *(26)* Six complete remissions and 7 partial responses were observed (overall response rate, 72%), with a median response duration of six months. Four patients developed human anti-mouse antibodies (HAMA). Grade 4 myelosuppression was seen at doses above 50 mCi of Y-90, but no other serious toxicities were observed. Witzig performed a subsequent multi-center Phase I/II trial treating 51 patients with Ibritumomab Tiuxetan (Y2B8, Zevalen®). *(27)* In the phase I portion of the trial, patients received either 100 or 250 mg/m^2 of unlabeled rituximab followed by 0.2, 0.3, or 0.4 mCi/kg of Y2B8. The optimal dose of unlabeled rituximab was determined to be 250 mg/m^2 and 0.4 mCi/kg was the maximally tolerated dose of Yttrium-90. In an intent-to-treat analysis, the overall response rate was 67%, including 26% CRs and 41% PRs. Eighty-two per cent of the low grade lymphoma patients responded (26% CRs, 56% PRs), and 43% of 14 aggressive lymphomas responded. Hematologic toxicity was dose-limiting. Only one patient developed an immune response.

Witzig has recently presented an interim analysis of a randomized clinical trial comparing the remission rates of patients treated with rituximab (375 mg/m^2 weekly for 4 weeks) or Y-90-ibritumomab tiuxetan (0.4 mCi/kg). *(28)* One hundred and forty three patients were entered on the randomized trial, but only 90 patients were evaluable at the time of the interim analysis in December, 1999. The overall response rate was 80% for the Y-90-labeled antibody as compared with 44% for the chimeric unlabeled rituximab antibody (p<0.001). The complete response rate was also higher in the group receiving radioimmunotherapy compared with the rituximab group (21% vs 7%, respectively). Both regimens were well tolerated, but significantly more myelosuppression occurred in the radiolabeled antibody group compared with the unlabeled chimeric antibody, as expected. This is the first randomized, controlled trial demonstrating unequivocally that a radiolabeled antibody produces higher overall and complete response rates

than the corresponding unlabeled antibody. Whether the higher response rates with radiolabeled antibodies will translate into longer event-free or overall survival will require longer follow-up.

Despite the impressive efficacy of single agent radiolabeled anti-CD20 antibodies for patients with relapsed lymphomas, several problems remain. Despite high response rates to non-myeloablative doses of Iodine-131 anti-B1 antibody it is unlikely that many will be permanently cured. High doses of I-131-labeled anti-CD20 induce complete remissions in most patients, and approximately half will be alive and disease-free after 5 years, but the treatment is toxic and expensive and requires a protracted hospitalization. The current protocol will study the tolerability, feasibility and efficacy of combining standard CHOP chemotherapy with non-myeloablative doses of Iodine-131 anti-B1 antibody or with rituximab, in hopes of developing a regimen which will induce durable remissions or cures in patients with follicular lymphomas without serious toxicities.

Rationale for Current Protocol

The Southwest Oncology Group and other cooperative groups have recently focussed their activities on studying the possible utility of chemotherapy followed by adjuvant immunotherapy for changing the natural history of follicular lymphoma. **SWOG-8809** tested the use of interferon as a maintenance agent compared to no maintenance therapy; unfortunately, interferon was not found to be beneficial in this study and was poorly tolerated by patients. *(23)* **S9800** was a Phase II pilot study studying the feasibility and toxicity of administering CHOP followed by a chimeric anti CD20 antibody, rituximab (Rituxan®, IDEC Pharmaceuticals). Similarly, **S9911** was a Phase II pilot study studying the feasibility and toxicity CHOP followed by an Iodine-131-labelled anti-CD20 (anti-B1) antibody (tositumomab + I-131 tositumomab). Both of these pilot studies have documented the feasibility of combining CHOP with anti-CD20 targeted therapy and documented high response rates and tolerable toxicities. The present study is intended to be a definitive Phase III, randomized comparison of CHOP chemotherapy alone with CHOP +rituximab and CHOP + I-131-tositumomab to assess whether adjuvant anti-CD20 targeted immunotherapy affects the overall and progression-free survival of patients with newly diagnosed follicular lymphomas, compared with chemotherapy alone.

The choice of a chemotherapy regimen for such a study is controversial, since no regimen has demonstrated clear superiority over any other (Miller, 1997). Although a strong case can be made for using chlorambucil or CVP as the "standard" regimen for indolent NHL, CHOP was chosen for this trial for several compelling reasons. First, the use of a moderately aggressive regimen such as CHOP is considered most likely to produce a state of minimal tumor burden, which is considered the ideal setting for immunotherapies. (Antibody molecules are large proteins which penetrate large tumor masses poorly. Furthermore, theoretical models suggest that the maximal crossfire from radiolabeled antibodies occurs when cell clusters are less than 1 mm in diameter). *(29)* Second, other adjuvant immunotherapies such as interferon-alpha have been shown to produce an advantage in terms of progression-free or overall survival only in studies using aggressive chemotherapy regimens such as CHOP and not when combined with regimens such as chlorambucil or CVP. *(30, 31)* Finally, a pilot trial by Myron Czuczman combining CHOP chemotherapy with rituximab has demonstrated a promising 95% overall response rate and a 75% two year progression free survival in newly diagnosed patients. *(20)* In the current Phase III trial we plan to administer concurrent CHOP + rituximab on one of the treatment arms since preliminary data by Czuczman and by Demidem suggest that this schedule may provide synergistic effects. Concurrent administration of CHOP and I-131-tositumomab is not possible because both produce myelosuppression, and therefore I-131-tositumomab will be given sequentially approximately four weeks after the last cycle of CHOP chemotherapy, as we have done in our pilot study, **S9911**.

Patients known to be HIV-positive are not eligible for this study because the severely depressed immune system and poor bone marrow reserve found in HIV infected patients, as well as the possibility of premature death, would compromise study objectives. Pregnant or nursing women are also not eligible for this study, due to the possibility that congenital abnormalities or harm to nursing infants may be caused by this treatment regimen.

Inclusion of Women and Minorities:

This study is open to women and minorities. based on recent registrations to studies involving non-Hodgkin's disease (follicular type) studies it is anticipated that accrual in the race and sex subgroups will be as shown in the table below.

	White, not of Hispanic Origin	Hispanic	Black, not of Hispanic Origin	Native Hawaiian or other Pacific Islander	Asian	American Indian or Alaskan Native
Female	283	5	14	2	4	0
Male	433	8	20	1	4	1

We are aware of no evidence indicating an interactive effect of treatment by sex or race in this group of patients.

There are no plans to incorporate sex or race specific accrual goals into this study. However, there will be an exploratory analysis of interaction between treatment effect and sex or race on outcome

3.0 DRUG INFORMATION

3.1 Cyclophosphamide (Cytoxan®) (NSC-26271)

a. DESCRIPTION

2-[bis(2-chloroethyl)amino]tetrahydro-2H-1,3,2-oxazaphosphorine 2-oxidemonohydrate. Cyclophosphamide is biotransformed principally in the liver to active alkylating metabolites which cross-link to tumor cell DNA.

b. TOXICOLOGY

Human Toxicology: Toxicity from cyclophosphamide includes bone marrow suppression which usually occurs 10 to 12 days after administration, nausea, vomiting, anorexia, abdominal discomfort, diarrhea, stomatitis, hemorrhagic colitis, jaundice, reversible alopecia, hemorrhagic cystitis which can frequently be prevented with increased hydration, hematuria, ureteritis, tubular necrosis, fibrosis of the bladder, cardiac toxicity which may potentiate doxorubicin-induced cardiotoxicity, rare anaphylactic reaction, skin rash, hyperpigmentation of the skin and nails, interstitial pulmonary fibrosis, and cross sensitivity with other aklylating agents. Treatment with cyclophosphamide may cause significant suppression of the immune system

Second malignancies, most frequently of the urinary bladder and hematologic systems, have been reported when cyclophosphamide is used alone or with other anti-neoplastic drugs. It may occur several years after treatment has been discontinued. It interferes with oogenesis and spermatogenesis and may cause sterility in both sexes which is dose and duration related. It has been found to be teratogenic, and women of childbearing potential should be advised to avoid becoming pregnant. Increased myelosuppression may be seen with chronic administration of high doses of phenobarbital. Cyclophosphamide inhibits cholinesterase activity and potentiates effect of succinylcholine chloride. If patient requires general anesthesia within 10 days after cyclophosphamide administration, the anesthesiologist should be alerted. Adrenal insufficiency may be worsened with cyclophosphamide. Cyclophosphamide is excreted in breast milk, and it is advised that mothers discontinue nursing during cyclophosphamide administration. The occurrence of acute leukemia has been reported rarely in patients treated with anthracycline/alkylator combination chemotherapy.

c. PHARMACOLOGY

Kinetics: Cyclophosphamide is activated principally in the liver by a mixed function microsomal oxidase system. PO administration is well absorbed, with bioavailability greater than 75%. Five to twenty-five percent of unchanged drug is excreted in the urine. Several active and inactive metabolites have been identified with variable plasma protein binding. There appears to be no evidence of clinical toxicity in patients with renal failure, although elevated levels of metabolites have been observed.

Formulation: Cyclophosphamide is supplied in 100 mg, 200 mg, 500 mg, 1 gram and 2 gram vials as a white powder. The drug should be reconstituted with Sterile Water for Injection, USP, and may be diluted in either normal saline or D5W.

Storage and Stability: Although the reconstituted cyclophosphamide is stable for six days under refrigeration, it contains no preservatives and therefore should be used within 6 hours.

Administration: Cyclophosphamide should be diluted in about 150 cc of normal saline or D5W and infused IV. An added dose of IV fluids may help prevent bladder toxicity. The tablet form of the drug may also be administered PO.

Supplier: Cyclophosphamide is commercially available and should be purchased by a third party. This drug will not be supplied by the NCI.

3.2 Doxorubicin (NSC-123127)

a. DESCRIPTION

Mechanism of Action: Doxorubicin is a cytotoxic anthracycline antibiotic different from daunorubicin by the presence of a hydroxyl group in the C-14 position. Doxorubicin is produced by fermentation from S. *Peucetius* var. *caesius*. Its mechanism of action is thought to be the binding of nucleic acids, preventing DNA and possibly RNA synthesis.

b. TOXICOLOGY

Human Toxicology: Studies with doxorubicin have shown that the major toxic effects of this drug are alopecia, which is often total but always reversible; nausea and vomiting, which develops shortly after drug administration, occasionally persisting for 2 - 3 days; fever on the day of administration; and phlebitis at the site of the drug's injection. Extravasation of the drug will lead to soft tissue necrosis. Phlebosclerosis, cellulitis, vesication and erythematous streaking have also been seen. Mucositis may be seen 5 - 10 days after administration. Ulceration and necrosis of the colon, particularly the cecum, with bleeding and severe infection have been reported with concomitant administration of cytarabine. Anorexia and diarrhea have also been observed. Hyperpigmentation of nailbeds and dermal creases, onycholysis and recall of skin reaction from prior radiotherapy may occur. Cardiac toxicity manifested as acute left ventricular failure, congestive heart failure, arrhythmia or severe cardiomyopathy has been reported, but appears to occur predominantly in patients who receive total doses in excess of 550 mg/M^2. Myelosuppression, predominantly neutropenia, is common with nadir occurring approximately two weeks after a single injection; lesser degrees of anemia and thrombocytopenia have been reported. Rapid recovery of the blood counts approximately two and a half weeks after a single injection generally permits an every three week schedule. Patients with obstructive liver disease have more severe myelosuppression due to impaired drug excretion. Thus, patients with hepatic dysfunction may need to have reduced dosage or to be excluded from therapy.

Renal excretion of doxorubicin is minimal, but enough to color the urine red; thus impaired renal function does not appear to increase the toxicity of doxorubicin. Other side effects include fever, chills, facial flushing, itching, anaphylaxis, conjunctivitis and lacrimation. The occurrence of acute leukemia has been reported rarely in patients treated with anthracycline/alkylator combination chemotherapy.

c. PHARMACOLOGY

Kinetics: Intravenous administration is followed by a rapid plasma clearance with significant tissue binding. Urinary excretion is negligible; biliary excretion accounts for 40 to 50% of the administered dose being recovered in the bile or the feces in 7 days. The drug does not cross the blood-brain barrier.

Formulation: Doxorubicin is supplied in 10, 20 and 50 mg single-use vials, and 150 mg multidose vials as a red-orange, lyophilized powder which has a storage stability of at least two years - see expiration date on vial. Doxorubicin should be reconstituted with 5, 10, 25 and 75 ml respectively, of Sodium Chloride Injection, USP (0.9%) to give a final concentration of 2 mg/ml.

Storage and Stability: The reconstituted doxorubicin is stable for 24 hours at room temperature and 48 hours under refrigeration (2° - 8°C). It should be protected from exposure to sunlight. Discard any unused solution from the vials. Bacteriostatic diluents with preservatives are NOT recommended as they might possibly worsen the reaction to extravasated drug.

Administration: Doxorubicin may be further diluted in 5% dextrose or sodium chloride injection and should be administered slowly into tubing of a freely flowing intravenous infusion with great care taken to avoid extravasation.

Supplier: This drug is commercially available for purchase by the third party. This drug will not be supplied by the NCI.

3.3 Prednisone (NSC-10023)

a. DESCRIPTION

Prednisone is a glucocorticoid rapidly absorbed from the GI tract.

b. TOXICOLOGY

Human Toxicology: Possible adverse effects associated with the use of prednisone are: fluid and electrolyte disturbances, congestive heart failure in susceptible persons, hypertension, euphoria, personality changes, insomnia, mood swings, depression, exacerbation of infection (e.g., tuberculosis), exacerbation or symptoms of diabetes, psychosis, muscle weakness, osteoporosis, vertebral compression fractures, pancreatitis, esophagitis, peptic ulcer, dermatologic disturbances, convulsions, vertigo and headache, endocrine abnormalities, ophthalmic changes, and metabolic changes. Some patients have experienced itching and other allergic, anaphylactic or other hypersensitivity reactions. Withdrawal from prolonged therapy may result in symptoms including fever, myalgia and arthralgia. Phenytoin phenobarbitol and ephedrine enhance metabolic clearance of corticosteroids.

Corticosteroids should be used cautiously in patients with hypothyroidism, cirrhosis, ocular herpes simplex, existing emotional instability or psychotic tendencies, nonspecific ulcerative colitis, diverticulitis, fresh intestinal anastomoses, peptic ulcer, renal insufficiency, hypertension, osteoporosis and myasthenia gravis. Immunization procedures (especially smallpox vaccination) should not be undertaken in patients on corticosteroids.

c. PHARMACOLOGY

Kinetics: Natural and synthetic glucocorticoids are readily and completely absorbed from the GI tract. Prednisone is very slightly soluble in water. Glucocorticoids have salt-retaining properties. The anti-inflammatory property of this drug is its ability to modify the body's immune system. On the other hand, glucocorticoids suppress the body's response to viral as well as bacterial infections. Equivalent doses are as follows:

Dexamethasone	Methyl-prednisolone and Triamcinolone	Prednisolone and Prednisone	Hydrocortisone	Cortisone
0.75 mg	4 mg	5 mg	20 mg	25 mg

Formulation: Prednisone is available in 2.5 mg, 5 mg, 10 mg, 20 mg and 50 mg tablets.

Storage and Stability: Prednisone should be stored at room temperature.

Administration: Prednisone is administered orally.

Supplier: Prednisone is commercially available and should be purchased by third party. Prednisone will not be supplied by the NCI.

3.4 Vincristine (Oncovin) (NSC-67574)

a. DESCRIPTION

Chemistry: Vincristine is one of the so-called vinca-alkaloids and is extracted from the plant cantharanthus roseus (vinca rosea).

Biochemistry: This drug appears to produce the arrest of mitosis in animal cells by interfering with microtubule function.

b. TOXICOLOGY

Human Toxicology: The primary toxic effects of vincristine are neurological with paresthesia, weakness, muscle wasting, motor difficulties including difficulty walking and slapping gait, loss of deep tendon reflexes, sensory loss, neuritic pain, paralytic ileus, bladder atony, and constipation. Rarely, it produces myelosuppression. Other side effects may include alopecia, allergic reactions, (including rare anaphylaxis, rash and edema), jaw pain, hypertension, hypotension, nausea, vomiting, diarrhea, fever, headache, oral ulceration, optic atrophy with blindness, ptosis, diplopia and photophobia. The occurrence of acute leukemia has been reported rarely in patients treated with anthracycline/alkylator combination chemotherapy.

c. PHARMACOLOGY

Kinetics: After IV administration, a triphasic serum decay pattern follows with half-lives of 5 minutes, 2-3 hours, and 85 hours. The range of terminal half-life is 19-155 hours. Excretion is 80% in the feces and 10-20 % in the urine.

The liver is the major excretory organ in humans and animals, and biliary obstruction causes increased toxicity in man.

Formulation: 1 mg/1 ml, 2 mg/2 ml, and 5 mg/5 ml vials containing solution. It is also available in 1 mg/ml and 2 mg/2 ml disposable syringes.

Storage and Stability: It should be stored under refrigeration. Vincristine is available with and without preservatives so the time-frame for use once the vial has been entered varies. The intact vials have a labelled expiration date. Protect from light. Parenteral drug products should be inspected visually for particulate matter and discoloration prior to administration.

Administration: Vincristine should be administered intravenously through a freely-running IV. If it extravasates, it produces a severe local reaction with skin slough. **FATAL IF GIVEN INTRATHECALLY, FOR INTRAVENOUS USE ONLY.**

Supplier: Vincristine is commercially available, and should be purchased through a third party. This drug will NOT be supplied by the NCI.

3.5 Iodine-131-labeled Murine Monoclonal Anti-B1Antibody (tositumomab)(BB-IND-8283)

a. DESCRIPTION

Anti-B1 Antibody/Iodine-131 Anti-B1 Antibody

Anti-B1 Antibody is an IgG2a Kappa (murine) monoclonal antibody that binds to the CD20 antigen on the surface of the normal and malignant human B cells to induce apoptosis and mediate antibody-dependent cellular cytotoxicity. Iodine-131 Anti-B1 Antibody is the radionuclide-labeled monoclonal antibody that can recognize tumor-associated antigens to selectively target radioactivity to tumor cells. By using isotopes emitting beta particles to label this antibody, the radiation emitted from the radiolabeled antibody bound to a tumor cell also kills neighboring cells because the path length of beta particles can extend over several cell diameters. This crossfire of beta particles can destroy antigen-positive and -negative tumor cells, as well as untargeted antigen-positive tumor cells within a tumor.

Iodine-131 Anti-B1 Antibody, used in conjunction with Anti-B1 Antibody, is a radioimmunotherapeutic agent being studied for the treatment of non-Hodgkin's lymphoma and other CD20-expressing B-cell malignancies.

b. TOXICOLOGY

Human Toxicology: Unlabeled anti-B1 antibody and Iodine-131 anti-B1 antibody infusions have been administered to over 250 patients in non-myeloablative doses for the treatment of non Hodgkin's lymphoma. The infusions are accompanied by few or no adverse experiences in most patients. The most frequent non-hematologic adverse experience reported was a transient mild to moderate flu-like syndrome consisting of fever (39%), and chills (19%). Other adverse experiences commonly reported include nausea (39%), asthenia (36%), headache (23%), rash (18%), anorexia (16%), infection (16%), pain (16%), myalgia (16%), arthralgia (14%), pruritus (14%), abdominal pain (13%), vomiting (13%), pharyngitis (11%), diarrhea (10%), and increased cough (10%). The concomitant administration of SSKI or other oral iodine product may contribute to the nausea and other gastrointestinal adverse experiences. Only 3% and 4% of patients experienced an adverse experience which required an adjustment to the rate of infusion during the administration of the dosimetric and therapeutic doses, respectively.

Bone marrow suppression is the dose-limiting toxicity and a total body dose of 75 cGy was determined to be the maximum tolerated dose for a non-myeloablative regimen in patients previously treated with chemotherapy. An absolute neutrophil count (ANC) of <100 cells/mm^3 has occurred in 3% of patients, and an ANC of < 1,000 cells/mm^3 has occurred in 47% of patients. A platelet count of < 10,000 cells/mm^3 has occurred in 5% of patients, and a platelet count of < 50,000

cells/mm^3 has occurred in 37% of patients. A hemoglobin of < 6.5 g/dL has occurred in 4% of patients, and a hemoglobin of < 8.0 g/dL has occurred in 12% of patients. A white blood cell count of < 2,000 cells/mm^3 has occurred in 41% of patients. The median nadirs were 62,000 cells/mm^3 for platelet count, 1,000 cells/mm^3 for ANC, and 11.1 gm/dL for hemoglobin. Blood count nadirs (which occur approximately 4 to 6 weeks after therapy) were higher and the time to recovery shorter in patients who were less heavily pretreated. The need for hematologic supportive care, which included transfusions and colony stimulating factors and were used at the discretion of the investigators, ranged from 0% in previously untreated patients to 24% in patients having received 4 or more prior therapies and was 18% overall. The frequency of HAMA-positivity was related to the extent of prior therapy; 38% in previously untreated patients versus 4% in previously treated patients (≥ 1 prior therapies). Thyroid function has been followed long-term and elevated thyroid stimulating hormone has been noted in 5 out of 106 (4%) of patients. Four of these patients have been started on oral thyroid supplementation, although clinical hypothyroidism has not been diagnosed. No significant changes in serum immunoglobulins have occurred post-treatment. Four patients developed a myelodysplastic syndrome or acute myelocytic leukemia in long-term follow-up.

The safety of Iodine-131 anti-B1 antibody in pregnant or nursing women has not been proven. Women of child-bearing potential should only be treated after negative pregnancy tests have been confirmed.

c. PHARMACOLOGY

Pharmacokinetics: After IV administration, a two compartmental model best fit the data with a median terminal half-life of 70.4 hours. The mean clearance was 97.9 ± 109.2 mL/hr (mean ± standard deviation). Dose-dependent pharmacokinetics were observed with a larger area under the curve (AUC), slower clearance, longer terminal half-life, and smaller volume of distribution at steady state observed with increasing predose levels of anti-B1 antibody. The route of excretion was renal with 65 ± 13% of the injected dose recovered in the urine over the initial 5 day time period. The mean total body effective half-lives were 65.2 ± 12.5 and 65.8 ± 12.9 hours by sodium iodide probe counts and gamma camera counts, respectively. Organ doses were modest and below normal tissue tolerances. The mean splenic dose was 399 ± 215 cGy/75 cGy total body dose (TBD). The kidney received 630 ± 201 cGy/75 cGy TBD. The cGy doses to other normal tissues from a 75 cGy whole body dose were quite modest, with the liver and the lungs receiving an average dose of 256 ± 80 cGy and 182 ± 58 cGy, respectively. The mean bladder wall dose was 202 ± 49 cGy/75 cGy TBD, the mean bone marrow dose was 103 ± 15 cGy/75 cGy TBD, and the mean blood dose was 369 ± 97 cGy/75 cGy TBD.

Formulation: Anti-B1 Antibody and Iodine-131 Anti-B1 Antibody:

Anti-B1 Antibody is a murine anti-human B-cell monoclonal antibody of the IgG_2a subclass. As formulated, Anti-B1 Antibody is a sterile, clear, colorless liquid supplied in a 3 ml or 20 ml glass vial stoppered with a gray silicone-coated butyl rubber stopper and capped with an aluminum crimp seal. Each single-use 3 ml vial contains not less than 2.5 ml of solution; each single-use 20 ml vial contains not less than 16.1 ml of solution. The formulation or each single-use vial is:

Protein concentration 14.0 ± 0.7 mg/ml

Potassium phosphate: 10 mM, pH 7.2 ± 0.2

Sodium Chloride: 145 mM

Maltose: 10%

Iodine-131 Anti-B1 Antibody:

The Iodine-131 Anti-B1 antibody is a sterile, colorless liquid in a glass vial stoppered with a gray silicone-coated butyl rubber stopper and capped with an aluminum crimp seal. Each vial is intended for single-use only.

The dosimetric vial contains not less than 1.4 ml of solution in a 10 ml vial consisting of:

Protein concentration 1.1 - 2.5 mg/ml

Calibrated activity: 8 - 12 mCi

Povidone: 5.5%

Ascorbic Acid: 0.1%

Potassium phosphate: 12.5 mM, pH 6.8 - 7.2

Sodium Chloride: 0.9%

Maltose: 1 - 2%

OR, the dosimetric vial may contain not less than 20.0 ml of solution in a 30 ml vial consisting of:

Protein concentration 0.10 - 0.25 mg/ml

Calibrated activity: 12 - 18 mCi

Povidone: 5.5%

Ascorbic Acid: 0.1%

Potassium phosphate: 12.5 mM, pH 6.5 - 7.2

Sodium Chloride: 0.9%

Maltose: 1 - 2%

The therapeutic vial contains not less than 20 ml solution in a 30 ml vial consisting of:

Protein concentration: 1.1 - 2.5 mg/mL

Calibrated activity: 112 - 168 mCi

Povidone: 5.5%

Ascorbic Acid: 0.1%

Potassium Phosphate: 12.5 mM, pH 6.5 - 7.5

Sodium Chloride: 0.9%

Maltose: 1 - 2%

Storage and Stability:

Anti-B1: Non-radioactive anti-B1 will be shipped overnight as needed to the study site. Anti-B1 must be stored at 2 - 8°C in a secure area until it is needed for use. The vials are single-use as they do not contain preservative.

I-131-Anti-B1: The lead pot containing Iodine-131 anti-B1 must be stored in a freezer until it is thawed for administration to the patient. See the section on Thawing for Administration in the User's Instructions for complete instructions on the procedure for thawing the vials. Allow approximately 20 minutes for thawing of the 10 ml dosimetric vial and approximately 60 minutes for thawing of the 30 ml dosimetric or therapeutic vial. The thawed vial may be refrigerated at 2 - 8°C for up to 6 hours. The product must be administered to the patient within 72 hours of the calibration date and time specified on the product label.

Administration: See section 7.5c for detailed administration instructions for both unlabeled and I-131-labeled anti-B1.

Supplier: Coulter Pharmaceutical, Inc. will supply anti-B1 and I-131-anti-B1 antibody free of charge for this study. The latter will be labeled and distributed by MDS Nordion, Inc. of Canada. Any questions regarding Iodine-131 anti-B1 antibody may be directed to:

David Magnuson, Pharm.D.
Coulter Pharmaceutical, Inc.
600 Gateway Boulevard
South San Francisco, CA 94080
Phone: 650/553-1931

The unlabeled Anti-B1 Antibody will be provided by CPI, South San Francisco, California at no cost. Ordering of unlabeled Anti-B1 Antibody will be performed by faxing a completed Clinical Site Order Form for Non-Radiolabeled Anti-B1 Antibody (FRM021, see Appendix 19.4a) to CPI's Clinical Research Department [fax number: 650/553-1890]. Anti-B1 Antibody must be ordered by Thursday, 4:00 p.m. Eastern Time (EST) prior to the treatment week.

The dosimetric and therapeutic vials of Iodine-131 Anti-B1 Antibody must be ordered separately by faxing a completed form Clinical Site Order Form for Iodine-131 Anti-B1 Antibody (FRM023, see Appendix 19.4b) to CPI's Clinical Research Department [fax number: 650/553-1890] by 4:00 p.m. EST on a Thursday in order to receive the product by Tuesday of the following week (i.e., 5 days later). In addition, the site must phone CPI [phone number 650/553-1886] and leave a message notifying them that an order has been faxed. The product will be shipped frozen on dry ice and will be sent to the site using priority overnight delivery. Upon receipt, immediately follow the section on Procedure for Opening in the User's Instructions. For questions related to radiolabeled antibody ordering, call the Coulter Pharmaceutical, Inc. Hot-Line at 650/553-1886 (Teresa White).

NOTE: Institutions must be approved by Coulter Pharmaceutical, Inc. and receive an on-site training session prior to administering I-131 labeled anti-B1 antibody. (See Section 15.1 for specific instructions.)

3.6 Rituximab Chimeric Monoclonal anti-CD20 Antibody (IDEC-C2B8) (Rituxan®) (NSC-687451)

a. DESCRIPTION

Rituximab is a mouse/human chimeric monoclonal antibody consisting of human IgG1 heavy and kappa light chain constant regions with murine variable regions from the murine IgG1 kappa anti-human CD20 monoclonal antibody rituximab. The rituximab antibody is produced by a Chinese hamster ovary transfectoma.

b. TOXICOLOGY

Human Toxicology: Single doses of up to 500 mg/m^2 and weekly x 4 doses of 375 mg/m^2 have been administered without dose limiting toxicity. Adverse events are most common during the initial antibody infusion and usually consist of grade I or 2 fever (73%), asthenia (16%) chills (38%) nausea (19%), vomiting (11%), rash (14%) and tumor site pain (3%). Grade 1 or 2 hypotension (8%) may be treated with IV fluids. Hematologic toxicity is usually mild and reversible. Transient decreases in the WBC or platelet count have been observed - especially in patients with high levels of circulating tumor cells or bone marrow involvement. Two patients have had late-onset Grade 4 neutropenia at four and ten months that was attributed to an unknown cause, was transient and resolved. Infections (grade 1 and 2) have not been related to dose level. Symptoms are generally associated with the initial antibody infusions and diminish in frequency with each successive infusion.

Infusion Reaction: An infusion-related symptom complex consisting of fever and chills/rigors has occurred in the majority of patients during the first rituximab infusion. Other frequent infusion-related symptoms include nausea, urticaria, fatigue, headache, pruritis, bronchospasm, dyspnea, sensation of tongue or throat swelling (angioedema), rhinitis, vomiting, hypotension, flushing, and pain at disease sites. These reactions generally occurred within 30 minutes to 2 hours of beginning the first infusion, and resolved with slowing or interruption of the rituximab infusion and with supportive care (IV saline, diphenhydramine, and acetaminophen).

Tumor Lysis Syndrome: Rituximab rapidly decreases benign and malignant CD20 positive cells. Tumor lysis syndrome has been reported to occur within 12 to 24 hours after the first rituximab infusion in patients with high numbers of circulating malignant lymphocytes. Patients with high tumor burden (bulky lesions) may also be at risk. Patients at risk for developing tumor lysis syndrome should be followed closely and appropriate laboratory monitoring performed.

The safety of rituximab chimeric Monoclonal anti-CD20 Antibody in pregnant or lactating women has not been proven.

c. PHARMACOLOGY

Kinetics: In prior studies patients treated at the 375 mg/m^2 dose levels exhibited detectable antibody concentrations throughout the treatment period. Most patients exhibited increasing pre-infusion antibody concentrations with each subsequent infusion. In nine patients, the $T_{1/2}$ following the first antibody infusion was 59.8 hours (11.1-104.6 hr) with a C_{max} of 271 µg/ml. Following the fourth antibody infusion when circulating B cells had been depleted and antigenic sites coated, the $T_{1/2}$ was 174 hr (26.4-442.3 hr) and C_{max} 496.7 µg/ml.

Formulation: Rituximab antibody will be provided in 100 mg (10 mL) and 500 mg (50 ml) pharmaceutical grade vials at a concentration of 10.0 mg of protein per mL (actual concentration should be noted on the product label).

Storage and Stability: Rituximab should be stored at 2 - 8°C. Do not freeze or store at room temperature. The product is a protein- HANDLE GENTLY AND AVOID FOAMING. The avoidance of foaming during product handling, preparation and administration is important, as foaming may lead to the de-naturing of the product proteins.

Administration: The total amount of rituximab needed for a patient's entire six infusions (one course) will be determined AT STUDY ENTRY. A single dose of 375 mg/m^2 will be based upon the patient's actual body surface area calculated during the baseline evaluation. The dose level of rituximab will not be adjusted.

Prepare the rituximab infusion solution as follows:

a. If a delay in administration of the infusion occurs after the product is prepared, the properly identified container may be kept refrigerated at 2 - 8°C for up to six hours.

b. Use sterile, non-pyrogenic, disposable containers, syringes, needles, stopcocks and transfer tubing, etc.

c. Transfer of the rituximab from the glass vial should be made by using a suitable sterile graduated syringe and large gauge needle.

d. Transfer the appropriate amount of rituximab from the graduated syringe, into a partially filled IV pack containing sterile, pyrogen-free 0.9% sodium chloride solution, USP (saline solution). The final concentration of rituximab in saline solution should be a maximum of 1 mg/ml. Mix by inverting the bag gently. DO NOT USE A VACUUM APPARATUS to transfer the product from the syringe to the plastic bag.

e. Place an IV administration set into the outflow port of the bag containing the infusion solution.

f. NOTE: DO NOT USE evacuated glass containers which require vented administration sets because this causes foaming as air bubbles pass through the solution.

The administration of rituximab will be accomplished by slow IV infusion. CAUTION: DO NOT ADMINISTER AS AN INTRAVENOUS PUSH OR BOLUS. IV pumps such as the IMED 960 may be used with the rituximab infusion. DO NOT INFUSE CONCOMITANTLY with another IV solution or IV medications. Prime the line with the rituximab solution such that approximately 30 mL are delivered. This will saturate the filter and tubing.

Supplier: This drug is commercially available for purchase by the third party. This drug will not be supplied by the NCI.

4.0 STAGING CRITERIA

4.1 The Ann Arbor staging criteria will be used. Stage is determined based on extent of disease at the time of diagnosis. Bulky disease determination is made after surgical resection, if applicable.

4.2 Ann Arbor Classification (AJCC Manual for Staging of Cancer, 5th ed., 1997)

STAGE II	Involvement of two or more lymph node regions on the same side of the diaphragm (II) or localized involvement of a single associated extralymphatic organ or site and its regional nodes with or without other lymph node regions on the same side of the diaphragm (II_E).
STAGE III	Involvement of lymph node regions on both sides of the diaphragm (III) that may also be accompanied by localized involvement of an extralymphatic organ or site (III_E) by involvement of the spleen (III_S) or both (III_{SE}).
STAGE IV	Disseminated (multifocal) involvement of one or more extralymphatic organs with or without associated lymph node involvement, or isolated extralymphatic organ involvement with distant (non-regional) nodal involvement).

A = Asymptomatic

B = Fever, sweats, weight loss > 10% of body weight

4.3 "Bulky" is defined as a mediastinal mass > 1/3 of the maximum chest diameter (i.e., internal dimension of the thoracic cavity measured at its widest point per radiograph) or any other mass ≥ 10 cm in maximum diameter.

5.0 ELIGIBILITY CRITERIA

Each of the criteria in the following section must be met in order for a patient to be considered eligible for registration. Use the spaces provided to confirm a patient's eligibility. For each patient, this section may be photocopied, completed and submitted to the Statistical Center (see Section 14.4e) in lieu of comprehensive documentation of these items on the initial flow sheet.

SWOG Patient No. ____________________

Patient's Initials (L, F, M) ____________________________

______ 5.1 **The registering institution must have submitted the Nuclear Medicine/Radiation Oncology Investigator Questionnaire (Appendix 19.5) to Coulter Pharmaceutical, Inc. and been approved by Coulter for this study. (The approval process is required only for the FIRST patient registered to this study by any one institution.) Institutions previously approved by Coulter for the Southwest Oncology Group study, S9911, need not repeat the approval process.)**

Approved (circle one) YES NO Date of Approval ____________________

______ 5.2 All patients must have previously untreated follicular Non-Hodgkin's lymphoma (Grade I, II, or III).

______ 5.3 Lymphomas must express the CD20 antigen as demonstrated by either flow cytometry or immunoperoxidase staining of paraffin sections using anti-CD20 antibodies. A report providing confirmation of CD20 expression must be submitted per Section 14.4.

______ 5.4 Patients must have Stage III, Stage IV, or bulky Stage II extent of disease by the Ann Arbor classification (see Section 4.0).

______ 5.5 All patients must have bidimensionally measurable disease (as defined in Section 10.1a) documented within 28 days prior to registration. Patients with non-measurable disease (as defined in Section 10.1b) in addition to measurable disease must have all non-measurable disease assessed within 42 days prior to registration.

Date measurable disease assessed ____________

Date non-measurable disease assessed ____________

______ 5.6 **Pathology Review:** Adequate sections from the original diagnostic specimen or core needle biopsies which are large enough to show the architecture (bone marrow biopsies and needle aspirates are insufficient) must be available for submission as outlined in Section 12.0.

Patients are also eligible to register to and submit serum for SWOG-8947 and register to and submit tissue for SWOG-8819.

______ 5.7 Patients must have a bone marrow aspirate and biopsy performed within 42 days prior to registration.

Date of bone marrow biopsy/aspirate ____________ Positive/Negative (circle one)

______ 5.8 **Pretreatment specimens of heparinized marrow (2-3cc) for t(14;18)/bcl2 assessment must be submitted to Dr. Rita Braziel per Section 15.2**

Date specimen submitted ____________________

SWOG Patient No. ______________________

Patient's Initials (L,F,M) ______________________

______ 5.9 Patients must have a chest x-ray or CT scan of the chest and a CT scan of the abdomen and pelvis within 28 days prior to registration.

Chest x-ray or CT Scan Date ____________ Positive/Negative (circle one)

Abdomen and Pelvis CT Scan Date ____________ Positive/Negative (circle one)

______ 5.10 Patients must have a β_2 microglobulin performed within 28 days prior to registration.

β_2 microglobulin value ____________ IULN ____________

Date ____________

______ 5.11 Patients must not have clinical evidence of central nervous system involvement by lymphoma. Any laboratory tests that are performed to assess central nervous system involvement must be negative within 42 days of registration.

Date tests performed (if needed) ______________________

______ 5.12 Patients must not have received prior chemotherapy for lymphoma. Patients must not have received prior monoclonal antibodies for malignant disease.

______ 5.13 Patients must not have received prior radiation therapy for lymphoma.

______ 5.14 Patients must not have a history of hypersensitivity to iodine.

______ 5.15 All patients must have a Zubrod performance status of 0, 1 or 2 (see Section 10.4).

______ 5.16 Patients must have passed their 18th birthday.

______ 5.17 Patients must have granulocytes >1,500 /µl and platelets > 100,000/µl within 28 days prior to registration.

Granulocytes ______________ Date ______________

Platelets ______________ Date ______________

______ 5.18 Patients must have fewer than 5000 circulating lymphoid cells per µl within 28 days prior to registration.

Circulating lymphoid cells/µl ____________ Date ______________

______ 5.19 Patients with a history of impaired cardiac status (including history of severe coronary artery disease, cardiomyopathy, congestive heart failure or serious arrhythmia) are not eligible. If the patient's history is questionable, a MUGA Scan must be obtained within 42 days prior to registration (patients with ejection fractions < institutional lower limit of normal will not be eligible).

MUGA scan (if performed) ______________________

ILLN____________ Date obtained ____________

SWOG Patient No. ______________________

Patient's Initials (L,F,M) ______________________

______ 5.20 Patients known to be HIV-positive are not eligible. (For justification of this exclusion, see Section 2.0.)

______ 5.21 Pregnant or nursing women may not participate. Women or men of reproductive potential must agree to use an effective contraceptive method from the time of registration to 6 months after receiving the Iodine-I31 Anti-B1 Antibody. (For justification of this exclusion, see Section 2.0 - 3.0)

______ 5.22 No prior malignancy is allowed except for adequately treated basal cell or squamous cell skin cancer, in situ cervical cancer, or other cancer for which the patient has been disease-free for five years.

5.23 If Day 28 or 42 falls on a weekend or holiday, the limit may be extended to the next working day. **In calculating days of tests and measurements, the day a test or measurement is done is considered Day 0. Therefore, if a test is done on a Monday, the Monday four weeks later would be considered Day 28. This allows for efficient patient scheduling without exceeding the guidelines.**

______ 5.24 All patients must be informed of the investigational nature of this study and give written informed consent in accordance with institutional and federal guidelines.

______ 5.25 At the time of patient registration, the treating institution's name and ID number must be provided to the Statistical Center in order to ensure that the current (within 365 days) date of institutional review board approval for this study has been entered into the data base.

6.0 STRATIFICATION FACTORS

A dynamic allocation scheme will be used to randomize patients to the three arms at registration. Patients will be balanced with respect to the following stratification factor:

β_2 microglobulin > IULN: yes vs. no

7.0 TREATMENT PLAN

For treatment or dose modification related questions, please contact Dr. Press at 206/598-4938 or Dr. Maloney at 206/667-5616.

7.1 Good Medical Practice

The following pre-study tests should be obtained within 28 days prior to initial registration in accordance with good medical practice. Results of these tests do not determine eligibility and minor deviations would be acceptable if they do not impact on patient safety in the clinical judgement of the treating physician. The Study Coordinator must be contacted if there are significant deviations, in the opinion of the treating investigator, in the values of these tests.

a. A pretreatment serum bilirubin < 2 x the institutional upper limit of normal and a serum creatinine < 2 x the institutional upper limit of normal.

b. A serum level of LDH (lactose dehydrogenase) should be obtained.

c. An EKG should be performed within 42 days prior to registration and it should be free of any arrhythmias (excluding sinus arrhythmia or infrequent premature ventricular contractions).

d. Urinalysis, uric acid, and SGOT and/or Alkaline Phosphatase be should performed in order to assess potential treatment-related toxicities.

7.2 At the time of registration, patients will be randomized to one of the three treatment arms described below.

NOTE: Institutions with patients randomized to Arm 3 must make arrangements with Coulter immediately for on-site training in the administration of the I-131 Anti-B1 antibody (see in Sections 7.5 and 15.1). (The on-site training is required only for the FIRST patient randomized to Arm 3 of this study by any one institution. Institutions that have been previously undergone training by Coulter for Southwest Oncology Group study, S9911, need not repeat the training.)

7.3 **Arm 1 - CHOP Chemotherapy:** Treatment with CHOP as described below will be administered every 21 days for a maximum of 6 cycles. Patients with progressive disease at any time while receiving treatment will be removed from protocol treatment (see Section 7.9a). All patients will receive identical starting doses regardless of marrow status.

TABLE 1 - CHOP CHEMOTHERAPY:

DRUG	DOSE	ROUTE	DAYS	RE-TX Interval
Cyclophosphamide	750 mg/m^2	IV infusion over 15 minutes	1	q 21 days for up to 6 cycles
Doxorubicin	50 mg/m^2	Slow IV injection	1	q 21 days for up to 6 cycles
Vincristine	1.4 mg/m^2 (max 2.0 mg)	Slow IV injection	1	q 21 days for up to 6 cycles
Prednisone*	100 mg	PO	1 - 5	q 21 days for up to 6 cycles

*Prednisone should be omitted if the patient has a history of recent active peptic ulcer disease or if peptic ulcer symptoms occur during treatment. The reason for omitting this drug must be noted on flow sheet.

Patients randomized to Arm 1 (CHOP only) should not receive any further treatment after completion of the six cycles of CHOP. NO additional therapy will be administered to these patients (interferon, rituximab, radiolabeled antibodies).

See Section 8.1 for CHOP treatment dose modifications.

7.4 **Arm 2 - CHOP + concurrent rituximab**:

a. CHOP Chemotherapy: Patients randomized to Arm 2 will be treated with six cycles of CHOP + six doses of rituximab according to the schedule described by Czuczman et al. *(20)* The CHOP chemotherapy will be administered at three week intervals as described below for a maximum of six cycles.

b. Rituximab Infusions: The six rituximab infusions will be administered concurrently with the CHOP chemotherapy as described below.

TABLE 2 - CHOP CHEMOTHERAPY PLUS RITUXIMAB INFUSIONS:

DRUG	DOSE	ROUTE	DAYS	NOTES
Cyclophosphamide	750 mg/m^2	IV infusion over 15 minutes	8, 29, 50, 71, 92, 113	
Doxorubicin	50 mg/m^2	Slow IV injection	8, 29, 50, 71 92, 113	
Vincristine	1.4 mg/m^2 (max 2.0 mg)	Slow IV injection	8, 29, 50, 71 92, 113	
Prednisone*	100 mg	PO	First 5 days of each CHOP cycle	
Rituximab Infusions #1, #2	375 mg/M^2	Slow IV (see Section 7.4c for dose rate)	1, 6	CHOP Cycle 1 only
Rituximab Infusions #3, #4	375 mg/M^2	slow IV (see Section 7.4c for dose rate)	48, 90 (48 hours before beginning CHOP cycle)	CHOP Cycles 3 and 5 ONLY
Rituximab Infusions #5, #6	375 mg/M^2	slow IV (see Section 7.4c for dose rate)	134,141	3 and 4 weeks FOLLOWING CHOP Cycle 6

*Prednisone should be omitted if the patient has a history of recent active peptic ulcer disease or if peptic ulcer symptoms occur during treatment. The reason for omitting this drug must be noted on flow sheet.

Rituximab infusions #1 and #2 will be administered on Days 1 and 6 before the first CHOP cycle, with CHOP chemotherapy being administered on Day 8.

Rituximab infusions #3 and #4 will be administered on Days 48 and 90 (two days before the third and fifth cycles of CHOP).

Rituximab infusions #5 and #6 will be given after Cycle 6 of CHOP on Days 134 and 141, respectively (three and four weeks, respectively after Cycle 6 of CHOP).

c. <u>Although patients with non-Hodgkin's lymphoma that are eligible for this study should not have markedly elevated numbers of circulating malignant cells, the following should be noted.</u> For some patients, tumor lysis syndrome has been reported to occur within 12 - 24 hours after the first rituximab infusion. Prophylaxis to prevent the rare event of tumor lysis syndrome in patients with bulky tumors (> 10 cm) or with markedly elevated numbers of circulating malignant cells is recommended.

Oral premedication (2 tablets [350 mg]) of acetaminophen and 50 to 100 mg oral diphenhydramine hydrochloride) may be administered 30 - 60 minutes prior to starting each infusion of rituximab. A peripheral or central intravenous (IV) line will be established. During the rituximab infusion, the patients vital signs (blood pressure, pulse, respiration, temperature) should be monitored every 15 minutes x 4 until stable and then hourly until the infusion is discontinued. Available at bedside prior to rituximab administration will be ephinephrine for subcutaneous injection, diphenhydramine hydrochloride for intravenous injection, and resuscitation equipment for the emergency management of anaphylactoid reactions. <u>The initial dose rate at the time of the first rituximab infusion should be 50 mg/hr for the first hour</u>. If no toxicity is seen, the dose rate may be escalated

gradually (50 mg/hr increments at 30-minute intervals) to a maximum of 300 mg/hr. If the first dose of rituximab is well tolerated, the starting flow rate for the administration of subsequent doses will be 100 mg/hr, then increased gradually (100 mg/hr increments at 30-minute intervals) not to exceed 400 mg/hr.

Precautionary hospitalization for patients experiencing severe symptoms or infusion reactions which do not resolve after discontinuation or completion of the cycle is recommended.

See Section 8.1 for CHOP treatment dose modifications.

See Section 8.2 for rituximab dose modifications.

7.5 Arm 3 - CHOP followed by I-131 antibody: **Institutions must have completed an on-site training session with Coulter representatives to review the details of drug administration and dosimetry prior to treating a patient with Iodine-131 anti-B1 antibody. The training session must occur prior to the dosimetric infusion of I-131-tositumomab, and the date of the training session must be noted on the Flow Sheet. For subsequent patients, the original training session date must still be noted on the Flow Sheets. To arrange for a training session, contact Teresa White at Coulter Pharmaceutical at 650/553-1886 (see Section 15.1). (The training session must be performed only for the FIRST patient randomized to ARM 3 of this study at any one institution.) Institutions that have undergone on site Coulter training for Southwest Oncology Group study S9911 need not repeat the training.**

a. CHOP chemotherapy: Patients randomized to Arm 3 will receive six cycles of CHOP chemotherapy at three week intervals as described in Table 3 of Section 7.5c.

b. Re-evaluation: Patients on Arm 3 will be re-evaluated no earlier than 4 weeks but no later than 8 weeks after the completion of CHOP chemotherapy (see Section 9.3). **The following conditions must be met prior to proceeding with I-131 - tositumomab antibody treatment. Patients who do not qualify for I-131 tositumomab antibody treatment upon re-evaluation will be removed from protocol treatment.**

1. The training session with Coulter Pharmaceutical must have been completed.

2. Patients must have no more than 25% of the intratrabecular marrow space involved by lymphoma in bone marrow biopsy specimens as assessed microscopically after completion of 6 cycles of CHOP chemotherapy. Bilateral posterior iliac crest core biopsies are required if the percentage of intratrabecular space involved exceeds 10% on a unilateral biopsy. The mean of bilateral biopsies must be no more than 25%. The procedure for bilateral bone marrow biopsy analysis of marrow involvement is included in Appendix 19.3.

3. Patients must have granulocytes ≥ 1,500 /μl and platelets ≥ 100,000/μl within 14 days of the planned dosimetric infusion. Also, patients must not have active obstructive hydronephrosis.

c. **TABLE 3** - CHOP CHEMOTHERAPY PLUS TOSITUMOMAB INFUSIONS:

Administration of I-131-Tositumomab: Tositumomab will be administered after the completion of CHOP chemotherapy and re-evaluation as described below.

DRUG	DOSE	ROUTE	DAYS	RE-TX Interval
Cyclophosphamide	750 mg/m^2	IV infusion over 15 minutes	1	q 21 days for up to 6 cycles
Doxorubicin	50 mg/m^2	Slow IV injection	1	q 21 days for up to 6 cycles
Vincristine	1.4 mg/m^2 (max 2.0 mg)	Slow IV injection	1	q 21 days for up to 6 cycles
Prednisone*	100 mg	PO	1 - 5	q 21 days for up to 6 cycles
Unlabeled Anti-B1 Antibody **	450 mg	IV over 1 hour	Day 134^f	
Dosimetric Dose$^+$	35 mg	IV over 20 minutes	Day 134	
Unlabeled Anti-B1 Antibody**	450 mg	IV over 1 hour	Day141$^{\surd}$	
Radioimmunotherapy Dose$^+$	35 mg	IV over 20 minutes	Day141$^{\surd}$	

* Prednisone should be omitted if the patient has a history of recent active peptic ulcer disease or if peptic ulcer symptoms occur during treatment. The reason for omitting this drug must be noted on flow sheet.

** Patients must receive 450 mg of unlabeled Anti-B1 Antibody (to 50 mL using 0.9% sodium chloride) prior to both the dosimetric and radioimmunotherapy dose. Patients must be premedicated with acetaminophen 650 mg po and diphenhydramine 50 mg po prior to administration of unlabeled anti-B1 antibody (see Sections 7.5c.2ii and 7.5c.3ii).

\+ Patients must receive SSKI, Lugol's Solution or Potassium Iodide at least 24 hours prior the first infusion of the dosimetric dose. Treatment will continue until 14 days after the last infusion of the radioimmunotherapy dose (see Section 7.5c.2iii).

^{f} Ideally, the dosimetric infusion will be given on Day 134 and the therapeutic infusion on Day 141. Due to the logistics of ordering, receiving and administering I-131-tositumomab, a 4 week period of flexibility after Day 134 will be allowed within which the radiolabeled antibody therapy may be given.

√ See Section 7.5c.3ii.

1. Preparation, Dosing, and Administration:

Patients will undergo two phases of tositumomab administration. The first phase, termed "dosimetric dose", involves the intravenous (IV) administration of a low-radioactive dose (five mCi) of Iodine-131 anti-B1 antibody for the purpose of determining the rate of total body clearance of radioactivity (residence time) so that a total body radiation dose can be calculated (see Appendix 19.2). The calculated total body radiation dose per mCi administered can then be used to determine how many mCi of Iodine-131 conjugated with anti-B1 antibody will be required to deliver the total body radiation dose in the second phase of the study, termed "radioimmunotherapy dose." Both the dosimetric dose and the

radioimmunotherapy dose will be immediately preceded by an infusion of 450 mg unlabeled anti-B1 antibody (see Sections 7.5c2.iv and 7.5c.3ii).

Administration of the radiolabeled anti-B1 antibody will be performed by personnel authorized to deliver such doses of radioisotope to patients. Special radiation precautions will be used during and after the administration of the radioimmunotherapy dose, as required by the national and/or regional regulations for the radiopharmaceutical industry. Restrictions on patient contact with others will be set in accordance with these regulatory guidelines [Nuclear Regulatory Commission (NRC) and state laws]. The dosimetric and therapeutic doses may be given as either an outpatient or inpatient procedure depending on current NRC and state regulations.

2. Dosimetric Dose:

i. Preparation of Unlabeled Anti-B1 Antibody

To prepare unlabeled anti-B1 antibody for administration to patients, 450 mg unlabeled anti-B1 antibody is sterilely-removed from the product vials and diluted to 50 mL using 0.9% sodium chloride for injection.

ii. Preparation of Dosimetric Dose (i.e., Tracer Dose)

To prepare the dosimetric dose, an amount of anti-B1 antibody (33 - 34 mg) is added to the trace-labeled antibody preparation (1-2 mg of anti-B1 antibody radiolabeled with 5 mCi of 131-Iodine) sufficient to result in a final amount of 35 mg of anti-B1 antibody. This latter preparation is then diluted to a final volume of 30 mL using 0.9% sodium chloride for injection.

iii. Administration of Saturated Solution Potassium Iodide (SSKI), Lugol's Solution, or Potassium Iodide Tablets

Patients will be treated with either saturated solution of potassium iodide (SSKI) four drops po tid, Lugol's solution 20 drops po tid, or potassium iodide tablets 130 mg po qd starting at least 24 hours prior to the first infusion of the Iodine-131 Anti-B1 Antibody (i.e., the dosimetric dose) and continuing for 14 days following the last infusion of Iodine-131 Anti-B1 Antibody (i.e., therapeutic dose). The SSKI or Lugol's solution may be given with juice or cola to mask taste. In no instance should a patient receive the dosimetric dose of Iodine-131 Anti-B1 Antibody if they have not yet received at least 3 doses of SSKI, three doses of Lugol's solution, or one 130 mg potassium iodide tablet (at least 24 hours prior to the dosimetric dose). Patients should be monitored for compliance with regard to SSKI, Lugol's solution, or potassium iodide tablets.

All concomitant medications must be recorded on the Flow Sheets.

iv. Administration of Dosimetric Dose

On Day 1, patients will receive the intravenous (IV) administration of 450 mg unlabeled anti-B1 antibody followed by the IV administration of the dosimetric dose (five mCi of Iodine-131 anti-B1 antibody). The unlabeled antibody must be administered through an in-line filter [Abbott lab filter set with 0.22 micron filter and injection site-15 inch option-lock (part 2679)]. These filters will be provided by Coulter Pharmaceutical, Inc. The in-line filter may remain connected to or removed from the infusion line following the unlabeled antibody. A new filter should not be added for the radiolabeled infusion. Thirty to sixty minutes before the unlabeled anti-B1 antibody infusion, patients will be premedicated with acetaminophen 650 mg po and diphenhydramine 50 mg po (unless the patient is hypersensitive to acetaminophen or diphenhydramine). Unlabeled anti-B1 antibody (see Section 7.5c.2ii) will then be given as an intravenous (IV) infusion over 1 hour or longer depending on infusion-related adverse experiences. The dosimetric dose (see Section 7.5c2ii) will be given as an intravenous infusion over 20 minutes. At the end of the infusion of the dosimetric dose, the syringe or IV bag must be refilled with 0.9% sodium chloride and the contents infused over a period of 10 minutes. Vital signs must be taken every 15 minutes during each of the anti-B1 antibody infusions.

v. Whole Body Dosimetry

Whole body dosimetry will be performed separately for each patient as described in Appendix 19.2, using the worksheets provided. For all patients, whole body anterior gamma camera scans will be obtained within one hour after the completion of the administration of the dosimetric dose on Day 1 (before any urination), and then either on Day 3, 4, or 5 (after urination) and again on either Day 7 or 8 (after urination) using a gamma camera with appropriate medium- or high-energy collimator. The anterior whole body scans will be obtained at 30 -100 cm/minute scan speed. Anterior whole body counts, anterior background counts, and anterior counts of a calibrated standard will be obtained and recorded. All static and whole body scan images for dosimetry will be retained electronically for submission upon request to Coulter Pharmaceutical, Inc. or its designee.

The above determined counts will be used to calculate the activity to be administered to deliver 75 cGy (unless adjusted for obesity and/or platelet count - see below). The mCi dose will be calculated as described in Appendix 19.2 and accompanying worksheets.

Dose Adjustments based on weight and platelet counts:

For excessively obese patients, the calculations to determine the Iodine-131 anti-B1 antibody activity to administer will be performed using an upper limit of mass (maximum effective mass) based upon height and gender (see Table 1, Appendix 19.2e).

The administered activity (mCi of Iodine-131 anti-B1 antibody) for patients with platelet counts of 100,000 - 149,999 cells/mm^3 will be adjusted to deliver 65 cGy, with additional adjustment of activity for obesity, if indicated. **Iodine I-131 antibody should not be given if platelets are less than 100,000/mm^3.**

The dose calibrator used for measuring the mCi of activity of Iodine-131 anti-B1 antibody to be administered to the patient must be appropriately calibrated.

The dosimetry worksheets for the first 3 patients at each clinical site must be submitted to Coulter Pharmaceutical, Inc. by fax to confirm that the calculations were performed correctly (Fax: 650/553-1890). A dosimetry hotline will be maintained by Coulter to assist in calculation of the proper therapeutic dose (650/553-1859, Teresa White).

3. Radioimmunotherapy Dose:

i Preparation

To prepare the radioimmunotherapy dose, an amount of anti-B1 antibody is added to the radiolabeled preparation [anti-B1 antibody labeled with enough 131 Iodine to administer the specified whole body radiation dose calculated for the patient from the dosimetric dose] sufficient to result in a final amount of 35 mg of anti-B1 antibody, unless the amount of anti-B1 antibody in the radiolabeled preparation is already ≥ 35 mg. This latter preparation is then diluted to a final volume of 30 mL using 0.9% sodium chloride for injection. In rare cases, greater than 30 ml of Iodine-131 anti-B1 antibody will be required and the dose will then be prepared in 60 ml.

ii. Administration

The radioimmunotherapy dose is to be given 7 days after the administration of the dosimetric dose (may be delayed but no longer than 14 days after dosimetric dose). **Those patients who experienced an anaphylactic response or serious adverse experience felt to be related to study drug during or following trace-labeled antibody administration will be removed from protocol treatment.** Patients will be premedicated with acetaminophen and diphenhydramine as they were prior to the dosimetric dose. Patients should also still be receiving SSKI or Lugol's solution as described in Section 7.5c.2iii. The unlabeled antibody must be administered through an in-line filter [Abbott Lab filter set with 0.22 micron filter and y injection site - 15 inch option-lock (part 2679)]. These filters will be provided by Coulter Pharmaceutical, Inc. The in-line filter may remain connected to or removed from the infusion line following the unlabeled antibody. A new filter should not be added for the radiolabeled infusion. Unlabeled anti-B1 antibody will then be given as an intravenous (IV) infusion over 1 hour or longer depending on infusion-related adverse experiences. The radioimmunotherapy dose will be given as an intravenous infusion over 20 minutes. At the end of the infusion of the radioimmunotherapy dose, the syringe or IV bag must be refilled with 30 ml 0.9% sodium chloride and the contents infused over a period of 10 minutes. Vital signs must be taken every 15 minutes during each of the anti-B1 antibody infusions.

7.6 CNS Prophylaxis: There will be no CNS prophylaxis on any of the three treatment arms of this protocol.

7.7 Allopurinol: To prevent the rare event of tumor lysis syndrome in patients with bulky tumors (> 10 cm), oral or IV fluid intake in excess of 2,000 ml daily is encouraged during therapy for all patients on the study. In addition, the routine administration of allopurinol (300 mg/d) is also recommended prior to and during the first cycle of therapy on all three arms of the study. If rash occurs, allopurinol can be discontinued.

7.8 Restaging: All patients will be assessed for response 200 and 365 days after initiation of therapy and then every six months as specified on the study calendars (see Section 9.0). Day 200 was chosen for restaging because this uniform assessment timepoint should allow evaluation of patients on all three arms of the protocol at least four weeks after completion of therapy.

Restaging will include history, physical examination, complete blood cell counts with differential, chest X-ray or Chest CT scan (depending on which was done prestudy), abdominal and pelvic CT scans, and bone marrow aspiration and biopsy (only required at Day 200 if marrow was initially involved with lymphoma). For all patients who do not progress, a bone marrow aspirate and biopsy is required at Day 365 for t(14;18) studies.

7.9 Criteria for Removal from Protocol Treatment

a. Documented progression of disease as defined in Section 10.2f.

b. Development of unacceptable toxicity, as defined in Section 8.0.

c. Failure to meet criteria for I-131 antibody administration following completion of CHOP chemotherapy (for Arm 3 patients only, see Section 7.5b).

d. Completion of protocol treatment.

e. The patient may withdraw from the study at any time for any reason.

7.10 All reasons for discontinuation of treatment must be documented in the flow sheets.

7.11 All patients will be followed until death.

8.0 TOXICITIES TO BE MONITORED AND DOSAGE MODIFICATIONS

8.1 CHOP Dose Modification (for Arms 1-3): In the case of multiple toxicities, dose modifications should be based on the most severe dose-limiting toxicity.

a. Hematologic Toxicity: The CHOP regimen should be given as described in Section 7.0 if the granulocytes are > 1,500 cells/μl and the platelets are > 100,000 cells/μl by the time the next cycle is due. If the blood counts have not recovered, treatment should be delayed one week and counts repeated unless low peripheral counts are due to tumor. If, after two weeks, counts have not yet recovered, the patient should be treated at 75% of the last dose received of cyclophosphamide and doxorubicin.

Grade 3 or 4 infection (NCI Common Toxicity Criteria Version 2.X) due to chemotherapy-related neutropenia requires a decrease in the doses of cyclophosphamide and doxorubicin to 75% of the last dose received. Re-escalation is at the discretion of the treating physician. In this study, G-CSF or GM-CSF will not be administered to prevent neutropenia.

For patients who experience Grade 3 or 4 neutropenia or develop neutropenic fever between cycles of chemotherapy, G-CSF (or GM-CSF) may be added to all subsequent cycles of chemotherapy. Dose of CHOP may be re-escalated on future cycles at the discretion of the investigator.

Both growth factors are commercially available and should be purchased through third party mechanisms. The Southwest Oncology Group will not provide either G-CSF or GM-CSF for this study.

The use of G-CSF or GM-CSF must be documented on the Flow Sheets. Any toxicities associated with G-CSF or GM-CSF must also be documented on the Flow Sheets.

b. Impaired Hepatic Function: All patients with bilirubin ≤ 2 x the institutional upper limit of normal will receive a full initial dose of doxorubicin and vincristine. If the bilirubin rises to > 2 x the institutional upper limit of normal (but ≤ 5 x IULN), the doxorubicin and vincristine doses must be reduced by 50% to avoid undue hepatic toxicity. Full doses should be given once the bilirubin is ≤ 2 x the institutional upper limit of normal. If the bilirubin rises to > 5 x the institutional upper limits of normal, doxorubicin and vincristine should be discontinued for that cycle. If hepatic function has not recovered to ≤ 2 x the institutional upper limits of normal by the time the next cycle is due, then remove patient from protocol treatment. In cases of obstruction of the biliary duct by tumor mass, a biliary drainage shunt should be placed prior to chemotherapy.

Bilirubin	Doxorubicin Dose	Vincristine Dose
≤ 2 x IULN	100%	100%
> 2 - 5 x IULN	50%	50%
> 5 x IULN	0%	0%

c. Impaired Renal Function: All patients with serum creatinine levels ≤ 2 x the institutional upper limit of normal will receive full doses of all drugs. If the creatinine rises > 2 x the institutional upper limit of normal, the dose of cyclophosphamide must be reduced by 25%. Re-escalation to full dose is at the discretion of the treating physician if the serum creatinine level drops to ≤ 2 x the institutional upper limit of normal.

d. Hemorrhagic cystitis: Cyclophosphamide will be discontinued and the patient removed from protocol treatment if Grade 3 or 4 hemorrhagic cystitis resulting from this drug occurs. Adequate fluid intake and allopurinol are recommended during therapy.

e. Neuropathy: Patients experiencing Grade 3 vincristine-neuropathy (e.g., obstipation, weakness) will have the dose of vincristine reduced by 50% for all further cycles of CHOP. Patients experiencing Grade 4 vincristine neuropathy will have vincristine omitted from all future cycles of CHOP.

8.2 Rituximab Antibody Dose Modification and Cycle Delay (for Arm 2):

a. Patients may experience transient fever and rigors with infusion of chimeric anti-CD20 antibody. If Grade 3 fever (or Grade 2 fever with rigors) or Grade 2 rigors are noted, the antibody infusion should be temporarily discontinued, the patient should be observed, and the severity of the side effects should be evaluated. The patient should be treated according to the best available local practices and procedures. Following observation, when fever resolves to Grade 2 or less and rigors to Grade 1 or less, the infusion should be continued, initially, at 1/2 the previous rate. Following the antibody infusion , the IV line should be kept open for medications, as needed.

b. Hypotension, bronchospasm and angioedema have occurred as part of an infusion related symptom complex. If a Grade 3 or greater hypersensitivity/allergic reaction occurs, the rituximab infusion should be interrupted and may be resumed at a 50% reduction in rate when symptoms have completely resolved. Treatment with diphenhydramine and acetaminophen is recommended; additional treatment with broncodialators or IV saline may be used at the physician's discretion. Precautionary hospitalization for patients experiencing severe infusion symptoms which do not resolve after discontinuation of the cycle is recommended.

If there are no complications during the rituximab infusion, the IV line may be discontinued one hour after completion of the infusion. If complications occur during the rituximab infusion, the patient should be observed for two hours after the completion of the infusion. If a patient experiences a Grade 3 toxicity that persists until the next scheduled infusion, the patient must discontinue treatment until toxicities have resolved to Grade 2 or less. If treatment is delayed for more than three weeks, remove the patient from protocol treatment.

Appropriate medical therapy should be provided for patients who develop tumor lysis syndrome. Following treatment for and resolution of tumor lysis syndrome, subsequent rituximab therapy may be administered in conjunction with prophylactic therapy for this syndrome. Contact the Study Coordinator prior to resuming treatment in these patients.

8.3 Iodine-131 Anti-B1 Antibody Dose Modification and Cycle Delay (form Arm 3):

Iodine-131 should be given as specified in Section 7.5 as long as counts have recovered to granulocytes ≥ 1,500 and platelets ≥ 100,000.

a. Dose Adjustments based on weight and platelet counts:

For excessively obese patients, the calculations to determine the Iodine-131 anti-B1 antibody activity to administer will be performed using an upper limit of mass (maximum effective mass) based upon height and gender (see Table 1, Appendix 19.2e).

The administered activity (mCi of Iodine-131 anti-B1 antibody) for patients with platelet counts of 100,000 - 149,999 cells/mm^3 will be adjusted to deliver 65 cGy, with additional adjustment of activity for obesity, if indicated. **Iodine-131 antibody should not be given if platelets are less than 100,000/mm^3.**

The dose calibrator used for measuring the mCi of activity of Iodine-131 anti-B1 antibody to be administered to the patient must be appropriately calibrated.

b. Other dose adjustments:

During the administration of the unlabeled anti-B1 antibody, tracer, and radioimmunotherapeutic doses, emergency support for anaphylaxis is to be readily available, including a tray for epinephrine, diphenhydramine, hydrocortisone, a laryngoscope, and an endotracheal tube. Although acute adverse experiences occurring during the infusion or up to 24 hours after the infusion of anti-B1 antibody have been infrequent, based upon past experience, symptoms of fever, nausea, vomiting, rigors, hypotension, pruritis, erythematous rash, urticaria, mucus membrane congestion, arthralgias, and myalgias may occur. The patient should be treated according to physician's judgment. However, it is recommended that acetaminophen 650 mg po and/or diphenhydramine 50 mg po or IV be given to control these symptoms if they occur. Severe rigors should also be treated at the physician's discretion but may be controlled by meperidine 25 - 50 mg IV. Experience has shown that rigors generally abate within 30 minutes without pharmaceutical intervention.

If any of these toxicities occur during antibody infusion, the rate of antibody infusion should be decreased as indicated below:

Infusion Rate Adjustment

Fever	Rigors	Mucosal Congestion/ Edema	% Drop in Systolic BP	Infusion Rate Adjustment
Grade 1 (38.0 - 39.0°C)	Grade 1 -2 (Mild to Moderate)	Grade 1 - 2 (Mild to Moderate)	30 - 49<br>by 1/2	Decrease
Grade ≥ 2 (≥ 39°C)	Grade ≥ 3 (Severe)	Grade ≥ 3 (Severe)	≥ 50	Stop Infusion*

* Temporarily discontinue infusion until adverse experiences have reversed (generally 15 to 30 min.) and then resume infusion at 25 - 50% of initial rate.

c. Use of Colony Stimulating Factors (CSF) and Platelet and Red Blood Cell Transfusions:

Colony Stimulating Factors (CSF) should be administered only in accordance with published ASCO guidelines. *(33)* Use of CSF under these conditions will be at the discretion of the treating investigator, but must be recorded on the flow sheets.

Platelet transfusions should be administered only in patients with Grade 3 or 4 thrombocytopenia with obvious bleeding. The use of platelet and red cell transfusions under these conditions will be at the discretion of the treating investigator, but must be recorded on the Flow Sheets.

8.4 For treatment or dose modification related questions, please contact Dr. Press at 206/598-4938 or Dr. Maloney at 206/667-5616.

8.5 Unexpected or fatal toxicities (including suspected reactions) must be reported to the Operations Office, to the Study Coordinator, to the IRB and the NCI. The procedure for reporting adverse reactions is outlined in Section 16.0.

9.0 STUDY CALENDAR **S0016**, "A Phase III Trial of CHOP vs CHOP + Rituximab vs. CHOP + Iodine-131-Labeled Monoclonal Anti-B1 (Tositumomab) Antibody for Treatment of Newly Diagnosed Follicular Non-Hodgkin's Lymphomas"

9.1 S0016 STUDY CALENDAR: ARM 1 - CHOP ONLY

		Cycle 1	Cycle 2	Cycle 3	Cycle 4	Cycle 5	Cycle 6	Ω	Ω	√
**REQUIRED STUDIES**	**PRE STUDY**	**Day 1**	**Day 22**	**Day 43**	**Day 64**	**Day 85**	**Day 106**	**Day 200**	**Day 365**	**Follow Up**
**PHYSICAL**										
History & Physical Exam	X		X	X	X	X	X	X	X	X
Weight & Performance Status	X							X	X	X
Tumor Assessment *f*	X							X	X	X
Toxicity Notation		X	X	X	X	X	X	X	X	X
**LABORATORY**										
CBC, Platelets & Differential	X		X	X	X	X	X	X	X	X
Serum Creatinine	X ß		X	X	X	X	X			X
Bilirubin	X ß		X	X	X	X	X			X
LDH	X ß									X
ß2 Microglobulin	X									
Immunophenotyping (CD20)	X									
Thyroid Stimulating Hormone (TSH)								X	X	X
Urinalysis	X ß									
Uric acid	X ß									
SGOT and/or Alk Phosphatase	X ß									X
Materials for pathology review ¥	X									
Bone marrow asp./biopsy	X							X*	X	X*
Bone marrow submission for bcl2 testing	X								X	
**X-RAYS AND SCANS**										
Chest X-Ray or Chest CT *f*	X*f*							X*f*	X*f*	X*f*
CT scan: abdomen, pelvis	X							X	X	X
EKG	X ß									
MUGA	X Σ									
**TREATMENT (See Section 7.3)** π										
Cyclophosphamide		X	X	X	X	X	X			
Doxorubicin		X	X	X	X	X	X			
Vincristine		X	X	X	X	X	X			
Prednisone (see Section 7.3)		X	X	X	X	X	X			

NOTE: Data submission forms are found in Section 18.0. Forms submission guidelines may be found in Section 14.0.

f The same scanning technique as baseline must be used to allow uniformity of results.

¥ See Section 12.0.

Ω Restaging will occur on Day 200 and on Day 365 after initiation of CHOP. The same assessments that were used at baseline should be used for restaging.

√ Patients removed from protocol treatment for any reason will be evaluated at that time by repeating CBC with platelets, LDH, creatinine, liver enzymes, CT of chest, abdomen and pelvis, and all pre-treatment scans to evaluate disease. Follow-up evaluations will include all restaging tests and will occur every 6 months for the first two years, then annually thereafter.

π Patients with progressive disease at any time will discontinue protocol treatment (see Section 10.2f).

ß These tests are suggested at pre-study for Good Medical Practices (see Section 7.1), but must be obtained as listed above for follow-up toxicity assessment.

Σ Required if clinically indicated (see Section 5.16).

* Repeat if initially abnormal

9.0 STUDY CALENDAR S0016, "A Phase III Trial of CHOP vs CHOP + Rituxamab vs. CHOP + Iodine-131-Labeled Monoclonal Anti-B1 (Tositumomab) Antibody for Treatment of Newly Diagnosed Follicular Non-Hodgkin's Lymphomas"

9.2 S0016 ARM 2 - CHOP + Rituximab

		Cycle 1			Cycle 2	Cycle 3		Cycle 4	Cycle 5		Cycle 6			Ω	Ω	√
**REQUIRED STUDIES**	**PRE STUDY**	**Day 1**	**Day 6**	**Day 8**	**Day 29**	**Day 48**	**Day 50**	**Day 71**	**Day 90**	**Day 92**	**Day 113**	**Day 134**	**Day 141**	**Day 200**	**Day 365**	**Follow Up**
**PHYSICAL**																
History & Physical Exam	X		X	X	X	X	X	X	X	X	X	X	X	X	X	X
Weight & Performance Status	X													X	X	X
Tumor Assessment ƒ	X													X	X	X
Toxicity Notation		X	X	X	X	X	X	X	X	X	X	X	X	X	X	X
**LABORATORY**																
CBC, Platelets & Differential	X				X		X	X		X	X		X	X	X	X
Serum Creatinine	X ß				X		X	X		X	X		X			X
Bilirubin	X ß				X		X	X		X	X		X			X
LDH	X ß															X
ß2 Microglobulin	X															
Immunophenotyping (CD20)	X															
Thyroid Stimulating Hormone														X	X	X
Urinalysis	X ß															
Uric acid	X ß															
SGOT and/or Alk Phosphatase	X ß															X
Materials for pathology review ¥	X															
Bone marrow asp./biopsy	X													X*	X	X*
Bone marrow submission for bcl2 testing	X														X	
**X-RAYS AND SCANS**																
Chest X-Ray or Chest CT ƒ	Xƒ													Xƒ	Xƒ	Xƒ
CT scan: abdomen, pelvis	X													X	X	X
EKG	X ß															
MUGA	X Σ															
**TREATMENT (See Section 7.4) π**																
Rituximab		X	X			X			X			X	X			
Cyclophosphamide				X	X		X	X		X	X					
Doxorubicin				X	X		X	X		X	X					
Vincristine				X	X		X	X		X	X					
Prednisone (see Section 7.3)				X	X		X	X		X	X					

NOTE: Data submission forms are found in Section 18.0. Forms submission guidelines may be found in Section 14.0.

* Repeat if initially abnormal

ƒ The same scanning technique as baseline must be used to allow uniformity of results.

¥ See Section 12.0.

Ω Restaging must occur on Day 200 and Day 365 after initiation of CHOP. The same assessments that were used at baseline should be used for restaging.

√ Patients removed from protocol treatment for any reason will be evaluated at that time by repeating CBC with platelets, LDH, creatinine, liver enzymes, CT of chest, abdomen and pelvis, and all pre-treatment scans to evaluate disease. Follow-up evaluations will include all restaging tests and will occur every 6 months for two years and then annually thereafter.

π Patients with progressive disease at any time will discontinue protocol treatment (see Section 10.2f).

ß These tests are suggested at pre-study for Good Medical Practices (see Section 7.1), but must be obtained as listed above for follow-up toxicity assessment.

Σ Required if clinically indicated (see Section 5.16).

9.0 STUDY CALENDAR S0016, "A Phase III Trial of CHOP vs CHOP + Rituxamab vs. CHOP + Iodine-131-Labeled Monoclonal Anti-B1 (Tositumomab) Antibody for Treatment of Newly Diagnosed Follicular Non-Hodgkin's Lymphomas"

9.3 STUDY CALENDAR: S0016 ARM 3- CHOP + I-131-tositumomab

		Cycle 1	Cycle 2	Cycle 3	Cycle 4	Cycle 5	Cycle 6	£ Re-Eval	Antibody treatment		Ω	Ω	√
**REQUIRED STUDIES**	**PRE STUDY**	**Day 1**	**Day 22**	**Day 43**	**Day 64**	**Day 85**	**Day 106**	**Day 133**	**Day 134**	**Day 141**	**Day 200**	**Day 365**	**Follow Up**
**PHYSICAL**													
History & Physical Exam	X		X	X	X	X	X	X			X	X	X
Weight & Performance Status	X							X			X	X	X
Tumor Assessment ƒ	X							X			X	X	X
Toxicity Notation		X	X	X	X	X	X	X			X	X	X
**LABORATORY**													
CBC, Platelets & Differential	X		X	X	X	X	X	X			X	X	X
Serum Creatinine	X ß		X	X	X	X	X	X					X
Bilirubin	X ß		X	X	X	X	X	X					X
LDH	X ß							X					X
ß2 Microglobulin	X												
Immunophenotyping (CD20)	X												
Thyroid Stimulating Hormone (TSH)								X			X	X	X
Urinalysis	X ß							X ß					
Uric acid	X ß							X ß					
SGOT and/or Alk Phosphatase	X ß							X					X
Materials for pathology review £	X												
Bone marrow asp./biopsy	X							X			X*	X	X*
Coulter approval	X												
Coulter training session						X							
Bone marrow submission for bcl2 testing	X											X	
**X-RAYS AND SCANS**													
Chest X-Ray or Chest CT ƒ	Xƒ										Xƒ	Xƒ	Xƒ
CT scan: abdomen, pelvis	X										X	X	X
EKG	X ß												
MUGA Σ	X												
**TREATMENT (See Section 7.5) π**													
Cyclophosphamide		X	X	X	X	X	X						
Doxorubicin		X	X	X	X	X	X						
Vincristine		X	X	X	X	X	X						
Prednisone (see Section 7.3)		X	X	X	X	X	X						
SSKI, Lugol's, Potassium Iodide §								X §	X §	X §			
Unlabeled anti-B1 antibody									X	X			
I-131 anti-B1 dosimetric infusion									X				
Whole Body Dosimetry ¶									X ¶	X ¶			
I-131 anti-B1 therapeutic infusion ¥										X ¥			

NOTE: Data submission forms are found in Section 18.0. Forms submission guidelines may be found in Section 14.0.

ƒ The same scanning technique as baseline must be used to allow uniformity of results.

£ See Section 12.0.

Ω Restaging must occur on Day 200 and Day 365 after initiation of CHOP. The same assessments that were used at baseline should be used for restaging.

√ Patients removed from protocol treatment for any reason will be evaluated at that time by repeating CBC with platelets, LDH, creatinine, liver enzymes, CT of chest, abdomen and pelvis, and all pre-treatment scans to evaluate disease. Follow-up evaluations will include all restaging tests and will occur every six months for the first two years and then annually thereafter.

£ Re--evaluation will occur 4 weeks after completion of the sixth cycle of CHOP (see Section 7.5b).

π Patients with progressive disease at any time will discontinue protocol treatment (see Section 10.2f).

ß These tests are suggested at pre-study for Good Medical Practices (see Section 7.1), but <u>must</u> be obtained as listed above for follow-up toxicity assessment.

Σ Required if clinically indicated (see Section 5.16).

* Repeat if initially abnormal

§ SSKI, Lugol's or Potassium Iodide will be administered at least 24 hours prior to the dosimetric dose and will continue for 14 days following the last infusion of radiolabeled anti B1-antibody.

¶ Three dosimetric scans are done on Day 1 of antibody treatment (projected to be Day 134 of the protocol treatment), and then either on Day 3, 4, or 5 after antibody treatment and again on either Day 7 or 8 after antibody treatment (see Section 7.5c.3v).

¥ The radioimmunotherapy dose is to be given 7 days after the administration of the dosimetric dose (may be delayed but no longer than 14 days after the dosimetric dose). **A CBC, platelets and differential should be checked weekly after the therapeutic infusion until the patient experiences a nadir and recovers from the nadir (6-8 weeks).**

10.0 CRITERIA FOR EVALUATION AND ENDPOINT DEFINITIONS

10.1 Measurability of Lesions:

a. **Measurable Disease:** Lesions that can be accurately measured in two dimensions by CT, MRI, medical photograph (skin or oral lesion), plain x-ray, or other conventional technique and a greatest transverse diameter of 1 cm or greater; or palpable lesions with both diameters ≥ 2cm. **Note**: CT scans remain the standard for evaluation of nodal disease.

b. **Non-measurable Disease:** All other lesions including unidimensional lesions, lesions too small to be considered measurable, pleural or pericardial effusion, ascites, bone disease, leptomeningeal disease, lymphangitis, pulmonitis, abdominal masses not confirmed or followed by imaging techniques or disease documented by indirect evidence only (e.g., lab values).

10.2 Objective Disease Status:

Objective status is to be recorded at each evaluation. All measurable lesions up to a maximum of 6 lesions (largest) should be identified as target lesions at baseline. If there are more than 6 measurable lesions the remaining will be identified as non-target lesions and included as non-measurable disease. The 6 lesions should be selected according to the following features: they should be from disparate regions of the body as possible and they should include mediastinal and retroperitoneal areas of disease if these sites have measurable lesions.

Measurements must be provided for target lesions, while presence or absence must be noted for non-target measurable and non-measurable disease.

a. **Complete Response (CR):** Complete disappearance of all measurable and non-measurable disease with the exception of nodes for which the following must be true: for patients with at least one measurable lesion and all nodal masses > 1.5cm in greatest transverse diameter (GTD) at baseline must have regressed to ≤ 1.5 cm in GTD and all nodal masses ≥ 1cm and ≤ 1.5cm GTD must have regressed to < 1.0cm in GTD or they must have reduced by 75% in sum of products of greatest diameters (SPD). No new lesions. Spleen and other previously enlarged organs must have regressed in size and must not be palpable. If bone marrow was positive at baseline, it must be negative based on biospy and aspirate at same site. Normalization of markers. (e.g., LDH definitely assignable to NHL). All disease must be assessed using the same technique as baseline.

b. **Complete Response Unconfirmed (CRU):** For patients who do not qualify for CR. Complete disappearance of all measurable and non-measurable disease, regressed, non-palpable spleen and other previously enlarged organs, except with one or more of the following features: 1) all residual nodal masses > 1.5cm in GTD at baseline reduced by 75% in SPD or 2) bone marrow indeterminate. No new lesions. All disease must be assessed using the same technique as baseline.

c. **Partial Response (PR):** Applies to patients with at least one measurable lesion that do not quality for a CR or CRU. A 50% decrease in the SPD for up to six identified dominant lesions identified at baseline. No new lesions and no increase in the size of liver or spleen or other nodes. Spleenic and hepatic nodules must have regressed in size by at least 50% in SPD. All disease must be assessed using the same technique as baseline.

d. **Stable:** Does not qualify for CR, CRU, PR, Relapsed/Progressive Disease. All disease must be assessed using the same technique as baseline.

e. **Relapsed Disease:** If a (CR,CRU) was achieved at a previous assessment, a 50% increase in the SPD of target measurable lesions over the smallest sum observed (over baseline if no decrease during therapy) or 50% increase in the GTD of any node greater than 1cm in shortest axis using the same techniques as baseline. Unequivocal progression of non-measurable disease in the opinion of the treating physician (an explanation must be provided). Appearance of a new lesion/site. Death due to disease without prior documentation of progression.

f. **Progressive Disease:** If a (CR,CRU) was not achieved at a previous assessment, a 50% increase in the SPD of target measurable lesions over the smallest sum observed (over baseline if no decrease during therapy) using the same techniques as baseline. Appearance of a new lesion/site. Unequivocal progression of non-measurable disease in the opinion of the treating physician (an explanation must be provided). Death due to disease without prior documentation of progression.

g. **Assessment inadequate, objective status unknown:** Progression has not been documented and one or more target lesions or other sites of disease have not been assessed or inconsistent methods of assessment were used.

Notes: Bone marrow status is evaluated as follows:

Positive: Unequivocal cytological or architectural evidence of malignancy.

Negative: No aggregates or only a few well-circumscribed lymphoid aggregates.

Indeterminate: Does not qualify for either positive or negative status. Note this typically consists of increased number or size of aggregates without cytological or architectural atypia.

10.3 **Best Response:**

a. CR: One objective status of CR documented before relapse.

b. CRU: One objective status of CRU documented before relapse but not qualifying as a CR.

c. PR: One objective status of PR documented before progression but not qualifying as a CR or CRU.

d. Stable: At least one objective status of stable documented at least 6 weeks after registration, not qualifying as anything else above.

e. Increasing Disease: Objective status of progression within 12 weeks of registration not qualifying as anything else above.

f. Inadequate assessment, response unknown: Progression greater than 12 weeks after registration and no other response category applies.

10.4 **Performance Status**: Patients will be graded according to the Zubrod performance status scale.

POINT	DESCRIPTION
0	Fully active, able to carry on all pre-disease performance without restriction.
1	Restricted in physically strenuous activity but ambulatory and able to carry out work of a light or sedentary nature, e.g., light housework, office work.
2	Ambulatory and capable of self-care but unable to carry out any work activities; up and about more than 50% of waking hours.
3	Capable of limited self-care, confined to bed or chair more than 50% of waking hours.
4	Completely disabled; cannot carry on any self-care; totally confined to bed or chair.

10.5 **Progression-Free Survival**: From date of registration to date of first observation of progressive disease (as defined in Section 10.2f) or death due to any cause.

10.6 **Time to Death**: From date of registration to date of death due to any cause.

11.0 STATISTICAL CONSIDERATIONS

11.1 Based on S9800 and S9911 the anticipated accrual rate is 11 - 12 patients per month.

11.2 The primary objective of this trial is to determine if either of the two new experimental regimens are superior to CHOP in terms of progression-free survival. If both are found to be superior, then the two experimental arms will be compared. Assume the 2 year progression-free survival rate is 65% in the control arm. One-sided (level .025) logrank tests of 1) CHOP vs CHOP/tositumomab and 2) CHOP vs CHOP/rituximab and a two-sided test between the experimental arms will be conducted only if both of the two experimental regimens is shown to be superior to the CHOP arm. Approximately 775 eligible patients randomized over 5.5 years with 2 additional years of follow-up will be required to have power of .90 to detect a hazard ratio of conventional therapy to either experimental therapy arm of 1.50 based on a one-sided test and assuming exponential progression-free survival distributions.

A sample size of approximately 228 per arm is sufficient to estimate the response rate or any given toxicity to within ± 7% for each regimen.

It is estimated that 80% of patients will be bcl2 positive at baseline. At least 75% of these patients are expected to be progression free at 1 year. Therefore, it is expected that 155 patients per arm are expected to be assessable for bcl2 disappearance at 1 year given complete ascertainment of samples. One hundred and 155 patients are sufficient to estimate the rate of bcl2 disappearance to within +/- .08.

11.3 This study will be monitored throughout accrual and follow-up periods by the Southwest Oncology Group Data and Safety Monitoring Committee (DSMC). In addition to monitoring by the DSMC, formal interim analyses will be done after 50% of eligible patients have been randomized and again after 75% of the eligible patients have been randomized. Evidence to suggest termination of an inferior arm of the study at the time of analysis if the null hypothesis of no difference, or the alternative hypothesis of a hazard ratio of conventional therapy to experimental therapy of 1.50 with respect to progression-free survival is rejected at the .0025 level. There will be no comparison of experimental arms unless both experimental arms are found to be superior to the standard. The actual decision to terminate the study early will be made by the DSMC, and will take into consideration overall survival, progression-free survival and other factors such as toxicities and complications. If accrual at 2 years is less than 50% of the expected

+ final d

accrual at 2 years, a recommendation will be made that the DSMC consider closing the study for poor accrual. If the study is not terminated early, reporting of results at the time closure will be considered if the null hypothesis of no difference, or the alternative of a hazard ratio of conventional therapy to experimental therapy of 1.5 for progression-free survival were rejected at the .0025 level. If the study is not terminated early or the hypothesis tests at the time of closure are not rejected, the final analysis on progression-free survival will be completed after approximately 2 years of follow-up after closing randomization.

12.0 DISCIPLINE REVIEW

12.1 Pathology Review

All patients registered on this study will undergo pathology review. The purpose of this review is to verify the histologic diagnosis of follicular non-Hodgkin's lymphoma (Grade I, II, or III) and that patients are CD20 positive. Any excess diagnostic tissue will be retained by the Southwest Oncology Group (unless the patient denies consent).

12.2 Pathology materials are to be submitted within 30 days of registration to:

Dr. Thomas Grogan
Department of Pathology
Arizona Cancer Center
1501 N. Campbell Avenue
Tucson, AZ 85724-0001
Phone: 520/626-2212

An additional copy of the Protocol Specific Pathology Submission Form (Form #) must be submitted to the Statistical Center each time submissions are made to Dr. Grogan.

The materials must be identified with a "SWOG Pathology Materials" label on the outside of each package. If this label is missing, the materials will not be reviewed, rendering the patient ineligible. These labels will be provided by the Statistical Center. To obtain additional labels, please call 206/667-4623 and ask for the Pathology Coordinator.

12.3 The following materials are to be submitted for review:

a. Representative H&E stained slides from the original diagnostic biopsy. (Note: Needle aspirates are not adequate for this submission. Consult with Dr. Grogan if adequacy of specimen is in question.)

b. One representative paraffin block which will be conserved (no more than eight additional slides will be cut).

c. One copy of pathology report.

d. One copy of Southwest Oncology Group Study Specific Pathology Submission Form (Form #). (A copy of the Study Specific Pathology Submission Form (Form #) must also be sent to the Statistical Center.)

12.4 Failure to submit a registered patient's pathology materials for pathology review will make the patient ineligible.

13.0 REGISTRATION GUIDELINES

13.1 Patients from Member and Affiliate institutions must be registered with the Southwest Oncology Group Statistical Center by telephoning 206/667-4623, 6:30 a.m. to 5:00 p.m. Pacific time, Monday through Friday, excluding holidays. Patients must be registered prior to initiation of treatment (no more than one working day prior to planned start of treatment).

Patients from CCOP Institutions must be registered with the Southwest Oncology Group CCOP Office by telephoning 206/652-CCOP (206/652-2267), 7:00 a.m. to 4:00 p.m., Pacific Time, Monday through Friday, excluding holidays. Patients must be registered prior to initiation of treatment (no more than one working day prior to the planned start of treatment).

13.2 The caller must have completed the appropriate Southwest Oncology Group Registration Form. The completed form must be referred to during the registration but should not be submitted as part of patient data.

13.3 The caller must also be prepared to provide the treating institution's name and ID number to the Statistical Center in order to ensure that the current (within 365 days) date of institutional review board approval for this study has been entered into the data base. Patients will not be registered if the IRB approval date has not been provided or is > 365 days prior to the date of registration.

13.4 Exceptions to the current registration policies will not be permitted. Therefore, late registrations (after initiation of treatment), exceptions to eligibility requirements, participation by an institution/member not identified as eligible **AND/OR** cancellations will not be allowed.

14.0 DATA SUBMISSION SCHEDULE

14.1 Data must be submitted according to the protocol requirements for **ALL** patients registered, whether or not assigned treatment is administered, including patients deemed to be ineligible. Patients for whom documentation is inadequate to determine eligibility will be deemed ineligible

14.2 Master forms are included in Section 18.0 and (with the exception of the sample consent form and the Registration Form) must be photocopied for data submission to the Statistical Center.

14.3 Group Members and Affiliates

Group members must submit one copy of all data forms directly to the Statistical Center in Seattle. Affiliates must submit (number of copies to be determined by the Group member) copies of all forms to their Group member institution for forwarding to the Statistical Center.

CCOP Institutions

CCOP Institutions must submit one copy of all data forms to the SWOG CCOP Office in Seattle at the following address:

Cancer Research and Biostatistics (CRAB)
ATTN: SWOG CCOP Office
1100 Olive Way, Suite 1150
Seattle, Washington 98101-1892

OR CCOP members may submit data via facsimile to 206/652-4612. Faxed data must be accompanied by the Data Submission Facsimile Cover Sheet.

14.4 WITHIN 14 DAYS OF REGISTRATION:

Submit a copy of the following:

a. Non-Hodgkin's Lymphoma (Follicular) Prestudy Form (Form #)

b. Lymphoma Baseline Tumor Assessment Form (Form #)

c. Pathology Report confirming histology and CD20 antigen expression

d. Initial Study Specific Flow Sheet. The Flow Sheet must include history and physical, prestudy tests/exams results (Section 5.0 for First Registration, may also be submitted in lieu of comprehensive documentation of those items), the first five days or protocol treatment, dose calculations and toxicity notations.

Submit bone marrow for bcl2 assessment along with a Specimen Submission Form (Form #1951) to Dr. Rita Braziel per Section 15.2. A copy of the Specimen Submission Form (Form #1951) should be sent to the Statistical Center at the time the sample is submitted to Dr. Braziel.

14.5 WITHIN 30 DAYS OF REGISTRATION:

Submit histopathologic materials along with a copy of the pathology reports and a copy of the Study Specific Pathology Submission Form (Form #) to Dr. Grogan (see Section 12.0). (A copy of the Study Specific Pathology Submission Form (Form #) must also be sent to the Statistical Center.)

14.6 EVERY THREE MONTHS WHILE ON PROTOCOL TREATMENT:

Submit a copy of the Study Specific Flow Sheets.

14.7 EVERY SIX MONTHS AFTER OFF TREATMENT FOR TWO YEARS AND THEN ANNUALLY THEREAFTER:

Submit a copy of the Southwest Oncology Group Follow-up Form (Form #1512).

14.8 AT ONE YEAR FOLLOW-UP:

Submit marrow for bcl2 assessment along with a Specimen Submission Form (Form #1951) to Dr. Rita Braziel per Section 15.2. A copy of the Specimen Submission Form (Form #1951) should also be sent to the Statistical Center at the time the sample is submitted to Dr. Braziel.

14.9 WITHIN 14 DAYS OF PROGRESSION OR RELAPSE:

If the patient progresses while on treatment, submit a copy of the Study Specific Flow Sheet documenting date, site and method for determining progression or relapse. Otherwise, if the patient progresses after off-treatment, submit the Southwest Oncology Group Follow-Up Form (Form #1512).

14.10 WITHIN 14 DAYS AFTER DISCONTINUATION OF ALL PROTOCOL TREATMENT:

Submit a copy of the Off Treatment Notice (Form #22204) and a final Flow Sheet documenting all toxicity and disease assessments required at the end of treatment.

14.11 WITHIN FOUR WEEKS OF KNOWLEDGE OF DEATH:

Submit either a final Study Specific Flow Sheet documenting death information (if death occurs while on treatment) or a final Southwest Oncology Group Follow-Up Form (Form #1512) (If death occurs after off treatment). Also, submit a copy of the Notice of Death (Form #1821).

15.0 SPECIAL INSTRUCTIONS

15.1 Instructions for obtaining institutional approval and training prior to registration and I-131 treatment of patients on **S0016**:

Prior to the registration of any patient to **S0016**, the registering institution must be approved by Coulter Pharmaceutical for the delivery of I-131 therapy. The Nuclear Medicine Physician Questionnaire (Appendix 19.5) must be completed and faxed to Coulter at the number listed on the bottom of the form. If approved, Coulter will send the institution an approval notice and number and will also notify the Southwest Oncology Group Statistical Center that the institution is approved to register patients to this study.

Once a patient is randomized to Arm 3, the institution must contact Coulter immediately by calling Teresa White at Coulter Pharmaceutical (650/553-1886) to arrange for an on-site training session for Iodine I-131 therapy. Training may occur at any time after the 5th cycle of CHOP, but it must be completed before the patient can receive the Iodine-131 Anti-B1 Antibody therapy.

NOTE: Coulter approval and training be must performed prior to registration and I-131 treatment respectively only for the first patient registered to and treated on this study at any one institution. Institutions that have previously completed on-site training for the Southwest Oncology Group study, S9911, need not repeat the training.

15.2 Instructions for **S0016** bone marrow specimen submission for t(14:18)/bcl2 testing:

a. Timing	*Type of specimen	Quantity	Destination
1) Study entry, and<br>2) 1 year follow-up*	Marrow for t(14:18)/bcl2	2 - 3 cc (heparinized)	Rita Braziel, M.D.<br>Flow Cytometry Lab<br>Department of Pathology, L471<br>Oregon Health Sciences University<br>3181 SW Sam Jackson Park Road<br>Portland, OR 97201<br>Phone: 503/494-2302<br>Fax: 503/494-0731

* Samples should be submitted for all patients that have not progressed.

1. Samples must be sent **overnight**, the day they are obtained, to Dr. Braziel at the address listed above. Samples should be shipped at room temperature.

2. **A Southwest Oncology Group Specimen Submission Form must be completed and sent with every specimen. Since multiple specimens will be collected from each patient, the status of the patient is critical (i.e., prestudy, after completion of CHOP, after completion of Iodine-131 anti-B1 antibody) and must be clearly indicated on each form.**

b. The Federal guidelines for shipment are as follows:

1. The specimen must be wrapped in an absorbable material;

2. The specimen must then be placed in an AIRTIGHT container (like a resealable bag);

3. Pack the resealable bag and specimen in a styrofoam shipping container;

4. Pack the styrofoam shipping container in a cardboard box.

5. The cardboard box must be marked as "BIOHAZARD".

c. Weekend Shipping (Arrival on Saturday)

Samples will be accepted on Saturdays if the bone marrow evaluation cannot be postponed until Monday for clinical reasons; however, the OHSU Flow Cytometry Laboratory MUST be contacted at 503/494-2302 at least two days before shipping the sample so that special mailing instructions for specimens can be obtained. Indicate Saturday delivery on the overnight mailing label.

15.3 Patients may also have tissue and serum submitted as part of **SWOG-8819** and **SWOG-8947** respectively.

16.0 ETHICAL AND REGULATORY CONSIDERATIONS

The following must be observed to comply with Food and Drug Administration regulations for the conduct and monitoring of clinical investigations; they also represent sound research practice:

Informed Consent

The principles of informed consent are described by Federal Regulatory Guidelines (Federal Register Vol. 46, No. 17, January 27, 1981, part 50) and the Office for Protection from Research Risks Reports: Protection of Human Subjects (Code of Federal Regulations 45 CFR 46). They must be followed to comply with FDA regulations for the conduct and monitoring of clinical investigations.

Institutional Review

This study must be approved by an appropriate institutional review committee as defined by Federal Regulatory Guidelines (Ref. Federal Register Vol. 46, No. 17, January 27, 1981, part 56) and the Office for Protection from Research Risks Reports: Protection of Human Subjects (Code of Federal Regulations 45 CFR 46).

Drug Accountability

For each drug supplied for a study, an accountability ledger containing current and accurate inventory records covering receipt, dispensing, and the return of study drug supplies must be maintained. Drug supplies must be kept in a secure, limited access storage area under the recommended storage conditions. During the course of the study, the following information must be noted on the accountability ledger; the identification code of the subject to whom drug is dispensed, the date(s) and quantity of drug dispensed to the subject, and the date(s) and quantity of drug returned by the subject; subjects should return empty containers to the investigator, with the return noted on the ledger. These Accountability Forms must be readily available for inspection and are open to FDA inspection at any time.

Adverse Experiences

Any adverse experience, if deemed drug related, must be reported to the Operations Office Adverse Drug Reaction (ADR) representative (210/677-8808), who will obtain information on the ADR. Depending on the nature of the reaction and whether it was caused by an investigational or commercial agent, the ADR representative will advise whether the report to the NCI should be phoned in, written in, or both. See guidelines below. On Phase II and III studies, all deaths considered drug-related must be reported immediately to the ADR representative. On double-blinded studies, if the investigator must know what treatment the subject received to make

therapeutic decisions, the code for that particular subject can be broken by telephoning the Statistical Center.

All adverse experiences must also be reported to the Institutional Review Board within 10 days and documentation of this report sent to the Operations Office.

All adverse experiences must also be recorded in the appropriate section of the case report form. The report should include, whenever possible, the investigator's written medical judgment as to relationship of the adverse experience to study medication(s) (i.e., "probable", "possible" or "unrelated").

Monitoring

This study will be monitored by the Clinical Data Update System (CDUS) version 2.X. Cumulative CDUS data will be submitted quarterly to CTEP by electronic means. Reports are due January 31, April 30, July 31 and October 31.

GUIDELINES FOR REPORTING OF
ADVERSE EVENTS (AE) / ADVERSE DRUG REACTIONS (ADR)
OCCURRING WITH **COMMERCIAL** AGENTS

1. **WITHIN 24 HOURS OF THE EVENT CALL THE OPERATIONS OFFICE AT 210-677-8808**

2. WITHIN 10 DAYS, SEND TO THE OPERATIONS OFFICE

    a) A COPY OF THE FDA FORM 3500 (or the NCI/CTEP Secondary AML/MDS Report Form for reporting cases of secondary AML or MDS)
    b) COPIES OF PRESTUDY FORMS, AND FLOW SHEETS FROM PRESTUDY THROUGH THE EVENT
    c) IRB NOTIFICATION DOCUMENTATION
    d) OTHER DATA AS REQUESTED DURING TELEPHONIC REPORT.

3. IN ADDITION, FOLLOW THE GUIDELINES BELOW

The following guidelines for reporting an AE/ADR apply to any research protocol which uses commercial anticancer agents. The following AE/ADR experienced by patients accrued to these protocols and attributed to the commercial agent(s) should be reported:

(a) Any AE/ADR which is life threatening (Grade 4) or fatal (Grade 5) and unknown.[1,2,3] Any occurrence of secondary AML or MDS must also be reported[4].

(b) Any increased incidence of a known AE/ADR reported in the protocol.

(c) Any AE/ADR which is fatal (Grade 5), even though known.[3]

The AE report, documented on Form FDA-3500 or NCI/CTEP Secondary AML/MDS Report Form, should be mailed to the address below within 10 working days:

Investigational Drug Branch
P. O. Box 30012
Bethesda, MD 20824-0012

Send a copy of the Form FDA-3500 or NCI/CTEP Secondary AML/MDS Report Form, plus prestudy form, flowsheets, and a copy of IRB notification to the Operations Office within 10 working days:

Southwest Oncology Group Operations Office
ATTN: ADR Program
14980 Omicron Drive
San Antonio, TX 78245-3217

[1] For grading reactions, see NCI Common Toxicity Criteria, Section 19.0.

[2] All known toxicities can be found in either the Drug Information, Background or Informed Consent Form sections of the protocol.

[3] **A report shall be submitted if there is only a reasonable suspicion of drug effect.** Reactions judged definitely not treatment related should not be reported, except that all deaths while on treatment or within 30 days after treatment must be reported. Any death more than 30 days after treatment which is felt to be treatment related must also be reported.

[4] For reporting cases of secondary AML or MDS, use the NCI/CTEP Secondary AML/MDS Report Form in lieu of Form FDA-3500. The Operations Office will forward this form to the Statistical Center within one working day of receipt.

GUIDELINES FOR REPORTING OF
ADVERSE EVENTS (AE)/ADVERSE DRUG REACTIONS (ADR)
OCCURRING WITH **INVESTIGATIONAL** AGENTS
ON PHASE II AND III STUDIES

WITHIN 24 HOURS OF THE EVENT

CALL THE OPERATIONS OFFICE AT 210/677-8808

WITHIN 10 DAYS, SEND TO THE OPERATIONS OFFICE

1) **A COPY OF THE ADR REPORTING FORM OR THE "NCI/CTEP SECONDARY AML/MDS REPORT FORM" (for reporting cases of secondary AML or MDS)**
2) **IRB NOTIFICATION DOCUMENTATION**
3) **COPIES OF ALL DATA RECORDS**

IN ADDITION, FOLLOW THE GUIDELINES BELOW

UNKNOWN EVENT[4,5,6]

Grade 2-3[2]	Written report to IDB within 10 working days.[3]
Grade 4-5	Report by phone to IDB within 24 hours.[1]<br>Written report to follow within 10 working days.

KNOWN EVENT[4,5,6]

Grade 1-3	Not to be reported as an AE/ADR. These toxicities should be submitted on the flow sheets.
Grade 4-5	Written report to IDB within 10 working days.<br>Grade 4 myelosuppression not to be reported, but should be submitted on the flow sheets.<br>For patients with leukemia that develop Grade 5 aplasia, a written report is required within 10 working days.

1. IDB telephone number available 24 hours daily: 301/230-2330 (Recorder after hours).

2. For grading reaction, see NCI Common Toxicity Criteria, Section 19.0.

3. The event should be documented on the SWOG AE/ADR form and sent to:

   ATTN: ADR Program
   Southwest Oncology Group
   14980 Omicron Drive
   San Antonio, TX 78245-3217

   and

   Investigational Drug Branch
   P.O. Box 30012
   Bethesda, MD 20824

4. A list of all known toxicities can be found in either the Drug Information, Background or Informed Consent Form of the protocol.

5. Reactions judged definitely not to be treatment related should not be reported except any death while on treatment or within 30 days of treatment must be reported. Any death more than 30 days after treatment but which is felt to be treatment related, must also be reported. **However, a report shall be submitted if there is only a reasonable suspicion of drug effect.**

6. For reporting cases of secondary AML or MDS, please use the "NCI/CTEP Secondary AML/MDS Report Form" in lieu of the AE/ADR Reporting Form. Copies of the "NCI/CTEP Secondary Leukemia Report Form" will be forwarded from the Operations Office to the Statistical Center within one working day.

17.0 BIBLIOGRAPHY

1. Horning SJ. Treatment approaches to the low-grade lymphomas. Blood 83:881-4, 1994.

2. Morrison VA, Peterson BA. Combination chemotherapy in the treatment of follicular low-grade lymphoma. Leuk Lymphoma 10:29-33, 1993.

3. Jones SE, Grozea PN, Miller TP, Van Slyck EJ, Balcerzak SP, Costanzi JJ, Morrison FS, Eyre HJ, Fabian CJ, Dabich L, et al. Chemotherapy with cyclophosphamide, doxorubicin, vincristine, and prednisone alone or with levamisole or with levamisole plus BCG for malignant lymphoma: a Southwest Oncology Group Study. J Clin Oncol 3:1318-24, 1985.

4. McKelvey EM, Gottlieb JA, Wilson HE, Haut A, Talley RW, Stephens R, Lane M, Gamble JF, Jones SE, Grozea PN, Gutterman J, Coltman C, Moon TE. Hydroxyldaunomycin (Adriamycin) combination chemotherapy in malignant lymphoma. Cancer 38:1484-93, 1976.

5. Jones SE, Grozea PN, Metz EN, Haut A, Stephens RL, Morrison FS, Talley R, Butler JJ, Byrne GE, Jr., Hartsock R, Dixon D, Salmon SE. Improved complete remission rates and survival for patients with large cell lymphoma treated with chemoimmunotherapy. A Southwest Oncology Group Study. Cancer 51:1083-90, 1983.

6. Dana BW, Dahlberg S, Nathwani BN, Chase E, Coltman C, Miller TP, Fisher RI. Long-term follow-up of patients with low-grade malignant lymphomas treated with doxorubicin-based chemotherapy or chemoimmunotherapy. J Clin Oncol 11:644-51, 1993.

7. Kantarjian HM, McLaughlin P, Fuller LM, Dixon DO, Osborne BM, Cabanillas F. Follicular large cell lymphoma: analysis and prognostic factors in 62 patients. J Clin Oncol 2:811-9, 1984.

8. Horning SJ, Weiss LM, Nevitt JB, Warnke RA. Clinical and pathologic features of follicular large cell (nodular histiocytic) lymphoma. Cancer 59:1470-4, 1987.

9. Anderson JR, Vose JM, Bierman PJ, Weisenberger DD, Sanger WG, Pierson J, Bast M, Armitage JO. Clinical features and prognosis of follicular large-cell lymphoma: a report from the Nebraska Lymphoma Study Group. J Clin Oncol 11:218-24, 1993.

10. Bartlett NL, Rizeq M, Dorfman RF, Halpern J, Horning SJ. Follicular large-cell lymphoma: intermediate or low grade? J Clin Oncol 12:1349-57, 1994.

11. Velasquez W, Lew D, Miller T, Fisher R. **SWOG-9501**: A phase II trial of a combination of fludarabine and mitoxantrone (FN) in untreated advanced low grade lymphoma: an effective well tolerated therapy. Proc. Am. Soc. Clin. Oncol. 18: 9a (abstract 27), 1999.

12. Gribben JG, Neuberg D, Freedman AS, Gimmi CD, Pesek KW, Barber M, Saporito L, Woo SD, Coral F, Spector N, et al. Detection by polymerase chain reaction of residual cells with the bcl-2 translocation is associated with increased risk of relapse after autologous bone marrow transplantation for B-cell lymphoma. Blood 81:3449-57, 1993.

13. Maloney DG, Liles TM, Czerwinski DK, Waldichuk C, Rosenberg J, Grillo-Lopez A, Levy R. Phase I clinical trial using escalating single-dose infusion of chimeric anti-CD20 monoclonal antibody (IDEC-C2B8) in patients with recurrent B-cell lymphoma. Blood 84:2457-66, 1994.

14. Maloney DG, Grillo-Lopez AJ, White CA, Bodkin D, Schilder RJ, Neidhart JA, Janakiraman N, Foon KA, Liles TM, Dallaire BK, Wey K, Royston I, Davis T, Levy R. Idec-C2B8 (Rituximab) anti-CD20 monoclonal antibody therapy in patients with relapsed low grade non-Hodgkin's lymphoma. Blood: 90: 2188, 1997. PMID: 9310469; UI: 97454394.

15. McLaughlin P, Cabanillas F, Grillo-Lopez AJ, Link BK, Levy R, Czuczman M, Heyman MR, Williams M, Jain V, Bence-Bruckler I, Ho AD, Lister J, Rosenberg J, Dallaire BK, Shen D. IDEC-C2B8 anti-CD20 antibody: final report on a phase III pivotal trial in patients with relapsed low-grade or follicular lymphoma. Blood 88 (supp 1):349a, 1996.

16. Solal-Celigny Ph, Salles G, Brousse N, et al. Rituximab as first-line treatment of patients with follicular lymphoma and a low-burden tumor: clinical and molecular evaluation. Blood 94: 631a (abstract 2802) (Suppl 1)

17. Coiffier B, Haioun C, Ketterer N, et al: Rituximab (anti-CD20 monoclonal antibody) for the treatment of patients with relapsing or refractory aggressive lymphoma: a multicenter Phase II study. Blood 92: 1927, 1998

18. Foran JM, Rohatiner AZ, Cunningham D, et al. European phase II study of rituximab (chimeric anti-CD20 monoclonal antibody) for patients with newly diagnosed mantle-cell lymphoma and previously treated mantle-cell lymphoma, immunocytoma, and small B-cell lymphocytic lymphoma. J Clin Oncol 18:317-24, 2000

19. Byrd JC, Waselenko JK, Maneatis TJ, et al. Rituximab therapy in hematologic malignancy patients with circulating blood tumor cells: Association with increased infusion-related side effects and rapid blood tumor clearance. J Clin Oncol 17:791, 1999

20. Czuczman MS, Grillo-López AJ, White CA, Saleh M, Gordon L, LoBuglio AF, Jonas C, Klippenstein D, Dallaire B, Varns C. Treatment of patients with low-grade B-cell lymphoma with the combination of chimeric anti-CD20 monoclonal antibody and CHOP chemotherapy. J Clin Oncol. 17: 268-276, 1999. PMID: 10458242; UI: 99385424.

21. Kaminski MS, Zasadny KR, Francis IR et al. Radioimmunotherapy of B-cell lymphoma with I-131 anti-B1 (anti-CD20) antibody. NEJM 329: 459, 1993

22. Kaminski M, Zasadny K, Francis I, et al. Iodine-131-anti-B1 radioimmunotherapy for B-cell Lymphoma. J Clin Oncol 14:1974, 1996.

23. Kaminski M, Gribbin T, Estes J et al. I-131-Anti-B1 Antibody for previously untreated follicular lymphoma: clinical and molecular remissions. Proc. Am. Soc. Clin. Oncol. 17: 2a, 1998.

24. Press OW, Eary J, Appelbaum FR, Martin PJ, Badger CC, Nelp WB, Glenn S, Butchko G, Fisher D, Porter B, Matthews D, Fisher L, Bernstein ID. Radiolabeled antibody therapy of B cell lymphomas with autologous bone marrow support. NEJM 324: 1219-1224, 1993.

25. Press O, Eary J, Appelbaum F, et al. Phase II trial of 131I-B1 (anti-CD20) antibody therapy with autologous stem cell transplantation for relapsed B cell lymphomas. Lancet 346: 336, 1995.

26. Knox S, Goris M. Trisler K, et al. Yttrium-90-labeled anti-CD20 monoclonal antibody therapy of recurrent B-cell lymphoma. Clin Cancer Res 2:457, 1996.

27. Witzig TE, White CA, Wiseman GA, et al. Phase I/II trial of IDEC-Y2B8 radioimmunotherapy for treatment of relapsed or refractory CD20-positive B-cell non-Hodgkin's lymphoma. J Clin Oncol 1999 (in press).

28. Witzig TE, White CA, Gordon LI et al. Prospective randomized controlled study of Zevalin (Idec-Y2B8) radioimmunotherapy compared to rituximab immunotherapy for B cell NHL: Report of interim results. Blood 94: 631a (abstract 2805) (Suppl 1).

29. O'Donoghue JA. Optimal therapeutic strategies for radioimmunotherapy. Recent Results in Cancer Research 36(10):1910-2. PMID: 7562063; UI: 96007646, 1996.

30. Solal-Celigny P, et al. Recombinant interferon alfa-2b combined with a regimen containing doxorubicin in patients with advanced follicular lymphoma. Groupe d'Etude des Lymphomes de l'Adulte. NEJM 329(22):1608-14. PMID: 8232429; UI: 94049998, 1993.

31. Rohatiner AZS, Gregory W, Peterson B, Smalley R, Solal-Celigny P, Hagenbeek A, Bijnens L, Unterhalt M, Chisesi T, Aviles A, Lister TA. A meta-analysis of randomises trials evaluating the role of interferon as treatment for follicular lymphomas. Proc Am. Soc. Clin. Oncol. 17: 4a (abstract 11), 1998.

32. Demidem A, Lam T, Alas S, Hariharan K, Hanna H, Bonavida B. Chimeric anti-CD20 antibody (IDEC-C2B8) monoclonal antibody sensitizes a B cell lymphoma cell line to cell killing by cytotoxic drugs. Cancer Biotherapy & Radiopharmaceuticals 12:177, 1997.

33. Smith TJ, Ozer H, Miller L, et al. Update of recommendations for the use of hematopoietic colony-stimulating factors: evidence-based clinical practice guidelines. J. Clin. Oncol. 14: 1957, 1996.

34. Dana BW, Unger J, Fisher RI. A randomized study of Alpha-Interferon consolidation in patients with low-grade lymphoma who have responded to Pro-MACE-MOPP (Day 1 - 8) (**SWOG-8809**). ASCO 17:3 (#10), 1998.

18.0 MASTER FORMS SET

18.1 Attached are copies of all data forms which must be completed for this study. The model informed consent form is also included, and must be reviewed and approved by the institutional review board prior to registration and treatment of patients on this study.

18.2 Forms to be used for patients treated on this study include:

a. **S0016** Registration Form (Form #_____)

b. Non-Hodgkin's Lymphoma (Follicular) Prestudy Form (Form #_____)

c. Lymphoma Baseline Tumor Assessment Form (Form # ______)

d. Study Specific Flow Sheet

e. Study Specific Pathology Submission Form (Form #_____)

f. Southwest Oncology Group Specimen Submission Form (Form #1951)

g. Off Treatment Notice (Form #22204)

h. Notice of Death (Form #1821)

i. Southwest Oncology Group Follow-Up Form (Form #1512)

S0016, "A Phase III Trial Of CHOP Vs CHOP + Rituximab Vs CHOP + Iodine-131-Labeled Monoclonal Anti-B1 (Tositumomab) Antibody For Treatment Of Newly Diagnosed Follicular Non-Hodgkin's Lymphomas"

This is a clinical trial (a type of research study). Clinical trials include only patients who choose to take part. Please take your time to make your decision. Discuss it with your family and friends.

You are being asked to take part in this study because you have a kind of cancer called "follicular" lymphoma. This is a cancer of the lymph nodes which is not curable with current treatments.

WHY IS THIS STUDY BEING DONE?

The purpose of this study is to find out if a combination of drugs (called CHOP) either alone or followed by an antibody (rituximab) or a radioactive antibody (Iodine-131 anti-B1 antibody - also called tositumomab), is able to stop the growth of your cancer. We also want to find out if the side effects of this treatment are tolerable. The rituximab antibody is a protein which is partly mouse in origin and partly of human origin. The Iodine-131 anti-B1 antibody is a mouse antibody attached to radioactive Iodine (radiolabeled). These antibodies attach to white blood cells (B cells) in your blood and tumor. The combination of CHOP chemotherapy and these antibodies have been used to treat patients with lymphoma and have been shown to be active against lymphoma. CHOP chemotherapy has been used for 30 years to treat patients with lymphomas and results in tumor shrinkage in 60 - 90% of patients. We are conducting this trial to see if patients treated with CHOP chemotherapy followed by the rituximab antibody or by tositumomab may have longer lasting tumor shrinkage, and hopefully cures, than patients treated with CHOP chemotherapy alone. We also want to find out whether either of the two antibody treatments increase the side effects of CHOP treatment.

Another goal of this study is to find out whether this treatment causes changes in the cells of your bone marrow. Because of this goal, you will have samples of your bone marrow sent to a special laboratory for testing.

Researchers would also like to do laboratory testing on tissue samples in order to find out as much as possible about non-Hodgkin's lymphoma and how this treatment might affect the disease. Some of your tissue must be submitted for this study for testing in order to confirm your type of non-Hodgkin's lymphoma. If any tissue is left over, you may choose to allow this tissue to be kept for research purposes. Also, you may choose to allow additional tissue, both preserved and fresh-frozen, to be kept and used for research purposes.

As part of the ongoing scientific and biotechnological activities of the Southwest Oncology Group and its agents, blood (or tissue) samples may be preserved and used for research and development purposes. As a result of these biotechnological activities, an economic benefit may be derived directly or indirectly by the Southwest Oncology Group, individual researchers, and others engaged in these activities. By signing this consent form, you authorize the preservation and use of these blood samples and any tissue specimens taken from you.

HOW MANY PEOPLE WILL TAKE PART IN THE STUDY?

About 775 people will take part in this study.

WHAT IS INVOLVED IN THE STUDY?

Registration/Randomization

Arm 1: CHOP chemotherapy only

Arm 2: CHOP chemotherapy with concurrent rituximab antibody

Arm 3: CHOP chemotherapy followed by I-131 anti-B1 antibody (tositumomab)

If you choose to participate in this study you will be "randomized" into one of the study groups described below. Randomization means that you are put into a group by chance. It is like flipping a coin. A computer decides which group you will be in. Neither you nor the researcher will choose what group you will be in. You will have an equal chance of being placed in any group.

If you are randomized to the first group (Arm 1) you will receive CHOP chemotherapy only. If you are randomized to the second group (Arm 2) you will receive CHOP chemotherapy plus rituximab antibody concurrently. If you are randomized to the third group (Arm 3) you will receive CHOP chemotherapy followed by the I-131 anti-B1 antibody, tositumomab

Arm 1: If you are assigned by the computer to Arm 1, you will receive the drugs cyclophosphamide, doxorubicin, and vincristine through a needle in your vein on the first day of each treatment "cycle". Each "cycle" lasts 21 days. You will also take prednisone pills for the first five days of the cycle. This combination of drugs is known as CHOP. ***It will take about 15 - 45 minutes to receive the cyclophosphamide, 5 - 20 minutes for the doxorobucin, and 5 - 15 minutes for the vincristine. You may also receive a drug called G-CSF or GM-CSF during some cycles of your CHOP therapy if your doctor determines it is needed to help keep your blood counts from becoming too low. If you receive G-CSF or GM-CSF, it will be injected under your skin once a day as long as your blood counts are low.*** This treatment will be repeated every 21 days for 6 cycles as long as your disease is getting better. If your disease or symptoms get worse, you will stop treatment on this study and be offered other treatment by your doctor.

Arm 2: If you are assigned by the computer to Arm 2, you will receive six cycles of CHOP chemotherapy every 21 days exactly as described above in Arm 1. The CHOP chemotherapy cycles will begin on Days 8, 29, 50, 71, 92, and 113 of your treatment. You will also receive the rituximab antibody through a needle in your vein along with the CHOP chemotherapy on Days 1, 6, 48, 90, 134, and 141. ***It will take about 4 - 6 hours for the rituximab infusion. You may also receive the drugs acetaminophen and diphenhydramine orally before the rituximab infusions to help control pain and prevent possible allergic reaction.*** If your disease or symptoms get worse, you will stop treatment on this study and be offered other treatment by your doctor.

Arm 3: If you are assigned by the computer to Arm 3, you will receive the CHOP chemotherapy treatment every twenty-one days for six cycles exactly as described for Arm 1. In addition, about four to eight weeks after you finish the sixth cycle of this chemotherapy, you will receive a test dose of Iodine-131 tositumomab *("dosimetric" dose)*. ***It will take about 1 1/2 hours to receive your test dose. You will first receive an infusion of "cold" nonradioactive antibody over 60 minutes, followed by an infusion of radioactive "hot" antibody over 20 - 30 minutes.*** Following this test dose, your whole body will be scanned three times over the period of a week as an outpatient to determine the correct treatment dose of the antibody. One to two weeks after the test dose, you will receive a treatment dose *("therapeutic" dose)* of the Iodine-131 tositumomab antibody ***over about 1/2 to 1 hour. You will again receive the "cold" and then the "hot" antibody infusions as described above. Each time you receive the antibody you will receive the drugs acetaminophen and diphenhydramine before your anti-B1 antibody dose to help control pain and prevent allergic reaction. You will also be given either a potassium iodide solution or potassium iodide tablets to protect your thyroid gland from damage at least 24 hours before you receive the first dose of the Iodine -131 Anti-B1 Antibody (dosimetric dose). You will continue treatment with the potassium iodide for at least 14 days after you receive the second dose (therapeutic dose). You may also receive the drug meperidine if you have trouble with muscle stiffening (rigors) during or after your infusions.*** Four or five visits to a nuclear medicine physician will be required during this

period of time to administer the test dose of I-131-tositumomab, perform three gamma scans, and administer the therapeutic dose of I-131-tositumomab. As mentioned above, you will need to take potassium iodide for several weeks before and after I-131-tositumomab to prevent damage to your thyroid gland from the radioactive iodine. Depending on the regulations in your state, you may require radiation isolation in the hospital for two to four days following the radiolabeled antibody treatment. If your state permits outpatient therapy with I-131-tositumomab, you will be given special instructions by the nuclear medicine physician on how to limit exposure of other family members to the radioactivity which has been given to you. Patients who have had bad side effects with the test dose will not receive the antibody treatment. If your disease or symptoms get worse, you will stop treatment on this study and be offered other treatment by your doctor.

If you take part in this study, you will have the following tests and procedures:

During your therapy, you will have x-rays, laboratory and other tests, including blood tests and bone marrow exams. You will have blood tests about every three weeks during the CHOP therapy and at the end of your CHOP treatment so your doctor can see how your body is responding to the therapy.

If you are assigned to Arm 3 and receive the I-131 anti-B1 antibody, blood tests will be done weekly during and after the Iodine-131 anti-B1 antibody until your blood counts return to normal.

You will also have your bone marrow examined (called "bone marrow aspiration and biopsy") at the start of this study, four to eight weeks after completion of therapy, one year after treatment, and annually thereafter as long as your lymphoma does not progress. Your skin over your hipbone will be numbed by a shot of local anesthetic (lidocaine) given just under your skin. A needle will be inserted through the numbed skin and into the hipbone. The bone marrow will be removed by using suction and a twisting motion of the needle. You may have minor discomfort, and minor infection is also possible. Rarely allergic reactions to the anesthetic may occur.

The bone marrow will be looked at to determine if any lymphoma cells are present. In addition, a portion of the bone marrow taken at the start of the study and again at one year after you finish treatment will be sent to a special laboratory for testing to detect chromosome breakages (translocations). This laboratory testing is for research purposes only and will not affect your cancer treatment.

How Long Will I Be in the Study?

We think it will take you about 4 - 5 months to complete any of the three different treatments. You will then return to your doctor about 2 months and about 7 - 8 months after you complete your treatment for tests and scans. After that ***at a***

minimum, you will return for a follow-up visit to your doctor every 6 months for two years and then once a year after that. ***Your doctor may wish to see you at more frequent intervals.***

The researcher may decide to take you off this study if your disease gets worse despite the treatment; the side effects of the treatment are too dangerous for you; new information about the treatment becomes available and this information suggests the treatment will be ineffective or unsafe for you. It is unlikely, but the study may be stopped early due to lack of drug supply or lack of funding.

WHAT ARE THE RISKS OF THE STUDY?

While on the study, you are at risk for these side effects. You should discuss these with the researcher and/or your regular doctor. There also may be other side effects that we cannot predict. Other drugs will be given to make side effects less serious and uncomfortable. Many side effects go away shortly after treatments are stopped, but in some cases side effects can be serious or long-lasting or permanent.

Risks and side effects related to the CHOP chemotherapy treatment (cyclophosphamide, doxorubicin, vincristine, prednisone) include the following:

Very Likely

- Nausea/vomiting
- Hair loss
- Decrease in appetite
- Decrease in blood cell counts
- Facial and/or abdominal swelling/puffy appearance

Less Likely

- Allergic reaction (may include rash, itching, swelling, cough, lowered blood pressure)
- Inflammation of the blood vessels in the skin where the drugs are given
- Sores in the mouth
- Discoloration of skin, nails
- Loosening of fingernails, toenails
- Facial flushing
- Itching of skin
- Headache
- Abdominal pain
- Jaw pain

- Brittle bones
- Diarrhea/constipation
- Fever
- ***Infection***
- Chills
- Fatigue
- Lower or higher blood pressure
- Muscle weakness
- Tingling in the arms and legs
- Itchy, swollen eyes
- Watery eyes
- Changes in eyesight
- EKG changes
- Bladder irritation (avoided by drinking 8-10 glasses of water a day)
- Change in color of urine
- Dizziness
- Mood swings/depression
- Changes in personality
- Menstrual changes

Less Likely, But Serious

- Scarring of lungs/shortness of breath
- Convulsions
- Heart failure
- Chance of acute leukemia

Risks and side effects related to the antibody rituximab include the following:

Very Likely

- Fever
- Chills
- Rigors (muscle stiffening)
- Headache
- Muscle Pain
- Malaise ***(bodily discomfort)***
- Nausea/vomiting
- Loss of appetite
- Flushing
- Fatigue
- Cough
- Shortness of breath
- Sensation of tongue or throat swelling
- Inflammation of the nose

- Dizziness
- Depression
- Anxiety
- Night sweats
- Tumor site pain

Less Likely

- Weakness
- Rash
- Itching
- Joint pain
- Diarrhea
- Greatly lowered red blood cell counts
- Lowered white blood cell counts
- Lowered platelet counts
- Faster or slower heartbeat
- Lowered blood pressure
- Increased blood pressure
- Numbness, tingling, burning sensations
- Swelling in the limbs (arms and legs)
- Agitation
- ***Trouble sleeping***
- Trigeminy
- Decreased immunoglobulins - B cells

Less Likely, but Serious

- Tumor Lysis syndrome (only a risk in patients with large numbers of circulating lymphocytes)
- Pulmonary failure
- Chest pain
- Heart attack
- Seizure
- Anapylaxis (difficulty breathing)

Risks and side effects related to the antibody tositumomab include the following:

Very Likely

- Fever
- Weakness
- Chills
- Loss of appetite
- Nausea/vomiting

- Diarrhea
- Rash
- Tumor site pain
- ***Decrease in blood counts***

Less Likely

- Lowered blood pressure causing lightheadedness/dizziness
- Allergic reaction including rash, hives, itching, shortness of breath
- Lower blood cell counts
- Development of "human anti-mouse antibodies" (limiting ability for further treatment with antibodies)
- Infection
- Joint pain
- Muscle pain
- Abdominal pain
- Headache
- Nose inflammation
- Throat inflammation
- Cough
- Diarrhea

Less Likely, but Serious

- Severe allergic reaction
- Bone marrow damage
- Chance of developing acute leukemia or other cancers

Reproductive risks: Because the drugs in this study can affect an unborn baby, you should not become pregnant or father a baby while on this study. You should not nurse your baby while on this study. Ask about counseling and more information about preventing pregnancy. *[Include a statement about possible sterility when appropriate.]*
[Attach additional information about contraception, etc.]

For more information about risks and side effects, ask the researcher or contact
____________________________________.

ARE THERE BENEFITS TO TAKING PART IN THE STUDY?

We cannot and do not guarantee you will benefit if you take part in this study. The treatment you receive may even be harmful. Your doctors feel that your participation in this study will give you at least as good a chance as you might expect from other treatments. We hope the information learned from this study will benefit other patients with Non-Hodgkin's lymphoma in the future.

The possible benefits of taking part in the study are the same as receiving CHOP chemotherapy with or without an antibody without being in the study.

WHAT OTHER OPTIONS ARE THERE?

Instead of being in this study, you have these options:
You may receive chemotherapy as recommended by your doctor for your type of lymphoma, the rituximab antibody alone, radiation therapy or a stem cell transplant. Also you may choose no anti-cancer treatment at this time (with care to help you feel more comfortable).

You can get treatment for Non-Hodgkin's lymphoma without being on this study. All of the treatment on this study may be available at this center or at other locations.

Please talk to your regular doctor about these and other options.

WHAT ABOUT CONFIDENTIALITY?

Efforts will be made to keep your personal information confidential. We cannot guarantee absolute confidentiality. Your personal information may be disclosed if required by law.

Organizations that may inspect and/or copy your research records for quality assurance and data analysis include groups such as: the National Cancer Institute, the Food and Drug Administration, Coulter Pharmaceuticals, Genentech Pharmaceuticals and the Southwest Oncology Group.

If we publish the information we learn from this study in a medical journal, you will not be identified by name or in any other way.

WHAT ARE THE COSTS?

Taking part in this study may lead to added costs to you or your insurance company. Please ask about any expected added costs or insurance problems.

In the case of injury or illness resulting from this study, emergency medical treatment is available but will be provided at the usual charge. No funds/funds have been set aside to compensate you in the event of injury. *(local institutions must choose the option that best fits the hospital's situation)*

You or your insurance company will be charged for continuing medical care and/or hospitalization.

You will receive no payment for taking part in this study.

Administration of the drug will be (provided free of charge/charged in the usual way). The parts of the research consisting of keeping research records will be paid by those organizing and conducting the research. The research requires that you receive certain standard medical tests and examinations. These standard tests and examinations will be (charged in the usual way/provided at a reduced rate). *(local institutions must choose the option that best fits the hospital's situation)*

The drugs cyclophosphamide, doxorubicin, and vincristine, prednisone ***and rituximab*** are commercially available. Tositumomab is considered investigational for this study and will be supplied free of charge by Coulter Pharmaceutical ***until it becomes commercially available***.

Although the tositumomab is provided free of charge for this study, extra charges may be incurred for its administration.

What Are My Rights as a Participant?

Taking part in this study is voluntary. You may choose not to take part or may leave the study at any time. Leaving the study will not result in any penalty or loss of benefits to which you are entitled. You can stop participating at any time. However, if you decide to stop participating in the study, we encourage you to talk to the researcher and your regular doctor first.

A Data Safety and Monitoring Board, an independent group of experts, will be reviewing the data from this research throughout the study. We will tell you about important new information from this or other studies that may affect your health, welfare, or willingness to stay in this study.

Whom Do I Call if I Have Questions or Problems?

For questions about the study or a research-related injury, contact the researcher *NAME(S)* at *TELEPHONE NUMBER*.

For questions about your rights as a research participant, contact the *NAME OF CENTER* Institutional Review Board (which is a group of people who review the research to protect your rights) at *TELEPHONE NUMBER*. *[And, if available, list patient representative (or other individual who is not on the research team or IRB).]*

WHERE CAN I GET MORE INFORMATION?

[To IRB/Investigators: Attach information materials and checklist of attachments. Signature page should be at the end of package. You may also wish to include the following informational resources]

You may call the NCI's Cancer Information Service at
1–800–4–CANCER (1–800–422–6237) or TTY: 1–800–332–8615

Visit the NCI's Web sites...
cancerTrials: comprehensive clinical trials information
http://cancertrials.nci.nih.gov.

CancerNet™: accurate cancer information including PDQ
http://cancernet.nci.nih.gov.

You will get a copy of this form. You may also request a copy of the protocol (full study plan).

SIGNATURE

You are deciding whether or not to take part in this study. If you sign, it means that you have decided to volunteer to take part in this study, and that you have read and understood all the information on this form.

Participant ________________________________ Date ____________________

Consent for use of excess diagnostic tissue for research purposes.

Preserved tissue from your tumor must be submitted for this study in order to confirm your type of non-Hodgkin's lymphoma. There may be some tissue remaining once your diagnosis has been confirmed.

If you are willing to allow this excess tissue to be used for unspecified, future research studies, please specify your consent below.

The results of tissue research may help find new ways to learn about, prevent, or treat cancer and other diseases. Please read each sentence below and think about your choice. After reading each sentence, circle the answer that is right for you. If you have any questions, please talk to your doctor or nurse, or call the National Cancer Institute's Cancer Information Service at 1/800-422-6237 (1/800-4-CANCER).

1) My tissue (specimen) may be kept for use in research to learn about, prevent or treat cancer.

Yes__________ No.________ Initial________

2) Someone from ________ may contact me in the future to ask me to take part in more research.

Yes__________ No.________ Initial________

3) My tissue (specimen) may be kept for use in research to learn about, prevent, or treat other health problems (for example: diabetes, Alzheimer's disease, or heart disease).

Yes__________ No.________ Initial________

If you are willing to submit additional frozen and preserved tissue for unspecified future research purposes, you will be registered to the Central Lymphoma Repository Tissue Procurement Protocol, SWOG-8819. Please specify your consent below.

The results of tissue research may help find new ways to learn about, prevent, or treat cancer and other diseases. Please read each sentence below and think about your choice. After reading each sentence, circle the answer that is right for you. If you have any questions, please talk to your doctor or nurse, or call the National Cancer Institute's Cancer Information Service at 1/800-422-6237 (1/800-4-CANCER).

1) My tissue (specimen) may be kept for use in research to learn about, prevent or treat cancer.

Yes__________ No.________ Initial________

2) Someone from ________ may contact me in the future to ask me to take part in more research.

Yes__________ No.________ Initial________

3) My tissue (specimen) may be kept for use in research to learn about, prevent, or treat other health problems (for example: diabetes, Alzheimer's disease, or heart disease).

Yes__________ No.________ Initial________

Southwest Oncology Group Statistical Center
1100 Fairview Avenue North, MP557
PO Box 19024
Seattle, WA 98109-1024
Patient Registration (206) 667-4623
CCOP Patient Registration (206) 652-2267

Southwest Oncology Group Operations Office
14980 Omicron Drive
San Antonio, TX 78245-3217
(210) 677-8808

Southwest Oncology Group Registration Form

SWOG Protocol Number	Registration Step	Tx Assignment	Activation Date:
S 0 0 1 6	1		Last Amended Date:

A Phase III Trial of CHOP Vs CHOP + Rituximab Vs CHOP + Iodine-131-Labeled Monoclonal Anti-B1 (Tositumomab) Antibody for Treatment of Newly Diagnosed Follicular Non-Hodgkin's Lymphomas

Patient Name ______________________

SWOG Patient Number ☐☐☐☐☐☐

INSTRUCTIONS: All of the information on this Registration Form and the Protocol Eligibility Section must be answered appropriately for a patient to be considered eligible for registration. This Registration Form must be entirely filled out and referred to during the registration. Do NOT submit this form as part of the patient data.

Caller's SWOG Roster ID ☐☐☐☐☐

SWOG Investigator Number ☐☐☐☐☐

SWOG Member/CCOP Number ☐☐☐☐☐

SWOG Affiliate/Component Number ☐☐☐☐☐

IRB Approval Date ☐☐ / ☐☐ / ☐☐☐☐

Date of Informed Consent ☐☐ / ☐☐ / ☐☐☐☐

Projected Start Date of Treatment ☐☐ / ☐☐ / ☐☐☐☐

Patient Consent for Specimen Use:

Research on cancer: ☐ Yes ☐ No

Contact for other research: ☐ Yes ☐ No

Research about other health problems: ☐ Yes ☐ No

Patient's Date of Birth: ☐☐ / ☐☐ / ☐☐☐☐

Patient's Race / Ethnicity: ☐☐ / ☐☐

Patient's Sex: ☐ Female ☐ Male

Method of Payment: ☐☐

Patient's Social Security Number: ☐☐☐ - ☐☐ - ☐☐☐☐

Patient's Zip Code (USA): ☐☐☐☐☐

Country of Residence (if not USA): ☐

Southwest Oncology Group Registration Form Code Sheet

Patient's race:

0 - Unknown	1 -Caucasian	2 - African American	3 - Native American
4 - Eskimo	5 -Aleut	6 - Chinese	7 - Filipino
8 - Hawaiian	9 - Korean	10 - Vietnamese	11 - Japanese
12 - Asian Indian	13 - Samoan	14 - Guamanian	15 - Hmong
16 - Fijian	17 - Laotian	18 - Thai	19 - Tongan
20 - Pakistani	21 - Cambodian	22 - Other API	23 - Other race

Patient's Ethnicity (Spanish/Hispanic Origin):

0 - Unknown	1 - No (not Spanish)	2 - Yes, Mexican	3 - Yes, Puerto Rican
4 - Yes, Cuban	5 - Yes, Central American		6 - Yes, South American
7 - Yes, Other	8 - Yes, NOS		

Method of Payment:

1 - Private	2 - Medicare	3 - Medicare and Private	4 - Medicaid
5 - Medicaid and Medicare		7 - No insurance (self-pay)	
8 - No insurance (no means)		9 - Other-specify________________	
10 - Unknown		11 - Veterans Admin	12 - Military

Other Group Registration Code:

9981 - NCIC	9982 - CALGB	9984 - GOG	9987 - MDACC
9995 - ECOG	9996 - NCCTG	9997 - RTOG	

SOUTHWEST ONCOLOGY GROUP
NON-HODGKIN'S LYMPHOMA (FOLLICULAR) PRESTUDY

SWOG Patient No. ☐☐☐☐☐☐ **SWOG Study No.** S 0 0 1 6 **Protocol Step:** 1

Patient Initials ______________ (L,F,M)

Institution/Member ______________________________ Physician ______________________________

Instructions: All dates are MONTH, DAY, YEAR. Explain any blank fields or blank dates in the Notes section at the bottom of the prestudy form. Place an [X] in appropriate boxes. Circle AMENDED items in red.

PATIENT AND DISEASE DESCRIPTION **Date of First Pathologic Diagnosis:** ☐☐ / ☐☐ / ☐☐☐☐

Height (cm): ☐☐☐ **Weight (kg):** ☐☐☐.☐ **BSA (m2):** ☐.☐☐ **Performance Status:** ☐

REAL Classification Histology
B Cell: ☐ Follicular Grade I ☐ Follicular Grade II ☐ Follicular Grade III

Current Stage of Disease: ☐ II ☐ III ☐ IV

Does the patient have bulky disease (any mass >= 10 cm in diameter or a mediastinal mass > 1/3 chest diameter)? ☐ No ☐ Yes

Symptoms: ☐ A (No Symptoms) ☐ B (Fever, Weight Loss, and/or Night Sweats)

CURRENT LABORATORY VALUES

LDH (U/l) ☐☐☐☐ **Albumin (gm/dl)** ☐☐.☐☐

LDH ULN ☐☐☐☐ **Serum Beta2 Microglobulin (mg/ml)** ☐☐.☐

CURRENT LYMPHATIC TISSUE INVOLVEMENT

Is there current nodal involvement above the diaphragm? ☐ No ☐ Yes

If yes, number of sites involved above the diaphragm*: ☐ 1 ☐ 2 ☐ >2

Is there current nodal involvement below the diaphragm? ☐ No ☐ Yes

If yes, number of sites involved below the diaphragm*: ☐ 1 ☐ 2 ☐ >2

Is there current splenic involvement? ☐ No ☐ Yes

* Bilateral disease in the same nodal pair is counted as one site

CURRENT EXTRANODAL INVOLVEMENT

Is there current extranodal involvement? ☐ No ☐ Yes

If yes, fill in box for each involved: ☐ Bone Marrow ☐ Lung ☐ Liver ☐ CNS/Brain ☐ GI Tract ☐ Other: ______________________

Notes

SOUTHWEST ONCOLOGY GROUP
LYMPHOMA BASELINE TUMOR ASSESSMENT FORM

Page 1 of 1

SWOG Patient No. ☐☐☐☐☐☐ SWOG Study No. S☐☐☐☐ Protocol Step: 1

Patient Initials ________ (L,F,M)

Institution / Member ____________________ Physician ____________________

Groups other than SWOG: Group Name/Study No./Pt. No. ________ / ________ / ________

Instructions: Please use black ink. Circle AMENDED items in red. Record the requested information for all measurable lesions and all sites of evaluable and non-evaluable disease. Please refer to section 10.1 of the protocol for definitions. If an organ or site has too many measurable lesions to measure at each evaluation, choose three to follow as measurable disease and record the rest as evaluable disease. For measurable lesions, check the RT-No Progression box if the lesion was previously irradiated and has not progressed since.
The same test procedures used for baseline disease assessment must be used for all required subsequent disease assessments

Site of Target Lesions

	Site of Measurable Lesions	RT No Progr.	Tumor Measurement (cm)	Assessment Code*	Date of Assessment
L1		☐	☐☐.☐ x ☐☐.☐	☐☐	☐☐/☐☐/☐☐☐☐
L2		☐	☐☐.☐ x ☐☐.☐	☐☐	☐☐/☐☐/☐☐☐☐
L3		☐	☐☐.☐ x ☐☐.☐	☐☐	☐☐/☐☐/☐☐☐☐
L4		☐	☐☐.☐ x ☐☐.☐	☐☐	☐☐/☐☐/☐☐☐☐
L5		☐	☐☐.☐ x ☐☐.☐	☐☐	☐☐/☐☐/☐☐☐☐
L6		☐	☐☐.☐ x ☐☐.☐	☐☐	☐☐/☐☐/☐☐☐☐

	Other Sites of Disease	Extent	Assessment Code*	Date of Assessment
S1			☐☐	☐☐/☐☐/☐☐☐☐
S2			☐☐	☐☐/☐☐/☐☐☐☐
S3			☐☐	☐☐/☐☐/☐☐☐☐
S4			☐☐	☐☐/☐☐/☐☐☐☐
S5			☐☐	☐☐/☐☐/☐☐☐☐

List all negative diagnostic tests/studies used to evaluate patient for malignancy.

Tests/studies	Date	Tests/studies	Date
	☐☐/☐☐/☐☐☐☐		☐☐/☐☐/☐☐☐☐
	☐☐/☐☐/☐☐☐☐		☐☐/☐☐/☐☐☐☐

* Assessment Codes:

01-Palpation
02-Visualization
03-Colposcopy
04-CA-125 assay
05-Endoscopy
10-Plain film/X-ray without contrast
11-Plain film/X-ray with contrast
12-CT scan
13-MRI scan
14-Radioisotope scan
15-Ultrasound
20-Histologic confirmation
21-Cytologic confirmation
99-Other (specify below and indicate lesion number)

FLOW SHEET S0016

PT. # ____ REG DATE ____
UNIT # ____ PAGE ____
PATIENT ____
INVESTIG. ____
INSTITUTION ____
STUDY # S0016 RX. # ____
DISEASE CATEGORY NHL

PROGRESS NOTES (DATE EACH)

DATE Mo./Day					
DAY ON STUDY					
**TREATMENT**					
WEIGHT (KG) (LB)					
HEIGHT (CM) (IN)					
BSA					
Cyclophosphamide					
Doxorubicin					
Vincristine					
Prednisone					
Rituximab antibody					
Coulter Training Session					
SSKI, Lugol's or Potassium Iodide (circle one)					
Dosimetric: Anti-B1 antibody predose(mg)					
Dosimetric: Anti-B1 antibody hot dose(mg)					
Dosimetric: Iodine-131dose (mCi prescribed)					
Therapeutic: Anti-B1 antibody predose(mg)					
Therapeutic: Anti-B1 antibody hot dose(mg)					
Therapeutic: Iodine-131 dose (mCi prescribed)					
G-CSF/GM-CSF (if given)					
Allopurinol					
Other supportive medications					
**MARROW BIOPSY**					
CELLULARITY					
TUMOR CELLS					
**BLOOD**					
HGB					
HCT					
PLTS/μl (ILLN=)					
WBC/μl					
NEUTROPHILES %					
LYMPHOCYTES %					
MONOCYTES %					
EOSINOPHILES					
Circulating lymphoid cells					
**LAB**					
*Serum Creatinine (ULN=)					
*SGOT (ULN=)					
Alkaline Phosphatase (ULN=)					
LDH (ULN=)					
*Bilirubin (ULN=)					
Urinalysis					
Uric acid					
Beta2 microglobulin (IULN=)					
Thyroid Stimulating Hormone (TSH)					
Immunophenotyping (CD20)					
Bone marrow asp./biopsy					
Specimens for pathology review					
Bone marrow submission for bcl2 testing					
**X-RAYS AND SCANS**					
Chest x-ray or CT scan of chest					
CT of Abdomen and pelvis					
Other radiographic tests					
EKG					
MUGA					
**PHYSICAL**					
TEMP					
INFECTION					
**LESIONS**					
#1					
#2					
#3					
#4					
#5					
#6					
**SYMPTOMS**					
Performance Status					

* List institutional lower limit of normal for platelets and upper limit of normal for laboratory tests as indicated.

FLOW SHEET S0016

DATE Mo./Day					
DAY ON STUDY					
**TOXICITY**					
Hematologic-other(specify)					
Cardiac-dysrythmia					
Cardiac EF/CHF					
Cardiac-ischemia					
Cardiac - other (specify)					
Hypertension					
Hypotension/Disorientation					
Edema					
Skin rash					
Blistering					
Pruritis					
Erythema					
Pigmentation changes					
Dermatologic-other(specify)					
Sterility					
Menses					
Cushingnoid					
Endocrine-other(specify)					
Conjunctivitis					
Glaucoma					
Eye-other(specify)					
Fever					
Chills					
Myalgia/Arthralgia					
Sweats					
Facial flushing					
Flu-like symptoms (spec)					
Nausea/Vomiting					
Diarrhea/Constipation					
Ileus					
Allergy					
Immnunosensitivity Reaction					
Immuno-other(specify)					
Dyspnea					
Pulmonary Fibrosis					
Hyponatremia					
Hypokalemia					
Hypocalcemia					
Stomatitis					
Pharynx/Esophagitis					
Other mucositis(specify site)					
Sommolence					
Personality change/Dizziness					
Convulsions					
Depression					
CNS-other(specify)					
Reflexes/weakness					
Incoordination/Ataxia	/	/	/	/	/
Paresthesia					
Neurosensory-other(specify)					
Headache					
Taste					
Neuro-other (specify)					
Abdominal pain					
Other pain (specify)					
Insomnia					
Hemorrhagic cystitis					
Bladder-other(specify)					
Renal - other (specify)					
Alopecia					
Anorexia					
Miscellaneous-other(specify)					

PT. # ______ REG DATE ______
UNIT # ______ PAGE ______
PATIENT ______
INVESTIG. ______
INSTITUTION ______
STUDY # S0016 RX. # ______
DISEASE CAT NHL

PROGRESS NOTES (DATE EACH)

SOUTHWEST ONCOLOGY GROUP
PATHOLOGY SUBMISSION FORM

SWOG Study No. S 0 0 1 6

Protocol Step 1

Instructions: Submit a copy of this form to the SWOG Statistical Center. Mail required materials and this form to the following pathologist:

Thomas M. Grogan, MD
Department of Pathology
Arizona Cancer Center
1501 N. Campbell Avenue
Tucson, AZ 85724-0001

SWOG Patient No.: ☐☐☐☐☐☐ Patient Initals __________ (L,F,M)

Institution / Member ______________________ Physician ______________________

Reason for Submission: ☐ Prestudy

No. of Slides: ☐☐ **No. of Blocks:** ☐☐ **Date Sample Obtained:** (month, day, year) ☐☐ / ☐☐ / ☐☐☐☐

Required Materials:
(if not enclosed, explain below in Notes)

	**Enclosed**		**Specimen #**
Representative H&E Stained Slides Original Diagnostic Specimen	☐ Yes	☐ No	__________
1 Representative Paraffin Block	☐ Yes	☐ No	__________
Pathology Report	☐ Yes	☐ No	

Notes:

4/3/2000

Draft

SOUTHWEST ONCOLOGY GROUP
SPECIMEN SUBMISSION FORM

SWOG Patient No. ☐☐☐☐☐☐ **SWOG Study No.** ☐☐☐☐☐ **Reg Type:** ☐

Patient Initials ____________ (L, F, M) **Disease** ____________

Institution/Member ____________ **Physician** ____________

Contact Person at Institution ____________ **Telephone No.** ____________

Groups other than SWOG: Group Name/Study No./Pt. No. ______/______/______

Instructions: Submit a SEPARATE Specimen Submission Form with EACH specimen type (e.g.- blood and bone marrow are separate submissions). Check protocol for submission details. All dates are **MONTH, DAY, YEAR.** Explain any blank fields or dates in the Notes section at the bottom of the form. Place an [X] in appropriate boxes. Circle **AMENDED** items in red.

SPECIMEN

Type of Specimen: (Check only one)

- ☐ Tubes of blood
- ☐ Tubes of bone marrow
- ☐ Tubes of serum
- ☐ Tissue, specify site(s): ____________
  - check one: ☐ fresh ☐ frozen ☐ paraffin embedded
- ☐ Slides, type and number: ____________
- ☐ Karyotype(s), number: ____________
- ☐ Other, specify: ____________

Date specimen collected: (month, day, year) ☐☐ / ☐☐ / ☐☐☐☐

Time specimen collected: (24 hour time) ☐☐ : ☐☐

REASONS FOR SPECIMEN SUBMISSION

(Check all that apply)

STATUS	TREATMENT STUDY NO.
☐ Prestudy specimen	☐☐☐☐☐☐
☐ Complete remission/response specimen	☐☐☐☐☐☐
☐ Relapse/recurrence specimen	☐☐☐☐☐☐
☐ Other specimen, specify: ____________	☐☐☐☐☐☐

By: ____________ Date: ____________

Notes from submitting institution:

For Central Laboratory Use Only

Note - Central Laboratory: Complete and return form to SWOG Statistical Center

Central laboratory identification number: ☐☐☐

Date specimen received: ☐☐ / ☐☐ / ☐☐☐☐

Time specimen received: ☐☐ : ☐☐

Condition of specimen (check only one):

- ☐ usable as received
- ☐ not usable as received; adequate submission
- ☐ not usable as received; inadequate submission

By: ____________ Date: ____________

Notes from central laboratory:

Southwest Oncology Group

OFF TREATMENT NOTICE

SWOG Patient No. [| | | | |] SWOG Study No. [S| | | |] Protocol Step [ ]

Disease Committee ____________________ Patient Initials (L, F, M) __________

Institution / Member ____________________ Physician ____________________

Groups other than SWOG: Group Name/Study No./Pt. No. __________ / __________ / __________

INSTRUCTIONS: For each protocol step, submit this form within 2 weeks after completion (or discontinuation) of treatment. List protocol-directed treatments that the patient received.

Chemotherapy: List regimens, start and stop dates. For multidrug regimens, do not list individual drugs separately; stop date would be the date all drugs in the regimen were discontinued.

Surgery: List type of surgery and in the "stop" column the date of surgery.

Radiation: List sites, start and stop dates (inclusive of boosts and implants).

Indicate an unknown part of a date with a horizontal line drawn across the appropriate boxes.

Start Date (mm,dd,yyyy)	Stop Date (mm,dd,yyyy)	REGIMEN or PROCEDURE or SITE(S)
/ /	/ /	
/ /	/ /	
/ /	/ /	
/ /	/ /	
/ /	/ /	

(If more room is needed, please continue on a separate page)

Reason OFF TREATMENT (select one)

- ☐ Treatment completed per protocol
- ☐ Toxicity, medically required, specify:
- ☐ Patient refused, due to toxicity, specify:
- ☐ Patient refused, other than toxicity, specify:
- ☐ Progression or relapse. Sites:
- ☐ Death (attach Notice of Death form)
- ☐ Other, specify: ____________________

Date OFF TREATMENT
Date of completion, progression, death or decision to discontinue therapy [|] / [|] / [| | |]

Will patient receive FURTHER TREATMENT?
☐ No ☐ Yes, specify: ____________________ ☐ Unknown

Date of Last Contact (or death): [|] / [|] / [| | |]

VITAL STATUS: ☐ Alive ☐ Dead (attach Notice of Death form)

Notes:

BY: ____________________ DATE: ____________________

NOTICE OF DEATH

SWOG Patient No. ☐☐☐☐☐☐ Most Recent SWOG Study No. S☐☐☐☐

Disease Committee ____________ Patient Initials (L, F, M) ________

Institution / Member ____________ Physician ____________

Groups other than SWOG: Group Name/Study No./Pt. No. ______/______/______

INSTRUCTIONS: Submit within 4 weeks of knowledge of death. AMENDED data: ☐ Yes, mark amended items in red.

Date of Death ☐☐ / ☐☐ / ☐☐☐☐ (month, day, year)

Causes of Death

Any cancer (check one)

☐ No ☐ Primary Cause ☐ Contributory ☐ Possible ☐ Unknown

If patient has had multiple tumor types, specify those which were causes of death:

____

____

____

Toxicity from disease related treatment (check one)

☐ No ☐ Primary cause ☐ Contributory ☐ Possible ☐ Unknown

If Primary Cause, Contributory or Possible, specify treatment and toxicity: ________

____

Non-cancer and non-treatment related causes (check one)

☐ No ☐ Primary cause ☐ Contributory ☐ Possible ☐ Unknown

If Primary Cause, Contributory or Possible, specify ________

____

Autopsy done? ☐ No ☐ Yes ☐ Unknown

Death information obtained from (check all that apply)

- ☐ Autopsy report
- ☐ Medical record / death certificate
- ☐ Physician
- ☐ Relative or friend
- ☐ Other, specify ________________

Notes:

BY: ____________ DATE: ____________

SOUTHWEST ONCOLOGY GROUP
FOLLOW UP FORM

SWOG Patient No. ☐☐☐☐☐☐ SWOG Study No. S☐☐☐☐ Protocol Step: ☐

Patient Initials (L, F, M) ______

Institution/Member ______ **Physician** ______

Groups other than SWOG: Group Name/Study No./ Pt. No. ______ / ______ / ______

Instructions: Please submit at each follow up after completion of treatment until recurrence, at time of recurrence, and at protocol specified intervals after recurrence. All dates are **MONTH, DAY, YEAR.** Answer all questions and explain any blank fields or blank dates in the **Notes** section. Place an X in appropriate boxes. Circle AMENDED items in red.

VITAL STATUS

Vital Status: ☐ Alive ☐ Dead **Date of last contact or death:** ☐☐ / ☐☐ / ☐☐☐☐

If vital status is Dead, complete and submit Notice of Death form.

DISEASE FOLLOW UP STATUS

Has the patient been assessed for this cancer since submission of the last form?

☐ No ☐ Yes. If Yes, Date of Assessment: ☐☐ / ☐☐ / ☐☐☐☐

NOTICE OF FIRST RELAPSE OR PROGRESSION

Has the patient had a first relapse or progression of this cancer that has not been previously reported?

☐ No ☐ Yes. If Yes, Date of Relapse: ☐☐ / ☐☐ / ☐☐☐☐

Site(s) of Relapse ______

NOTICE OF NEW PRIMARY

Has a new malignant neoplasm or myelodysplastic syndrome (MDS) been diagnosed since the last form?

☐ No ☐ Yes. If Yes, Date of New Primary: ☐☐ / ☐☐ / ☐☐☐☐

Specify New Primary Site ______

Comments:

19.2 DOSIMETRY INSTRUCTIONS AND WORKSHEETS

a DOSIMETRY INSTRUCTIONS

1. INTRODUCTION

The dosimetric methodology for determining the therapeutic Iodine-131 activity (dose) to be administered to the patient as Iodine-131 anti-B1 antibody involves the following two steps: (a) following administration of the dosimetric dose, sequentially measuring the whole-body elimination kinetics of the radiolabeled antibody with an appropriately collimated and calibrated gamma camera operated in whole body scanning mode [serial anterior whole body scans on Day 1 (within 1 hour of completion of Iodine-131 anti-B1 antibody infusion), Day 3, 4 or 5, and Day 7 or 8], and (b) calculating the Iodine-131 activity (therapeutic dose) to be administered to the patient. The following sections describe the required techniques for gamma camera validation, background determination, whole body counts, and calculating the Iodine-131 activity to be administered for therapy as Iodine-131 anti-B1 antibody.

2. GAMMA CAMERA PROCEDURES, QUALITY CONTROL, AND COUNTS

a. Gamma Camera and Dose Calibrator Procedures

The gamma camera can be either a single- or dual-head camera with a large or extra large field of view and can be equipped with either a high-energy or medium-energy parallel hole collimator suitable for performing whole body scans and whole body counts with Iodine-131. A 5 x 10^6 count ^{99m}Tc or ^{57}Co extrinsic flood image using the selected collimator should be obtained before the gamma camera/collimator system is used for dose calculations.

Institution- and/or manufacturer-specific quality control procedures for the gamma camera/computer system and the dose calibrator should be followed each day patient imaging is to be performed. The performance of the gamma camera and dose calibrator must be within performance guidelines. Steps must be taken to ascertain reasons for variation and corrective measures must be taken before injecting or imaging the patient.

b. Gamma Camera Quality Control

Camera sensitivity must be determined each day the patient is to be imaged by scanning a calibrated activity of Iodine-131. This is accomplished by obtaining an Iodine-131 source (approximately 200-250 μCi) at the time the first scanning is to be initiated per patient, placing it in a glass or plastic 10cc or 20-cc normal saline vial (as used for dilutions), adding 10 - cc of water or saline, and sealing the vial. This 200-250 μCi source can be Iodine-131 anti-B1 antibody or other soluble Iodine-131. Note that by the final day, this source will contain approximately 100-150 μCi due to physical decay.

Check the source activity immediately before the scanning is to be done with an NIST-traceable-calibrated dose calibrator at the Iodine-131 setting. Record the activity.

Set up computer and camera as follows:

High-energy or medium-energy parallel hole collimator
Symmetric window centered on the 364 keV photopeak of Iodine-131 (314-414 keV)

Matrix: minimum 128 x 128

Scanning speed: 30-100 cm/minute

The same computer and camera setup, collimator, ROI, and scanning speed must be used for the patient and background scans. The scan should be obtained with the camera at a distance of 30 cm above the table to simulate patient imaging parameters and should be of the same length as the patient scans.
Draw a rectangular ROI around the entire field of view to obtain the source counts. Record the counts. Only anterior counts are used in calculations. The same ROI should be used for source, background, and whole body patient counts on each day. The counts should be done just before scanning the patient.

The collection of background counts is described in the next section. Following the collection of the background counts on each day, the background-corrected source count (defined as the source count minus the background count) is calculated. The counts per μCi are calculated by dividing the background-corrected source count by the calibrated activity of that day. For a specific camera and collimator, the counts per μCi should be relatively constant. If the ratio on a post-infusion day is within ±10% of the ratio from the infusion day (Day 1), the quality control is adequate and the patient can be scanned. When values vary more than 10% from the established ratio, the reason for the discrepancy must be ascertained and corrected before treating the patient.

c. <u>Background counts</u>
The background in the patient imaging room must be determined before the patient is scanned, as outlined below:

Maintain the same camera and computer setup, collimator, ROI, and scanning speed used for the quality control scanning of the calibrated source. The scan should be obtained with the camera at a distance of 30 cm above the table to simulate patient imaging parameters.

Draw a rectangular ROI around the entire field of view of the image to obtain the background counts (C_B). Record the background counts. Only anterior counts are used in calculations. The same ROI should be used for the background counts on each day. This should be done just before imaging the patient. The same ROI that is used for the background scans must be used for the patient whole body dosimetry.

If abnormally high background counts are measured, the source should be identified and, if possible, removed. Verify that the same scanning speed, the camera window setting, and collimator are being used for each scan before the background radioactivity level is re-measured. If abnormally low background counts are measured, it must be assured that the ROI is correctly delineated. Similarly, the camera energy window setting and collimator should be verified before repeating the background counts.

19.0 APPENDIX

19.1 This study will utilize the CTC (NCI Common Toxicity Criteria) version 2.X for toxicity and Adverse Event reporting. A copy of the CTC version 2.X can be downloaded from the CTEP home page (http://ctep.info.nih.gov). **All appropriate treatment areas should have access to a copy of the CTC version 2.X.**

19.2 Dosimetry Instructions and Worksheets

a. Dosimetry Instructions

b. Worksheet #1 - Gamma Camera Daily Quality Control

c. Worksheet #2 - Gamma Camera Whole Body Dosimetry

d. Table 1 - Maximum Effective Mass

e. Table 2 - Activity Hours

f. Graph 1 - Total Body Residence Time Estimation

19.3 Assessment of Bone Marrow Involvement

19.4 Clinical Site Order Forms

a. Clinical Site Order Form for non-Radiolabeled Anti-B1 Antibody

b. Clinical Site Order Form for Iodine-131 Anti-B1 Antibody

19.5 Nuclear Medicine Physician Questionnaire

The dosimetry worksheets for the first 3 patients at each clinical site must be submitted to Coulter Pharmaceutical, Inc. by fax to confirm that the calculations were performed correctly (fax: 650/225-1890). A dosimetry hotline will be maintained by Coulter to assist in calculation of the proper therapeutic dose (telephone: 650/225-1859, Teresa White).

d. Gamma Camera Whole Body Counts for Dosimetry

Anterior whole body counts should be performed immediately after the background counts on each of the 3 days (Day 1; Day 3, 4, or 5; and Day 7 or 8).

The following procedures must be used to determine the kinetics of Iodine-131 from the whole body. The whole body counts will be obtained at three timepoints (Day 1; Day 3, 4, or 5; and Day 7 or 8 post-infusion). On Day 1 ($\leq$1 hour from end of infusion), the whole body counts must be obtained before any bladder emptying has occurred. For the other two timepoints, the counts will all be obtained immediately following patient voiding. The patient scans are to be obtained immediately following the background determinations. Details are as follows:

Maintain the camera and computer set up from quality control and background determination.

Scan the anterior whole body. For any particular patient, the same gamma camera, collimator and scan speed must be used for all scans. Extremities should be included in the scans. To obtain proper counts, the arms should not cross over the body. Bring the camera head(s) as close to the patient as possible. The scans should be centered on the midline of the patient. Record the time of day that the counts are obtained. Draw a rectangular ROI around the entire field of view to obtain the anterior counts (C_A). Record the patient anterior counts. The same ROI should be used for the patient counts on each day. The patient scanning distance must always be the same.

3. CALCULATION OF IODINE-131 ACTIVITY (THERAPEUTIC DOSE) AND SAMPLE CALCULATION

a. Calculation of Iodine-131 Activity
The following equation is used to calculate the activity of Iodine-131 to administer to the patient as Iodine-131 anti-B1 antibody to achieve the desired total body dose of radiation (cGy):

Iodine-131 Activity (mCi) =

$$\frac{\textbf{Activity Hours (mCi h)}}{\textbf{Residence Time (h)}} \times \frac{\textbf{Desired Total Body dose (cGy)}}{\textbf{75 cGy}}$$

The methods for determining the activity hours (mCi h), residence time (h), and desired total body dose (cGy) are described below:

1. Activity Hours (mCi h)

In order to determine the activity hours (mCi h), look up the patient's maximum effective mass derived from the patient's sex and height in Table 1. Then, use either the patient's weight in kg or the maximum effective mass, whichever is less, to look up the value for activity hours (mCi h) in Table 2.

2. Residence Time (h)

In order to determine the residence time (h), the % injected activity remaining at the two post-dosimetric dose-imaging timepoints [calculated from the start of the infusion to the time of image acquisition on Day 3, 4, or 5, and Day 7 or 8] must be calculated. [For each timepoint, calculate the total body count by subtracting the background count from the anterior patient count.]

Once the background-corrected total body counts have been calculated for the two timepoints, the % injected activity remaining at each timepoint is calculated by dividing the total body counts from that timepoint by the total body counts from Day 1 and multiplying by 100.

The residence time can now be calculated by using the graphical method:

Graphical Method

The residence time (h) is determined by plotting the time from the start of the infusion and the % injected activity values for the last 2 imaging timepoints on Graph 1. A best-fit line is then drawn from 100% (the pre-plotted Day 0 value) through the 2 plotted points (if the line does not intersect the two points, one point must lie above the best-fit line and one point must lie below the best-fit line). The residence time (h) is read from the x-axis of the graph at the point where the fitted line intersects the horizontal 37% injected activity line.

3. Desired Total Body Dose (cGy)

The desired total body dose is 75 cGy for patients with a baseline platelet count of ≥ 150,000 cells/mm^3. The desired total body dose (cGy) is 65 cGy for patients with a baseline platelet count of 100,000 to 149,999 cells/mm^3.

b. Sample Calculation

RH is a 63-year-old, 5'6" male who weighs 90 kg. His baseline platelet count is 121,000 cells/mm^3 and his % injected activities from 1 h, 72 h, and 168 h were 100%, 50%, and 20%, respectively. From Table 1, his maximum effective mass is determined to be 88.5 kg. As his maximum effective mass is less than his weight, it is used to look up the value for activity hours from Table 2; the activity hours is determined to be 9490 mCi h. By plotting the % injected activity values on Graph 1, the residence time is determined to be 103 hours. As the patient's platelet count is between 100,000 and 149,999 cells/mm^3, the desired total body dose is 65 cGy. The equation for Iodine-131 activity is then solved as follows:

$$\textbf{Iodine-131 Activity (mCi)} = \frac{9490 \text{ mCi h}}{103 \text{ h}} \times \frac{65 \text{ cGy}}{75 \text{ cGy}} = 80 \text{ mCi}$$

Coulter
PHARMACEUTICAL

Patient Initials: ______
Site Number: ______
Patient Identification Number: ______

IODINE-131 ANTI-B1 ANTIBODY DOSIMETRY CALCULATION WORKSHEETS

Instructions for completing worksheets:

- **Enter all dates as MM/DD/YYYY and times as HH:MM based on a 24-hour clock (i.e. 13:30)**
- **Gamma Camera and settings should be consistent for all scans done (i.e. I-131 source, background, and patient counts)**
- **A preliminary dose estimate must be done after the second day of scans to determine if an additional vial may be required for the therapeutic dose**
- **When patient dose calculation is complete, fax all Worksheets to Coulter Pharmaceutical at 650-553-1890 for confirmation of calculations (for the first 3 patients at each site).**
- **If using a commercial radiopharmacy, fax Worksheet #2 to radiopharmacy, *after receiving confirmation of calculations.***

Worksheet #1 — Gamma Camera Daily Quality Control

STUDY DAY	Day 1	Day 3, 4, or 5	Day 7 or 8
**DATE**	______	______	______
**DOSE CALIBRATOR ACTIVITY**			
Time Measured	______	______	______
I-131 source activity (μCi_{I-131})	______	______	______
**GAMMA CAMERA COUNTS**			
CAMERA SETTINGS			
Camera Name: ______			
Collimator: ☐ Medium Energy ☐ High Energy			
Whole Body Scan Field of View (30-200 cm)	______	______	______
Camera Height from Table (anterior head: cm)	______	______	______
BACKGROUND COUNTS			
Time Started	______	______	______
TOTAL COUNTS: Anterior (C_{BA})	______	______	______
I-131 SOURCE (200-250μCi on Day 0)			
Time Started (t)	______	______	______
TOTAL COUNTS: Anterior (C_A)	______	______	______
**CALCULATIONS**			
1. Background Corrected Counts $C_S = C_A - C_{BA}$	______	______	______
2. Percent Initial Count $\%ISC = C_{S(2\ or\ 3)}/C_{S1} \times 100$	______	______	______
3. Counts per μCi $= C_S / \mu Ci_{I-131}$	______	______	______

Date Recorded/Initials ______ ______ ______

Coulter PHARMACEUTICAL

Patient Initials: ____________
Site Number: ____________
Patient Identification Number: ____________

Worksheet #2 — Gamma Camera Whole Body Dosimetry

A. Dosimetric Dose Infusion

Date of Infusion: ________________

Start Time (t_{INF}): __________ End Time: __________

B. Determination of Activity Hours

Patient gender: ☐ Male ☐ Female

Patient height: ________cm Patient weight: ________kg

Patient maximum effective mass (Table 1) ________kg

Is patient weight above maximum effective mass?

☐ Yes - Use maximum effective mass (from Table 1) to determine Activity Hours

☐ No - Use patient's actual weight to determine Activity Hours

Activity Hours (Table 2): ____________ mCi-hour

C. Determination of Total Body Dose (cGy)

Platelet count: ______________ cells/ mm^3

Date: ______________

Prescribed Total Body Dose:

☐ **65 cGy (for platelet count of 100,000 to 149,999 cells/mm^3)**

☐ **75 cGy (for platelet count of ≥150,000 cells/mm^3)**

D. Determination of Residence Time (h) using whole body gamma camera counts

**STUDY DAY**	**Day 1**	**Day 3, 4, or 5**	**Day 7 or 8**
**DATE**	________	________	________
**GAMMA CAMERA COUNTS**			
BACKGROUND COUNTS			
Time Started	________	________	________
TOTAL COUNTS: Anterior (C_{BA})	________	________	________
PATIENT TOTAL BODY COUNTS			
Time Started (t)	________	________	________
TOTAL COUNTS: Anterior (C_A)	________	________	________
**CALCULATIONS**			
1. Background Corrected Patient Counts $C_n = C_A - C_{BA}$	C_{1} =	C_{2} =	C_{3} =
2. Time from start of ^{131}I Anti-B1 infusion to start of Patient Counts $t - t_{INF}$	t_1 = ____ hr	t_2 = ____ hr	t_3 = ____ hr
3. Percent Injected Activity %IA = $C_{(2 \text{ or } 3)}/C_1$ X 100	$\%IA_1$ = 100%	$\%IA_2$ =	$\%IA_3$ =
Date Recorded/Initials	________	________	________

Residence Time (from Graph 1) = __________ hours

E. Calculation of Iodine-131 Anti-B1 Antibody Activity (*Date Calculated/Initials:* ________________)

$$^{131}\text{Iodine Activity (mCi)} = \frac{\text{Activity Hours (mCi h)}}{\text{Residence Time (h)}} \times \frac{\text{Prescribed Total Body Dose (cGy)}}{75\text{ cGy}}$$

$$= \frac{\_\_\_\_\_\_\text{ mCi h}}{\_\_\_\_\_\_\text{ h}} \times \frac{\_\_\_\_\text{ cGy}}{75\text{ cGy}} = \boxed{\_\_\_\_\_\_\text{ mCi}}$$

DATE AND TIME OF PLANNED ADMINISTRATION: ______________________________

Coulter
PHARMACEUTICAL

Patient Initials: ____________
Site Number: ____________
Patient Identification Number: ____________

Table 1
Maximum Effective Mass

**MEN**		
Height (ft—inches)	Height (cm)	**Maximum Effective Mass (kg)**
4'-5"	134.5	**40.5**
4'-6"	137.0	**44.2**
4'-7"	140.0	**47.9**
4'-8"	142.0	**51.6**
4'-9"	145.0	**55.3**
4'-10"	147.5	**59.0**
4'-11"	150.0	**62.7**
5'-0"	152.5	**66.3**
5'-1"	155.0	**70.0**
5'-2"	157.5	**73.7**
5'-3"	160.0	**77.4**
5'-4"	162.5	**81.1**
5'-5"	165.0	**84.8**
5'-6"	167.5	**88.5**
5'-7"	170.0	**92.2**
5'-8"	172.5	**95.8**
5'-9"	175.5	**99.5**
5'-10"	178.0	**103.2**
5'-11"	180.5	**106.9**
6'-0"	183.0	**110.6**
6'-1"	185.5	114.3
6'-2"	188.0	118.0
6'-3"	190.5	**121.7**
6'-4"	193.0	**125.4**
6'-5"	195.5	**129.0**
6'-6"	198.0	**132.7**
6'-7"	200.5	**136.4**
6'-8"	203.0	**140.0**
6'-9"	205.5	**143.8**
6'-10"	208.5	**147.5**
6'-11"	211.0	**151.2**
7'-0"	213.5	**154.9**

**WOMEN**		
Height (ft—inches)	Height (cm)	**Maximum Effective Mass (kg)**
4'-5"	134.5	**40.7**
4'-6"	137.0	**43.8**
4'-7"	140.0	**47.0**
4'-8"	142.0	**50.2**
4'-9"	145.0	**53.3**
4'-10"	147.5	**56.5**
4'-11"	150.0	**59.7**
5'-0"	152.5	**62.8**
5'-1"	155.0	**66.0**
5'-2"	157.5	**69.2**
5'-3"	160.0	**72.3**
5'-4"	162.5	**75.5**
5'-5"	165.0	**78.7**
5'-6"	167.5	**81.8**
5'-7"	170.0	**85.0**
5'-8"	172.5	**88.2**
5'-9"	175.5	**91.3**
5'-10"	178.0	**94.5**
5'-11"	180.5	**97.7**
6'-0"	183.0	**100.8**
6'-1"	185.5	104.0
6'-2"	188.0	107.2
6'-3"	190.5	**110.3**
6'-4"	193.0	**113.5**
6'-5"	195.5	**116.7**
6'-6"	198.0	**119.8**
6'-7"	200.5	**123.0**
6'-8"	203.0	**126.2**
6'-9"	205.5	**129.3**
6'-10"	208.5	**132.5**
6'-11"	211.0	**135.7**
7'-0"	213.5	**138.8**

Multiply pounds by 0.454 to obtain kilograms. Multiply inches by 2.54 to obtain centimeters. To calculate the maximum effective mass for patient heights not included in above table, use the following formulas:

Males: **Maximum Effective Mass (kg) = 65.76 + 1.452 (Ht. in cm - 152)**
Females: **Maximum Effective Mass (kg) = 62.34 + 1.247 (Ht. in cm - 152)**

Adapted from (K. Zasadny, R. Wahl, *et al.*, J Nuc Med 1995; 36(5):214. "Total Body Mass Lean").

Coulter
PHARMACEUTICAL

Patient Initials: ____________
Site Number: ____________
Patient Identification Number: ____________

Table 2
Activity Hours

Mass[1] (kg)	Activity Hours (mCi h)	Mass[1] (kg)	Activity Hours (mCi h)	Mass[1] (kg)	Activity Hours (mCi h)	Mass[1] (kg)	Activity Hours (mCi h)	Mass[1] (kg)	Activity Hours (mCi h)
40.0	**4638**	60.0	**6686**	80.0	**8670**	100.0	**10595**	120.0	**12463**
40.5	**4690**	60.5	**6737**	80.5	**8718**	100.5	**10643**	120.5	**12509**
41.0	**4743**	61.0	**6787**	81.0	**8767**	101.0	**10690**	121.0	**12556**
41.5	**4796**	61.5	**6838**	81.5	**8816**	101.5	**10738**	121.5	**12602**
42.0	**4848**	62.0	**6888**	82.0	**8864**	102.0	**10785**	122.0	**12648**
42.5	**4901**	62.5	**6938**	82.5	**8913**	102.5	**10833**	122.5	**12694**
43.0	**4953**	63.0	**6989**	83.0	**8961**	103.0	**10880**	123.0	**12741**
43.5	**5005**	63.5	**7039**	83.5	**9010**	103.5	**10927**	123.5	**12787**
44.0	**5057**	64.0	**7089**	84.0	**9058**	104.0	**10975**	124.0	**12833**
44.5	**5109**	64.5	**7139**	84.5	**9106**	104.5	**11022**	124.5	**12879**
45.0	**5160**	65.0	**7189**	85.0	**9154**	105.0	**11069**	125.0	**12925**
45.5	**5212**	65.5	**7238**	85.5	**9202**	105.5	**11116**	125.5	**12971**
46.0	**5264**	66.0	**7288**	86.0	**9251**	106.0	**11163**	126.0	**13017**
46.5	**5315**	66.5	**7338**	86.5	**9299**	106.5	**11210**	126.5	**13063**
47.0	**5366**	67.0	**7387**	87.0	**9347**	107.0	**11257**	127.0	**13109**
47.5	**5418**	67.5	**7437**	87.5	**9394**	107.5	**11304**	127.5	**13155**
48.0	**5469**	68.0	**7486**	88.0	**9442**	108.0	**11351**	128.0	**13200**
48.5	**5520**	68.5	**7536**	88.5	**9490**	108.5	**11398**	128.5	**13246**
49.0	**5571**	69.0	**7585**	89.0	**9538**	109.0	**11445**	129.0	**13292**
49.5	**5621**	69.5	**7634**	89.5	**9585**	109.5	**11492**	129.5	**13337**
50.0	**5672**	70.0	**7683**	90.0	**9633**	110.0	**11538**	130.0	**13383**
50.5	**5724**	70.5	**7733**	90.5	**9682**	110.5	**11585**	130.5	**13429**
51.0	**5775**	71.0	**7783**	91.0	**9730**	111.0	**11632**	131.0	**13474**
51.5	**5826**	71.5	**7833**	91.5	**9779**	111.5	**11678**	131.5	**13520**
52.0	5878	72.0	7883	92.0	9827	112.0	11725	132.0	13565
52.5	5929	72.5	7932	92.5	9875	112.5	**11771**	132.5	**13611**
53.0	**5980**	73.0	**7982**	93.0	**9924**	113.0	**11818**	133.0	**13656**
53.5	**6031**	73.5	**8031**	93.5	**9972**	113.5	**11864**	133.5	**13701**
54.0	**6082**	74.0	**8081**	94.0	**10020**	114.0	**11910**	134.0	**13747**
54.5	**6133**	74.5	**8130**	94.5	**10068**	114.5	**11957**	134.5	**13792**
55.0	6184	75.0	8180	95.0	10117	115.0	12003	135.0	13837
55.5	6234	75.5	8229	95.5	10165	115.5	12049	135.5	13882
56.0	6285	76.0	8278	96.0	10213	116.0	12095	136.0	13928
56.5	6335	76.5	8327	96.5	10261	116.5	12141	136.5	13973
57.0	6386	77.0	8376	97.0	10309	117.0	12187	137.0	14018
57.5	6436	77.5	8425	97.5	10357	117.5	12233	137.5	14063
58.0	6486	78.0	8474	98.0	10404	118.0	12279	138.0	14108
58.5	6536	78.5	8523	98.5	10452	118.5	12325	138.5	14153
59.0	6586	79.0	8572	99.0	10500	119.0	12371	139.0	14198
59.5	6636	79.5	8621	99.5	10548	119.5	12417	139.5	14242

[1] The minimum of the patient's actual weight (kg) or maximum effective mass (kg) from Table 1. For values between 140 kg and 160 kg, use the following formula:

Activity Hours (mCi h) = 14287 + (88.74) (Wt in kg - 140)

Coulter
PHARMACEUTICAL

Patient Initials: ____________
Site Number: ____________
Patient Identification Number: ____________

Graph 1
Total Body Residence Time Estimation

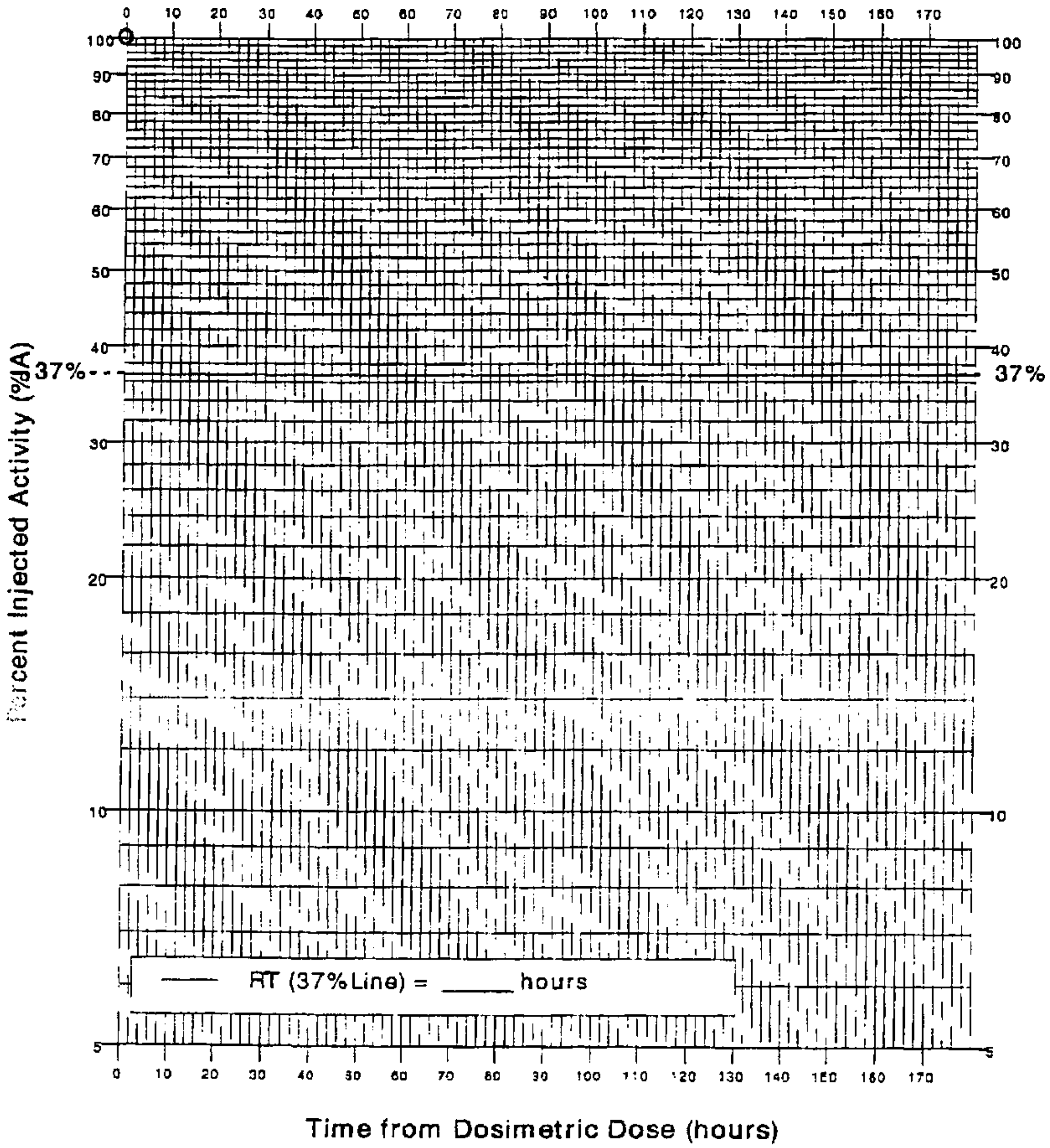

19.3 ASSESSMENT OF BONE MARROW INVOLVEMENT

Pathology procedure for determining percentage of bone marrow involvement with lymphoma by bilateral biopsy.

1. Bone marrow core biopsies are collected bilaterally. Results of a unilateral biopsy are acceptable if that result indicates that less than 10% of the intratrabecular space is lymphoma. The results of reading the two biopsies are averaged and extrapolated to the entire bone marrow. The core biopsy specimens are sectioned longitudinally if they are longer than 0.5 cm in length. Three or 4 of these sections for each biopsy specimen can be positioned on a slide, stained, and read. One slide is prepared per biopsy sample. If a biopsy specimen is 0.5 cm or less in length, then it is sectioned sagittally and about 10 to 12 sections of each biopsy are positioned on a slide, stained, and read. Again one slide is prepared from each specimen.

2. The sections for each patient are generally fixed, embedded, and stained using hematoxylin and eosin. Where the histopathology of the specimen is not clear with H & E, sections of the specimen will be analyzed for cell surface markers by immunohistopathology using antibodies, such as L26, which binds to the CD20 antigen. The monoclonal nature of lymphoma cells cannot be established on fixed specimens, as the kappa and lambda antigens are denatured by fixation.

3. The lymphoma disease of almost all patients occurs as foci of disease cells within the bone marrow. The percentage of the total field areas that are composed of these foci are estimated visually over each section. A few patients have diffuse disease which is more difficult to quantitate, but again the percentage of bone marrow area on sections which is involved with lymphoma is visually estimated. The mean percentage of the total fields consisting of lymphoma cells is calculated for the two biopsy specimens. It is assumed that all bone marrow has a similar level of infiltration with disease as the core biopsies.

19.4 Clinical Site Order Forms

a. Clinical Site Order Form For Non-Radiolabeled Anti-B1 Antibody

b. Clinical Site Order Form For Iodine-131 Anti-B1 Antibody

Clinical Site Order Form for Iodine-131 Anti-B1 Antibody

THIS FORM MUST BE FAXED TO

COULTER PHARMACEUTICAL, INC.
ATTENTION: TERESA WHITE

FAX No. (650) 553-1890

BY 4:00 P.M. Eastern Time Thursday of each week

Today's Date:_______________ (MM-DD-YY) Protocol No.: CP-00-044 (SWOG S0016)

Site Name:_______________

Shipping Address:_______________

____

Contact Name:_______________ (Please print)

Phone No.:_______________

Fax No.:_______________

ORDER FORM for Next Week: week beginning _______________ (Month - Day - Year)

Patient Initials	Date of Administration (Month - Day - Year)	Treatment *(check one box only per patient; order dosimetric and therapeutic doses for the same patient on separate order forms)* Dosimetric*	Therapeutic**
___ ___ ___	___________	☐	☐
___ ___ ___	___________	☐	☐
___ ___ ___	___________	☐	☐
___ ___ ___	___________	☐	☐

* A standard order of one (1) dosimetric vial per patient will be sent.

** A standard order of one (1) therapeutic vial per patient will be sent. At a later date, if the dosimetric scans indicate that a specific patient may require more than one therapeutic vial, immediately contact Teresa White immediately at (650) 553-1886.

For Coulter Pharmaceutical, Inc. (CPI) Use Only

Form received by CPI on: ___________ (Date) By: _______________ (Signature)

Form faxed to clinical site on: ___________ (Date) By: _______________ (Signature)

Form faxed to Commercial Radiopharmacy on: ___________ (Date) ☐ N/A By: _______________ (Signature)

Assigned Order Number:___________

SWOG S0016 Site Assessment
Nuclear Medicine / Radiation Oncology Investigator Questionnaire

**Name:**		**Degree:**	
**Title:**			
**Institution:**			
**Address:**			
**Address:**			
**City:**		**State:**	**Zip:**
**Telephone:**	( ) -	**Facsimile:**	( ) -
**Email:**			

Which department administers oral Iodine-131 therapy for thyroid cancer at your institution?

Nuclear Medicine ☐ **Radiation Oncology** ☐

Location of radiopharmacy used for preparation of Iodine-131 doses?

Nuclear Medicine ☐ **Radiation Oncology** ☐

Name of Nuclear medicine technologist: ____________________

Phone #: ____________________

Fax#: ____________________

Name of radiopharmacist: ____________________

Phone #: ____________________

Fax #: ____________________

Commercial RadioPharmacy ☐

Name of RadioPharmacy: ____________________

Contact Person: ____________________

Phone #: ____________________

Fax #: ____________________

Address: ____________________

Gamma Camera(s) available - check all applicable: Single Head ☐ Dual Head ☐

Collimator: High Energy ☐ Medium Energy ☐ Low Energy ☐

Has your institution implemented the new Nuclear Regulatory Commission (NRC) guidelines release of patients administered radioactive materials?

Yes ☐ No ☐ Would like to implement, need assistance ☐

Please forward this form, your curriculum vitae, and your institution's radioactive license to Coulter Pharmaceutical, Inc. Fax: (650) 553-1890

SOUTHWEST ONCOLOGY GROUP

A PHASE III TRIAL OF CHOP + RITUXIMAB VS CHOP + IODINE-131-LABELED MONOCLONAL ANTI-B1 ANTIBODY (Tositumomab) FOR TREATMENT OF NEWLY DIAGNOSED FOLLICULAR NON-HODGKIN'S LYMPHOMAS **(ARM 1, CHOP ONLY, OF THIS STUDY WAS PERMANENTLY CLOSED, EFFECTIVE 12/15/02)**

		Page
	SCHEMA	2
1.0	OBJECTIVES	3
2.0	BACKGROUND	3
3.0	DRUG INFORMATION	8
4.0	STAGING CRITERIA	18
5.0	ELIGIBILITY CRITERIA	19
6.0	STRATIFICATION FACTORS	22
7.0	TREATMENT PLAN	22
8.0	TOXICITIES TO BE MONITORED AND DOSAGE MODIFICATIONS	30
9.0	STUDY CALENDAR	34
10.0	CRITERIA FOR EVALUATION AND ENDPOINT DEFINITIONS	37
11.0	STATISTICAL CONSIDERATIONS	39
12.0	DISCIPLINE REVIEW	40
13.0	REGISTRATION GUIDELINES	40
14.0	DATA SUBMISSION SCHEDULE	42
15.0	SPECIAL INSTRUCTIONS	43
16.0	ETHICAL AND REGULATORY CONSIDERATIONS	45b
17.0	BIBLIOGRAPHY	48
18.0	MASTER FORMS SET	51
19.0	APPENDIX	81

STUDY COORDINATORS:

Oliver W. Press, M.D., Ph.D. (Medical Oncology)
Fred Hutchinson Cancer Research Center
1100 Fairview Avenue North, D3-190
Seattle, WA 98109
Phone: 206/667-1872
Fax: 206/667-1874
E-mail: press@u.washington.edu

David G. Maloney, M.D., Ph.D. (Medical Oncology)
Fred Hutchinson Cancer Research Center
1100 Fairview Avenue North, D1-100
Seattle, WA 98109
Phone: 206/667-5616
Fax: 206/667-6124
E-mail: dmaloney@fhcrc.org

Rita M. Braziel, M.D. (Hematopathology)
Department of Pathology, L471
Oregon Health Sciences University
3181 SW Sam Jackson Park Road
Portland, OR 97239
Phone: 503/494-2315
Fax: 503/494-8148
E-mail: braziel@ohsu.edu

AGENTS:

Cyclophosphamide (Cytoxan®) (NSC-26271)
Doxorubicin (NSC-123127)
Prednisone (NSC-10023)
Vincristine (Oncovin) (NSC-67574)
Iodine-131 Anti-B1 Antibody and Iodine-131 Anti-B1 Antibody (Tositumomab + I-131 tositumomab) (BB-IND-8283)
Rituximab Chimeric anti CD-20mab (IDEC-C2B8) (NSC-687451)

Michael LeBlanc, Ph.D. (Biostatistics)
Joseph M. Unger, M.S.
Southwest Oncology Group Statistical Center
Fred Hutchinson Cancer Research Center
1100 Fairview Avenue North, M3-C102
P.O. Box 19024
Seattle, WA 98109-1024
Phone: 206/667-4623
Fax: 206/667-4408
E-mail: mleblanc@fhcrc.org
E-mail: junger@fhcrc.org

mb

(Version Date 12/19/14)

CLOSED EFFECTIVE 09/15/2008

CALGB STUDY COORDINATOR (CALGB 50102):

Myron S. Czuczman, M.D.
Roswell Park Cancer Institute
Department of Medicine
Elm and Carlton Streets
Buffalo, NY 14263

ECOG STUDY COORDINATOR (ECOG S0016):

Sandra Horning, M.D.
Stanford Cancer Center
875 Blake Wilbur Drive, Site CC-2338
Stanford, CA 94305
Phone: 650/725-6496
FAX: 650/725-8222
E-mail: sandra.horning@stanford.edu

PARTICIPANTS

ALLIANCE/Alliance for Clinical Trials in Oncology

ECOG-ACRIN/ECOG-ACRIN Cancer Research Group

SWOG/SWOG

CLOSED EFFECTIVE 09/15/2008

SWOG Authorized Document

This study is supported by the NCI Cancer Trials Support Unit (CTSU).

Institutions not aligned with SWOG will participate through the CTSU mechanism as outlined below and detailed in the CTSU logistical appendix.

- The **study protocol and all related forms and documents** must be downloaded from the protocol-specific Web page of the CTSU
- Member Web site located at https://www.ctsu.org
- Send completed **site registration documents** to the CTSU Regulatory Office. Refer to the CTSU logistical appendix for specific instructions and documents to be submitted.
- **Patient enrollments** will be conducted by the CTSU. Refer to the CTSU logistical appendix for specific instructions and forms to be submitted.
- Data management will be performed by the Southwest Oncology Group. **Case report forms** (with the exception of patient enrollment forms)**, clinical reports, and transmittals** must be sent to the Southwest Oncology Group Data Operations Center unless otherwise directed by the protocol. Do not send study data or case report forms to the CTSU Data Operations.
- **Data query and delinquency reports** will be sent directly to the enrolling site by the Southwest Oncology Group. Please send query responses and delinquent data to the Southwest Oncology Group Data Operations Center and do not copy the CTSU Data Operations.
- Each site should have a designated CTSU Administrator and Data Administrator and must keep their CTEP AMS account contact information current. This will ensure timely communication between the clinical site and the SWOG data center.

CANCER TRIALS SUPPORT UNIT (CTSU) ADDRESS AND CONTACT INFORMATION

**To submit site registration documents:**	**For patient enrollments:**	**Submit study data directly to the Lead Cooperative Group unless otherwise specified in the protocol:**
CTSU Regulatory Office<br>1818 Market Street, Suite 1100<br>Philadelphia, PA 19103<br>Phone: 1-888/823-5923<br>Fax: 215/569-0206	CTSU Patient Registration<br>Voice Mail: 1-888/462-3009<br>Fax: 1-888/691-8039<br>Hours: 8:00 am – 8:00 pm EST, Monday – Friday (excluding holidays)<br><br>[For CTSU patient enrollments that must be completed within approximately one hour, or other extenuating circumstances, call 301/704-2376. Please use the 1-888/462-3009 number for ALL other CTSU patient enrollments.]	Southwest Oncology Group Data Operations Center<br>Fax: 1-800/892-4007<br>[Please do not use a cover sheet for faxed data.]<br><br>Do not submit study data or forms to CTSU Data Operations. Do not copy the CTSU on data submissions.
For treatment- or toxicity-related questions contact the Study PI of the Coordinating Group.		
For eligibility questions contact the Southwest Oncology Group Data Operations Center by phone or email: Phone: 206/652-2267; Email: lymphquestion@crab.org		
For questions unrelated to patient eligibility, treatment, or data submission contact the CTSU Help Desk by phone or e-mail: CTSU General Information Line: 1-888/823-5923, or ctsucontact@westat.com. All calls and correspondence will be triaged to the appropriate CTSU representative.		
**The CTSU Public Web site is located at: www.ctsu.org**<br>**The CTSU Registered Member Web site is located at https://www.ctsu.org**		

CTSU logistical information is located in Appendix 19.6

CLOSED EFFECTIVE 09/15/2008

SWOG Authorized Document

SCHEMA

REGISTRATION/RANDOMIZATION

ARM 1: CHOP ONLY | **ARM 2: CHOP+RITUXIMAB** | **ARM 3: CHOP + TOSITUMOMAB + I-131 Tositumomab**

(THE CHOP ONLY ARM OF THIS STUDY WAS PERMANENTLY CLOSED, EFFECTIVE 12/15/02.)

Arm 1: CHOP only

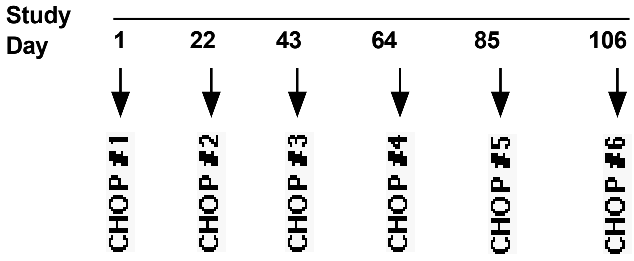

Arm 2: CHOP + Rituximab

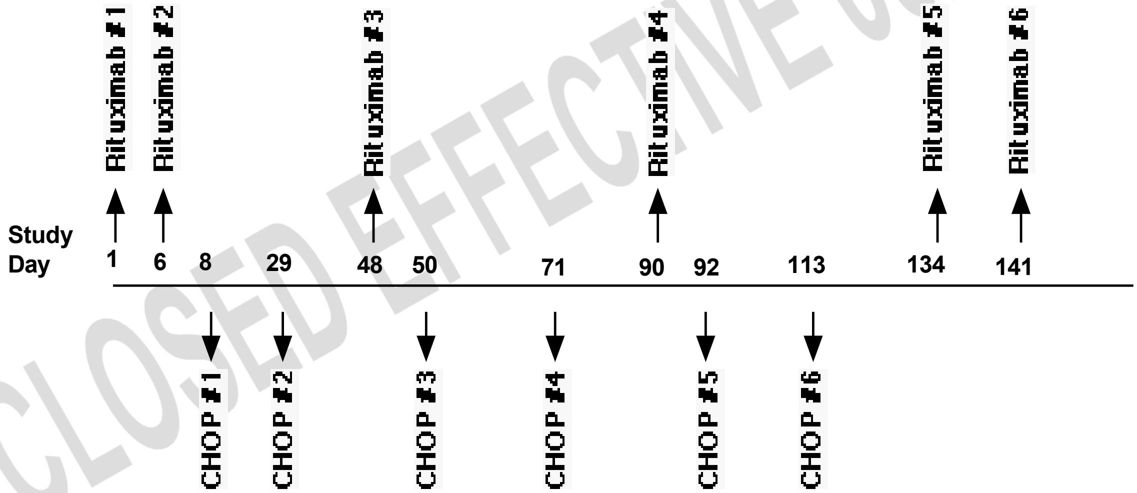

Arm 3: CHOP + Tositumomab + I-131 Tositumomab

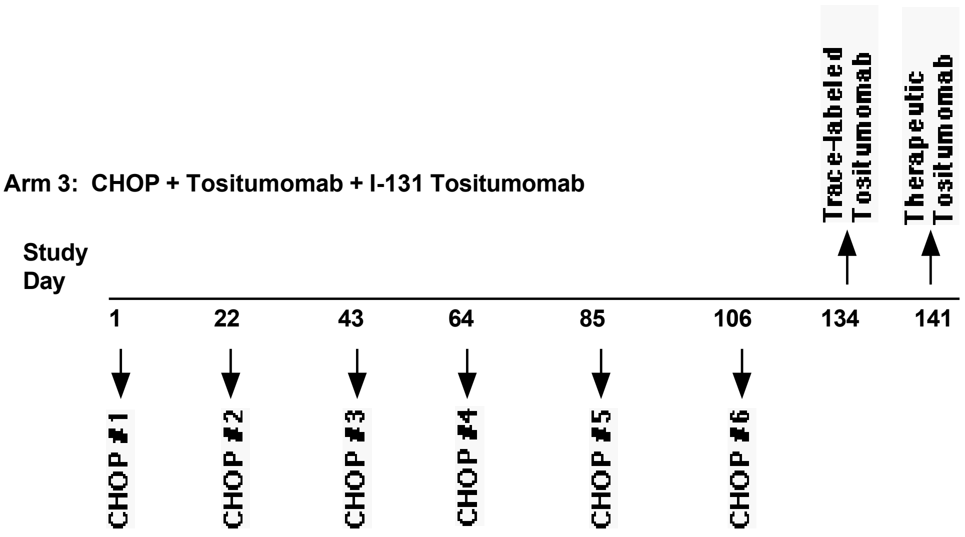

CLOSED EFFECTIVE 09/15/2008

SWOG Authorized Document

1.0 OBJECTIVES

1.1 To compare the progression-free survival and overall survival of patients with newly diagnosed follicular lymphoma (CD20+) treated with six cycles of CHOP chemotherapy alone, six cycles of CHOP with rituximab or six cycles of CHOP followed by Anti-B1 Antibody and Iodine-131 Anti-B1 Antibody (tositumomab + I-131 tositumomab). **(THE CHOP ONLY ARM OF THIS STUDY WAS PERMANENTLY CLOSED, EFFECTIVE 12/15/02.)**

1.2 To evaluate the response rate for patients with newly diagnosed follicular lymphoma (CD20+) treated with these regimens.

1.3 To evaluate the toxicities of CHOP with or without rituximab or Anti-B1 Antibody and Iodine-131 Anti-B1 Antibody in patients with newly diagnosed follicular lymphomas. **(THE CHOP ONLY ARM OF THIS STUDY WAS PERMANENTLY CLOSED, EFFECTIVE 12/15/02.)**

1.4 To compare the molecular remission rates by measuring clonal t(14;18)/bcl2 rearrangements in the bone marrow at baseline and at one year post-treatment.

1.5 To determine the incidence and time to development of human anti-mouse antibody (HAMA) positivity.

2.0 BACKGROUND

Follicular lymphomas are characterized by an indolent clinical course with predominant nodal and marrow involvement. With the exception of a small fraction of patients (10 - 15%) with truly localized involvement, the majority of low-grade lymphoma patients do not achieve long-term remissions. *(1, 2)* There is no consensus on the best treatment approach for follicular lymphoma. Many asymptomatic patients are followed with "watchful waiting", because conventional chemotherapy regimens have not been shown to improve survival in this disease compared with a policy of expectant therapy. *(1)* Symptomatic patients are commonly treated with oral alkylating agents (e.g., chlorambucil), or with an alkylating agent (cyclophosphamide) in combination with a vinca alkaloid and corticosteroids ("CVP"), but studies have not demonstrated a survival advantage for patients treated with these regimens. Previous Southwest Oncology Group studies have shown approximately 65% two-year progression-free survival for this patient group. Because of the curative potential of anthracycline-based regimens (e.g., CHOP) for intermediate and high grade Non-Hodgkin's lymphomas, CHOP has been tested extensively by the Southwest Oncology Group in patients with low-grade lymphomas. *(3 - 6)* Four hundred fifteen patients with low grade malignant lymphomas were treated with CHOP between 1972 and 1983 on **SWOG-7204**, **SWOG-7426**, and **SWOG-7713**. Approximately 90% of these patients achieved objective responses, including 61 - 78% with complete remissions. *(3 - 5)* With a median follow-up of 12.8 years, the median survival was 6.9 years. *(6)* Toxicities included myelosuppression, nausea, paresthesias, infection, and cardiomyopathy (< 5%). Unfortunately, these studies failed to demonstrate a plateau on the survival curves of patients treated with CHOP, indicating that by itself CHOP has little curative potential for low grade lymphomas, in contrast to its efficacy for diffuse large cell lymphomas. *(6)* Whether cures with CHOP are possible for patients with follicular large cell lymphoma remains controversial, with conflicting studies from several institutions. *(7 - 10)* Nevertheless, all investigators agree on the necessity of developing better treatment regimens for patients with follicular lymphomas.

New chemotherapeutic agents (fludarabine, 2-chlorodeoxyadenosine) have shown considerable activity in patients with relapsed low grade lymphomas; however, no study has demonstrated that these drugs are curative for follicular lymphomas or that survival is prolonged by their use. **SWOG-9501** investigated the combination of fludarabine plus mitoxantrone in patients with newly diagnosed follicular lymphomas. This regimen was well tolerated and produced a high response rate, but the survival curves do not appear superior to those obtained with other regimens, including CHOP. *(11)*

High-dose chemoradiotherapy with bone marrow or peripheral blood stem cell transplantation is commonly employed for treatment of patients with relapsed lymphomas of all types. Preliminary data from the Dana Farber Cancer Institute suggest the disease-free survival after transplantation for follicular lymphomas relates to the quality of initial morphological remission obtained and the ability to decrease the number of cells bearing clonal rearrangements of the bcl2-oncogene (which is present in 80% of newly diagnosed follicular lymphomas). *(12)* However, the curability

CLOSED EFFECTIVE 09/15/2008

SWOG Authorized Document

of follicular lymphomas with stem cell transplantation and the advisability of performing this procedure are controversial.

Clinical Studies with Rituximab

Rituximab is a chimeric mouse-human anti-CD20 monoclonal antibody which has been extensively evaluated as a single agent for the treatment of patients with multiply -relapsed B cell lymphoma. Data from a Phase I, dose-escalation study in 15 patients with relapsed NHL demonstrated no dose-limiting toxicity, two PRs, and four minor responses to a single infusion of the mAb using doses ranging from 10 to 500 mg/m^2. *(13)* The serum half-life at the higher dose levels ranged from 1.6 to 10.5 days and antibody levels > 10 ug/ml persisted for more than 14 days in 6 of 9 patients. Antibody infusion produced CD20 positive B-cell depletion in peripheral blood at 24 to 72 hours that persisted for 2 to 3 months in most patients. Flow cytometric evaluation of cell suspensions from lymph node biopsy specimens revealed B-cell depletion and tumor cells coated with antibody two weeks post-treatment.

A Phase I/II clinical trial utilized 4 weekly infusions of the antibody in doses ranging from 125 mg/m^2 to 375 mg/m^2. *(14)* There was no dose limiting toxicity observed in this trial and the 375 mg/m^2 dose was selected for Phase II and III clinical trials. In the Phase II portion of the trial, 37 patients with relapsed lymphoma were treated with 4 weekly infusions of rituximab at 375 mg/m^2 dose level. Overall, there was a 46% response rate. The median duration of these responses was 10.2 months. Three patients continue with ongoing response with durations of greater than 25 months. Tumor responses were observed in bulky lymph nodes, spleen, bone marrow, blood and extranodal masses. Toxicity was mild, with the majority of adverse events (67%) observed during the initial antibody infusion with a dramatically decreased incidence of any adverse events occurring with the subsequent antibody infusions. The most common adverse events include Grade 1 or 2 fever (73%), asthenia (16%), chills (38%), nausea (19%), vomiting (11%), rash (14%), and pain at tumor sites (3%). Grade 1 or 2 hypotension occurred in 3 patients (8%). The 37 patients received 142 antibody infusions, and 4 patients had events reported as Grade 3 or 4 (thrombocytopenia or neutropenia). B-cell depletion in the peripheral blood was observed in all patients and lasted approximately 6 months with gradual slow recovery.

A subsequent multi-center trial published by McLaughlin confirmed these findings, with 48% of 166 relapsed lymphoma patients (151 evaluable) achieving remissions with minimal toxicity (15), including 6% CRs. *(15)* The median response duration was approximately one year. Solal-Celigny has recently demonstrated that the response rates are substantially higher if newly diagnosed patients with follicular lymphoma are treated. *(16)* Of 50 previously untreated patients given rituximab, 28% achieved a complete remission and 46% a partial response, for an overall response rate of 74%.

Coiffier evaluated the efficacy of rituximab in 52 patients with aggressive lymphomas and demonstrated 5 complete and 12 partial remissions. *(17)* Approximately 30-35% of patients with diffuse large cell lymphomas and mantle cell lymphomas responded to the standard rituximab regimen in this study. These results have been confirmed in a recent multi-center European study treating 131 patients with newly diagnosed mantle cell lymphoma, immunocytoma, and small lymphocytic lymphoma. *(18)* The overall response rate was 30% (36 of 120 evaluable patients) including 37% responses in mantle cell lymphoma, 28% responses in immunocytoma, and 14% responses in small lymphocytic lymphoma.

Rituximab has been shown to deplete circulating B lymphocytes from the bloodstream for approximately six months after standard doses. Fortunately, this period of B cell depletion has not been associated with a high risk of infections, presumably because serum immunoglobulin levels remain near baseline levels. In addition, patients can be treated repetitively with chimeric anti-CD20 antibody since less than 1% of patients develop anti-chimeric antibody immune responses. Toxicities are usually limited to the infusional period with fever, chills, headache, pruritis, sweats, rash, and transient hypotension being most frequently observed. Classic toxicities associated with chemotherapy such as alopecia, nausea, and myelosuppression are rare with rituximab. However, rare fatal reactions have been observed, including adult respiratory

CLOSED EFFECTIVE 10/15/2008

SWOG Authorized Document

distress syndrome and tumor lysis syndrome. These serious complications appear to be more common in patients with large numbers of circulating malignant B cells and patients with pulmonary infiltration by lymphoma. *(19)* Hepatitis B virus (HBV) reactivation with fulminant hepatitis, hepatic failure, and death has been reported in some patients with hematologic malignancies treated with rituximab. The majority of patients received rituximab in combination with chemotherapy. The median time to the diagnosis was approximately 4 months after the initiation of rituximab and approximately one month after the last dose.

Despite the encouraging clinical results and success of rituximab, 40-50 per cent of patients with low grade lymphomas and 60 - 70 % of patients with aggressive lymphomas fail to respond to single-agent rituximab, only 5 - 10% of patients attain complete remissions, and the median response duration is only approximately one year. Consequently, many investigators have begun exploring methods of enhancing the potency of antibodies, including combining them with chemotherapy and conjugating them to toxins or radionuclides. Czuczman conducted a pilot trial administering CHOP chemotherapy (cyclophosphamide, doxorubicin, vincristine, and prednisone) with rituximab to forty patients with indolent lymphomas. *(20)* Objective remissions were observed in 38 patients (95%), including 22 CRs (55%), with a median time-to-treatment failure of more than 29 months.

Clinical Studies with Iodine-131 Anti-B1 Antibody

Phase I/II Study at University of Michigan (RIT-I-000)

An alternative approach to enhance the efficacy of anti-CD20 antibodies involves conjugating them to cytotoxic radionuclides which can then be selectively targeted to B lymphoid tumors. Study RIT-I-000 was the initial Phase I/II, open-label study of non-myeloablative doses of Iodine-131 anti-B1 antibody for the treatment of patients with B-cell NHL of all histologic types. *(21 - 23)* Fifty-nine patients were enrolled. Twenty-eight patients had low-grade NHL, 14 had transformed low-grade NHL, 15 had intermediate-grade NHL, and 2 had high-grade NHL. The median time from diagnosis was 45 months, the median number of prior therapies was 4, 88% had Stage III or IV disease, 36% had bulky disease, 51% had an elevated LDH, and 24% had failed bone marrow transplant (BMT). Patients received 1 - 3 dosimetric doses followed by a therapeutic dose. The dosimetric dose(s) involved the IV administration of 5 mCi of Iodine-131 anti-B1 antibody to determine the rate of whole body clearance so that a whole body radiation dose (cGy) could be calculated. Each dosimetric dose was preceded by 0, 95, or 475 mg of unlabeled antibody. Therapeutic dose-escalation was initiated at 25 cGy and adjusted in 10 cGy increments until the maximum tolerated dose (MTD) was reached. Fifty-three of the 59 patients received a therapeutic dose. The MTD was 75 cGy for patients who had not undergone BMT. Based on all data through 7/31/97, a response was observed in 42/59 (71%) patients and a complete response (CR) was observed in 20/59 (34%) patients. The median duration of response was 271 days (95% confidence interval: 140 - 394 days) and median duration of CR was 566 days (95% confidence interval: 385 days to upper limit not reached). Nine of the 59 patients remain in CR. Responses were observed in 50% of post-BMT patients and 52% of bulky disease patients. A response was observed in 24/28 (86%) patients and a CR was observed in 13/28 (46%) patients with low-grade NHL. A response was observed in 11/14 (79%) patients and a CR was observed in 7/14 (50%) patients with transformed low-grade NHL. Dose-dependent pharmacokinetics were observed. The mean tumor dose was 14.5 times the whole body dose. The dose-limiting toxicity was hematologic; 3 patients developed a platelet count < 10,000 cells/mm^3 and 2 patients had an ANC < 100 cells/mm^3. The most prevalent non-hematologic toxicities were transient, mild to moderate fever, nausea, asthenia, and chills. Nine of 59 (15%) patients developed human anti-murine antibodies (HAMA).

CLOSED EFFECTIVE 09/15/2008

SWOG Authorized Document

Iodine-131 Anti-B1 Antibody Phase II Dosimetry Validation Study (RIT-II-001)

Study RIT-II-001 was a Phase II, multicenter, open-label study of non-myeloablative doses of Iodine-131 anti-B1 antibody for the treatment of patients with low-grade B-cell lymphomas and transformed low-grade lymphomas. Thirty-seven patients had low-grade NHL and 10 had transformed low-grade NHL. The median time from diagnosis was 41 months, the median number of prior therapies was 4, 91% had Stage III or IV disease, 44% had bulky disease, 44% had an elevated LDH. Patients received 1 dosimetric dose followed by a therapeutic dose. The dosimetric dose involved the IV administration of 450 mg of unlabeled antibody and 35 mg (5 mCi) of Iodine-131 anti-B1 antibody to determine the rate of whole body clearance so that a

CLOSED EFFECTIVE 09/15/2008

SWOG Authorized Document

whole body radiation dose (cGy) could be calculated. The therapeutic dose involved IV administration of 450 mg of unlabeled antibody and 35 mg of Iodine-131 anti-B1 antibody with radioactive Iodine-131 titrated to deliver 75 cGy. Forty-five of the 47 patients received a single dosimetric and therapeutic dose of Iodine-131 anti-B1 antibody as described in the protocol. A response was observed in 27/47 (57%) patients and a complete response (CR) was observed in 14/47 (30%) patients. The median duration of response was 248 days (95% confidence interval: 136 days to upper limit not reached) and median duration of CR was not reached (95% confidence interval: 414 days to upper limit not reached). Ten of the 47 patients remain in CR with ongoing responses ranging from 245-606 days. All clinical sites had at least 1 patient who achieved a CR. Responses were observed in 59% of bulky disease patients. A response was observed in 21/37 (57%) patients and a CR was observed in 9/37 (24%) patients with low-grade NHL. A response was observed in 6/10 (60%) patients and a CR was observed in 5/10 (50%) patients with transformed low-grade NHL. The mean tumor dose was 10.6 times the whole body dose. The dose-limiting toxicity was hematologic; 5 patients developed a platelet count < 10,000 cells/mm^3 and, mild to moderate asthenia, nausea and fever. Only 1 of 46 (2%) patients developed HAMA following treatment as assessed by centralized validated HAMA assay.

<u>HAMA with Tositumomab/Iodine I-131 Tositumomab (BEXXAR®)</u>

One of the objectives of this protocol is to monitor the incidence and prevalence of HAMA since their presence may affect the accuracy of the results of *i*n vitro and in vivo diagnostic tests and may affect the toxicity profile and efficacy of therapeutic agents that rely on murine antibody technology. *(24)* Patients with pre-existing evidence of HAMA positivity may be at increased risk of anaphylaxis or other serious allergic hypersensitivity reactions during administration of BEXXAR®. *(25)* Cumulative safety data for the BEXXAR® therapeutic regimen are derived primarily from 230 patients with low-grade or transformed low-grade NHL who were treated with BEXXAR® in one of 5 clinical studies using the recommended dose and schedule. Patients had a median follow-up of 39 months and 79% of the patients were followed at least 12 months for survival and selected adverse events. *(24)* Safety data on serious or delayed adverse events (e.g., HAMA) from the expanded access program, including 765 patients, were used to supplement the characterization of these events (for a total of 995 patients). *(24)* In patients with relapsed or refractory low-grade or transformed low-grade NHL treated with BEXXAR, 10% to 11% converted to HAMA positivity. *(24)* The median time to a positive HAMA was 5 to 6 months. Data are presented in the Table below.

Incidence and Cumulative Incidence of HAMA *(24)*

Study Population	Evaluable patients (n)	Median follow-up	Incidence of HAMA positivity	Median time to positivity	6-, 12-, and 18-month cumulative incidence
**5 clinical trials (N = 230)**	**219**	**6 months**	**23 (11%)**	**6 months**	**6%, 17%, and 21%**
**EAP (N = 765)**	**569**	**7 months**	**57 (10%)**	**5 months**	**7%, 12%, and 13%**
**N = Number of patients; n = number of evaluable patients, i.e., patients who are seronegative prior to BEXXAR and had post-BEXXAR HAMA level(s) available**					

Limited data are available on the efficacy and safety of BEXXAR® in patients who developed HAMA after BEXXAR® therapeutic regimen. Therefore, no conclusions can be drawn at this time. The possibility exists that the presence of HAMA may affect the efficacy and toxicity profiles of therapeutic agents that are produced by murine antibody technology. *(24)*

The observed incidence of antibody positivity in an assay is highly dependent on the sensitivity and specificity of the assay and may be influenced by several factors such as sample handling, concomitant medications, and underlying disease. Therefore, comparisons of the incidence of HAMA/HACA of one product with that of the other products may be misleading *(24, 26)*.

CLOSED EFFECTIVE 09/15/2008

SWOG Authorized Document

High Dose Therapy with I-131 Tositumomab

Other groups have also investigated the therapeutic efficacy of radiolabeled anti-CD20 antibodies. Press and colleagues studied myeloablative doses of the same radioiodinated antibody (anti-B1, Coulter Pharmaceuticals) with autologous stem cell support in Phase I and I I trials in Seattle. *(27 - 28)* Twenty-nine patients with multiple relapsed B cell lymphomas were treated with single agent I-131-anti-CD20 (B1) antibody (2.5 mg/kg, 280 to 785 mCi) followed by autologous hematopoeitic stem cell rescue between 2/90 and 7/94. Objective responses occurred in 86% of patients, including 79% complete responses. Early toxicities included Grade 4 myelosuppression in all patients, Grade 2 - 3 nausea in 8 of 29, and fatal sepsis in one. Reversible cardiopulmonary failure was the dose-limiting non-hematopoietic toxicity (2 pt.), occurring at an estimated absorbed lung dose of 27 Gy. Overall survival and progression-free survival were 83% and 52%, respectively, after a median follow-up of 38 months. None of the surviving patients had long term objective impairment of performance status or cardiopulmonary function, though one patient with a pre-existing anthracycline-induced cardiomyopathy remained on digoxin (with an ejection fraction of 69%) four years after radioimmunotherapy. No serious delayed cardiopulmonary complications occurred. Late toxicities were minimal, except for elevation of the thyroid stimulating hormone level in 59% of the subjects. Two patients who had been heavily pretreated with alkylating agents and external beam irradiation developed acute leukemia eight years after radioimmunotherapy. Two patients developed secondary solid neoplasms 3 years after treatment (1 noninvasive transitional cell carcinoma of the bladder; 1 metastatic colon cancer).

Studies With Other Radioimmunoconjugates

A similar series of trials have been conducted using the Y-90-labeled 2B8 antibody (Ibritumomab Tiuxetan, Zevalin®). Knox administered Yttrium-90-labeled anti-CD20 antibodies to 18 patients with relapsed B cell lymphomas (4 treated with Y-90-tositumomab antibody and 14 with ibritumomab tiuxetan) in escalating single doses of 13.5 to 50 mCi. *(29)* Six complete remissions and 7 partial responses were observed (overall response rate, 72%), with a median response duration of six months. Four patients developed human anti-mouse antibodies (HAMA). Grade 4 myelosuppression was seen at doses above 50 mCi of Y-90, but no other serious toxicities were observed. Witzig performed a subsequent multi-center Phase I/II trial treating 51 patients with Ibritumomab Tiuxetan (Y2B8, Zevalin®). *(30)* In the phase I portion of the trial, patients received either 100 or 250 mg/m^2 of unlabeled rituximab followed by 0.2, 0.3, or 0.4 mCi/kg of Y2B8. The optimal dose of unlabeled rituximab was determined to be 250 mg/m^2 and 0.4 mCi/kg was the maximally tolerated dose of Yttrium-90. In an intent-to-treat analysis, the overall response rate was 67%, including 26% CRs and 41% PRs. Eighty-two per cent of the low grade lymphoma patients responded (26% CRs, 56% PRs), and 43% of 14 aggressive lymphomas responded. Hematologic toxicity was dose-limiting. Only one patient developed an immune response.

Witzig has presented an interim analysis of a randomized clinical trial comparing the remission rates of patients treated with rituximab (375 mg/m^2 weekly for 4 weeks) or Y-90-ibritumomab tiuxetan (0.4 mCi/kg). *(31)* One hundred and forty three patients were entered on the randomized trial, but only 90 patients were evaluable at the time of the interim analysis. The overall response rate was 80% for the Y-90-labeled antibody as compared with 44% for the chimeric unlabeled rituximab antibody ($p<0.001$). The complete response rate was also higher in the group receiving radioimmunotherapy compared with the rituximab group (21% vs 7%, respectively). Both regimens were well tolerated, but significantly more myelosuppression occurred in the radiolabeled antibody group compared with the unlabeled chimeric antibody, as expected. This is the first randomized, controlled trial demonstrating unequivocally that a radiolabeled antibody produces higher overall and complete response rates than the corresponding unlabeled antibody. Whether the higher response rates with radiolabeled antibodies will translate into longer event-free or overall survival will require longer follow-up.

Despite the impressive efficacy of single agent radiolabeled anti-CD20 antibodies for patients with relapsed lymphomas, several problems remain. Despite high response rates to non-myeloablative doses of Iodine-131 anti-B1 antibody it is unlikely that many will be permanently cured. High doses of I-131-labeled anti-CD20 induce complete remissions in most patients, and approximately half will be alive and disease-free after 5 years, but the treatment is toxic and

CLOSED EFFECTIVE 09/15/2008

SWOG Authorized Document

expensive and requires a protracted hospitalization. The current protocol will study the tolerability, feasibility and efficacy of combining standard CHOP chemotherapy with non-myeloablative doses of Iodine-131 anti-B1 antibody or with rituximab, in hopes of developing a regimen which will induce durable remissions or cures in patients with follicular lymphomas without serious toxicities.

Rationale for Current Protocol

The Southwest Oncology Group and other cooperative groups have recently focussed their activities on studying the possible utility of chemotherapy followed by adjuvant immunotherapy for changing the natural history of follicular lymphoma. **SWOG-8809** tested the use of interferon as a maintenance agent compared to no maintenance therapy; unfortunately, interferon was not found to be beneficial in this study and was poorly tolerated by patients. *(23)* **S9800** was a Phase II pilot study studying the feasibility and toxicity of administering CHOP followed by a chimeric anti CD20 antibody, rituximab (Rituxan®, IDEC Pharmaceuticals). Similarly, **S9911** was a Phase II pilot study studying the feasibility and toxicity CHOP followed by an Iodine-131-labelled anti-CD20 (anti-B1) antibody (tositumomab + I-131 tositumomab). Both of these pilot studies have documented the feasibility of combining CHOP with anti-CD20 targeted therapy and documented high response rates and tolerable toxicities. The present study is intended to be a definitive Phase III, randomized comparison of CHOP chemotherapy alone with CHOP +rituximab and CHOP + I-131-tositumomab to assess whether adjuvant anti-CD20 targeted immunotherapy affects the overall and progression-free survival of patients with newly diagnosed follicular lymphomas, compared with chemotherapy alone. **(THE CHOP ONLY ARM OF THIS STUDY WAS PERMANENTLY CLOSED, EFFECTIVE 12/15/02.)**

The choice of a chemotherapy regimen for such a study is controversial, since no regimen has demonstrated clear superiority over any other (Miller, 1997). Although a strong case can be made for using chlorambucil or CVP as the "standard" regimen for indolent NHL, CHOP was chosen for this trial for several compelling reasons. First, the use of a moderately aggressive regimen such as CHOP is considered most likely to produce a state of minimal tumor burden, which is considered the ideal setting for immunotherapies. (Antibody molecules are large proteins which penetrate large tumor masses poorly. Furthermore, theoretical models suggest that the maximal crossfire from radiolabeled antibodies occurs when cell clusters are less than 1 mm in diameter). *(32)* Second, other adjuvant immunotherapies such as interferon-alpha have been shown to produce an advantage in terms of progression-free or overall survival only in studies using aggressive chemotherapy regimens such as CHOP and not when combined with regimens such as chlorambucil or CVP. *(33, 34)* Finally, a pilot trial by Myron Czuczman combining CHOP chemotherapy with rituximab has demonstrated a promising 95% overall response rate and a 75% two year progression free survival in newly diagnosed patients. *(20)* In the current Phase III trial we plan to administer concurrent CHOP + rituximab on one of the treatment arms since preliminary data by Czuczman and by Demidem suggest that this schedule may provide synergistic effects. *(20, 35)* Concurrent administration of CHOP and I-131-tositumomab is not possible because both produce myelosuppression, and therefore I-131-tositumomab will be given sequentially approximately four weeks after the last cycle of CHOP chemotherapy, as we have done in our pilot study, **S9911**.

CLOSED EFFECTIVE 09/15/2008

SWOG Authorized Document

Patients known to be HIV-positive are not eligible for this study because the severely depressed immune system and poor bone marrow reserve found in HIV infected patients, as well as the possibility of premature death, would compromise study objectives. Pregnant or nursing women are also not eligible for this study, due to the possibility that congenital abnormalities or harm to nursing infants may be caused by this treatment regimen.
Inclusion of Women and Minorities:

This study is open to women and minorities. Based on recent registrations to studies involving non-Hodgkin's disease (follicular type) studies it is anticipated that accrual in the race and sex subgroups will be as shown in the table below.

	White, not of Hispanic Origin	Hispanic	Black, not of Hispanic Origin	Native Hawaiian or other Pacific Islander	Asian	American Indian or Alaskan Native
Female	283	5	14	2	4	0
Male	433	8	20	1	4	1

We are aware of no evidence indicating an interactive effect of treatment by sex or race in this group of patients.

There are no plans to incorporate sex or race specific accrual goals into this study. However, there will be an exploratory analysis of interaction between treatment effect and sex or race on outcome

3.0 DRUG INFORMATION

3.1 Cyclophosphamide (Cytoxan®) (NSC-26271)

a. DESCRIPTION

2-[bis(2-chloroethyl)amino]tetrahydro-2H-1,3,2-oxazaphosphorine 2-oxidemonohydrate. Cyclophosphamide is biotransformed principally in the liver to active alkylating metabolites which cross-link to tumor cell DNA.

b. TOXICOLOGY

Human Toxicology: Toxicity from cyclophosphamide includes bone marrow suppression which usually occurs 10 to 12 days after administration, nausea, vomiting, anorexia, abdominal discomfort, diarrhea, stomatitis, hemorrhagic colitis, jaundice, reversible alopecia, hemorrhagic cystitis which can frequently be prevented with increased hydration, hematuria, ureteritis, tubular necrosis, fibrosis of the bladder, cardiac toxicity which may potentiate doxorubicin-induced cardiotoxicity, rare anaphylactic reaction, skin rash, hyperpigmentation of the skin and nails, interstitial pulmonary fibrosis, and cross sensitivity with other aklylating agents. Treatment with cyclophosphamide may cause significant suppression of the immune system.

Second malignancies, most frequently of the urinary bladder and hematologic systems, have been reported when cyclophosphamide is used alone or with other anti-neoplastic drugs. It may occur several years after treatment has been discontinued. It interferes with oogenesis and spermatogenesis and may cause sterility in both sexes which is dose and duration related. It has been found to be teratogenic, and women of childbearing potential should be advised to avoid becoming pregnant. Increased myelosuppression may be seen with chronic administration of high doses of phenobarbital. Cyclophosphamide inhibits cholinesterase activity and potentiates effect of succinylcholine chloride. If

CLOSED EFFECTIVE 09/15/2008

SWOG Authorized Document

patient requires general anesthesia within 10 days after cyclophosphamide administration, the anesthesiologist should be alerted. Adrenal insufficiency may be worsened with cyclophosphamide. Cyclophosphamide is excreted in breast milk, and it is advised that mothers discontinue nursing during cyclophosphamide administration. The occurrence of acute leukemia has been reported rarely in patients treated with anthracycline/alkylator combination chemotherapy.

c. PHARMACOLOGY

Kinetics: Cyclophosphamide is activated principally in the liver by a mixed function microsomal oxidase system. PO administration is well absorbed, with bioavailability greater than 75%. Five to twenty-five percent of unchanged drug is excreted in the urine. Several active and inactive metabolites have been identified with variable plasma protein binding. There appears to be no evidence of clinical toxicity in patients with renal failure, although elevated levels of metabolites have been observed.

Formulation: Cyclophosphamide is supplied in 100 mg, 200 mg, 500 mg, 1 gram and 2 gram vials as a white powder. The drug should be reconstituted with Sterile Water for Injection, USP, and may be diluted in either normal saline or D5W.

Storage and Stability: Although the reconstituted cyclophosphamide is stable for six days under refrigeration, it contains no preservatives and therefore should be used within 6 hours.

Administration: Cyclophosphamide should be diluted in about 150 cc of normal saline or D5W and infused IV. An added dose of IV fluids may help prevent bladder toxicity. The tablet form of the drug may also be administered PO.

Supplier: Cyclophosphamide is commercially available and should be purchased by a third party. This drug will not be supplied by the NCI.

3.2 Doxorubicin (NSC-123127)

a. DESCRIPTION

Mechanism of Action: Doxorubicin is a cytotoxic anthracycline antibiotic different from daunorubicin by the presence of a hydroxyl group in the C-14 position. Doxorubicin is produced by fermentation from S. *Peucetius* var. *caesius*. Its mechanism of action is thought to be the binding of nucleic acids, preventing DNA and possibly RNA synthesis.

b. TOXICOLOGY

Human Toxicology: Studies with doxorubicin have shown that the major toxic effects of this drug are alopecia, which is often total but always reversible; nausea and vomiting, which develops shortly after drug administration, occasionally persisting for 2 - 3 days; fever on the day of administration; and phlebitis at the site of the drug's injection. Extravasation of the drug will lead to soft tissue necrosis. Phlebosclerosis, cellulitis, vesication and erythematous streaking have also been seen. Mucositis may be seen 5 - 10 days after administration. Ulceration and necrosis of the colon, particularly the cecum, with bleeding and severe infection have been reported with concomitant administration of cytarabine. Anorexia and diarrhea have also been observed. Hyperpigmentation of nailbeds and dermal creases, onycholysis and recall of skin reaction from prior radiotherapy may occur. Cardiac toxicity manifested as acute left ventricular failure, congestive heart failure, arrhythmia or severe cardiomyopathy has been reported, but appears to occur predominantly in patients who receive total doses in excess of 550 mg/M^2. Myelosuppression,

CLOSED EFFECTIVE 09/15/2008

SWOG Authorized Document

predominantly neutropenia, is common with nadir occurring approximately two weeks after a single injection; lesser degrees of anemia and thrombocytopenia have been reported. Rapid recovery of the blood counts approximately two and a half weeks after a single injection generally permits an every three week schedule. Patients with obstructive liver disease have more severe myelosuppression due to impaired drug excretion. Thus, patients with hepatic dysfunction may need to have reduced dosage or to be excluded from therapy. Renal excretion of doxorubicin is minimal, but enough to color the urine red; thus impaired renal function does not appear to increase the toxicity of doxorubicin. Other side effects include fever, chills, facial flushing, itching, anaphylaxis, conjunctivitis and lacrimation. The occurrence of acute leukemia has been reported rarely in patients treated with anthracycline/alkylator combination chemotherapy.

c. PHARMACOLOGY

Kinetics: Intravenous administration is followed by a rapid plasma clearance with significant tissue binding. Urinary excretion is negligible; biliary excretion accounts for 40 to 50% of the administered dose being recovered in the bile or the feces in 7 days. The drug does not cross the blood-brain barrier.

Formulation: Doxorubicin is supplied in 10, 20 and 50 mg single-use vials, and 150 mg multidose vials as a red-orange, lyophilized powder which has a storage stability of at least two years - see expiration date on vial. Doxorubicin should be reconstituted with 5, 10, 25 and 75 ml respectively, of Sodium Chloride Injection, USP (0.9%) to give a final concentration of 2 mg/ml.

Storage and Stability: The reconstituted doxorubicin is stable for 24 hours at room temperature and 48 hours under refrigeration (2° - 8°C). It should be protected from exposure to sunlight. Discard any unused solution from the vials. Bacteriostatic diluents with preservatives are NOT recommended as they might possibly worsen the reaction to extravasated drug.

Administration: Doxorubicin may be further diluted in 5% dextrose or sodium chloride injection and should be administered slowly into tubing of a freely flowing intravenous infusion with great care taken to avoid extravasation.

Supplier: This drug is commercially available for purchase by the third party. This drug will not be supplied by the NCI.

3.3 Prednisone (NSC-10023)

a. DESCRIPTION

Prednisone is a glucocorticoid rapidly absorbed from the GI tract.

b. TOXICOLOGY

Human Toxicology: Possible adverse effects associated with the use of prednisone are: fluid and electrolyte disturbances, congestive heart failure in susceptible persons, hypertension, euphoria, personality changes, insomnia, mood swings, depression, exacerbation of infection (e.g., tuberculosis), exacerbation or symptoms of diabetes, psychosis, muscle weakness, osteoporosis, vertebral compression fractures, pancreatitis, esophagitis, peptic ulcer, dermatologic disturbances, convulsions, vertigo and headache, endocrine abnormalities, ophthalmic changes, and metabolic changes. Some patients have experienced itching and other allergic, anaphylactic or other hypersensitivity reactions. Withdrawal from prolonged therapy may result in symptoms including fever, myalgia and arthralgia. Phenytoin phenobarbitol and ephedrine enhance metabolic clearance of corticosteroids.

CLOSED EFFECTIVE 09/15/2008

SWOG Authorized Document

Corticosteroids should be used cautiously in patients with hypothyroidism, cirrhosis, ocular herpes simplex, existing emotional instability or psychotic tendencies, nonspecific ulcerative colitis, diverticulitis, fresh intestinal anastomoses, peptic ulcer, renal insufficiency, hypertension, osteoporosis and myasthenia gravis. Immunization procedures (especially smallpox vaccination) should not be undertaken in patients on corticosteroids.

c. PHARMACOLOGY

Kinetics: Natural and synthetic glucocorticoids are readily and completely absorbed from the GI tract. Prednisone is very slightly soluble in water. Glucocorticoids have salt-retaining properties. The anti-inflammatory property of this drug is its ability to modify the body's immune system. On the other hand, glucocorticoids suppress the body's response to viral as well as bacterial infections. Equivalent doses are as follows:

Dexamethasone	Methyl-prednisolone and Triamcinolone	Prednisolone and Prednisone	Hydrocortisone	Cortisone
0.75 mg	4 mg	5 mg	20 mg	25 mg

Formulation: Prednisone is available in 2.5 mg, 5 mg, 10 mg, 20 mg and 50 mg tablets.

Storage and Stability: Prednisone should be stored at room temperature.

Administration: Prednisone is administered orally.

Supplier: Prednisone is commercially available and should be purchased by third party. Prednisone will not be supplied by the NCI.

3.4 Vincristine (Oncovin) (NSC-67574)

a. DESCRIPTION

Chemistry: Vincristine is one of the so-called vinca-alkaloids and is extracted from the plant cantharanthus roseus (vinca rosea).

Biochemistry: This drug appears to produce the arrest of mitosis in animal cells by interfering with microtubule function.

b. TOXICOLOGY

Human Toxicology: The primary toxic effects of vincristine are neurological with paresthesia, weakness, muscle wasting, motor difficulties including difficulty walking and slapping gait, loss of deep tendon reflexes, sensory loss, neuritic pain, paralytic ileus, bladder atony, and constipation. Rarely, it produces myelosuppression. Other side effects may include alopecia, allergic reactions, (including rare anaphylaxis, rash and edema), jaw pain, hypertension, hypotension, nausea, vomiting, diarrhea, fever, headache, oral ulceration, optic atrophy with blindness, ptosis, diplopia and photophobia. The occurrence of acute leukemia has been reported rarely in patients treated with anthracycline/alkylator combination chemotherapy.

c. PHARMACOLOGY

Kinetics: After IV administration, a triphasic serum decay pattern follows with half-lives of 5 minutes, 2-3 hours, and 85 hours. The range of terminal half-life is 19-155 hours. Excretion is 80% in the feces and 10-20 % in the urine.

CLOSED EFFECTIVE 09/15/2008

SWOG Authorized Document

The liver is the major excretory organ in humans and animals, and biliary obstruction causes increased toxicity in man.

Formulation: 1 mg/1 ml, 2 mg/2 ml, and 5 mg/5 ml vials containing solution. It is also available in 1 mg/ml and 2 mg/2 ml disposable syringes.

Storage and Stability: It should be stored under refrigeration. Vincristine is available with and without preservatives so the time-frame for use once the vial has been entered varies. The intact vials have a labelled expiration date. Protect from light. Parenteral drug products should be inspected visually for particulate matter and discoloration prior to administration.

Administration: Vincristine should be administered intravenously through a freely-running IV. If it extravasates, it produces a severe local reaction with skin slough. **FATAL IF GIVEN INTRATHECALLY, FOR INTRAVENOUS USE ONLY.**

Supplier: Vincristine is commercially available, and should be purchased through a third party. This drug will NOT be supplied by the NCI.

3.5 Iodine-131-labeled Murine Monoclonal Anti-B1Antibody (tositumomab)(BB-IND-8283)

a. DESCRIPTION

Anti-B1 Antibody/Iodine-131 Anti-B1 Antibody

Anti-B1 Antibody is an IgG2a Kappa (murine) monoclonal antibody that binds to the CD20 antigen on the surface of the normal and malignant human B cells to induce apoptosis and mediate antibody-dependent cellular cytotoxicity. Iodine-131 Anti-B1 Antibody is the radionuclide-labeled monoclonal antibody that can recognize tumor-associated antigens to selectively target radioactivity to tumor cells. By using isotopes emitting beta particles to label this antibody, the radiation emitted from the radiolabeled antibody bound to a tumor cell also kills neighboring cells because the path length of beta particles can extend over several cell diameters. This crossfire of beta particles can destroy antigen-positive and -negative tumor cells, as well as untargeted antigen-positive tumor cells within a tumor.

Iodine-131 Anti-B1 Antibody, used in conjunction with Anti-B1 Antibody, is a radioimmunotherapeutic agent being studied for the treatment of non-Hodgkin's lymphoma and other CD20-expressing B-cell malignancies.

b. TOXICOLOGY

Human Toxicology: Unlabeled anti-B1 antibody and Iodine-131 anti-B1 antibody infusions have been administered to over 250 patients in non-myeloablative doses for the treatment of non Hodgkin's lymphoma. The infusions are accompanied by few or no adverse experiences in most patients. The most frequent non-hematologic adverse experience reported was a transient mild to moderate flu-like syndrome consisting of fever (39%), and chills (19%). Other adverse experiences commonly reported include nausea (39%), asthenia (36%), headache (23%), rash (18%), anorexia (16%), infection (16%), pain (16%), myalgia (16%), arthralgia (14%), pruritus (14%), abdominal pain (13%), vomiting (13%), pharyngitis (11%), diarrhea (10%), and increased cough (10%). The concomitant administration of SSKI or other oral iodine product may contribute to the nausea and other gastrointestinal adverse experiences. Only 3% and 4% of patients experienced an adverse experience which required an adjustment to the rate of infusion during the administration of the dosimetric and therapeutic doses, respectively.

CLOSED EFFECTIVE 09/15/2008

SWOG Authorized Document

Bone marrow suppression is the dose-limiting toxicity and a total body dose of 75 cGy was determined to be the maximum tolerated dose for a non-myeloablative regimen in patients previously treated with chemotherapy. An absolute neutrophil count (ANC) of < 100 cells/mm^3 has occurred in 3% of patients, and an ANC of < 1,000 cells/mm^3 has occurred in 47% of patients. A platelet count of < 10,000 cells/mm^3 has occurred in 5% of patients, and a platelet count of < 50,000 cells/mm^3 has occurred in 37% of patients. A hemoglobin of < 6.5 g/dL has occurred in 4% of patients, and a hemoglobin of < 8.0 g/dL has occurred in 12% of patients. A white blood cell count of < 2,000 cells/mm^3 has occurred in 41% of patients. The median nadirs were 62,000 cells/mm^3 for platelet count, 1,000 cells/mm^3 for ANC, and 11.1 gm/dL for hemoglobin. Blood count nadirs (which occur approximately 4 to 6 weeks after therapy) were higher and the time to recovery shorter in patients who were less heavily pretreated. The need for hematologic supportive care, which included transfusions and colony stimulating factors and were used at the discretion of the investigators, ranged from 0% in previously untreated patients to 24% in patients having received 4 or more prior therapies and was 18% overall. The frequency of HAMA-positivity was related to the extent of prior therapy; 38% in previously untreated patients versus 4% in previously treated patients (≥ 1 prior therapies). Thyroid function has been followed long-term and elevated thyroid stimulating hormone has been noted in 5 out of 106 (4%) of patients. Four of these patients have been started on oral thyroid supplementation, although clinical hypothyroidism has not been diagnosed. No significant changes in serum immunoglobulins have occurred post-treatment. Four patients developed a myelodysplastic syndrome or acute myelocytic leukemia in long-term follow-up.

The safety of Iodine-131 anti-B1 antibody in pregnant or nursing women has not been proven. Women of child-bearing potential should only be treated after negative pregnancy tests have been confirmed.

c. PHARMACOLOGY

Pharmacokinetics: After IV administration, a two compartmental model best fit the data with a median terminal half-life of 70.4 hours. The mean clearance was 97.9 ± 109.2 mL/hr (mean ± standard deviation). Dose-dependent pharmacokinetics were observed with a larger area under the curve (AUC), slower clearance, longer terminal half-life, and smaller volume of distribution at steady state observed with increasing predose levels of anti-B1 antibody. The route of excretion was renal with 65 ± 13% of the injected dose recovered in the urine over the initial 5 day time period. The mean total body effective half-lives were 65.2 ± 12.5 and 65.8 ± 12.9 hours by sodium iodide probe counts and gamma camera counts, respectively. Organ doses were modest and below normal tissue tolerances. The mean splenic dose was 399 ± 215 cGy/75 cGy total body dose (TBD). The kidney received 630 ± 201 cGy/75 cGy TBD. The cGy doses to other normal tissues from a 75 cGy whole body dose were quite modest, with the liver and the lungs receiving an average dose of 256 ± 80 cGy and 182 ± 58 cGy, respectively. The mean bladder wall dose was 202 ± 49 cGy/75 cGy TBD, the mean bone marrow dose was 103 ± 15 cGy/75 cGy TBD, and the mean blood dose was 369 ± 97 cGy/75 cGy TBD.

Formulation: Anti-B1 Antibody and Iodine-131 Anti-B1 Antibody:

Anti-B1 Antibody is a murine anti-human B-cell monoclonal antibody of the IgG$_2$a subclass. As formulated, Anti-B1 Antibody is a sterile, clear, colorless liquid supplied in a 3 ml or 20 ml glass vial with a gray silicone-coated butyl rubber stopper and capped with an aluminum crimp seal. Each single-use 3 ml vial contains not less than 2.5 ml of solution; each single-use 20 ml vial contains not less than 16.1 ml of solution. The formulation or each single-use vial is:

CLOSED EFFECTIVE 09/15/2008

SWOG Authorized Document

Protein concentration 14.0 ± 0.7 mg/ml

Potassium phosphate: 10 mM, pH 7.2 ± 0.2

Sodium Chloride: 145 mM

Maltose: 10%

<u>Iodine-131 Anti-B1 Antibody</u>:

The Iodine-131 Anti-B1 antibody is a sterile, colorless liquid in a glass vial with a gray silicone-coated butyl rubber stopper and capped with an aluminum crimp seal. Each vial is intended for single-use only.

The dosimetric vial contains not less than 1.4 ml of solution in a 10 ml vial consisting of:

Protein concentration 1.1 - 2.5 mg/ml

Calibrated activity: 8 - 12 mCi

Povidone: 5.5%

Ascorbic Acid: 0.1%

Potassium phosphate: 12.5 mM, pH 6.8 - 7.2

Sodium Chloride: 0.9%

Maltose: 1 - 2%

OR, the dosimetric vial may contain not less than 20.0 ml of solution in a 30 ml vial consisting of:

Protein concentration 0.10 - 0.25 mg/ml

Calibrated activity: 12 - 18 mCi

Povidone: 5.5%

Ascorbic Acid: 0.1%

Potassium phosphate: 12.5 mM, pH 6.5 - 7.2

Sodium Chloride: 0.9%

Maltose: 1 - 2%

The therapeutic vial contains not less than 20 ml solution in a 30 ml vial consisting of:

Protein concentration: 1.1 - 2.5 mg/mL

Calibrated activity: 112 - 168 mCi

Povidone: 5.5%

Ascorbic Acid: 0.1%

Potassium Phosphate: 12.5 mM, pH 6.5 - 7.5

Sodium Chloride: 0.9%

Maltose: 1 - 2%

CLOSED EFFECTIVE 09/15/2008

SWOG Authorized Document

Storage and Stability:

Anti-B1: Non-radioactive anti-B1 will be shipped overnight as needed to the study site. Anti-B1 must be stored at 2 - 8°C in a secure area until it is needed for use. The vials are single-use as they do not contain preservative.

I-131-Anti-B1: The lead pot containing Iodine-131 anti-B1 must be stored in a freezer until it is thawed for administration to the patient. See the section on Thawing for Administration in the User's Instructions for complete instructions on the procedure for thawing the vials. Allow approximately 20 minutes for thawing of the 10 ml dosimetric vial and approximately 60 minutes for thawing of the 30 ml dosimetric or therapeutic vial. The thawed vial may be refrigerated at 2 - 8°C for up to 6 hours. The product must be administered to the patient within 72 hours of the calibration date and time specified on the product label.

Administration: See section 7.5c for detailed administration instructions for both unlabeled and I-131-labeled anti-B1.

Supplier: GlaxoSmithKline will supply anti-B1 antibody and I-131-anti-B1 antibody free of charge for this study. The latter will be labeled and distributed by MDS Nordion, Inc. of Canada. Any questions regarding Iodine-131 anti-B1 antibody may be directed to the Service Center at (toll free) 877/423-9927.

Drug Ordering: Unlabeled anti-B1 antibody and I-131 anti-B1 antibody (dosimetric and therapeutic) will be ordered by completing one form, the Study Drug Order Form for **S0016** (see Appendix 19.3) and faxing it to GlaxoSmithKline Clinical Research Department (fax number 610/917-6119). The Study Drug Order Form must be faxed to GlaxoSmithKline by Wednesday, 4:00 p.m. Eastern Time (EST) prior to the treatment week. The product will be shipped frozen on dry ice and will be sent to the site using priority overnight delivery. Upon receipt, immediately follow the section on Procedure for Opening in the User's Instructions. For questions related to radiolabeled antibody ordering, call the Service Center at (toll free) 877/423-9927.

NOTE: Institutions must be approved by GlaxoSmithKline and receive an on-site training session prior to administering I-131 labeled anti-B1 antibody. (See Section 15.1 for specific instructions.)

Drug Return: *Unused* I-131 anti-B1 antibody must be decayed at the site according to institutional policy. *Unused* non-radiolabeled anti-B1 antibody must be returned. Contact the Service Center (toll free: 1/877-423-9927) for instructions. Used empty vials (from both radiolabeled and non-radiolabeled antibody) should be disposed of at the site according to institutional policies.

CLOSED EFFECTIVE 09/15/2008

SWOG Authorized Document

3.6 Rituximab Chimeric Monoclonal anti-CD20 Antibody (IDEC-C2B8) (Rituxan®) (NSC-687451)

a. DESCRIPTION

Rituximab is a mouse/human chimeric monoclonal antibody consisting of human IgG1 heavy and kappa light chain constant regions with murine variable regions from the murine IgG1 kappa anti-human CD20 monoclonal antibody rituximab. The rituximab antibody is produced by a Chinese hamster ovary transfectoma.

b. TOXICOLOGY

Human Toxicology: Single doses of up to 500 mg/m^2 and weekly x 4 doses of 375 mg/m^2 have been administered without dose limiting toxicity. Adverse events are most common during the initial antibody infusion and usually consist of grade I or 2 fever (73%), asthenia (16%) chills (38%) nausea (19%), vomiting (11%), rash (14%) and tumor site pain (3%). Grade 1 or 2 hypotension (8%) may be treated with IV fluids. Hematologic toxicity is usually mild and reversible. Transient decreases in the WBC or platelet count have been observed - especially in patients with high levels of circulating tumor cells or bone marrow involvement. Two patients have had late-onset Grade 4 neutropenia at four and ten months that was attributed to an unknown cause, was transient and resolved. Infections (grade 1 and 2) have not been related to dose level. Symptoms are generally associated with the initial antibody infusions and diminish in frequency with each successive infusion.

Severe Infusion and Hypersensitivity Reactions: Rituximab has caused severe infusion reactions. In some cases, these reactions were fatal. An infusion-related symptom complex consisting of fever and chills/rigors has occurred in the majority of patients during the first rituximab infusion. Signs and symptoms of severe infusion reactions may include urticaria, hypotension, angioedema, hypoxia, or bronchospasm. The most severe manifestations and sequelae include pulmonary infiltrates, acute respiratory distress syndrome, myocardial infarction, ventricular fibrillation, cardiogenic shock, and anaphylactic and anaphylactoid events. These reactions generally occurred within 30 minutes to 2 hours of beginning the first infusion, and resolved with slowing or interruption of the rituximab infusion and with supportive care (including, but not limited to IV saline, diphenhydramine, and acetaminophen).

Tumor Lysis Syndrome: Rituximab rapidly decreases benign and malignant CD20 positive cells. Tumor lysis syndrome has been reported to occur within 12 to 24 hours after the first rituximab infusion in patients with high numbers of circulating malignant lymphocytes. Patients with high tumor burden (bulky lesions) may also be at risk. Patients at risk for developing tumor lysis syndrome should be followed closely and appropriate laboratory monitoring performed.

Hepatitis B Reactivation with Related Fulminant Hepatitis and Other Viral Infections: Hepatitis B virus (HBV) reactivation with fulminant hepatitis, hepatic failure, and death has been reported in some patients with hematologic malignancies treated with rituximab. The majority of patients received rituximab in combination with chemotherapy. The median tme to the diagnosis of hepatitis was approximately 4 months after the initiation of rituximab and approximately one month after the last dose.

CLOSED EFFECTIVE 09/15/2008

SWOG Authorized Document

Persons at high risk of of HBV infection should be screened before initiation of rituximab. Carriers of hepatitis B should be closely monitored for clinical and laboratory signs of active HBV infection and for signs of hepatitis during and for up to several months following rituximab therapy. In patients who develop viral hepatitis, rituximab and any concomitant chemotherapy should be discontinued and appropriate treatment including antiviral therapy initiated. There are insufficient data regarding the safety of resuming rituximab therapy in patients who develop hepatitis subsequent to HBV reactivation.

The following additional serious viral infections, either new, reactivated or exacerbated, have been identified in clinical studies or postmarketing reports. The majority of patients received rituximab in combination with chemotherapy or as part of a hematopoietic stem cell transplant. These viral infections included JC virus (progressive multifocal leukoencephalopathy [PML]), cytomegalovirus, herpes simplex virus, parvovirus B19, varicella zoster virus, West Nile virus, and hepatitis C. In some cases, the viral infections occurred up to one year following discontinuation of rituximab and have resulted in death.

Severe Mucocutaneous Reactions: Mucocutaneous reactions, some with fatal outcome, have been reported in patients treated with rituximab. These reports included paraneoplastic pemphigus (an uncommon disorder which is a manifestation of the patient's underlying malignancy), Stevens-Johnson syndrome, lichenoid dermatitis, vesiculobullous dermatitis, and toxic epidermal necrolysis. The onset of the reaction in the reported cases has varied from 1-13 weeks following rituximab exposure. Patients experiencing a severe mucocutaneous reaction should not receive any further infusions and seek prompt medical evaluation. Skin biopsy may help to distinguish among different mucocutaneous reactions and guide subsequent treatment. The safety of readministration of rituximab to patients with any of these mucocutaneous reactions has not been determined.

Bowel Obstruction and Perforation: Abdominal pain, bowel obstruction and perforation, in some cases leading to death, were observed in patients receiving rituximab in combination with chemotherapy for DLBCL. In post-marketing reports, which include both patients with low-grade or follicular NHL and DLBCL, the mean time to onset of symptoms was 6 days (range 1-77) in patients with documented gastrointestinal perforation. Complaints of abdominal pain, especially early in the course of treatment, should prompt a thorough diagnostic evaluation and appropriate treatment.

Cardiovascular: The incidence of serious cardiovascular events in the double-blind clinical trial for rheumatoid arthritis (RA) patients was 1.7% and 1.3% in rituximab and placebo groups, respectively. Three cardiovascular deaths occurred during the double-blind period of the RA studies, including all rituximab regimens (3/759 = 0.4%) as compared to none in the placebo group (0/389).

Since patients with RA are at increased risk for cardiovascular events compared to the general population, patients with RA should be monitored throughout the infusion and rituximab should be discontinued in the event of a serious or life-threatening cardiac event.

Rituximab infusions should be discontinued in the event of serious or life-threatening cardiac arrhythmias. Patients who develop clinically significant arrhythmias should undergo cardiac monitoring during and after subsequent infusions of rituximab. Patients with pre-existing cardiac conditions including arrhythmias and angina have had recurrences of these events during rituximab therapy and should be monitored throughout the infusion and immediate post-infusion period.

CLOSED EFFECTIVE 09/15/2008

SWOG Authorized Document

Renal: Rituximab administration has been associated with severe renal toxicity including acute renal failure requiring dialysis and in some cases, has led to a fatal outcome in hematologic malignancy patients. Renal toxicity has occurred in patients with high numbers of circulating malignant cells (> 25,000/mm^3) or high tumor burden who experience tumor lysis syndrome and in patients with NHL adminitered concomitant cisplatin therapy during clinical trials. The combination of cisplatin and rituximab is not an approved treatment regimen. If this combination is used in clinical trials *extreme caution* should be exercised; patients should be monitored closely for signs of renal failure. Discontinuation of rituximab should be considered for those with rising serum creatinine or oliguria.

Immunization: The safety of immunization with live viral vaccines following rituximab therapy has not been studied and vaccination with live virus vaccines is not recommended. The ability to generate a primary or anamnestic humoral response to vaccination is currently being studies. For patients with NHL, the benefits of primary and/or booster vaccinations should be weighed against the risks of delay in initiation of rituximab therapy.

Carcinogenesis, Impairment of Fertility, Pregnancy, and Nursing: No long-term animal studies have been performed to establish the carcinogenic potential of rituximab. Studies also have not been completed to assess mutagenic potential of rituximab, or to determine potential effects on fertility in males or females. Individuals of childbearing potential should use effective contraceptive methods during treatment and for up to 12 months following rituximab therapy.

It is not known whether rituximab is excreted in human milk. Because human IgG is excreted in human milk and the potential for absorption and immunosuppression in the infant is unknown, women should be advised to discontinue nursing until circulating drug levels are no longer detectable.

Comprehensive Adverse Event and Potential Risks (CAEPR) list for Rituximab (NSC-687451)

The Comprehensive Adverse Event and Potential Risks (CAEPR) list provides a single list of reported and/or potential adverse events (AE) associated with an agent using a uniform presentation of events by body system. In addition to the comprehensive list, a subset, the Agent Specific Adverse Event List (ASAEL), appears in a separate column and is identified with ***bold*** and ***italicized*** text. This subset of AEs (ASAEL) contains events that are considered 'expected' for expedited reporting purposes only. Refer to the 'CTEP, NCI Guidelines: Adverse Event Reporting Requirements' http://ctep.cancer.gov/reporting/adeers.html for further clarification. *Frequency is provided based on 356 patients*. Below is the CAEPR for Rituximab.

CLOSED EFFECTIVE 09/15/2008

SWOG Authorized Document

Version 2.0, November 17, 2006[1]

**Adverse Events with Possible Relationship to Rituximab (CTCAE v3.0 Term) [n=356 patients]**			**'Agent Specific Adverse Event List' (ASAEL)**
**Likely (>20%)**	**Less Likely (≤20%)**	**Rare but Serious (<3%)**	
ALLERGY/IMMUNOLOGY			
	Allergic reaction/hypersensitivity (including drug fever)		***Allergic reaction/ hypersensitivity (including drug fever)***
	Allergic rhinitis (including sneezing, nasal stuffiness, postnasal drip)		
	Serum sickness		***Serum sickness***
BLOOD/BONE MARROW			
	Blood/Bone Marrow - Other (Hyperviscosity: Waldenstrom's)		***Blood/Bone Marrow - Other (Hyperviscosity: Waldenstrom's)***
	Hemoglobin		***Hemoglobin***
	Leukocytes (total WBC)		***Leukocytes (total WBC)***
Lymphopenia			***Lymphopenia***
	Neutrophils/granulocytes (ANC/AGC)		***Neutrophils/ granulocytes (ANC/AGC)***
	Platelets		***Platelets***
CARDIAC ARRHYTHMIA			
	Sinus tachycardia		***Sinus tachycardia***
	Supraventricular arrhythmia NOS		***Supraventricular arrhythmia NOS***
	Ventricular fibrillation		
CARDIAC GENERAL			
	Cardiac ischemia/infarction		***Cardiac ischemia/infarction***
	Hypertension		***Hypertension***
	Hypotension		***Hypotension***
CONSTITUTIONAL SYMPTOMS			
Fatigue (asthenia, lethargy, malaise)			***Fatigue (asthenia, lethargy, malaise)***
Fever (in the absence of neutropenia, where neutropenia is defined as ANC <1.0 x 10e9/L)			***Fever (in the absence of neutropenia, where neutropenia is defined as ANC <1.0 x 10e9/L)***
Rigors/chills			***Rigors/chills***
	Sweating (diaphoresis)		***Sweating (diaphoresis)***

CLOSED EFFECTIVE 09/15/2008

SWOG Authorized Document

DERMATOLOGY/SKIN			
	Flushing		***Flushing***
	Pruritus/itching		***Pruritus/itching***
	Rash/desquamation		***Rash/desquamation***
		Rash: erythema - multiforme (e.g., Stevens-Johnson syndrome, toxic epidermal necrolysis)	***Rash: erythema multiforme (e.g., Stevens-Johnson syndrome, toxic epidermal necrolysis)***
	Urticaria (hives, welts, wheals)		***Urticaria (hives, welts, wheals)***
GASTROINTESTINAL			
	Diarrhea		***Diarrhea***
Nausea			***Nausea***
	Obstruction, GI - Select		
	Perforation, GI - Select		
	Vomiting		***Vomiting***
INFECTION			
	Febrile neutropenia (fever of unknown origin without clinically or microbiologically documented infection)(ANC <1.0 x 10e9/L, fever ≥ 38.5 °C)		***Febrile neutropenia (fever of unknown origin without clinically or microbiologically documented infection)(ANC <1.0 x 10e9/L, fever ≥ 38.5 °C)***
	Infection - Other (Infection in HIV Positive Patients)		
	Infection - Other (Activation of Hepatitis B, C, CMV, parvovirus B19, JC virus, varicella zoster, herpes simplex, West Nile virus,)		***Infection - Other (Activation of Hepatitis B, C, CMV, parvovirus B19, JC virus, varicella zoster, herpes simplex, West Nile virus,)***
	Infection with Grade 3 or 4 neutrophils - Select		***Infection with Grade 3 or 4 neutrophils - Select***
	Infection with normal ANC or Grade 1 or 2 neutrophils - Select		***Infection with normal ANC or Grade 1 or 2 neutrophils - Select***
LYMPHATICS			
	Edema: limb		
METABOLIC/LABORATORY			
	Calcium, serum-low (hypocalcemia)		***Calcium, serum-low (hypocalcemia)***
	Glucose, serum-high (hyperglycemia)		
	Potassium, serum-high (hyperkalemia)		
	Uric acid, serum-high (hyperuricemia)		

CLOSED EFFECTIVE 09/[illegible]/2008

SWOG Authorized Document

NEUROLOGY			
	Dizziness		***Dizziness***
	Mood alteration: anxiety		
	Seizure		**Seizure**
PAIN			
	Pain - abdomen NOS		***Pain - abdomen NOS***
	Pain - back		
	Pain - head/headache		***Pain - head/headache***
	Pain - joint		***Pain - joint***
	Pain - muscle		***Pain - muscle***
	Pain - throat/pharynx/larynx		
	Pain - tumor pain		***Pain - tumor pain***
PULMONARY/UPPER RESPIRATORY			
		Adult respiratory distress syndrome (ARDS)	
	Bronchospasm, wheezing		***Bronchospasm, wheezing***
	Cough		***Cough***
	Dyspnea (shortness of breath)		***Dyspnea (shortness of breath)***
	Hypoxia		***Hypoxia***
	Pneumonitis/ pulmonary infiltrates		***Pneumonitis/ pulmonary infiltrates***
RENAL/GENITOURINARY			
	Renal Failure		
SYNDROMES			
		Cytokine release syndrome/ acute infusion reaction	***Cytokine release syndrome/acute infusion reaction***
		Tumor lysis syndrome	***Tumor lysis syndrome***

[1] This table will be updated as the toxicity profile of the agent is revised. Updates will be distributed to all Principal Investigators at the time of revision. The current version can be obtained by contacting ADEERSMD@tech-res.com. Your name, the name of the investigator, the protocol and the agent should be included in the e-mail.

Also reported on rituximab trials but with the relationship to rituximab still undetermined:
ALLERGY/IMMUNOLOGY - vasculitis
BLOOD/BONE MARROW - bone marrow cellularity; hemolysis
CARDIAC ARRHYTHMIA - atrial flutter
CARDIAC GENERAL - cardiac troponin I; cardiac troponin T; left ventricular systolic dysfunction
CONSTITUTIONAL SYMPTOMS - insomnia; weight loss
DERMATOLOGY/SKIN – cyanosis; paraneoplastic pemphigus
GASTROINTESTINAL - anorexia; constipation; heartburn; stomatitis
INFECTION - opportunistic infection

CLOSED EFFECTIVE 09/15/2008
SWOG Authorized Document

METABOLIC/LABORATORY – hypercalcemia; hyperphosphatemia
MUSCULOSKELETAL/SOFT TISSUE – arthritis; fracture
NEUROLOGY - agitation; cranial neuropathy; depression; motor neuropathy; pyramidal tract dysfunction; sensory neuropathy
OCULAR/VISUAL - ocular surface disease; uveitis; watery eye
PAIN - chest/thorax pain; pain NOS
PULMONARY/UPPER RESPIRATORY - bronchiolitis obliterans; pleural effusion; pulmonary edema
VASCULAR - phlebitis

Note: Rituximab in combination with other agents could cause an exacerbation of any adverse event currently known to be caused by the other agent, or the combination may result in events never previously associated with either agent.

c. PHARMACOLOGY

Kinetics: In prior studies patients treated at the 375 mg/m^2 dose levels exhibited detectable antibody concentrations throughout the treatment period. Most patients exhibited increasing pre-infusion antibody concentrations with each subsequent infusion. In nine patients, the $T_{1/2}$ following the first antibody infusion was 59.8 hours (11.1-104.6 hr) with a C_{max} of 271 μg/ml. Following the fourth antibody infusion when circulating B cells had been depleted and antigenic sites coated, the $T_{1/2}$ was 174 hr (26.4-442.3 hr) and C_{max} 496.7 μg/ml.

CLOSED EFFECTIVE 09/15/2008

SWOG Authorized Document

Formulation: Rituximab antibody will be provided in 100 mg (10 mL) and 500 mg (50 ml) pharmaceutical grade vials at a concentration of 10.0 mg of protein per mL (actual concentration should be noted on the product label).

Storage and Stability: Rituximab should be stored at 2 - 8°C. Do not freeze or store at room temperature. The product is a protein- HANDLE GENTLY AND AVOID FOAMING. The avoidance of foaming during product handling, preparation and administration is important, as foaming may lead to the de-naturing of the product proteins.

Administration: The total amount of rituximab needed for a patient's entire six infusions (one course) will be determined AT STUDY ENTRY. A single dose of 375 mg/m^2 will be based upon the patient's actual body surface area calculated during the baseline evaluation. The dose level of rituximab will not be adjusted.

Prepare the rituximab infusion solution as follows:

a. If a delay in administration of the infusion occurs after the product is prepared, the properly identified container may be kept refrigerated at 2 - 8°C for up to six hours.

b. Use sterile, non-pyrogenic, disposable containers, syringes, needles, stopcocks and transfer tubing, etc.

c. Transfer of the rituximab from the glass vial should be made by using a suitable sterile graduated syringe and large gauge needle.

d. Transfer the appropriate amount of rituximab from the graduated syringe, into a partially filled IV pack containing sterile, pyrogen-free 0.9% sodium chloride solution, USP (saline solution). The final concentration of rituximab in saline solution should be a maximum of 1 mg/ml. Mix by inverting the bag gently. DO NOT USE A VACUUM APPARATUS to transfer the product from the syringe to the plastic bag.

e. Place an IV administration set into the outflow port of the bag containing the infusion solution.

f. NOTE: DO NOT USE evacuated glass containers which require vented administration sets because this causes foaming as air bubbles pass through the solution.

The administration of rituximab will be accomplished by slow IV infusion. CAUTION: DO NOT ADMINISTER AS AN INTRAVENOUS PUSH OR BOLUS. IV pumps such as the IMED 960 may be used with the rituximab infusion. DO NOT INFUSE CONCOMITANTLY with another IV solution or IV medications. Prime the line with the rituximab solution such that approximately 30 mL are delivered. This will saturate the filter and tubing.

Supplier: This drug is commercially available for purchase by the third party. This drug will not be supplied by the NCI.

CLOSED EFFECTIVE 09/15/2008

SWOG Authorized Document

4.0 STAGING CRITERIA

4.1 The Ann Arbor staging criteria will be used. Stage is determined based on extent of disease at the time of diagnosis. Bulky disease determination is made after surgical resection, if applicable.

4.2 Ann Arbor Classification (AJCC Manual for Staging of Cancer, 5th ed., 1997)

STAGE II — Involvement of two or more lymph node regions on the same side of the diaphragm (II) or localized involvement of a single associated extralymphatic organ or site and its regional nodes with or without other lymph node regions on the same side of the diaphragm (II_E).

STAGE III — Involvement of lymph node regions on both sides of the diaphragm (III) that may also be accompanied by localized involvement of an extralymphatic organ or site (III_E) by involvement of the spleen (III_S) or both (III_{SE}).

STAGE IV — Disseminated (multifocal) involvement of one or more extralymphatic organs with or without associated lymph node involvement, or isolated extralymphatic organ involvement with distant (non-regional) nodal involvement).

A = Asymptomatic

B = Fever, sweats, weight loss > 10% of body weight

4.3 "Bulky" is defined as a mediastinal mass > 1/3 of the maximum chest diameter (i.e., internal dimension of the thoracic cavity measured at its widest point per radiograph) or any other mass ≥ 10 cm in maximum diameter.

CLOSED EFFECTIVE 09/15/2008

SWOG Authorized Document

5.0 ELIGIBILITY CRITERIA

Each of the criteria in the following section must be met in order for a patient to be considered eligible for registration. Use the spaces provided to confirm a patient's eligibility. For each patient, this section must be photocopied, completed and submitted to the Statistical Center (see Section 14.4e).

SWOG Patient No. ________________________

Patient's Initials (L, F, M) ________________________________

_____ 5.1 **The registering institution must have submitted the S0016 Site Contact Information Form (Appendix 19.4) and a copy of their radioactive materials license to GlaxoSmithKline and been approved by GlaxoSmithKline for this study. (The approval process is required only for the FIRST patient registered to this study by any one institution.) Institutions previously approved for the Southwest Oncology Group study, S9911, need not repeat the approval process; however, they must still submit the completed form and license to GlaxoSmithKline.**

Approved (circle one) YES NO Date of Approval ______________________________

_____ 5.2 All patients must have previously untreated follicular Non-Hodgkin's lymphoma (Grade I, II, or III).

_____ 5.3 Lymphomas must express the CD20 antigen as demonstrated by either flow cytometry or immunoperoxidase staining of paraffin sections using anti-CD20 antibodies. A report providing confirmation of CD20 expression must be submitted per Section 14.4.

_____ 5.4 Patients must have Stage III, Stage IV, or bulky Stage II extent of disease by the Ann Arbor classification (see Section 4.0).

_____ 5.5 All patients must have bidimensionally measurable disease (as defined in Section 10.1a) documented within 28 days prior to registration. Patients with non-measurable disease (as defined in Section 10.1b) in addition to measurable disease must have all non-measurable disease assessed within 42 days prior to registration.

Date measurable disease assessed ________________

Date non-measurable disease assessed ______________

_____ 5.6 **Pathology Review**: Adequate sections from the diagnostic specimen or core needle biopsies which are large enough to show the architecture (bone marrow biopsies and needle aspirates are insufficient) must be available for submission as outlined in Section 12.0.

Patients are also eligible to register to and submit serum for **SWOG-8947** and register to and submit tissue for **SWOG-8819**.

_____ 5.7 Patients must have a bilateral or unilateral bone marrow aspirate and biopsy performed within 42 days prior to registration.

Date of bone marrow biopsy/aspirate _____________ Positive/Negative (circle one, prior to data submission as outlined in Section 14.4)

_____ 5.8 **Pretreatment specimens of heparinized marrow (2 - 3cc) for t(14;18)/bcl2 assessment must be submitted to Dr. Rita Braziel per Section 15.2**

Date specimen submitted _____________________

CLOSED EFFECTIVE 09/15/2008

SWOG Authorized Document

SWOG Patient No. ______________________

Patient's Initials (L,F,M) ______________________

____ 5.9 Patients must agree to the serum sample submission schedule for HAMA testing as outlined in Section 15.5.

____ 5.10 Patients must have a chest x-ray or CT scan of the chest and a CT scan of the abdomen and pelvis within 28 days prior to registration.

Chest x-ray or CT Scan Date ______________ Positive/Negative (circle one, prior to data submission as outlined in Section 14.4)

Abdomen and Pelvis CT Scan Date ______________ Positive/Negative (circle one, prior to data submission as outlined in Section 14.4)

____ 5.11 Patients must have a β_2 microglobulin performed within 28 days prior to registration, and the value must be known at the time of registration for stratification purposes.

β_2 microglobulin value ______________ IULN ______________

Date ______________

____ 5.12 Patients must not have clinical evidence of central nervous system involvement by lymphoma. Any laboratory tests that are performed to assess clinical signs of central nervous system involvement must have been performed within 42 days prior to registration, and the results must be negative.

Date tests performed (if needed) ______________________

____ 5.13 Patients must not have received prior chemotherapy for lymphoma. Patients must not have received prior monoclonal antibodies for malignant disease. (Prior treatment with prednisone for non-lymphoma related illness(es) is allowed.)

____ 5.14 Patients must not have received prior radiation therapy for lymphoma.

____ 5.15 Patients must not have a history of hypersensitivity to iodine.

____ 5.16 All patients must have a Zubrod performance status of 0, 1 or 2 (see Section 10.4).

____ 5.17 Patients must have passed their 18th birthday.

__ 5.18 Patients must have granulocytes >1,500 /µl and platelets > 100,000/µl within 28 days prior to registration.

Granulocytes ______________________ Date ______________________

Platelets ______________________ Date ______________________

___ 5.19 Patients must have fewer than 5000 circulating lymphoid cells per µl on a white blood cell differential count within 28 days prior to registration.

Circulating lymphoid cells/µl ______________ Date ______________________

____ 5.20 Patients with a history of impaired cardiac status (including history of severe coronary artery disease, cardiomyopathy, congestive heart failure or serious arrhythmia) are not eligible. If the patient's cardiac history is questionable, a MUGA Scan or 2-d ECHO must be obtained within 42 days prior to registration (patients with ejection fractions < institutional lower limit of normal will not be eligible).

MUGA scan or 2-d ECHO (if performed) ______________________

ILLN ______________ Date MUGA or 2-d ECHO obtained ______________

CLOSED EFFECTIVE 09/15/2008

SWOG Authorized Document

SWOG Patient No. ______________________________________

Patient's Initials (L,F,M) ___________________________________

______ 5.21 Patients known to be HIV-positive are not eligible. (For justification of this exclusion, see Section 2.0.) Patients at high risk of Hepatitis B virus infection should be screened before initiation of rituximab.

_____ 5.22 Pregnant or nursing women may not participate. Women or men of reproductive potential must agree to use an effective contraceptive method from the time of registration to 6 months after receiving the Iodine-I31 Anti-B1 Antibody. (For justification of this exclusion, see Section 2.0 - 3.0)

____ 5.23 No prior malignancy is allowed except for adequately treated basal cell or squamous cell skin cancer, in situ cervical cancer, or other cancer for which the patient has been disease-free for five years.

5.24 If Day 28 or 42 falls on a weekend or holiday, the limit may be extended to the next working day. **In calculating days of tests and measurements, the day a test or measurement is done is considered Day 0. Therefore, if a test is done on a Monday, the Monday four weeks later would be considered Day 28. This allows for efficient patient scheduling without exceeding the guidelines.**

____ 5.25 All patients must be informed of the investigational nature of this study and give written informed consent in accordance with institutional and federal guidelines.

____ 5.26 At the time of patient registration, the treating institution's name and ID number must be provided in order to ensure that the current (within 365 days) date of institutional review board approval for this study has been entered into the data base.

CLOSED EFFECTIVE 09/15/2008

SWOG Authorized Document

6.0 STRATIFICATION FACTORS

A dynamic allocation scheme will be used to randomize patients to the two remaining arms at registration. Arm 1: CHOP chemotherapy with concurrent rituximab antibody **[ARM 1, CHOP ONLY, WAS PERMANENTLY CLOSED EFFECTIVE 12/15/02]**, Arm 2: CHOP followed by I-131 antiB-1-antibody (tositumomab). Patients will be balanced with respect to the following stratification factor:

β_2 microglobulin > IULN: yes vs. no

7.0 TREATMENT PLAN

For treatment or dose modification related questions, please contact Dr. Press at 206/667-1872 or Dr. Maloney at 206/667-5616.

7.1 Good Medical Practice

The following pre-study tests should be obtained within 28 days prior to initial registration in accordance with good medical practice. Results of these tests do not determine eligibility and minor deviations would be acceptable if they do not impact on patient safety in the clinical judgement of the treating physician. The Study Coordinator must be contacted if there are significant deviations, in the opinion of the treating investigator, in the values of these tests.

a. A pretreatment serum bilirubin < 2 x the institutional upper limit of normal and a serum creatinine < 2 x the institutional upper limit of normal.

b. A serum level of LDH (lactose dehydrogenase) should be obtained.

c. An EKG should be performed within 42 days prior to registration and it should be free of any arrhythmias (excluding sinus arrhythmia or infrequent premature ventricular contractions).

d. Urinalysis, uric acid, and SGOT and/or Alkaline Phosphatase be should performed in order to assess potential treatment-related toxicities.

e. TSH

f. Hepatitis B Virus (HBV) screening

7.2 At the time of registration, patients will be randomized to one of the three treatment arms described below.

NOTE: Institutions with patients randomized to Arm 3 will be contacted by GlaxoSmithKline (by telephone) to provide assistance, discuss the drug order process, obtain current drug shipping address information and arrange for on-site training in the administration of the I-131 Anti-B1 antibody, if needed, (see Sections 7.5 and 15.1). A protocol specific training is required for all institutions with patients randomized to Arm 3. In addition, members of the treatment team are required to participate in the general Bexxar training program. Drug orders cannot be processed without this requirement being met.

CLOSED EFFECTIVE 09/15/2008

SWOG Authorized Document

7.3 **<u>Arm 1 - CHOP Chemotherapy</u>: (THE CHOP ONLY ARM OF THIS STUDY WAS PERMANENTLY CLOSED, EFFECTIVE 12/15/02.) Treatment with CHOP as described below will be administered every 21 days for a maximum of 6 cycles. Patients with progressive disease at any time while receiving treatment will be removed from protocol treatment (see Section 7.9a). <u>All patients will receive identical starting doses regardless of marrow status</u>.**

TABLE 1 - CHOP CHEMOTHERAPY:

DRUG	DOSE	ROUTE	DAYS	RE-TX Interval
Cyclophosphamide	750 mg/m^2	IV infusion over 15 minutes	1	q 21 days for up to 6 cycles
Doxorubicin	50 mg/m^2	Slow IV injection	1	q 21 days for up to 6 cycles
Vincristine	1.4 mg/m^2 (max 2.0 mg)	Slow IV injection	1	q 21 days for up to 6 cycles
Prednisone*	100 mg	PO	1 - 5	q 21 days for up to 6 cycles

***Prednisone should be omitted if the patient has a history of recent active peptic ulcer disease or if peptic ulcer symptoms occur during treatment. The reason for omitting this drug must be noted on flow sheet.**

Patients randomized to Arm 1 (CHOP only) <u>should not</u> receive any further treatment after completion of the six cycles of CHOP until progression.

See Section 8.2 for CHOP treatment dose modifications.

7.4 **<u>Arm 2 - CHOP + concurrent rituximab</u>**:

a. <u>CHOP Chemotherapy</u>: Patients randomized to Arm 2 will be treated with six cycles of CHOP + six doses of rituximab according to the schedule described by Czuczman et al. *(20)* The CHOP chemotherapy will be administered at three week intervals as described below for a maximum of six cycles.

b. <u>Rituximab Infusions:</u> The six rituximab infusions will be administered concurrently with the CHOP chemotherapy as described below.

CLOSED EFFECTIVE 09/15/2008

SWOG Authorized Document

TABLE 2 - CHOP CHEMOTHERAPY PLUS RITUXIMAB INFUSIONS:

DRUG	DOSE	ROUTE	DAYS	NOTES
Cyclophosphamide	750 mg/m^2	IV infusion over 15 minutes	8, 29, 50, 71, 92, 113	
Doxorubicin	50 mg/m^2	Slow IV injection	8, 29, 50, 71 92, 113	
Vincristine	1.4 mg/m^2 (max 2.0 mg)	Slow IV injection	8, 29, 50, 71 92, 113	
Prednisone*	100 mg	PO	First 5 days of each CHOP cycle	
Rituximab Infusions #1, #2	375 mg/m^2	Slow IV (see Section 7.4c for dose rate)	1, 6	CHOP Cycle 1 only
Rituximab Infusions #3, #4	375 mg/m^2	slow IV (see Section 7.4c for dose rate)	48, 90 (48 hours before beginning CHOP cycle)	CHOP Cycles 3 and 5 ONLY
Rituximab Infusions #5, #6	375 mg/m^2	slow IV (see Section 7.4c for dose rate)	134,141	3 and 4 weeks FOLLOWING CHOP Cycle 6

*Prednisone should be omitted if the patient has a history of recent active peptic ulcer disease or if peptic ulcer symptoms occur during treatment.

Rituximab infusions #1 and #2 will be administered on Days 1 and 6 before the first CHOP cycle, with CHOP chemotherapy being administered on Day 8.

Rituximab infusions #3 and #4 will be administered on Days 48 and 90 (two days before the third and fifth cycles of CHOP).

Rituximab infusions #5 and #6 will be given after Cycle 6 of CHOP on Days 134 and 141, respectively (three and four weeks, respectively after Cycle 6 of CHOP).

c. <u>Although patients with non-Hodgkin's lymphoma that are eligible for this study should not have markedly elevated numbers of circulating malignant cells, the following should be noted.</u> For some patients, tumor lysis syndrome has been reported to occur within 12 - 24 hours after the first rituximab infusion. Prophylaxis to prevent the rare event of tumor lysis syndrome in patients with bulky tumors (> 10 cm) or with markedly elevated numbers of circulating malignant cells is recommended.

Oral premedication (2 tablets [350 mg]) of acetaminophen and 50 to 100 mg oral diphenhydramine hydrochloride) may be administered 30 - 60 minutes prior to starting each infusion of rituximab. A peripheral or central intravenous (IV) line will be established. During the rituximab infusion, the patients vital signs (blood pressure, pulse, respiration, temperature) should be monitored every 15 minutes x 4 until stable and then hourly until the infusion is discontinued. Available at bedside prior to rituximab administration will be ephinephrine for subcutaneous injection, diphenhydramine hydrochloride for intravenous injection, and resuscitation equipment for the emergency management of anaphylactoid reactions. <u>The initial dose rate at the time of the first rituximab infusion should be 50 mg/hr for the first hour</u>. If no toxicity is seen, the dose rate may be escalated

CLOSED EFFECTIVE 09/15/2008

SWOG Authorized Document

gradually (50 mg/hr increments at 30-minute intervals) to a maximum of 300 mg/hr. If the first dose of rituximab is well tolerated, the starting flow rate for the administration of subsequent doses will be 100 mg/hr, then increased gradually (100 mg/hr increments at 30-minute intervals) not to exceed 400 mg/hr.

Precautionary hospitalization for patients experiencing severe symptoms or infusion reactions which do not resolve after discontinuation or completion of the cycle is recommended.

See Section 8.2 for CHOP treatment dose modifications.

See Section 8.3 for rituximab dose modifications.

7.5 **Arm 3 - CHOP followed by tositumomab and I-131 tositumomab: Institutions must have completed an on-site training session with GlaxoSmithKline representatives to review the details of drug administration and dosimetry prior to treating a patient with tositumomab and Iodine-131 tositumomab. The training session must occur prior to the dosimetric infusion of I-131 tositumomab, and the date of the training session must be noted on the S0016 Tositumomab Treatment Form (Form #57993). For subsequent patients, the original training session date must still be noted on the S0016 Tositumomab Treatment Form (Form #57993).**

GlaxoSmithKline will contact each institution (by telephone) to provide assistance and arrange for training (if required) shortly after randomization of the first patient to Arm 3 (see Section 15.1). (The training session must be performed only for the FIRST patient randomized to ARM 3 of this study at any one institution.) However, retraining is available upon institutional request.

a. CHOP chemotherapy: Patients randomized to Arm 3 will receive six cycles of CHOP chemotherapy at three week intervals as described in Table 3 of Section 7.5c.

b. Re-evaluation: Patients on Arm 3 will be re-evaluated no earlier than 4 weeks but no later than 8 weeks after the completion of CHOP chemotherapy (see Section 9.3). **The following conditions must be met prior to proceeding with I-131 tositumomab antibody treatment. Patients who do not qualify for I-131 tositumomab antibody treatment upon re-evaluation will be removed from protocol treatment.**

1. The training session with GlaxoSmithKline must have been completed.

2. Patients must have no more than 25% of the intratrabecular marrow space involved by lymphoma in bone marrow biopsy specimens as assessed microscopically after completion of 6 cycles of CHOP chemotherapy. Bilateral posterior iliac crest core biopsies are required if the percentage of intratrabecular space involved exceeds 10% on a unilateral biopsy. The mean of bilateral biopsies must be no more than 25%. The procedure for bilateral bone marrow biopsy analysis of marrow involvement is included in Appendix 19.2.

3. Patients must have granulocytes ≥ 1,500/µl and platelets ≥ 100,000/µl within 14 days of the planned dosimetric infusion.

Patients must not have active obstructive hydronephrosis.

CLOSED EFFECTIVE 09/15/2008

SWOG Authorized Document

c. **TABLE 3** - CHOP CHEMOTHERAPY PLUS TOSITUMOMAB INFUSIONS:

Administration of I-131-Tositumomab: Tositumomab will be administered after the completion of CHOP chemotherapy and re-evaluation as described below.

DRUG	DOSE	ROUTE	DAYS	RE-TX Interval
Cyclophosphamide	750 mg/m^2	IV infusion over 15 minutes	1	q 21 days for up to 6 cycles
Doxorubicin	50 mg/m^2	Slow IV injection	1	q 21 days for up to 6 cycles
Vincristine	1.4 mg/m^2 (max 2.0 mg)	Slow IV injection	1	q 21 days for up to 6 cycles
Prednisone*	100 mg	PO	1 - 5	q 21 days for up to 6 cycles
Unlabeled Anti-B1 Antibody **	450 mg	IV over 1 hour	Day 134 ƒ	
Dosimetric Dose +	35 mg	IV over 20 minutes	Day 134	
Unlabeled Anti-B1 Antibody**	450 mg	IV over 1 hour	Day141 √	
Therapeutic Dose +	35 mg 20 minutes	IV over	Day141 √	

* Prednisone should be omitted if the patient has a history of recent active peptic ulcer disease or if peptic ulcer symptoms occur during treatment.

** Patients must receive 450 mg of unlabeled Anti-B1 Antibody (to 50 mL using 0.9% sodium chloride) prior to both the dosimetric and therapeutic dose. Patients must be premedicated with acetaminophen 650 mg po and diphenhydramine 50 mg po prior to administration of unlabeled anti-B1 antibody (see Sections 7.5c.2ii and 7.5c.3ii).

\+ Patients must receive SSKI, Lugol's Solution or Potassium Iodide at least 24 hours prior the first infusion of the dosimetric dose. Treatment will continue until 14 days after the last infusion of the therapeutic dose (see Section 7.5c.2iii).

ƒ Ideally, the dosimetric infusion will be given on Day 134 and the therapeutic infusion on Day 141. Due to the logistics of ordering, receiving and administering I-131-tositumomab, a 4 week period of flexibility after Day 134 will be allowed within which the radiolabeled antibody therapy may be given. <u>However, no more than 14 days are allowed between the dosimetric and therapeutic infusions of tositumomab.</u>

√ See Section 7.5c.3ii.

1. <u>Preparation, Dosing, and Administration</u>:

Patients will undergo two phases of tositumomab administration. The first phase, termed "dosimetric dose", involves the intravenous (IV) administration of a low-radioactive dose (five mCi) of Iodine-131 anti-B1 antibody for the purpose of determining the rate of total body clearance of radioactivity (residence time) so that a total body radiation dose can be calculated (see Appendix 19.1). The calculated total body radiation dose per mCi administered can then be used to determine how many mCi of Iodine-131 conjugated with anti-B1 antibody will be required to deliver the total body radiation dose in the second phase of the study, termed "therapeutic dose." <u>Both the dosimetric dose and the</u>

CLOSED EFFECTIVE 09/15/2008

SWOG Authorized Document

therapeutic dose will be immediately preceded by an infusion of 450 mg unlabeled anti-B1 antibody (see Sections 7.5c2.iv and 7.5c.3ii).

Administration of the radiolabeled anti-B1 antibody will be performed by personnel authorized to deliver such doses of radioisotope to patients. Special radiation precautions will be used during and after the administration of the therapeutic dose, as required by the national and/or regional regulations for the radiopharmaceutical industry. Restrictions on patient contact with others will be set in accordance with these regulatory guidelines [Nuclear Regulatory Commission (NRC) and state laws]. The dosimetric and therapeutic doses may be given as either an outpatient or inpatient procedure depending on current NRC and state regulations.

NOTE: Unused drug must be returned as specified in Section 3.5c.

2. Dosimetric Dose:

i. Preparation of Unlabeled Anti-B1 Antibody

To prepare unlabeled anti-B1 antibody for administration to patients, 450 mg unlabeled anti-B1 antibody is sterilely-removed from the product vials and diluted to 50 mL using 0.9% sodium chloride for injection.

ii. Preparation of Dosimetric Dose (i.e., Tracer Dose)

To prepare the dosimetric dose, an amount of anti-B1 antibody (33 - 34 mg) is added to the trace-labeled antibody preparation (1-2 mg of anti-B1 antibody radiolabeled with 5 mCi of 131-Iodine) sufficient to result in a final amount of 35 mg of anti-B1 antibody. This latter preparation is then diluted to a final volume of 30 mL using 0.9% sodium chloride for injection.

iii. Administration of Saturated Solution Potassium Iodide (SSKI), Lugol's Solution, or Potassium Iodide Tablets

Patients will be treated with either saturated solution of potassium iodide (SSKI) four drops po tid, Lugol's solution 20 drops po tid, or potassium iodide tablets 130 mg po qd starting at least 24 hours prior to the first infusion of the Iodine-131 Anti-B1 Antibody (i.e., the dosimetric dose) and continuing for 14 days following the last infusion of Iodine-131 Anti-B1 Antibody (i.e., therapeutic dose). The SSKI or Lugol's solution may be given with juice or cola to mask taste. In no instance should a patient receive the dosimetric dose of Iodine-131 Anti-B1 Antibody if they have not yet received at least 3 doses of SSKI, three doses of Lugol's solution, or one 130 mg potassium iodide tablet (at least 24 hours prior to the dosimetric dose). Patients should be monitored for compliance with regard to SSKI, Lugol's solution, or potassium iodide tablets.

All concomitant medications given in conjunction with the administration of tositumomab treatment must be recorded in the comments section of the S0016 Tositumomab Treatment Form (Form #57993).

CLOSED EFFECTIVE 09/15/2008

SWOG Authorized Document

iv. Administration of Dosimetric Dose

On Day 1, patients will receive the intravenous (IV) administration of 450 mg unlabeled anti-B1 antibody followed by the IV administration of the dosimetric dose (five mCi of Iodine-131 anti-B1 antibody). The unlabeled antibody must be administered through a 0.22 micron in-line filter. The in-line filter may remain connected to or removed from the infusion line following the unlabeled antibody. A new filter should not be added for the radiolabeled infusion. Thirty to sixty minutes before the unlabeled anti-B1 antibody infusion, patients will be premedicated with acetaminophen 650 mg po and diphenhydramine 50 mg po (unless the patient is hypersensitive to acetaminophen or diphenhydramine). Unlabeled anti-B1 antibody (see Section 7.5c.2i) will then be given as an intravenous (IV) infusion over 1 hour or longer depending on infusion-related adverse experiences. The dosimetric dose (see Section 7.5c2ii) will be given as an intravenous infusion over 20 minutes. At the end of the infusion of the dosimetric dose, the syringe or IV bag must be refilled with 0.9% sodium chloride and the contents infused over a period of 10 minutes. Vital signs must be taken every 15 minutes during each of the anti-B1 antibody infusions.

v. Whole Body Dosimetry

Whole body dosimetry will be performed separately for each patient as described in Appendix 19.1d, using the worksheets provided. For all patients, whole body anterior gamma camera scans will be obtained within one hour after the completion of the administration of the dosimetric dose on Day 1 (Day 134 of Arm 3 treatment) before any urination, and then either on Day 3, 4, or 5 (Days 136, 137 or 138) after urination and again on either Day 7 or 8 (Day 140 or 141) after urination using a gamma camera with appropriate medium- or high-energy collimator. The anterior whole body scans will be obtained at 30 -100 cm/minute scan speed. Anterior whole body counts, anterior background counts, and anterior counts of a calibrated standard will be obtained and recorded. All static and whole body scan images for dosimetry will be retained electronically for submission upon request to GlaxoSmithKline or its designee.

The above determined counts will be used to calculate the activity to be administered to deliver 75 cGy (unless adjusted for obesity and/or platelet count - see below). The mCi dose will be calculated as described in Appendix 19.1 and accompanying worksheets.

Dose Adjustments based on weight and platelet counts:

For excessively obese patients, the calculations to determine the Iodine-131 anti-B1 antibody activity to administer will be performed using an upper limit of mass (maximum effective mass) based upon height and gender (see Table 1, Appendix 19.1d).

CLOSED EFFECTIVE 09/15/2008

SWOG Authorized Document

The administered activity (mCi of Iodine-131 anti-B1 antibody) for patients with platelet counts of 100,000 - 149,999 cells/mm^3 will be adjusted to deliver 65 cGy, with additional adjustment of activity for obesity, if indicated. **Iodine I-131 antibody should not be given if platelets are less than 100,000/mm^3.**

The dose calibrator used for measuring the mCi of activity of Iodine-131 anti-B1 antibody to be administered to the patient must be appropriately calibrated.

The dosimetry worksheets for the first 3 patients at each clinical site must be submitted to GlaxoSmithKline by fax to confirm that the calculations were performed correctly (Fax: 877/279-1512). A dosimetry hotline will be maintained by GlaxoSmithKline to assist in calculation of the proper therapeutic dose (Service Center, toll free 877/423-9927).

3. Therapeutic Dose:

i. Preparation

To prepare the therapeutic dose, an amount of anti-B1 antibody is added to the radiolabeled preparation [anti-B1 antibody labeled with enough 131 Iodine to administer the specified whole body radiation dose calculated for the patient from the dosimetric dose] sufficient to result in a final amount of 35 mg of anti-B1 antibody, unless the amount of anti-B1 antibody in the radiolabeled preparation is already ≥ 35 mg. This latter preparation is then diluted to a final volume of 30 mL using 0.9% sodium chloride for injection. In rare cases, greater than 30 ml of Iodine-131 anti-B1 antibody will be required and the dose will then be prepared in 60 ml.

ii. Administration

The therapeutic dose is to be given 7 days after the administration of the dosimetric dose (may be delayed but no longer than 14 days after dosimetric dose). **Those patients who experienced an anaphylactic response or serious adverse experience felt to be related to study drug during or following trace-labeled antibody administration will be removed from protocol treatment.** Patients will be premedicated with acetaminophen and diphenhydramine as they were prior to the dosimetric dose. Patients should also still be receiving SSKI or Lugol's solution as described in Section 7.5c.2iii. The unlabeled antibody must be administered through a 0.22 micron in-line filter. The in-line filter may remain connected to or removed from the infusion line following the unlabeled antibody. A new filter should not be added for the radiolabeled infusion. Unlabeled anti-B1 antibody will then be given as an intravenous (IV) infusion over 1 hour or longer depending on infusion-related adverse experiences. The therapeutic dose will be given as an intravenous infusion over 20 minutes. At the end of the infusion of the therapeutic dose, the syringe or IV bag must be refilled with 30 ml 0.9% sodium chloride and the contents infused over a period of 10 minutes. Vital signs must be taken every 15 minutes during each of the anti-B1 antibody infusions.

CLOSED EFFECTIVE 09/15/2008

SWOG Authorized Document

7.6 CNS Prophylaxis: There will be no CNS prophylaxis on any of the three treatment arms of this protocol.

7.7 Allopurinol: To prevent the rare event of tumor lysis syndrome in patients with bulky tumors (> 10 cm), oral or IV fluid intake in excess of 2,000 ml daily is encouraged during therapy for all patients on the study. In addition, the routine administration of allopurinol (300 mg/d) is also recommended prior to and during the first cycle of therapy on all three arms of the study. If rash occurs, allopurinol can be discontinued.

7.8 Restaging: All patients will be assessed for response 200 and 365 days after initiation of therapy and then every six months as specified on the study calendars (see Section 9.0). Day 200 was chosen for restaging because this uniform assessment timepoint should allow evaluation of patients on all three arms of the protocol at least four weeks after completion of therapy.

Restaging will include history, physical examination, complete blood cell counts with differential, chest X-ray or Chest CT scan (depending on which was done prestudy), abdominal and pelvic CT scans, and bone marrow aspiration and biopsy (only required at Day 200 if marrow was initially involved with lymphoma). For all patients who do not progress, a bone marrow aspirate and biopsy is required at Day 365 for routine bone marrow testing and t(14;18) studies (see Section 15.4).

7.9 Criteria for Removal from Protocol Treatment

a. Documented progression of disease as defined in Section 10.2f.

b. Development of unacceptable toxicity, as defined in Section 8.0.

c. Failure to meet criteria for I-131 antibody administration following completion of CHOP chemotherapy (for Arm 3 patients only, see Section 7.5b).

d. Completion of protocol treatment.

e. The patient may withdraw from the study at any time for any reason.

7.10 All reasons for discontinuation of treatment must be documented on the Off Treatment Notice (Form #22204).

7.11 All patients will be followed until death.

8.0 TOXICITIES TO BE MONITORED AND DOSAGE MODIFICATIONS

8.1 This study will utilize the CTC (NCI Common Toxicity Criteria) Version 2.0 for toxicity and Adverse Event reporting. A copy of the CTC version 2.0 can be downloaded from the CTEP home page (http://ctep.info.nih.gov). **All appropriate treatment areas should have access to a copy of the CTC Version 2.0.**

8.2 CHOP Dose Modification (for Arms 1 - 3): In the case of multiple toxicities, dose modifications should be based on the most severe dose-limiting toxicity. **(THE CHOP ONLY ARM OF THIS STUDY WAS PERMANENTLY CLOSED, EFFECTIVE 12/15/02.)**

a. Hematologic Toxicity: The CHOP regimen should be given as described in Section 7.0 if the granulocytes are > 1,500 cells/μl and the platelets are > 100,000 cells/μl by the time the next cycle is due. If the blood counts have not recovered, treatment should be delayed one week and counts repeated unless low peripheral counts are due to tumor. If, after two weeks, counts have not yet recovered, the patient should be treated at 75% of the last dose received of cyclophosphamide and doxorubicin.

Grade 3 or 4 infection (NCI Common Toxicity Criteria Version 2.0) due to chemotherapy-related neutropenia requires a decrease in the doses of

CLOSED EFFECTIVE 09/15/2008

SWOG Authorized Document

cyclophosphamide and doxorubicin to 75% of the last dose received. Re-escalation is at the discretion of the treating physician. In this study, growth factors will not be administered to prevent neutropenia.

For patients who experience Grade 3 or 4 neutropenia or develop neutropenic fever between cycles of chemotherapy, growth factors may be added to all subsequent cycles of chemotherapy. Dose of CHOP may be re-escalated on future cycles at the discretion of the investigator.

Pegfilgrastim (pegylated G-CSF), filgrastim (G-CSF), and sargramostim (GM-CSF) are acceptable growth factors. These growth factors are commercially available and should be purchased through third party mechanisms. The Southwest Oncology Group will not provide growth factors for this study.

b. Impaired Hepatic Function: All patients with bilirubin ≤ 2 x the institutional upper limit of normal will receive a full initial dose of doxorubicin and vincristine. If the bilirubin rises to > 2 x the institutional upper limit of normal (but ≤ 5 x IULN), the doxorubicin and vincristine doses must be reduced by 50% to avoid undue hepatic toxicity. Full doses should be given once the bilirubin is ≤ 2 x the institutional upper limit of normal. If the bilirubin rises to > 5 x the institutional upper limits of normal, doxorubicin and vincristine should be discontinued for that cycle. If hepatic function has not recovered to ≤ 2 x the institutional upper limits of normal by the time the next cycle is due, then remove patient from protocol treatment. In cases of obstruction of the biliary duct by tumor mass, a biliary drainage shunt should be placed prior to chemotherapy.

Bilirubin	Doxorubicin Dose	Vincristine Dose
≤ 2 x IULN	100%	100%
> 2 - 5 x IULN	50%	50%
> 5 x IULN	0%	0%

c. Impaired Renal Function: All patients with serum creatinine levels ≤ 2 x the institutional upper limit of normal will receive full doses of all drugs. If the creatinine rises > 2 x the institutional upper limit of normal, the dose of cyclophosphamide must be reduced by 25%. Re-escalation to full dose is at the discretion of the treating physician if the serum creatinine level drops to ≤ 2 x the institutional upper limit of normal.

d. Hemorrhagic cystitis: Cyclophosphamide will be discontinued and the patient removed from protocol treatment if Grade 3 or 4 hemorrhagic cystitis resulting from this drug occurs. Adequate fluid intake and allopurinol are recommended during therapy.

e. Neuropathy: Patients experiencing Grade 3 vincristine-neuropathy (e.g., obstipation, weakness) will have the dose of vincristine reduced by 50% for all further cycles of CHOP. Patients experiencing Grade 4 vincristine neuropathy will have vincristine omitted from all future cycles of CHOP.

8.3 Rituximab Antibody Dose Modification and Cycle Delay (for Arm 2):

a. Patients may experience transient fever and rigors with infusion of chimeric anti-CD20 antibody. If Grade 3 fever (or Grade 2 fever with rigors) or Grade 2 rigors are noted, the antibody infusion should be temporarily discontinued, the patient should be observed, and the severity of the side effects should be evaluated.

CLOSED EFFECTIVE 09/15/2008
SWOG Authorized Document

The patient should be treated according to the best available local practices and procedures. Following observation, when fever resolves to Grade 2 or less and rigors to Grade 1 or less, the infusion should be continued, initially, at 1/2 the previous rate. Following the antibody infusion , the IV line should be kept open for medications, as needed.

b. Hypotension, bronchospasm and angioedema have occurred as part of an infusion related symptom complex. If a Grade 3 or greater hypersensitivity/allergic reaction occurs, the rituximab infusion should be interrupted and may be resumed at a 50% reduction in rate when symptoms have completely resolved. Treatment with diphenhydramine and acetaminophen is recommended; additional treatment with broncodialators or IV saline may be used at the physician's discretion. Precautionary hospitalization for patients experiencing severe infusion symptoms which do not resolve after discontinuation of the cycle is recommended.

If there are no complications during the rituximab infusion, the IV line may be discontinued one hour after completion of the infusion. If complications occur during the rituximab infusion, the patient should be observed for two hours after the completion of the infusion. If a patient experiences a Grade 3 toxicity that persists until the next scheduled infusion, the patient must discontinue treatment until toxicities have resolved to Grade 2 or less. If treatment is delayed for more than three weeks, remove the patient from protocol treatment.

c. Tumor Lysis Syndrome: Appropriate medical therapy should be provided for patients who develop tumor lysis syndrome. Following treatment for and resolution of tumor lysis syndrome, subsequent rituximab therapy may be administered in conjunction with prophylactic therapy for this syndrome. Contact the Study Coordinator prior to resuming treatment in these patients.

d. Hepatitis B Reactivation with Related Fulminant Hepatitis and Other Viral Infections: Carriers of hepatitis B should be closely monitored for clinical and laboratory signs of active HBV infection and for signs of hepatitis throughout their study participation. Patients with any evidence of active hepatic disease or known HBV infection should be managed as clinically appropriate and should only receive rituximab if they have control of the infection and are adequately informed of the risks. Patients who have never received vaccination for HBV, and have not had serologic testing for HBsAg, should be tested for surface antigen positivity.

In patients who develop progressive multifocal leukoencephalopathy (PML), rituximab should be discontinued and reductions or discontinuation of concomitant immunosuppressive therapy and appropriate treatment, including antiviral therapy, should be considered. Physicians should consider PML in any patients presenting with new onsent neurologic manifestations, particularly in patients with systemic lupus erythematosus (SLE) or lymphoid malignancies. Consultation with a neurologist, brain MRI, and lumbar puncture should be considered as clinically indicated. There are no known interventions that can reliably prevent PML or adequately treat PML if it occurs.

e. Severe Mucocutaneous Reactions: All patients on and off rituximab therapy should be closely monitored for signs and symptoms suggestive of severe cutaneous and mucocutaneous reactions. Should these symptoms arise, discontinue rituximab therapy (if applicable) and support as clinically indicated.

CLOSED EFFECTIVE 09/15/2008

SWOG Authorized Document

f. Cardiovascular events: Patients with rheumatoid arthritis (RA) are at increased risk for cardiovascular events compared to the general population. Patients with RA should be monitored throughout the infusion, and rituximab should be discontinued in the event of a serious or life-threatening cardiac event.

Patients who develop clinically significant arrhythmias should undergo cardiac monitoring during and after subsequent infusions of rituximab. Patients with pre-existing cardiac conditions, including arrhythmias and angina, that have had recurrences of these events during rituximab therapy should be monitored throughout the infusion and immediate post-infusion period. Patients off rituximab therapy should be closely monitored for signs and symptoms suggestive of life-threatening cardiac events and supported as clinically indicated.

g. Bowel obstruction and perforation: Complaints of abdominal pain, especially early in the course, should prompt a thorough diagnostic evaluation and appropriate treatment. If patient experiences a bowel obstruction or perforation, discontinue rituximab therapy. Patients off rituximab therapy should be closely monitored for signs and symptoms suggestive of bowel obstruction and supported as clinically indicated.

h. Renal: Discontinuation of rituximab should be considered for those with rising serum creatinine or oliguria.

8.4 Iodine-131 Anti-B1 Antibody Dose Modification and Cycle Delay (form Arm 3):

Iodine-131 should be given as specified in Section 7.5 as long as counts have recovered to granulocytes ≥ 1,500 and platelets ≥ 100,000.

a. Dose Adjustments based on weight and platelet counts:

For excessively obese patients, the calculations to determine the Iodine-131 anti-B1 antibody activity to administer will be performed using an upper limit of mass (maximum effective mass) based upon height and gender (see Table 1, Appendix 19.1d).

The administered activity (mCi of Iodine-131 anti-B1 antibody) for patients with platelet counts of 100,000 - 149,999 cells/mm^3 will be adjusted to deliver 65 cGy, with additional adjustment of activity for obesity, if indicated. **Iodine-131 antibody should not be given if platelets are less than 100,000/mm^3.**

The dose calibrator used for measuring the mCi of activity of Iodine-131 anti-B1 antibody to be administered to the patient must be appropriately calibrated.

b. Other dose adjustments:

During the administration of the unlabeled anti-B1 antibody, tracer, and therapeutic doses, emergency support for anaphylaxis is to be readily available, including a tray for epinephrine, diphenhydramine, hydrocortisone, a laryngoscope, and an endotracheal tube. Although acute adverse experiences occurring during the infusion or up to 24 hours after the infusion of anti-B1 antibody have been infrequent, based upon past experience, symptoms of fever, nausea, vomiting, rigors, hypotension, pruritis, tachycardia, erythematous rash, urticaria, mucus membrane congestion, arthralgias, and myalgias may occur. The patient should be treated according to physician's judgment. However, it is recommended that acetaminophen 650 mg po and/or diphenhydramine 50 mg

CLOSED EFFECTIVE 09/15/2008

SWOG Authorized Document

po or IV be given to control these symptoms if they occur. Severe rigors should also be treated at the physician's discretion but may be controlled by meperidine 25 - 50 mg IV. Experience has shown that rigors generally abate within 30 minutes without pharmaceutical intervention.

If any of these toxicities occur during antibody infusion, the rate of antibody infusion should be decreased as indicated below:

Infusion Rate Adjustment

Fever	Rigors	Mucosal Congestion/ Edema	% Drop in Systolic BP	Infusion Rate Adjustment
Grade 1 (38.0 - 39.0°C)	Grade 1 -2 (Mild to Moderate)	Grade 1 - 2 (Mild to Moderate)	30 - 49	Decrease by 1/2
Grade ≥ 2 (≥ 39°C)	Grade ≥ 3 (Severe)	Grade ≥ 3 (Severe)	≥ 50	Stop Infusion*

* Temporarily discontinue infusion until adverse experiences have reversed (generally 15 to 30 min.) and then resume infusion at 25 - 50% of initial rate.

c. Use of Colony Stimulating Factors (CSF) and Platelet and Red Blood Cell Transfusions:

Colony Stimulating Factors (CSF), such as pegfilgrastim (pegylated G-CSF), filgrastim (G-CSF) and sargramostim (GM-CSF), should be administered only in accordance with published ASCO guidelines. *(36)* Use of CSF under these conditions will be at the discretion of the treating investigator, but must be recorded in the comments section of the appropriate treatment form.

Platelet transfusions should be administered only in patients with Grade 3 or 4 thrombocytopenia with obvious bleeding. The use of platelet and red cell transfusions under these conditions will be at the discretion of the treating investigator, but must be recorded in the comments section of the appropriate treatment form.

8.5 For treatment or dose modification related questions, please contact Dr. Press at 206/667-1872 or Dr. Maloney at 206/667-5616.

8.6 Unexpected or fatal toxicities (including suspected reactions) must be reported to the Operations Office, to the Study Coordinator, to the IRB and the NCI. The procedure for reporting adverse reactions is outlined in Section 16.0.

CLOSED EFFECTIVE 09/15/2008

SWOG Authorized Document

9.0 STUDY CALENDAR **S0016**, "A Phase III Trial of CHOP + Rituximab vs. CHOP + Iodine-131-Labeled Monoclonal Anti-B1 Antibody (Tositumomab) for Treatment of Newly Diagnosed Follicular Non-Hodgkin's Lymphomas"

(The CHOP only arm of this study was permanently closed, effective 12/15/02.)

9.1 S0016 STUDY CALENDAR: ARM 1 - CHOP ONLY

								Ω	Ω	√
		Cycle 1	Cycle 2	Cycle 3	Cycle 4	Cycle 5	Cycle 6			
**REQUIRED STUDIES**	**PRE**	**Day**	**Day**	**Day**	**Day**	**Day**	**Day**	**Day**	**Day**	**Follow**
	**STUDY**	**1**	**22**	**43**	**64**	**85**	**106**	**200**	**365**	**Up**
**PHYSICAL**										
History & Physical Exam	X		X	X	X	X	X	X	X	X
Weight & Performance Status	X							X	X	X
Tumor Assessment	X							X*ƒ*	X*ƒ*	X*ƒ*
Toxicity Notation		X	X	X	X	X	X	X	X	X
**LABORATORY**										
CBC, Platelets & Differential	X		X	X	X	X	X	X	X	X
Serum Creatinine	X ß		X	X	X	X	X			X
Bilirubin	X ß		X	X	X	X	X			X
LDH	X ß									X
ß2 Microglobulin	X									
Immunophenotyping (CD20)	X									
Thyroid Stimulating Hormone (TSH)								X	X	X
Urinalysis	X ß									
Uric acid	X ß									
SGOT and/or Alk Phosphatase	X ß									X
Materials for pathology review ¥	X									
Bone marrow asp./biopsy	X							X*	X	X*
Bone marrow submission for bcl2 testing ¢	X								X	
**X-RAYS AND SCANS**										
Chest X-Ray or Chest CT *ƒ*	X*ƒ*							X*ƒ*	X*ƒ*	X*ƒ*
CT scan: abdomen, pelvis	X							X	X	X
EKG	X ß									
MUGA	X∑									
**TREATMENT (See Section 7.3) π**										
Cyclophosphamide		X	X	X	X	X	X			
Doxorubicin		X	X	X	X	X	X			
Vincristine		X	X	X	X	X	X			
Prednisone (see Section 7.3)		X	X	X	X	X	X			

CLOSED EFFECTIVE 12/15/2002

NOTE: Data submission forms are found in Section 18.0. Forms submission guidelines may be found in Section 14.0.

ƒ The same scanning technique as baseline must be used to allow uniformity of results.
¥ See Section 12.0.
Ω Restaging must occur on Day 200 and on Day 365 after initiation of CHOP. The same assessments that were used at baseline should be used for restaging.
√ Patients removed from protocol treatment for any reason will be evaluated at that time by repeating CBC with platelets, LDH, creatinine, liver enzymes, CT of chest, abdomen and pelvis, and all pre-treatment scans to evaluate disease. Follow-up evaluations will include all restaging tests (except CT of chest, abdomen and pelvis) and will occur every 6 months for the first two years, then annually thereafter. The CT of chest, abdomen and pelvis will occur every 6 months for the first two years, then annually for seven years or until relapse.
π Patients with progressive disease at any time will discontinue protocol treatment (see Section 10.2f).
ß These tests are suggested at pre-study for Good Medical Practices (see Section 7.1), but must be obtained as listed above for follow-up toxicity assessment.
∑ Required if clinically indicated (see Section 5.19).
* Repeat if initially abnormal
¢ See Section 15.0.

SWOG Authorized Document

9.0 STUDY CALENDAR __S0016__, "A Phase III Trial of CHOP vs CHOP + Rituximab vs. CHOP + Iodine-131-Labeled Monoclonal Anti-B1 Antibody (Tositumomab) for Treatment of Newly Diagnosed Follicular Non-Hodgkin's Lymphomas"

9.2 S0016 ARM 2 - CHOP + Rituximab

														Ω	Ω	√
		Cycle 1			Cycle 2	Cycle 3		Cycle 4	Cycle 5		Cycle 6					
**REQUIRED STUDIES**	**PRE**	**Day**	**Day**	**Day**	**Day**	**Day**	**Day**	**Day**	**Day**	**Day**	**Day**	**Day**	**Day**	**Day**	**Day**	**Follow**
	**STUDY**	**1**	**6**	**8**	**29**	**48**	**50**	**71**	**90**	**92**	**113**	**134**	**141**	**200**	**365**	**Up**
**PHYSICAL**																
History & Physical Exam	X			X	X		X	X		X	X			X	X	X
Weight & Performance Status	X													X	X	X
Tumor Assessment	X													X *ƒ*	X *ƒ*	X *ƒ*
Toxicity Notation		X	X	X	X	X	X	X	X	X	X	X	X	X	X	X
**LABORATORY**																
CBC, Platelets & Differential	X				X		X	X		X	X		X	X	X	X
Serum Creatinine	X ß				X		X	X		X	X		X			X
Bilirubin	X ß				X		X	X		X	X		X			X
LDH	X ß															X
ß2 Microglobulin	X															
Immunophenotyping (CD20)	X															
Thyroid Stimulating Hormone	X ß											X Day 133		X	X	X
Urinalysis	X ß															
Uric acid	X ß															
SGOT and/or Alk Phosphatase	X ß															X
Materials for pathology review ¥	X															
Bone marrow asp./biopsy	X													X*	X	
Bone marrow submission for bcl2 testing ¢	X														X	
HBV screening ◊	X ß															
Serum for HAMA testing Δ												X Day 133		X	X	X Day 596
**X-RAYS AND SCANS**																
Chest X-Ray or Chest CT *ƒ*	X*ƒ*													X*ƒ*	X*ƒ*	
CT scan: abdomen, pelvis	X													X	X	X
EKG	X ß															
MUGA or 2-d ECHO	X∑															
**TREATMENT (See Section 7.4) π**																
Rituximab		X	X			X			X			X	X			
Cyclophosphamide				X	X		X	X		X	X					
Doxorubicin				X	X		X	X		X	X					
Vincristine				X	X		X	X		X	X					
Prednisone (see Section 7.3)				X	X		X	X		X	X					

NOTE: Data submission forms are found in __Section 18.0__. Forms submission guidelines may be found in Section 14.0.

* Repeat if initially abnormal

ƒ The same scanning technique as baseline must be used to allow uniformity of results.

¥ See Section 12.0.

Ω Restaging must occur on Day 200 and Day 365 after initiation of CHOP. The same assessments that were used at baseline should be used for restaging.

√ Patients removed from protocol treatment for any reason will be evaluated at that time by repeating CBC with platelets, LDH, creatinine, liver enzymes, CT of chest, abdomen and pelvis, and all pre-treatment scans to evaluate disease. Follow-up evaluations will include all restaging tests (except CT of chest, abdomen and pelvis) and will occur every 6 months for the first two years, then annually thereafter. The CT of chest, abdomen and pelvis will occur every 6 months for the first two years, then annually for seven years or until relapse.

π Patients with progressive disease at any time will discontinue protocol treatment (see Section 10.2f).

ß These tests are __suggested__ at pre-study for Good Medical Practices (see Section 7.1), but __must__ be obtained as listed above for follow-up toxicity assessment.

∑ Required if clinically indicated (see Section 5.19).

¢ See Section 15.0.

◊ Recommended for patients at high risk of HBV infection.

Δ See Section 15.5 for collection and submission instructions.

SWOG Authorized Document

9.0 STUDY CALENDAR S0016, "A Phase III Trial of CHOP vs CHOP + Rituximab vs. CHOP + Iodine-131-Labeled Monoclonal Anti-B1 Antibody (Tositumomab) for Treatment of Newly Diagnosed Follicular Non-Hodgkin's Lymphomas"

9.3 STUDY CALENDAR: S0016 ARM 3- CHOP + I-131-tositumomab

								£			Ω	Ω	√
		Cycle 1	Cycle 2	Cycle 3	Cycle 4	Cycle 5	Cycle 6	Re-Eval	Antibody treatment				
**REQUIRED STUDIES**	**PRE**	**Day**	**Day**	**Day**	**Day**	**Day**	**Day**	**Day**	**Day**	**Day**	**Day**	**Day**	**Follow**
	**STUDY**	**1**	**22**	**43**	**64**	**85**	**106**	**133**	**134**	**141**	**200**	**365**	**Up**
**PHYSICAL**													
History & Physical Exam	X		X	X	X	X	X	X			X	X	X
Weight & Performance Status	X							X			X	X	X
Tumor Assessment	X							X			X*f*	X*f*	X*f*
Toxicity Notation		X	X	X	X	X	X	X	X	X	X	X	X
**LABORATORY**													
CBC, Platelets & Differential	X		X	X	X	X	X	X			X	X	X
Serum Creatinine	X ß		X	X	X	X	X	X					X
Bilirubin	X ß		X	X	X	X	X	X					X
LDH	X ß							X					X
ß2 Microglobulin	X												
Immunophenotyping (CD20)	X												
Thyroid Stimulating Hormone (TSH)	X ß							X			X	X	X
Urinalysis	X ß							X					
Uric acid	X ß							X					
SGOT and/or Alk Phosphatase	X ß							X					X
Materials for pathology review #	X												
Bone marrow asp./biopsy	X							X			X*	X	
GlaxoSmithKline approval	X												
GlaxoSmithKline training session						X							
Bone marrow submission for bcl2 testing ¢	X											X	
Serum for HAMA testing Δ								X			X	X	X Day 596
**X-RAYS AND SCANS**													
Chest X-Ray or Chest CT *f*	X*f*										X*f*	X*f*	X*f*
CT scan: abdomen, pelvis	X										X	X	X
EKG	X ß												
MUGA or 2-d ECHO ∑	X												
**TREATMENT (See Section 7.5) π**													
Cyclophosphamide		X	X	X	X	X	X						
Doxorubicin		X	X	X	X	X	X						
Vincristine		X	X	X	X	X	X						
Prednisone (see Section 7.3)		X	X	X	X	X	X						
SSKI, Lugol's, Potassium Iodide §								X §	X §	X §			
Unlabeled anti-B1 antibody									X	X			
I-131 anti-B1 dosimetric infusion									X				
Whole Body Dosimetry ¶									X ¶	X ¶			
I-131 anti-B1 therapeutic infusion ¥										X ¥			

NOTE: Data submission forms are found in Section 18.0. Forms submission guidelines may be found in Section 14.0.

f The same scanning technique as baseline must be used to allow uniformity of results.
\# See Section 12.0.
Ω Restaging must occur on Day 200 and Day 365 after initiation of CHOP. The same assessments that were used at baseline should be used for restaging.
√ Patients removed from protocol treatment for any reason will be evaluated at that time by repeating CBC with platelets, LDH, creatinine, liver enzymes, CT of chest, abdomen and pelvis, and all pre-treatment scans to evaluate disease. Follow-up evaluations will include all restaging tests (except CT of chest, abdomen and pelvis) and will occur every 6 months for the first two years, then annually thereafter. The CT of chest, abdomen and pelvis will occur every 6 months for the first two years, then annually for seven years or until relapse.
£ Re--evaluation will occur no less than 4 and no greater than 8 weeks after completion of the sixth cycle of CHOP (see Section 7.5b).
π Patients with progressive disease at any time will discontinue protocol treatment (see Section 10.2f).
ß These tests are suggested at pre-study for Good Medical Practices (see Section 7.1), but <u>must</u> be obtained as listed above for follow-up toxicity assessment.
∑ Required if clinically indicated (see Section 5.19).
* Repeat if initially abnormal
§ SSKI, Lugol's or Potassium Iodide will be administered at least 24 hours prior to the dosimetric dose and will continue for 14 days following the last infusion of radiolabeled anti B1-antibody.
¶ Three whole body scans will be done: the first on Day 1 after the dosimetric infusion (projected to be Day 134 of the protocol treatment), the second on either Day 3, 4, or 5 after the dosimetric infusion and the third on either Day 7 or 8 after the dosimetric infusion (see Section 7.5c.2v).
¥ The therapeutic dose is to be given 7 days after the administration of the dosimetric dose (may be delayed but no longer than 14 days after the dosimetric dose). **A CBC, platelets and differential should be checked weekly after the therapeutic infusion until the patient experiences a nadir and recovers from the nadir (6-8 weeks).**
¢ See Section 15.0.
Δ See Section 15.5 for collection and submission instructions.

SWOG Authorized Document

10.0 CRITERIA FOR EVALUATION AND ENDPOINT DEFINITIONS

10.1 **Measurability of Lesions**:

a. **Measurable Disease:** Lesions that can be accurately measured in two dimensions by CT, MRI, medical photograph (skin or oral lesion), plain x-ray, or other conventional technique and a greatest transverse diameter of 1 cm or greater; or palpable lesions with both diameters ≥ 2 cm. **Note**: CT scans remain the standard for evaluation of nodal disease.

b. **Non-measurable Disease:** All other lesions including unidimensional lesions, lesions too small to be considered measurable, pleural or pericardial effusion, ascites, bone disease, leptomeningeal disease, lymphangitis, pulmonitis, abdominal masses not confirmed or followed by imaging techniques or disease documented by indirect evidence only (e.g., lab values).

10.2 **Objective Disease Status:** Objective status is to be recorded at each evaluation. All measurable lesions up to a maximum of 6 lesions (largest) should be identified as target lesions at baseline. If there are more than 6 measurable lesions the remaining will be identified as non-target lesions and included as non-measurable disease. The 6 lesions should be selected according to the following features: they should be from disparate regions of the body as possible and they should include mediastinal and retroperitoneal areas of disease if these sites have measurable lesions.

Measurements must be provided for target lesions, while presence or absence must be noted for non-target measurable and non-measurable disease.

a. **Complete Response (CR):** Complete disappearance of all measurable and non-measurable disease with the exception of nodes for which the following must be true: for patients with at least one measurable lesion and all nodal masses > 1.5 cm in greatest transverse diameter (GTD) at baseline must have regressed to ≤ 1.5 cm in GTD and all nodal masses ≥ 1 cm and ≤ 1.5 cm GTD must have regressed to < 1.0 cm in GTD or they must have reduced by 75% in sum of products of greatest diameters (SPD). No new lesions. Spleen and other previously enlarged organs must have regressed in size and must not be palpable. If bone marrow was positive at baseline, it must be negative based on biospy and aspirate at same site. Normalization of markers. (e.g., LDH definitely assignable to NHL). All disease must be assessed using the same technique as baseline.

b. **Complete Response Unconfirmed (CRU):** For patients who do not qualify for CR. Complete disappearance of all measurable and non-measurable disease, regressed, non-palpable spleen and other previously enlarged organs, except with one or more of the following features: 1) all residual nodal masses > 1.5cm in GTD at baseline reduced by 75% in SPD or 2) bone marrow indeterminate. No new lesions. All disease must be assessed using the same technique as baseline.

c. **Partial Response (PR):** Applies to patients with at least one measurable lesion that do not quality for a CR or CRU. A 50% decrease in the SPD for up to six identified dominant lesions identified at baseline. No new lesions and no increase in the size of liver or spleen or other nodes. Spleenic and hepatic nodules must have regressed in size by at least 50% in SPD. All disease must be assessed using the same technique as baseline.

d. **Stable:** Does not qualify for CR, CRU, PR, Relapsed/Progressive Disease. All disease must be assessed using the same technique as baseline.

CLOSED EFFECTIVE 09/15/2008

SWOG Authorized Document

e. **Relapsed Disease:** If a (CR,CRU) was achieved at a previous assessment, a 50% increase in the SPD of target measurable lesions over the smallest sum observed (over baseline if no decrease during therapy) or 50% increase in the GTD of any node greater than 1cm in shortest axis using the same techniques as baseline. Unequivocal progression of non-measurable disease in the opinion of the treating physician (an explanation must be provided). Appearance of a new lesion/site. Death due to disease without prior documentation of progression.

f. **Progressive Disease:** If a (CR,CRU) was not achieved at a previous assessment, a 50% increase in the SPD of target measurable lesions over the smallest sum observed (over baseline if no decrease during therapy) using the same techniques as baseline. Appearance of a new lesion/site. Unequivocal progression of non-measurable disease in the opinion of the treating physician (an explanation must be provided). Death due to disease without prior documentation of progression.

g. **Assessment inadequate, objective status unknown**: Progression has not been documented and one or more target lesions or other sites of disease have not been assessed or inconsistent methods of assessment were used.

Notes: Bone marrow status is evaluated as follows:

Positive: Unequivocal cytological or architectural evidence of malignancy.

Negative: No aggregates or only a few well-circumscribed lymphoid aggregates.

Indeterminate: Does not qualify for either positive or negative status. Note this typically consists of increased number or size of aggregates without cytological or architectural atypia.

10.3 **Best Response:**

a. CR: One objective status of CR documented before relapse.

b. CRU: One objective status of CRU documented before relapse but not qualifying as a CR.

c. PR: One objective status of PR documented before progression but not qualifying as a CR or CRU.

d. Stable: At least one objective status of stable documented at least 6 weeks after registration, not qualifying as anything else above.

e. Increasing Disease: Objective status of progression within 12 weeks of registration not qualifying as anything else above.

f. Inadequate assessment, response unknown: Progression greater than 12 weeks after registration and no other response category applies.

CLOSED EFFECTIVE 09/15/2008

SWOG Authorized Document

10.4 **Performance Status**: Patients will be graded according to the Zubrod performance status scale.

**POINT**	**DESCRIPTION**
0	Fully active, able to carry on all pre-disease performance without restriction.
1	Restricted in physically strenuous activity but ambulatory and able to carry out work of a light or sedentary nature, e.g., light housework, office work.
2	Ambulatory and capable of self-care but unable to carry out any work activities; up and about more than 50% of waking hours.
3	Capable of limited self-care, confined to bed or chair more than 50% of waking hours.
4	Completely disabled; cannot carry on any self-care; totally confined to bed or chair.

10.5 **Progression-Free Survival**: From date of registration to date of first observation of progressive disease (as defined in Section 10.2f) or death due to any cause.

10.6 **Time to Death:** From date of registration to date of death due to any cause.

11.0 STATISTICAL CONSIDERATIONS (NOTE: DUE TO PERMANENT CLOSURE OF CHOP ONLY, ARM 1, THE STATISTICAL CONSIDERATIONS HAVE BEEN RE-WRITTEN, EFFECTIVE 12/15/02)

11.1 Based on **S9800** and S9911 the anticipated accrual rate to the two arm study is 9-10 patients per month.

11.2 The primary objective of this trial is to compare CHOP/rituximab versus CHOP/tositumomab in terms of progression-free survival. Based on S9800 pilot data we assume that the hazard rate for CHOP/rituximab is approximately .175 (approximately 2 year progression-free survival rate of 70%). Approximately 500 eligible patients randomized over 4.5 years with 2 additional years of follow-up will be required to have power of .86 to detect a hazard ratio of conventional therapy to experimental therapy arm of 1.50 based on a one-sided .025 level stratified logrank test and assuming exponential progression-free survival distributions. Analysis of treatment differences will be adjusted (stratified) for the design specified stratification factor (serum beta 2 microglobulin) used in the patient randomization (see Section 6.0). A sample size of 250 per arm is sufficient to estimate the response rate or any given toxicity to within 6% for each regimen.

11.3 The proportion of patients testing HAMA positive will estimated at Days 133, 200, 365, and 596. Assuming samples are available on 100 patients, and given historical data showing HAMA rates are generally less than 20% at 18 months, then the fraction of patients that are HAMA positive can be estimated to at least ± .08 (95% confidence interval).

11.4 This study will be monitored throughout accrual and follow-up periods by the Southwest Oncology Group Data and Safety Monitoring Committee (DSMC). In addition to monitoring by the DSMC, formal interim analyses will be done after 50% of eligible patients have been randomized and again after 75% of the eligible patients have been randomized. Evidence to suggest early termination of the study at the time of an interim analysis would be if the null hypothesis of no difference, or the alternative of a hazard ratio of conventional therapy to experimental therapy of 1.50 progression-free survival were rejected at the .0025 level. The actual decision to terminate the study early will be made by the DSMC, and will take into consideration overall survival, progression-free survival and other factors such as toxicities and complications. If the study is not terminated early, reporting of results at the time closure will be considered if the null hypothesis of no difference, or the alternative of a hazard ratio of conventional therapy to experimental therapy of 1.5 for progression-free survival were rejected at the .0025 level. If the study is not terminated early or not reported early due to not rejecting the hypothesis tests at the time closure, the final analysis on progression-free survival using a one-sided .021 level stratified logrank test will be completed approximately 2 years after study closure.

CLOSED EFFECTIVE 09/15/2008

12.0 DISCIPLINE REVIEW

12.1 Pathology Review for Southwest Oncology Group and CALGB Institutions:

All patients registered to this study will undergo pathology review. The purpose of this review is to verify the histologic diagnosis of follicular non-Hodgkin's lymphoma (Grade I, II, or III) and that patients are CD20 positive. Any excess diagnostic tissue will be retained by the Southwest Oncology Group (unless the patient denies consent)

12.2 All pathology submissions for patients registered on this study by Southwest Oncology Group institutions and affiliates must be entered and tracked using the SWOG Online Specimen Tracking System. Southwest Oncology Group members may log on to the Specimen Tracking System via the CRA Workbench (http://gill.crab.org/txwb/logon.asp) using their SWOG roster ID numbers and passwords.

In the online Specimen Tracking System, laboratory ID numbers are used to identify the laboratories to which specimens are shipped. The laboratory ID number for this study may be found listed next to the laboratory name in Section 12.3 below.

ALL SPECIMENS MUST BE LOGGED VIA THIS SYSTEM: THERE ARE NO EXCEPTIONS.

To report technical problems with Specimen Tracking, such as database errors or connectivity issues, please send an email to technicalquestion@crab.org. For procedural help with logging and shipping specimens, there is an introduction to the system on the Specimen Tracking main page (http://dnet.crab.org/SpecTrack/Documents/SpecTPrimer-Insts.pdf); or contact the Data Operations Center at 206/652-2267 to be routed to the Data Coordinator for further assistance.

12.3 Pathology materials are to be submitted to within 30 days of registration to:

Lab #2: SWOG Lymphoma Repository - University of Arizona
Arizona Health Science Center
Department of Pathology, Room 5211, Box 245043
1501 N. Campbell Avenue
Tucson, AZ 85724-5043

Contact: Yvette Frutiger/Lisa M. Rimsza, M.D.
Phone: 520/626-7477
Fax: 520/626-6081
Email: frutiger@email.arizona.edu

12.4 The following materials are to be submitted for review:

a. One representative H&E section from each block of the original diagnostic specimen. (Note: Needle aspirates are not adequate for this submission. Consult with Dr. Rimsza's laboratory if adequacy of specimen is in question.)

b. Either 12 unstained slides or a paraffin block from the representative diagnostic specimen. If a block is sent, then the tissue will be conserved (no more than 12 additional cuts will be made).

c. One copy of pathology report.

12.5 Failure to submit a registered patient's pathology materials for pathology will make the patient ineligible.

13.0 REGISTRATION GUIDELINES

13.1 Patients must be registered prior to initiation of treatment (no more than one working day prior to planned start of treatment).

CLOSED EFFECTIVE 09/15/2008

SWOG Authorized Document

13.2 For either method of registration, the individual registering the patient must have completed the appropriate Southwest Oncology Group Registration Form. The completed form must be referred to during the registration but should not be submitted as part of the patient data.

The individual registering the patient must also be prepared to provide the treating institution's name and ID number in order to ensure that the current (within 365 days) date of institutional review board approval for this study has been entered into the data base. Patients will not be registered if the IRB approval date has not been provided or is > 365 days prior to the date of registration.

13.3 Southwest Oncology Group Registration Procedures

a. You may register patients from Member, CCOP and approved Affiliate institutions to a therapeutics study using the SWOG Registration program. To access the Registration program go to the SWOG Web site (http://swog.org) and click on the *Logon* link to go to the SWOG Members Area logon page (https://swog.org/visitors/logon.asp). This Web program is available at any time except for periods listed **under *Down Times*.** Log on as an Individual User using your SWOG Roster ID Number and individual web user password. Help for the logon process may be found at https://swog.org/visitors/logonhelp.asp. After you have logged on, click on the *Clinical Trials* link and then the *Patient Reg* link to go to the Entry Page for the Patient Registration program. If you are a Registrar at an institution with Internet access you are encouraged to register this way. For new users, the link to a "Starter Kit" of help files may be found by clicking on **Starter Kit link at the logon page.**

To register a patient the following must be done (in order):

1. You are entered into the Southwest Oncology Group Roster and issued a SWOG Roster ID Number,

2. You are associated as an investigator or CRA/RN to the institution where a registration is occurring, and

3. You are granted permission to use the Patient Registration program at that institution.

For assistance with points 1 and 2 call the SWOG Operations Office at 210/614-8808. For point 3 you must contact your Web User Administrator. Each SWOG institution has one or more Web User Administrators who may set up Web Users at their institution and assign permissions and passwords to these users. For other password problems or problems with the Patient Registration program, please e-mail webreghelp@crab.org. Include your name, Roster ID Number, and telephone number, when the problem occurred, and exactly what you were doing.

b. If the Web Reg program is not used, the registration must be done by phone.

Member, Affiliate and CCOP Institutions

Registration by phone of patients from member, affiliate and CCOP institutions must be done through the Southwest Oncology Group Data Operations Center in Seattle by telephoning 206/652-2267, 6:30 a.m. to 4:00 p.m. Pacific Time, Monday through Friday, excluding holidays.

CLOSED EFFECTIVE 09/15/2008
SWOG Authorized Document

13.4 CALGB Institutions Registration Procedures:

Prior to registering their first patient to this protocol, CALGB investigators must submit a S0016 study specific FDA 1572 form and a curriculum vitae (CV) to the Southwest Oncology Group Operations Office at the following address:

Southwest Oncology Group Operations Office
14980 Omicron Drive
San Antonio, TX 78245-3217

Each CALGB investigator must indicate their group affiliation ("CALGB") and CALGB roster ID number along with their name on the form. Subinvestigators may be listed on a single form FDA 1572 signed by the principal investigator. However, a CV from every investigator, including subinvestigators, must be submitted.

Registration/Randomization will be accepted through institutions with direct registration privileges. Confirm eligibility criteria (see Section 5.0). Call the CALGB Registrar (919/286-4704, Monday - Friday, 9:00 A.M. - 4:30 P.M. Eastern Time) with the information contained in Sections 5.1 - 5.25. In addition, the CALGB Registrar will request:

- Your name
- Diagnosis, Date of Diagnosis
- Stratification Factors (see Section 6.0)
- List of prior CALGB protocols
- Date of most recent Institutional Review Board approval (within 365 days)

The CALGB Data Management Center will then contact the Southwest Oncology Group Data Operations Center to register and randomize the patient. The CALGB Registrar will contact the institution to relay the appropriate treatment assignment. The Southwest Oncology Group Data Operations Center also will forward a confirmation of registration to the CALGB Data Management Center for routing to the participating institution. *The Main Member Institution will receive the Confirmation of Registration.* Please check for errors. Submit corrections in writing to CALGB Data Management Center, First Union Plaza, Suite 340, 2200 West Main Street, Durham, NC 27705.

13.5 For either method of registration, exceptions to Southwest Oncology Group registration policies will not be permitted.

a. Patients must meet all eligibility requirements.

b. Institutions must be identified as approved for registration.

c. Registrations may not be cancelled.

d. Late registrations (after initiation of treatment) will not be accepted.

13.6 CTSU Institutions (not aligned with SWOG or CALGB): Institutions participating in the Cancer Trials Support Unit (CTSU), please refer to Appendix 19.6.

CLOSED EFFECTIVE 09/15/2008

SWOG Authorized Document

14.0 DATA SUBMISSION SCHEDULE

14.1 Data must be submitted according to the protocol requirements for **ALL** patients registered, whether or not assigned treatment is administered, including patients deemed to be ineligible. Patients for whom documentation is inadequate to determine eligibility will generally be deemed ineligible.

14.2 Master forms are included in Section 18.0 and (with the exception of the sample consent form and the Registration Form) must be submitted to the Data Operations Center in Seattle. Data from approved SWOG institutions must be submitted on-line via the Web; see Section 14.3a for details. Exceptions to online data submission are patient-completed (e.g. Quality of Life) forms and source documents (e.g. pathology/operative/lab reports).

14.3 Data Submission Procedures

a. Southwest Oncology Group institutions must submit data electronically via the Web by using the SWOG CRA Workbench. To access the CRA Workbench, go to the SWOG Web site (http://swog.org) and logon to the Members Area. After you have logged on, click on the *CRA Workbench* link to access the home page for CRA Workbench website. Next, click on the *Data Submission* link and follow the instructions. For new users, the link to a "Starter Kit" of help files may be found by clicking on the **Starter Kit** link at the Members' logon page.

To submit data via the web the following must be done (in order):

1. You are entered into the Southwest Oncology Group Roster and issued a SWOG Roster ID Number,
2. You are associated as an investigator or CRA/RN at the institution where the patient is being treated or followed, and
3. Your Web User Administrator has added you as a web user and has given you the appropriate system permissions to submit data for that institution.

For assistance with points 1 and 2 call the Operations Office at 210/614-8808. For point 3, contact your local Web User Administrator (refer to the "Who is my Web User Administrator?" function on the swog.org Members logon page). For other difficulties with the CRA Workbench, please email *technicalquestion@crab.org*.

b. If you need to submit data that are not available for online data submission, the only alternative is via facsimile. Should the need for this occur, institutions may submit data via facsimile to 800/892-4007 or 206/342-1680 locally. Please do not use cover sheet for faxed data.

c. CALGB Institutions: CALGB participants should submit data forms as listed in these sections at the required intervals to the mailing address above. Include the Southwest Oncology Group protocol number and patient number as well as the CALGB study number and patient number on each piece of data submitted.

d. CTSU Institutions (not aligned with SWOG or CALGB): Institutions participating through the Cancer Trials Support Unit (CTSU), please refer to Appendix 19.6.

CLOSED EFFECTIVE 09/15/2018

SWOG Authorized Document

14.4 WITHIN 14 DAYS OF REGISTRATION:

Submit a copy of the following:

a. **S0016** Non-Hodgkin's Lymphoma (Follicular) Prestudy Form (Form #32770)

b. Lymphoma Baseline Tumor Assessment Form (Form #48010)

c. Pathology Report confirming histology and CD20 antigen expression

d. Submit bone marrow for bcl2 assessment to Dr. Rita Braziel per Section 15.4.

e. Completed Section 5.0 of the protocol.

14.5 WITHIN 30 DAYS OF REGISTRATION:

Submit histopathologic materials along with a copy of the pathology reports to Dr. Rimsza (see Section 12.0).

14.6 FOR PATIENTS ON THE I-131 TOSITUMOMAB ARM (ARM 3), AFTER EVERY CYCLE OF CHOP AND AFTER COMPLETION OF I-131 TOSITUMOMAB TREATMENT:

Submit the **S0016** Tositumomab Treatment Form (Form #62442), the **S0016** CHOP/R-CHOP Treatment Form (Form #43596) and the **S0016** Adverse Event Form (Form #41405).

14.7 AT DAYS 133, 200, 365, AND 596:

Submit serum specimens for HAMA testing as specified in Section 15.5a. For patients enrolled on this study before HAMA testing became mandatory, please see Section 15.5b for timing of serum collection.

14.8 AT PRESTUDY, DAYS 133, 200, 365, AND ANNUALLY AT YEARS 2, 3, 4 AND 5:

Submit **S0016** TSH Reporting Form (Form #55034).

14.9 FOR PATIENTS ON THE R-CHOP ARM (ARM 2), AFTER EVERY CYCLE OF R-CHOP:

Submit the **S0016** CHOP/R-CHOP Treatment Form (Form #43596) and the **S0016** Adverse Event Form (Form #41405).

14.10 FOR EITHER ARM, 3 MONTHS AFTER REMOVAL FROM PROTOCOL TREATMENT:

Submit the **S0016** Adverse Event Form (Form #41405).

14.11 WITHIN 14 DAYS OF FOLLOW-UP RESPONSE ASSESSMENT:

Submit the Lymphoma Follow-Up Tumor Assessment Form (Form #59058).

14.12 EVERY SIX MONTHS AFTER OFF TREATMENT FOR TWO YEARS AND THEN ANNUALLY THEREAFTER:

Submit a copy of the Follow-up Form (Form #8114).

14.13 AT ONE YEAR FOLLOW-UP:

Submit marrow for bcl2 assessment to Dr. Rita Braziel per Section 15.4.

14.14 WITHIN 14 DAYS OF PROGRESSION OR RELAPSE:

Submit a copy of the Follow-Up Form (Form #8114).

14.15 WITHIN 14 DAYS AFTER DISCONTINUATION OF ALL PROTOCOL TREATMENT:

Submit a copy of the Off Treatment Notice (Form #22204).

14.16 WITHIN FOUR WEEKS OF KNOWLEDGE OF DEATH:

Submit a copy of the Follow-Up Form (Form #8114) (If death occurs after off treatment) and a copy of the Notice of Death (Form #1821).

CLOSED EFFECTIVE 09/15/2008

SWOG Authorized Document

14.17 <u>FOR PATIENTS ON THE R-CHOP ARM (ARM 2) OF THE STUDY, SUBMIT THE **S0016** LABORATORY AND IMAGING FORM (FORM #33880) FOR DATA RECEIVED AT THE FOLLOWING TIMES</u>:

Baseline, Days 29, 50, 71, 92, 113, 141, 200 and 365, 1.5 years after registration and at Years 2, 2.5, 3 and 4 after registration.

14.18 <u>FOR PATIENTS ON THE I-131 TOSITUMOMAB ARM (ARM 3) OF THE STUDY, SUBMIT THE **S0016** LABORATORY AND IMAGING FORM (FORM #33880) FOR DATA RECEIVED AT THE FOLLOWING TIMES</u>:

Baseline, Days 22, 43, 64, 85, 106, 133, 200 AND 365, 1.5 years after registration and at Years 2, 2.5, 3 and 4 after registration.

CLOSED EFFECTIVE 09/15/2008

SWOG Authorized Document

15.0 SPECIAL INSTRUCTIONS

15.1 Southwest Oncology Group and CALGB Institutions: Instructions for obtaining institutional approval and training prior to registration and I-131 treatment of patients on **S0016**:

Prior to registration of any patient to **S0016**, the registering institution must be approved by GlaxoSmithKline for the delivery of I-131 therapy. The **S0016** Site Contact Information Form (Appendix 19.4) and radioactive materials license must be faxed to GlaxoSmithKline at the number listed on the bottom of the form. If approved, GlaxoSmithKline will send the institution an approval notice.

Once a patient is randomized to Arm 3, GlaxoSmithKline will contact the institution to provide assistance and to arrange for an on-site training session for Iodine I-131 therapy (if required or requested). Training may occur at any time after the 5th cycle of CHOP, but it must be completed before the patient can receive the Iodine-131 Anti-B1 Antibody therapy.

NOTE: GlaxoSmithKline approval must be received prior to the first patient registered to this study at any one institution. A protocol specific training is required for all institutions with patients randomized to Arm 3. In addition, members of the treatment team are required to participate in the general Bexxar training program. Training must be completed prior to placing drug orders for I-131 treatment. Drug orders cannot be processed without this requirement being met.

15.2 Institutions are **required** to submit the following specimens:

a. Bone marrow: These specimens are being collected in order to compare the molecular remission rates by measuring clonal t(14:18)/bcl-2 rearrangements in the bone marrow at baseline and at one year post-treatment. Samples will be collected and stored.

b. Serum: These specimens are being collected in order to determine the incidence and time to development of human anti-mouse antibody (HAMA) positivity.

15.3 General Specimen Submission Instructions

a. All submitted specimens must be labeled with the protocol number (**S0016**), SWOG patient number, patient's initials, and date of specimen collection.

b. The Federal Guidelines for Shipment are as follows:

1. The specimen must be wrapped in an absorbable material;

2. The specimen must then be placed in an AIRTIGHT container (like a resealable bag);

3. Pack the resealable bag and specimen in a styrofoam shipping container;

4. Pack the styrofoam shipping container in a cardboard box.

5. The cardboard box must be marked as "BIOHAZARD".

CLOSED EFFECTIVE 09/15/2008

SWOG Authorized Document

c. Specimen Tracking System

All specimen submissions for this protocol must be entered and tracked using the Southwest Oncology Group online specimen tracking system. Southwest Oncology Group Members may log onto the specimen tracking system via the CRA Workbench (https://gill.crab.org/txwb/logon.aspx) using their Southwest Oncology Group roster identification numbers and passwords. First-time non- Southwest Oncology Group users must refer to start-up instructions located at https://gill.crab.org/SpecTrack/. In the online specimen tracking system, laboratory ID numbers are used to identify the laboratories where specimens are to be shipped. The laboratory number for specimen submission is outlined in Section 15.4b.2.

ALL SPECIMENS MUST BE LOGGED VIA THIS SYSTEM; THERE ARE NO EXCEPTIONS.

To report technical problems with Specimen Tracking, such as database errors or connectivity issues, please send an email to technicalquestion@crab.org. For procedural help with logging and shipping specimens, there is an introduction to the system on the Specimen Tracking main page (http://dnet.crab.org/SpecTrack/Documents/SpecTPrimer-Insts.pdf); or contact the Data Operations Center at 206/652-2267 to be routed to the Data Coordinator for further assistance.

15.4 Instructions for submission of bone marrow for correlative studies

a. **Institutions are required to submit bone marrow for t(14:18)/bcl-2 testing.**

b. Directions for collecting, processing, and shipping samples

1. Timing: Using a heparinized needle, collect 2-3 cc of bone marrow into a green-top Vacutainer® (sodium heparin) tube at pre-study and at 1 year follow-up*.

   * Samples should be submitted for all patients that have not progressed.

2. Shipping: Bone marrow specimens should be shipped **at room temperature by overnight delivery** (the day they are obtained), to the following laboratory:

   Lab #*151* Flow Cytometry Laboratory
   Oregon Health Sciences University
   Department of Pathology, L471
   3181 SW Sam Jackson Park Road
   Portland, OR 97201

   Contact: Rita Braziel, M.D.
   Phone: 503/494-2315
   Fax: 503/494-0731
   Email: braziel@ohsu.edu

   i. **A copy of the Shipment Packing List produced by the Specimen Tracking system should be printed and sent with the specimens in a separate resealable bag.**

   ii. Samples should be shipped overnight Monday – Thursday.

CLOSED EFFECTIVE 09/15/2008

SWOG Authorized Document

iii. Weekend shipping (arrival on Saturday): Samples will be accepted on Saturdays if the bone marrow evaluation cannot be postponed until Monday for clinical reasons however, the OHSU Flow Cytometry Laboratory MUST be contacted at 503/494-2302 at least 2 days before shipping the sample so that special mailing instructions for specimens can be obtained. Indicate Saturday delivery on the overnight mailing label.

15.5 Instructions for submission of serum for HAMA testing

a. Institutions are **<u>required</u>** to submit serum for <u>all patients</u> for HAMA testing. (For a description of HAMA assay methods, see Section 19.7.)

b. Timing of serum collection

1. HAMA assessments will be performed by drawing serum on:

Day	Arm 2	Arm 3
133 (baseline)	Prior to 5th rituximab dose	Prior to Iodine-131 Anti-B1 dosimetric dose
200	~9 weeks after the 6th (final) rituximab dose	~9 weeks after the Iodine-131 Anti-B1 dosimetric dose
365	~8 months after the 6th (final) rituximab dose	~8 months after the Iodine-131 Anti-B1 dosimetric dose
596	~15 months after the 6th (final) rituximab dose	~15 months after the Iodine-131 Anti-B1 dosimetric dose

2. Patients enrolled on this study before HAMA testing became mandatory will have serum drawn for HAMA testing at their next clinic visit and then will resume the schedule above (if applicable). Patients who are beyond Day 596 at the time of activation of the amendment requiring HAMA submission will have a single serum specimen submitted at the time of their next scheduled assessment, regardless of how long that evaluation date is after protocol therapy.

c. Serum processing and packaging instructions

1. Collect blood into a 10 mL red-top Vacutainer® (no anticoagulant).

2. Allow blood to clot for 30 - 60 minutes at room temperature. Centrifuge the sample for 10 - 15 minutes at 2,800 rpm and remove the serum with a transfer pipette.

3. Transfer the serum to a plastic leak-proof cryovial and freeze at -20°C before sending via overnight express to the SWOG Lymphoma Serum Repository.

4. **Label cryovial with: 1) Patients initials; 2) SWOG I.D. number; 3) SWOG Study Number; 4) Date collected**.

5. Wrap the cryovial in an absorbable material and place the specimen in an <u>airtight container with absorbent sheet</u> according to the federal guidelines for shipment (see Section 15.3b).

6. Pack cryovial in a Styrofoam shipping container with dry ice. Place the Styrofoam container in a cardboard box. Label as “biohazard”. Include sufficient packing materials to adequately secure the tube for shipment.

CLOSED EFFECTIVE [illegible]2008

SWOG Authorized Document

d. Serum shipping instructions

1. A copy of the shipment packing list produced by the SWOG Online Specimen Tracking system should be printed and sent with the specimens in a separate resealable bag.

2. Ship the serum the same day as collected via overnight express to:

Lab #2 SWOG Lymphoma Repository – University of Arizona
Arizona Health Science Center
Department of Pathology, Room 5211, Box 245043
1501 N. Campbell Ave.
Tucson, AZ 85724-5043

Contact: Yvette Frutiger/Lisa M. Rimsza, M.D.
Phone: 520/626-7477
Fax: 520/626-6081
Email: frutiger@email.arizona.edu

NOTE: DO NOT SEND SERUM ON FRIDAY. SEND MONDAY THROUGH THURSDAY ONLY!

15.6 Instructions for submission of snap frozen tissue and serum for banking (SWOG and CALGB institutions only).

a. **Institutions are encouraged to seek additional patient consent for submission of snap frozen tissue and serum for banking.** (The **S0016** model informed consent document includes participant consent for both **SWOG-8819** [Central Lymphoma Repository Tissue Procurement Protocol] and **SWOG-8947** [Central Lymphoma Serum Repository Protocol]. See Section 18.1.)

b. Directions for collecting and shipping samples for banking.

Refer to SWOG protocols, **SWOG-8819** (Central Lymphoma Repository Tissue Procurement Protocol) and **SWOG-8947** (Central Lymphoma Serum Repository Protocol) for details.

16.0 ETHICAL AND REGULATORY CONSIDERATIONS

The following must be observed to comply with Food and Drug Administration regulations for the conduct and monitoring of clinical investigations; they also represent sound research practice:

Informed Consent

The principles of informed consent are described by Federal Regulatory Guidelines (Federal Register Vol. 46, No. 17, January 27, 1981, part 50) and the Office for Protection from Research Risks Reports: Protection of Human Subjects (Code of Federal Regulations 45 CFR 46). They must be followed to comply with FDA regulations for the conduct and monitoring of clinical investigations.

Institutional Review

This study must be approved by an appropriate institutional review committee as defined by Federal Regulatory Guidelines (Ref. Federal Register Vol. 46, No. 17, January 27, 1981, part 56) and the Office for Protection from Research Risks Reports: Protection of Human Subjects (Code of Federal Regulations 45 CFR 46).

CLOSED EFFECTIVE 09/15/2008

SWOG Authorized Document

Drug Accountability

For each drug supplied for a study, an accountability ledger containing current and accurate inventory records covering receipt, dispensing, and the return of study drug supplies must be maintained. Drug supplies must be kept in a secure, limited access storage area under the recommended storage conditions. During the course of the study, the following information must be noted on the accountability ledger; the identification code of the subject to whom drug is dispensed, the date(s) and quantity of drug dispensed to the subject, and the date(s) and quantity of drug returned by the subject; subjects should return empty containers to the investigator, with the return noted on the ledger. These Accountability Forms must be readily available for inspection and are open to FDA inspection at any time.

Monitoring

This study will be monitored by the Clinical Data Update System (CDUS) version 2.0. Cumulative CDUS data will be submitted quarterly to CTEP by electronic means. Reports are due January 31, April 30, July 31 and October 31.

Adverse Event Reporting For CALGB Institutions:

CALGB Institutions will report adverse events, both written and telephone reports, directly to the Southwest Oncology Group Operations Office and the NCI as specified in the following reporting institutions.

16.1 Adverse Event Reporting Requirements

a. Purpose

Adverse event data collection and reporting, which are required as part of every clinical trial, are done to ensure the safety of patients enrolled in the studies as well as those who will enroll in future studies using similar agents. Adverse events are reported in a routine manner at scheduled times during a trial. (Directions for routine reporting are provided in Section 14.0.) Additionally, certain adverse events must be reported in an expedited manner to allow for more timely monitoring of patient safety and care. The following guidelines prescribe expedited adverse event reporting for this protocol. See also **Appendix 19.5** for general and background information about expedited reporting.

b. Reporting methods

This study requires that expedited adverse event reporting use the NCI's Adverse Event Expedited Reporting System (AdEERS). The NCI's guidelines for AdEERS can be found at http://ctep.cancer.gov. An AdEERS report must be submitted to the Southwest Oncology Group Operations Office by one of the following methods:

- Electronically submit the report via the AdEERS Web-based application located at http://ctep.cancer.gov, or
- **Only if submitting electronically is not possible**, fax the completed NCI Adverse Event Expedited Report – Single Agent or Multiple Agents – paper template, located at http://ctep.cancer.gov, to 210/614-0006.

CLOSED EFFECTIVE 09/15/2008

SWOG Authorized Document

c. When to report an event in an expedited manner

Some adverse events require 24-hour notification (refer to Table 16.1) via AdEERS. When Internet connectivity is disrupted, a 24-hour notification is to be made to CTEP by telephone at 301-897-7497. Once Internet connectivity is restored, a 24-hour notification phoned in, must be entered electronically into AdEERS by the original submitter at the site.

When the adverse event requires expedited reporting, submit the report within the number of calendar days of learning of the event specified in Table 16.1 or 16.2, as applicable.

d. Other recipients of adverse event reports

The Operations Office will forward reports and documentation to the appropriate regulatory agencies and drug companies as required.

Adverse events determined to be reportable must also be reported according to local policy and procedures to the Institutional Review Board responsible for oversight of the patient.

e. **Expedited reporting for investigational agents**

Expedited reporting is required if the patient has received at least one dose of the investigational agent(s) as part of the trial. Reporting requirements are provided in Table 16.1. The investigational agent used in Arm 3 of this study is Iodine-131-lableled murine monoclonal anti-B1 antibody (tositumomab). If there is any question about the reportability of an adverse event or if on-line AdEERS cannot be used, please telephone or email the SAE Specialist at the Operations Office, 210/614-8808 or adr@swog.org, before preparing the report.

CLOSED EFFECTIVE 09/15/2008

SWOG Authorized Document

Table 16.1 Phase 2 and 3 Trials Utilizing an Agent under a CTEP IND or Non-CTEP IND: AdEERS Expedited Reporting Requirements for Adverse Events that Occur within 30 Days[1] of the Last Dose of the Investigational Agent Iodine-131-labeled murine monoclonal anti-B1 antibody (tositumomab) in this Study

	Grade 1	Grade 2	Grade 2	Grade 3		Grade 3		Grades 4 & 5[2]	Grades 4 & 5[2]
	Unexpected and Expected	Unexpected	Expected	Unexpected		Expected		Unexpected	Expected
				with Hospitali-zation	without Hospitali-zation	with Hospitali-zation	without Hospitali-zation		
**Unrelated Unlikely**	Not Required	Not Required	Not Required	10 Calendar Days	Not Required	10 Calendar Days	Not Required	10 Calendar Days	10 Calendar Days
**Possible Probable Definite**	Not Required	10 Calendar Days	Not Required	10 Calendar Days	10 Calendar Days	10 Calendar Days	Not Required	24-Hour; 5 Calendar Days	10 Calendar Days

[1] **Adverse events with attribution of possible, probable, or definite that occur greater than 30 days after the last dose of treatment with an agent under a CTEP IND or Non-CTEP IND require reporting as follows:**
AdEERS 24-hour notification (**via AdEERS for CTEP IND agents; via email to *adr@swog.org* for agents in non-CTEP IND studies**) followed by complete report within 5 calendar days for:
- Grade 4 and Grade 5 unexpected events

AdEERS 10 calendar day report:
- Grade 3 unexpected events with hospitalization or prolongation of hospitalization
- Grade 5 expected events

[2] Although an AdEERS 24-hour notification is not required for death clearly related to progressive disease, a full report is required as outlined in the table.

March 2005

Note: All deaths on study require both routine and expedited reporting regardless of causality. Attribution to treatment or other cause must be provided.

- Expedited AE reporting timelines defined:
  - ▶ "24 hours; 5 calendar days" – The investigator must initially report the AE via AdEERS within 24 hours of learning of the event followed by a complete AdEERS report within 5 calendar days of the initial 24-hour report.
  - ▶ "10 calendar days" - A complete AdEERS report on the AE must be submitted within 10 calendar days of the investigator learning of the event.

- Any medical event equivalent to CTCAE grade 3, 4, or 5 that precipitates hospitalization (or prolongation of existing hospitalization) must be reported regardless of attribution and designation as expected or unexpected with the exception of any events identified as protocol-specific expedited adverse event reporting exclusions.

- Any event that results in persistent or significant disabilities/incapacities, congenital anomalies, or birth defects must be reported via AdEERS if the event occurs following treatment with an agent under a CTEP IND.

CLOSED EFFECTIVE 09/13/2008
SWOG Authorized Document

- Use the NCI protocol number and the protocol-specific patient ID assigned during trial registration on all reports.

f. **Expedited reporting for <u>commercial</u> agents**

Commercial reporting requirements are provided in Table 16.2. The commercial agents used in Arms 1, 2, and 3 of this study are **cyclophosphamide, doxorubicin, prednisone, vincristine, and rituximab chimeric anti-CD20mAb (IDEC-C2B8)**. If there is any question about the reportability of an adverse event, please telephone or email the SAE Program at the Operations Office, 210/614-8808 or adr@swog.org, before preparing the report.

Table 16.2. Expedited reporting requirements for adverse events experienced by patients on study Arms 1, 2, and 3 who have received the commercial drugs listed in 16.1f above.

Attribution	Grade 4		Grade 5[a]	
	Unexpected	Expected	Unexpected	Expected
Unrelated or Unlikely			**AdEERS**	**AdEERS**
Possible, Probable, Definite	**AdEERS**		**AdEERS**	**AdEERS**

AdEERS: Indicates an expedited report is to be submitted using the NCI AdEERS system Commercial Drug pathway within 7 working days of learning of the event.

[a] This includes all deaths within 30 days of the last dose of treatment with a commercial agent(s), regardless of attribution. Any death that occurs more than 30 days after the last dose of treatment with a commercial agent(s) and is attributed (possibly, probably, or definitely) to the agent(s) and is not due to cancer recurrence must be reported according to the instructions above.

g. Reporting secondary AML/MDS/ALL

All cases of acute myeloid leukemia (AML) myelodysplastic syndrome (MDS), and acute lymphocytic leukemia (ALL) that occur in patients on NCI-sponsored trials following chemotherapy for cancer must be reported using the NCI/CTEP Secondary AML/MDS/ALL Report Form in lieu of AdEERS. The following supporting documentation must also be submitted within 30 days:

- a copy of the pathology report confirming the AML/MDS/ALL diagnosis; and
- (if available) a copy of the cytogenetics report.

Submit the Report and documentation to:

Investigational Drug Branch
by fax to 301-230-0159

<u>**and**</u>

Southwest Oncology Group
ATTN: SAE Program
4201 Medical Drive, Suite 250
San Antonio, TX 78229

NOTE: If a patient has been enrolled in more than one NCI-sponsored study, the AML/MDS/ALL Report must be submitted for the most recent trial.

CLOSED EFFECTIVE 09/15/2008

SWOG Authorized Document

17.0 BIBLIOGRAPHY

1. Horning SJ. Treatment approaches to the low-grade lymphomas. Blood 83:881-4, 1994.

2. Morrison VA, Peterson BA. Combination chemotherapy in the treatment of follicular low-grade lymphoma. Leuk Lymphoma 10:29-33, 1993.

3. Jones SE, Grozea PN, Miller TP, Van Slyck EJ, Balcerzak SP, Costanzi JJ, Morrison FS, Eyre HJ, Fabian CJ, Dabich L, et al. Chemotherapy with cyclophosphamide, doxorubicin, vincristine, and prednisone alone or with levamisole or with levamisole plus BCG for malignant lymphoma: a Southwest Oncology Group Study. J Clin Oncol 3:1318-24, 1985.

4. McKelvey EM, Gottlieb JA, Wilson HE, Haut A, Talley RW, Stephens R, Lane M, Gamble JF, Jones SE, Grozea PN, Gutterman J, Coltman C, Moon TE. Hydroxyldaunomycin (Adriamycin) combination chemotherapy in malignant lymphoma. Cancer 38:1484-93, 1976.

5. Jones SE, Grozea PN, Metz EN, Haut A, Stephens RL, Morrison FS, Talley R, Butler JJ, Byrne GE, Jr., Hartsock R, Dixon D, Salmon SE. Improved complete remission rates and survival for patients with large cell lymphoma treated with chemoimmunotherapy. A Southwest Oncology Group Study. Cancer 51:1083-90, 1983.

6. Dana BW, Dahlberg S, Nathwani BN, Chase E, Coltman C, Miller TP, Fisher RI. Long-term follow-up of patients with low-grade malignant lymphomas treated with doxorubicin-based chemotherapy or chemoimmunotherapy. J Clin Oncol 11:644-51, 1993.

7. Kantarjian HM, McLaughlin P, Fuller LM, Dixon DO, Osborne BM, Cabanillas F. Follicular large cell lymphoma: analysis and prognostic factors in 62 patients. J Clin Oncol 2:811-9, 1984.

8. Horning SJ, Weiss LM, Nevitt JB, Warnke RA. Clinical and pathologic features of follicular large cell (nodular histiocytic) lymphoma. Cancer 59:1470-4, 1987.

9. Anderson JR, Vose JM, Bierman PJ, Weisenberger DD, Sanger WG, Pierson J, Bast M, Armitage JO. Clinical features and prognosis of follicular large-cell lymphoma: a report from the Nebraska Lymphoma Study Group. J Clin Oncol 11:218-24, 1993.

10. Bartlett NL, Rizeq M, Dorfman RF, Halpern J, Horning SJ. Follicular large-cell lymphoma: intermediate or low grade? J Clin Oncol 12:1349-57, 1994.

11. Velasquez W, Lew D, Miller T, Fisher R. **SWOG-9501**: A phase II trial of a combination of fludarabine and mitoxantrone (FN) in untreated advanced low grade lymphoma: an effective well tolerated therapy. Proc. Am. Soc. Clin. Oncol. 18: 9a (abstract 27), 1999.

12. Gribben JG, Neuberg D, Freedman AS, Gimmi CD, Pesek KW, Barber M, Saporito L, Woo SD, Coral F, Spector N, et al. Detection by polymerase chain reaction of residual cells with the bcl-2 translocation is associated with increased risk of relapse after autologous bone marrow transplantation for B-cell lymphoma. Blood 81:3449-57, 1993.

13. Maloney DG, Liles TM, Czerwinski DK, Waldichuk C, Rosenberg J, Grillo-Lopez A, Levy R. Phase I clinical trial using escalating single-dose infusion of chimeric anti-CD20 monoclonal antibody (IDEC-C2B8) in patients with recurrent B-cell lymphoma. Blood 84:2457-66, 1994.

14. Maloney DG, Grillo-Lopez AJ, White CA, Bodkin D, Schilder RJ, Neidhart JA, Janakiraman N, Foon KA, Liles TM, Dallaire BK, Wey K, Royston I, Davis T, Levy R. Idec-C2B8 (Rituximab) anti-CD20 monoclonal antibody therapy in patients with relapsed low grade non-Hodgkin's lymphoma. Blood: 90: 2188, 1997. PMID: 9310469; UI: 97454394.

CLOSED EFFECTIVE 09/15/2008

SWOG Authorized Document

15. McLaughlin P, Cabanillas F, Grillo-Lopez AJ, Link BK, Levy R, Czuczman M, Heyman MR, Williams M, Jain V, Bence-Bruckler I, Ho AD, Lister J, Rosenberg J, Dallaire BK, Shen D. IDEC-C2B8 anti-CD20 antibody: final report on a phase III pivotal trial in patients with relapsed low-grade or follicular lymphoma. Blood 88 (supp 1):349a, 1996.

16. Solal-Celigny Ph, Salles G, Brousse N, et al. Rituximab as first-line treatment of patients with follicular lymphoma and a low-burden tumor: clinical and molecular evaluation. Blood 94: 631a (abstract 2802) (Suppl 1)

17. Coiffier B, Haioun C, Ketterer N, et al: Rituximab (anti-CD20 monoclonal antibody) for the treatment of patients with relapsing or refractory aggressive lymphoma: a multicenter Phase II study. Blood 92: 1927, 1998

18. Foran JM, Rohatiner AZ, Cunningham D, et al. European phase II study of rituximab (chimeric anti-CD20 monoclonal antibody) for patients with newly diagnosed mantle-cell lymphoma and previously treated mantle-cell lymphoma, immunocytoma, and small B-cell lymphocytic lymphoma. J Clin Oncol 18:317-24, 2000

19. Byrd JC, Waselenko JK, Maneatis TJ, et al. Rituximab therapy in hematologic malignancy patients with circulating blood tumor cells: Association with increased infusion-related side effects and rapid blood tumor clearance. J Clin Oncol 17:791, 1999

20. Czuczman MS, Grillo-López AJ, White CA, Saleh M, Gordon L, LoBuglio AF, Jonas C, Klippenstein D, Dallaire B, Varns C. Treatment of patients with low-grade B-cell lymphoma with the combination of chimeric anti-CD20 monoclonal antibody and CHOP chemotherapy. J Clin Oncol. 17: 268-276, 1999. PMID: 10458242; UI: 99385424.

21. Kaminski MS, Zasadny KR, Francis IR et al. Radioimmunotherapy of B-cell lymphoma with I-131 anti-B1 (anti-CD20) antibody. NEJM 329: 459, 1993

22. Kaminski M, Zasadny K, Francis I, et al. Iodine-131-anti-B1 radioimmunotherapy for B-cell Lymphoma. J Clin Oncol 14:1974, 1996.

23. Kaminski M, Gribbin T, Estes J et al. I-131-Anti-B1 Antibody for previously untreated follicular lymphoma: clinical and molecular remissions. Proc. Am. Soc. Clin. Oncol. 17: 2a, 1998.

24. Prescribing information for the BEXXAR® therapeutic regimen.

25. Reports of human anti-murine antibody (HAMA) after the BEXXAR® therapeutic regimen, GSK medical information letter, BXRADR02.

26. Kricka L. Human anti-animal antibody interferences in immunological assays. Clin Chem, 45(7): 942-56, 1999.

27. Press OW, Eary J, Appelbaum FR, Martin PJ, Badger CC, Nelp WB, Glenn S, Butchko G, Fisher D, Porter B, Matthews D, Fisher L, Bernstein ID. Radiolabeled antibody therapy of B cell lymphomas with autologous bone marrow support. NEJM 324: 1219-1224, 1993.

28. Press O, Eary J, Appelbaum F, et al. Phase II trial of 131I-B1 (anti-CD20) antibody therapy with autologous stem cell transplantation for relapsed B cell lymphomas. Lancet 346: 336, 1995.

29. Knox S, Goris M, Trisler K, et al. Yttrium-90-labeled anti-CD20 monoclonal antibody therapy of recurrent B-cell lymphoma. Clin Cancer Res 2:457, 1996.

30. Witzig TE, White CA, Wiseman GA, et al. Phase I/II trial of IDEC-Y2B8 radioimmunotherapy for treatment of relapsed or refractory CD20-positive B-cell non-Hodgkin's lymphoma. J Clin Oncol 1999 (in press).

CLOSED EFFECTIVE 09/15/2008

SWOG Authorized Document

31. Witzig TE, White CA, Gordon LI et al. Prospective randomized controlled study of Zevalin (Idec-Y2B8) radioimmunotherapy compared to rituximab immunotherapy for B cell NHL: Report of interim results. Blood 94: 631a (abstract 2805) (Suppl 1).

32. O'Donoghue JA. Optimal therapeutic strategies for radioimmunotherapy. Recent Results in Cancer Research 36(10):1910-2. PMID: 7562063; UI: 96007646, 1996.

33. Solal-Celigny P, et al. Recombinant interferon alfa-2b combined with a regimen containing doxorubicin in patients with advanced follicular lymphoma. Groupe d'Etude des Lymphomes de l'Adulte. NEJM 329(22):1608-14. PMID: 8232429; UI: 94049998, 1993.

34. Rohatiner AZS, Gregory W, Peterson B, Smalley R, Solal-Celigny P, Hagenbeek A, Bijnens L, Unterhalt M, Chisesi T, Aviles A, Lister TA. A meta-analysis of randomises trials evaluating the role of interferon as treatment for follicular lymphomas. Proc Am. Soc. Clin. Oncol. 17: 4a (abstract 11), 1998.

35. Demidem A, Lam T, Alas S, Hariharan K, Hanna H, Bonavida B. Chimeric anti-CD20 antibody (IDEC-C2B8) monoclonal antibody sensitizes a B cell lymphoma cell line to cell killing by cytotoxic drugs. Cancer Biotherapy & Radiopharmaceuticals 12:177, 1997.

36. Smith TJ, Ozer H, Miller L, et al. Update of recommendations for the use of hematopoietic colony-stimulating factors: evidence-based clinical practice guidelines. J. Clin. Oncol. 14: 1957, 1996.

CLOSED EFFECTIVE 09/15/2008

SWOG Authorized Document

18.0 MASTER FORMS SET

18.1 Attached are copies of all data forms which must be completed for this study. The model informed consent form is also included, and must be reviewed and approved by the institutional review board prior to registration and treatment of patients on this study.

18.2 Forms to be used for patients treated on this study include:

a. **S0016** Registration Form (Form #3645) (12/15/01); Southwest Oncology Group Registration Form Code Sheet (10/24/06)

b. **S0016** Non-Hodgkin's Lymphoma (Follicular) Prestudy Form (Form #32770) (7/1/07)

c. Lymphoma Baseline Tumor Assessment Form (Form #48010) (10/15/00)

d. **S0016** Tositumomab Treatment Form (Form #62442) (7/1/07)

e. **S0016** CHOP/R-CHOP Treatment Form (Form #43596) (7/1/07)

f. **S0016** Adverse Event Form (Form #41405) (2/15/06)

g. **S0016** TSH Reporting Form (Form #55034) (7/1/07)

h. Off Treatment Notice (Form #22204) (02/01/00)

i. Notice of Death (Form #1821) (02/01/00)

j. Follow-Up Form (Form #8114) (12/15/06)

k. Lymphoma Follow-Up Tumor Assessment Form (Form #59058) (09/01/01)

l. **S0016** Laboratory and Imaging Data Form (Form #33880) (11/3/09)

CLOSED EFFECTIVE 09/15/2008

SWOG Authorized Document

For IRB use only, not to be included in patient information.

This model informed consent form has been reviewed by the DCT/NCI and is the official consent document for this study. Local IRB changes to this document are allowed. (Institutions should attempt to use sections of this document which are in bold type in their entirety.) Editorial changes to these sections may be made as long as they do not change information or intent. If the institutional IRB insists on making deletions or more substantive modifications to the risks or alternatives sections, they may be justified in writing by the investigator and approved by the IRB. Under these circumstances, the revised language, justification and a copy of the IRB minutes must be forwarded to the Southwest Oncology Group Operations Office for approval before a patient may be registered to this study.

Readability Statistics:	Flesch Reading Ease	48.9 (targeted above 55) *(Updated 3/28/07)*
	Flesch-Kincaid Grade Level	11.1 (targeted below 8.5)

S0016, "A Phase III Trial Of CHOP + Rituximab Vs CHOP + Iodine-131-Labeled Monoclonal Anti-B1 Antibody (Tositumomab) For Treatment Of Newly Diagnosed Follicular Non-Hodgkin's Lymphomas" **(ARM 1, CHOP ONLY, OF THIS STUDY WAS PERMANENTLY CLOSED, EFFECTIVE 12/15/02)** *(11/22/02)*

This is a clinical trial (a type of research study). Clinical trials include only patients who choose to take part. Please take your time to make your decision. Discuss it with your family and friends.

You are being asked to take part in this study because you have a kind of cancer called "follicular" lymphoma. This is a cancer of the lymph nodes which is not curable with current treatments.

WHY IS THIS STUDY BEING DONE?

The purpose of this study is to find out if a combination of drugs (called CHOP) either alone or followed by an antibody (rituximab) or a radioactive antibody (Iodine-131 anti-B1 antibody - also called tositumomab), is able to stop the growth of your cancer. *(11/22/02)* **We also want to find out if the side effects of this treatment are tolerable. The rituximab antibody is a protein which is partly of mouse origin and partly of human origin. The Iodine-131 anti-B1 antibody is a mouse antibody attached to radioactive Iodine (radiolabeled). These antibodies attach to white blood cells (B cells) in your blood and tumor. The combination of CHOP chemotherapy and these antibodies have been used to treat patients with lymphoma and have been shown to be active against lymphoma. CHOP chemotherapy has been used for 30 years to treat patients with lymphomas and results in tumor shrinkage in 60 - 90% of patients. We are doing this study to see if patients treated with CHOP chemotherapy followed by the rituximab antibody or by tositumomab may have longer lasting tumor shrinkage, and hopefully cures, than patients treated with CHOP chemotherapy alone.** *(11/22/02)* **We also want to find out whether either of the two antibody treatments increase the side effects of CHOP treatment. (CHOP ONLY ARM OF THIS STUDY WAS PERMANENTLY CLOSED, EFFECTIVE 12/15/02)** *(11/22/02) (1/1/04)*

CLOSED EFFECTIVE 09/15/2008

Another goal of this study is to find out whether this treatment causes changes in the cells of your bone marrow. Because of this goal, you will have samples of your bone marrow sent to a special laboratory for testing.

Researchers would also like to do laboratory testing on tissue samples in order to find out as much as possible about non-Hodgkin's lymphoma and how this treatment might affect the disease. Some of your tissue must be submitted for this study for testing in order to confirm your type of non-Hodgkin's lymphoma. If any tissue is left over, you may choose to allow this tissue to be kept for research purposes. Also, you may choose to allow additional tissue, both preserved and fresh-frozen, to be kept and used for research purposes.

HOW MANY PEOPLE WILL TAKE PART IN THE STUDY?

About 500 people will take part in this study. *(2/15/06)*

WHAT IS INVOLVED IN THE STUDY?

Registration/Randomization

Arm 1: CHOP chemotherapy only (Permanent Closure, effective 12/15/02) *(11/22/02)*

Arm 2: CHOP chemotherapy with concurrent rituximab antibody

Arm 3: CHOP chemotherapy followed by I-131 anti-B1 antibody (tositumomab)

If you choose to participate in this study you will be "randomized" into one of the study groups described below. Randomization means that you are put into a group by chance. It is like flipping a coin. A computer decides which group you will be in. Neither you nor the researcher will choose what group you will be in. You will have an equal chance of being placed in any group.

CLOSED EFFECTIVE 09/15/2008

SWOG Authorized Document

If you are randomized to the first group (Arm 1) you will receive CHOP chemotherapy only. If you are randomized to the second group (Arm 2) you will receive CHOP chemotherapy plus the rituximab antibody along with it. If you are randomized to the third group (Arm 3) you will receive CHOP chemotherapy followed by the I-131 anti-B1 antibody, tositumomab

Arm 1: If you are assigned by the computer to Arm 1, you will receive the drugs cyclophosphamide, doxorubicin, and vincristine through a needle in your vein on the first day of each treatment "cycle". Each "cycle" lasts 21 days. You will also take the drug prednisone by mouth (pills) for the first five days of the cycle. This combination of drugs is known as CHOP. It will take about 15 - 45 minutes to receive the cyclophosphamide, 5 - 20 minutes for the doxorobucin, and 5 - 15 minutes for the vincristine. You may also receive a drug called G-CSF or GM-CSF during some cycles of your CHOP therapy if your doctor feels that it is needed to help keep your blood counts from becoming too low. If you receive G-CSF or GM-CSF, it will be injected under your skin once a day as long as your blood counts are low. This treatment will be repeated every 21 days for 6 cycles as long as your disease is getting better. If your disease or symptoms get worse, you will stop treatment on this study and be offered other treatment by your doctor.
(PERMANENT PARTIAL CLOSURE, EFFECTIVE 12/15/02) *(11/22/02)*

Arm 2: If you are assigned by the computer to Arm 2, you will receive six cycles of CHOP chemotherapy every 21 days exactly as described above in Arm 1. The CHOP chemotherapy cycles will begin on Days 8, 29, 50, 71, 92, and 113 of your treatment. You will also receive the rituximab antibody through a needle in your vein along with the CHOP chemotherapy on Days 1, 6, 48, 90, 134, and 141. It will take about 4 - 6 hours to receive the rituximab. You may also receive the drugs acetaminophen and diphenhydramine by mouth before the rituximab to help control pain and prevent possible allergic reaction. If your disease or symptoms get worse, you will stop treatment on this study and be offered other treatment by your doctor.

Arm 3: If you are assigned by the computer to Arm 3, you will receive the CHOP chemotherapy treatment every twenty-one days for six cycles exactly as described for Arm 1. In addition, about four to eight weeks after you finish the sixth cycle of this chemotherapy, you will receive a test dose of Iodine-131 tositumomab (called the "dosimetric" dose). It will take about 1 1/2 hours to receive your test dose. You will first receive the "cold" nonradioactive antibody through your vein over 60 minutes, followed by the "hot" radioactive antibody through your vein over 20 - 30 minutes. *(11/1/01)* Following this test dose, your whole body will be scanned three times with a special machine (called gamma scans) over the period of a week as an outpatient to determine the correct treatment dose of the antibody. One to two weeks after the test dose, you will receive a treatment dose (called the "therapeutic" dose) of the Iodine-131 tositumomab antibody over about 1/2 to 1 hour. You will again receive the "cold" and then the "hot" antibody through your vein as described above. Each time you receive the antibody you will receive the drugs acetaminophen and diphenhydramine before your antibody dose to help control pain and prevent allergic reaction. You will also be given

CLOSED EFFECTIVE 09/15/2008

SWOG Authorized Document

either a potassium iodide solution or potassium iodide tablets to protect your thyroid gland from damage at least 24 hours before you receive the first dose of the Iodine -131 Anti-B1 Antibody (dosimetric dose). You will continue to receive the potassium iodide for at least 14 days after you receive the second dose of the antibody (therapeutic dose). You may also receive the drug meperidine if you have trouble with muscle stiffening (rigors) during or after your infusions. Patients who have bad side effects with the test dose will not receive the treatment dose of the antibody. *(11/1/01)*

Four or five visits to a nuclear medicine physician will be required during this period of time in order to receive the test dose of I-131-tositumomab, have three gamma scans performed and then receive the treatment dose of I-131 tositumomab. *(11/1/01)* As mentioned above, you will need to take potassium iodide beginning the day before and continuing for several weeks after I-131-tositumomab to prevent damage to your thyroid gland from the radioactive iodine. *(11/1/01)* Depending on the regulations in your state, you may have to stay in "radiation isolation" in the hospital for two to four days following the radiolabeled antibody treatment. If your state permits outpatient therapy with I-131 tositumomab, you will be given special instructions by the nuclear medicine physician on how to limit exposure of other family members to the radioactivity which has been given to you. If your disease or symptoms get worse, you will stop treatment on this study and be offered other treatment by your doctor.

If you take part in this study, you will have the following tests and procedures:

During your therapy, you will have x-rays, laboratory and other tests, including blood tests and bone marrow exams. You will have blood tests about every three weeks during the CHOP therapy and at the end of your CHOP treatment so your doctor can see how your body is responding to the therapy. Also, a Hepatitis B virus screening is recommended for patients at high risk of Hepatitis B virus infection (only for patients assigned to Arm 2). *(sentence added 11/1/04)*

If you are assigned to Arm 2 or Arm 3, your study doctor will order a simple blood test to measure thyroid-stimulating hormone or "TSH". This test is used to measure and determine whether the protocol treatment is having any effect on your thyroid gland. The thyroid gland makes hormones that help control heart rate, blood pressure, body temperature, and weight. The frequency of the TSH blood test will occur as follows: before treatment, at Days 133, 200, 365 (while on protocol treatment), and annually at Years 2, 3, 4, and 5 (after the completion of protocol treatment). *(paragraph added 6/20/07)*

If you are assigned to Arm 3 and receive the I-131 anti-B1 antibody, blood tests will be done weekly during and after the Iodine-131 anti-B1 antibody until your blood counts return to normal.

CLOSED EFFECTIVE 09/15/2008

SWOG Authorized Document

You will also have your bone marrow examined (called "bone marrow aspiration and biopsy") at the start of this study, four to eight weeks after completion of CHOP therapy (if you are assigned to Arm 3), 200 days after beginning treatment (this is required only if lymphoma cells are present in your bone marrow at prestudy) and 365 days after beginning treatment. *(2/15/06) (8/8/07)* The bone marrow will be looked at to find out if any lymphoma cells are present. *(sentence added 11/21/06)* Your skin over your hipbone will be numbed by a shot of local anesthetic (lidocaine) given just under your skin. A needle will be inserted through the numbed skin and into the hipbone. The bone marrow will be removed by using suction and a twisting motion of the needle. You may have minor discomfort, and minor infection is also possible. Rarely allergic reactions to the anesthetic may occur.

CLOSED EFFECTIVE 09/15/2008

SWOG Authorized Document

(paragraph replaced 11/21/06)
You will need these tests and procedures that are either being tested in this study or being done to see how the study is affecting your body:

- Bone marrow specimen submission: a portion of the bone marrow (about 1 teaspoon) will be taken at the start of the study and again at one year (Day 365) after beginning treatment. The bone marrow specimen will be sent to a special laboratory for testing to detect chromosome breakages (translocations). This laboratory testing is for research purposes only and will not affect your cancer treatment.
- Serum specimen submission (if you are assigned to Arm 2): You will have 10 mL (about 2-3 teaspoons) of serum collected on Day 133 (prior to receiving the 5th dose of rituximab), Day 200 (about 9 weeks after the final rituximab dose), Day 365 (about 8 months after the final rituximab dose) and Day 596 (about 15 months after the final rituximab dose). These serum specimens are being collected in order to find out if your body is producing antibodies to the rituximab treatment (called human anti-mouse antibodies or "HAMA".) If you registered to the study prior to HAMA testing becoming mandatory, your serum will be drawn at the next scheduled clinic visit (if applicable). If you are already beyond Day 596 in your protocol treatment, you will have a single serum specimen submitted at the time of your next scheduled doctor's visit. *(bullet added 3/28/07)*
- Serum specimen submission (if you are assigned to Arm 3) *(1/15/07, 3/28/07)*: You will have 10 mL (about 2-3 teaspoons) of serum collected on Day 133 (prior to receiving the Iodine-131 Anti-B1 dosimetric dose), Day 200 (about 9 weeks after the dosimetric dose), Day 365 (about 8 months after the dosimetric dose) and Day 596 (about 15 months after the dosimetric dose). These serum specimens are being collected in order to find out if your body is producing antibodies to the Iodine-131 anti-B1 treatment (called human anti-mouse antibodies or "HAMA".) If you registered to the study prior to HAMA testing becoming mandatory, your serum will be drawn at the next scheduled clinic visit (if applicable). If you are already beyond Day 596 in your protocol treatment, you will have a single serum specimen submitted at the time of your next scheduled doctor's visit.

CLOSED EFFECTIVE 01/15/2008

HOW LONG WILL I BE IN THE STUDY?

We think it will take you about 4 - 5 months to complete any of the three different treatments. You will then return to your doctor about 2 months and about 7 - 8 months after you complete your treatment for tests and scans. After that at a minimum, you will return for a follow-up visit to your doctor every 6 months for two years and then once a year after that. Your doctor may wish to see you at more frequent intervals. **(ARM 1, CHOP ONLY, OF THIS STUDY WAS PERMANENTLY CLOSED, EFFECTIVE 12/15/02)** *(11/22/02)*

The researcher may decide to take you off this study if your disease gets worse despite the treatment; the side effects of the treatment are too dangerous for you; new information about the treatment becomes available and this information suggests the treatment will be ineffective or unsafe for you. It is unlikely, but the study may be stopped early due to lack of drug supply or lack of funding.

SWOG Authorized Document

WHAT ARE THE RISKS OF THE STUDY?

While on the study, you are at risk for these side effects. You should discuss these with the researcher and/or your regular doctor. There also may be other side effects that we cannot predict. Other drugs will be given to make side effects less serious and uncomfortable. Many side effects go away shortly after treatments are stopped, but in some cases side effects can be serious or long-lasting or permanent.

Risks and side effects related to the CHOP chemotherapy treatment (cyclophosphamide, doxorubicin, vincristine, and prednisone) include the following: (ARM 1, CHOP ONLY, OF THIS STUDY WAS PERMANENTLY CLOSED, EFFECTIVE 12/15/02) *(11/22/02)*

Very Likely

- **Nausea/vomiting**
- **Hair loss**
- **Decrease in appetite**
- **Decrease in blood cell counts**
- **Facial and/or abdominal swelling/puffy appearance**

Less Likely

- **Allergic reaction (may include rash, itching, swelling, cough, lowered blood pressure)**
- **Inflammation of the blood vessels in the skin where the drugs are given**
- **Sores in the mouth**
- **Discoloration of skin, nails**

CLOSED EFFECTIVE 09/15/2008

SWOG Authorized Document

- **Loosening of fingernails, toenails**
- **Facial flushing**
- **Itching of skin**
- **Headache**
- **Abdominal pain**
- **Jaw pain**
- **Brittle bones**
- **Diarrhea/constipation**
- **Fever**
- **Infection**
- **Chills**
- **Fatigue**
- **Lower or higher blood pressure**
- **Muscle weakness**
- **Tingling in the arms and legs**
- **Itchy, swollen eyes**
- **Watery eyes**
- **Changes in eyesight**
- **Changes in EKG (a test to measure heart functions)**
- **Bladder irritation (avoided by drinking 8-10 glasses of water a day)**
- **Change in color of urine**
- **Dizziness**
- **Mood swings/depression**
- **Changes in personality**
- **Menstrual changes**

<u>Less Likely, But Serious</u>

- **Scarring of lungs/shortness of breath**
- **Convulsions**
- **Heart failure**
- **Chance of acute leukemia**

Risks and side effects related to the antibody rituximab include the following: ***(section updated 3/28/07)***

<u>Likely</u>
- **Fever**
- **Chills**
- **Rigors (muscle stiffening)**
- **Nausea**
- **Fatigue (including weakness, malaise, and feelings of sluggishness or drowsiness)**
- **Decrease in white blood cells or B-cells (a type of white blood cell) that can increase your risk of developing an infection.**

CLOSED EFFECTIVE 09/15/2008

SWOG Authorized Document

Less Likely

- **Vomiting**
- **Diarrhea**
- **Headache**
- **Skin rash, development of hives or welts, or reddening of the skin**
- **Increased cough**
- **Throat irritation**
- **Sensation of tongue or throat swelling**
- **Pain in the abdomen, back, joints, and/or muscles**
- **Tumor site pain**
- **Dizziness**
- **Anxiety**
- **Irritability**
- **Bodily discomfort**
- **Loss of appetite**
- **Night sweats**
- **Lowered red blood cell counts, which may make you feel fatigued or tired.**
- **Lowered platelet counts, which may result in bruising easily and impair the ability of your blood to clot properly.**
- **Abnormal levels in blood chemistry tests that measure liver, kidney, and heart function.**
- **Decreased or increased blood pressure**
- **Numbness, tingling, or burning sensations**
- **Swelling of the arms and legs**
- **Inflammation of the mucous membranes of the nose with symptoms of sneezing, itching, nasal discharge and congestion.**
- **Shortness of breath and/or wheezing**
- **Trouble sleeping**
- **Serum sickness (allergic reaction to the rituximab 2-4 weeks after exposure)**

Rare, but serious

- **Pulmonary (lung) failure**
- **Seizure**
- **Kidney failure requiring dialysis or resulting in death.**
- **Heart problems including chest pain (angina) and life-threatening irregular heartbeats**
- **Tumor lysis syndrome (only a risk in patients with large numbers of tumor cells in their bloodstream): It is possible that this treatment may cause a rapid breakdown of the tumor cells, which can cause abnormally levels of uric acid, LDH (lactic dehydrogenase) and calcium in the blood. This can lead to complications with the kidneys and other organs if not treated immediately.**

CLOSED EFFECTIVE 09/15/2008
SWOG Authorized Document

- **<u>Allergic reactions</u>: Severe reactions from the initial infusion of rituximab have been reported. In some cases, these reactions were fatal. Signs and symptoms of severe allergic reactions include changes in blood pressure, shortness of breath or asthmatic-like conditions, and development of hives or welts beneath the skin.**
- **<u>Severe mucous membrane reactions</u>: Rarely, severe allergic-type skin reactions have been reported in patients who have received rituximab treatment. Some of these events have been fatal. The timing of these reactions has varied from 1 to 13 weeks following rituximab treatment. Depending on the type and extent of the skin changes, whether there is involvement of the mucous membrane (tissue lining or cover parts of the body such as the mouth and eyes), whether the reaction is localized or involves all of the body skin or mucous membranes, whether there are symptoms such as fever and achiness, and whether there is damage to vital organ systems, these reactions may be classified as one of a number of different disorders (Stevens-Johnson syndrome, toxic epidermal necrolysis, etc.). Should you experience any tenderness, redness, pain, lumps, sores, blistering, peeling, or loss of mucous membranes while you are on or off rituximab treatment, you should report these immediately to your study doctor so that he/she can determine whether you might be having an allergic reaction. Your study doctor will then take the appropriate steps to help you manage the reaction.**
- **<u>Stomach pain and bowel problems</u>: Serious stomach and bowel problems such as bowel obstruction (blockage of the small or large intestine) and bowel perforation (development of a hole in the small or large intestine) have been seen in some patients. In some cases, these events were fatal. A relationship between rituximab and these events has not been established. The average time for rituximab patients to develop a bowel perforation was 6 days from the start of therapy. If abdominal pain is experienced, especially early in treatment, you should contact your study doctor immediately.**
- **<u>Hepatitis B virus (HBV) reactivation and other viral infections</u>: In people who have ever been infected with hepatitis B virus, there is a risk that the virus can flare up during treatment with drugs that affect your immune system, such as rituximab. This could lead to liver failure or even death. This risk of hepatitis B virus flaring up may continue for several months after you stop taking rituximab. If you become jaundiced (yellowing of the skin and eyes) or develop viral hepatitis while taking rituximab or after stopping treatment, you should tell your study doctor immediately. Your study doctor will discuss this risk with you and explain what testing is recommended to check for hepatitis.**

  The following additional serious viral infections, either new, reactivated or made more severe, have been reported in some patients receiving rituximab in combination with chemotherapy. These viral infections include JC virus (which can lead to PML, a rare and often fatal brain

CLOSED EFFECTIVE 09/15/2008

SWOG Authorized Document

disease), cytomegalovirus (CMV), herpes simplex virus, parvovirus B19, varicella zoster virus, West Nile virus, and hepatitis C. In some cases, the viral infections occurred up to one year after stopping rituximab and resulted in death. Because there are no warning signs of PML, you should contact your study doctor immediately if you experience major changes in vision or unusual eye movements, loss of balance or coordination, and periods of disorientation or confusion. You should also contact your study doctor right away if you have a persistent cough, fever, chills, congestion, or any flu-like symptoms while receiving rituximab (or several months after discontinuation of rituximab therapy). These symptoms may be signs of a serious infection.

Immunizations: The safety of immunization with any vaccine, particularly live viral vaccines, following rituximab therapy has not been studied. It is recommended that you consult with your doctor before receiving immunizations following rituximab therapy.

CLOSED EFFECTIVE 09/15/2008

SWOG Authorized Document

Risks and side effects related to the antibody tositumomab include the following:

<u>Very Likely</u>

- **Fever**
- **Weakness**
- **Chills**
- **Loss of appetite**
- **Nausea/vomiting**
- **Diarrhea**
- **Rash**
- **Tumor site pain**
- **Decrease in blood counts**

<u>Less Likely</u>

- **Lowered blood pressure causing lightheadedness/dizziness**
- **Allergic reaction including rash, hives, itching, shortness of breath**
- *(This line removed 2/15/02)*
- **Development of "human anti-mouse antibodies" (limiting ability for further treatment with antibodies)**
- **Infection**
- **Joint pain**
- **Muscle pain**
- **Abdominal pain**
- **Headache**
- **Nose inflammation**
- **Throat inflammation**
- **Cough**
- **Diarrhea**
- **Reduced thyroid gland activity (fatigue, feeling cold, dry skin, constipation)** *(This line and those below added to "Less Likely" 11/1/01)*
- **Rapid heartbeat at time of injection**
- **Flushing**
- **Numbness**
- **Infrequent urination**
- **Bronchitis**

<u>Less Likely, but Serious</u>

- **Severe allergic reaction**
- **Bone marrow damage**
- **Chance of developing acute leukemia or other cancers**

CLOSED EFFECTIVE 09/15/2008

SWOG Authorized Document

- **Increased calcium and/or potassium** *(This line and those below added to "Less Likely, but Serious" 11/1/01)*
- **Increased uric acid**
- **Kidney failure**
- **Blood clot in the lung**

Reproductive risks: Because the drugs in this study can affect an unborn baby, you should not become pregnant or father a baby while on this study. You should not nurse your baby while on this study. Ask about counseling and more information about preventing pregnancy. ***[Include a statement about possible sterility when appropriate.]***

[Attach additional information about contraception, etc.]

(paragraph deleted 03/28/07)

For more information about risks and side effects, ask the researcher or contact ___________________________________.

CLOSED EFFECTIVE 09/15/2008

SWOG Authorized Document

ARE THERE BENEFITS TO TAKING PART IN THE STUDY?

We cannot and do not guarantee you will benefit if you take part in this study. The treatment you receive may even be harmful. Your doctors feel that your participation in this study will give you at least as good a chance as you might expect from other treatments. We hope the information learned from this study will benefit other patients with Non-Hodgkin's lymphoma in the future.

The possible benefits of taking part in the study are the same as receiving CHOP chemotherapy with or without an antibody without being in the study.

WHAT OTHER OPTIONS ARE THERE?

Instead of being in this study, you have these options:

You may receive chemotherapy as recommended by your doctor for your type of lymphoma, the rituximab antibody alone, radiation therapy or a stem cell transplant. Also you may choose no anti-cancer treatment at this time (with care to help you feel more comfortable).

You can get treatment for Non-Hodgkin's lymphoma without being on this study. All of the treatment on this study may be available at this center or at other locations.

Please talk to your regular doctor about these and other options.

WHAT ABOUT CONFIDENTIALITY?

Efforts will be made to keep your personal information confidential. We cannot guarantee absolute confidentiality. Your personal information may be disclosed if required by law.

Organizations that may inspect and/or copy your research records for quality assurance and data analysis include groups such as: the National Cancer Institute, the Food and Drug Administration, GlaxoSmithKline, the Cancer and Leukemia Group B, the Cancer Trials Support Group (CTSU), and the Southwest Oncology Group. *(12/15/01) (9/15/05) (2/15/06)*

If we publish the information we learn from this study in a medical journal, you will not be identified by name or in any other way.

CLOSED EFFECTIVE 09/15/2008

SWOG Authorized Document

WHAT ARE THE COSTS?

Taking part in this study may lead to added costs to you or your insurance company. Please ask about any expected added costs or insurance problems.

In the case of injury or illness resulting from this study, emergency medical treatment is available but will be provided at the usual charge. No funds/funds have been set aside to compensate you in the event of injury. *(local institutions must choose the option that best fits the hospital's situation)*

You or your insurance company will be charged for continuing medical care and/or hospitalization.

You will receive no payment for taking part in this study.

Administration of the drug will be (provided free of charge/charged in the usual way). The parts of the research consisting of keeping research records will be paid by those organizing and conducting the research. The research requires that you receive certain standard medical tests and examinations. These standard tests and examinations will be (charged in the usual way/provided at a reduced rate). *(local institutions must choose the option that best fits the hospital's situation)*

The drugs cyclophosphamide, doxorubicin, and vincristine, prednisone and rituximab are commercially available. Tositumomab is considered investigational for this study and will be supplied free of charge by GlaxoSmithKline until it becomes commercially available. *(9/15/05)*

Although the tositumomab is provided free of charge for this study, you may be charged for its administration.

WHAT ARE MY RIGHTS AS A PARTICIPANT?

Taking part in this study is voluntary. You may choose not to take part or may leave the study at any time. Leaving the study will not result in any penalty or loss of benefits to which you are entitled. You can stop participating at any time. However, if you decide to stop participating in the study, we encourage you to talk to the researcher and your regular doctor first.

A Data Safety and Monitoring Board, an independent group of experts, will be reviewing the data from this research throughout the study. We will tell you about important new information from this or other studies that may affect your health, welfare, or willingness to stay in this study.

CLOSED EFFECTIVE 03/15/2008

SWOG Authorized Document

WHOM DO I CALL IF I HAVE QUESTIONS OR PROBLEMS?

For questions about the study or a research-related injury, contact the researcher *NAME(S)* at *TELEPHONE NUMBER*.

For questions about your rights as a research participant, contact the *NAME OF CENTER* Institutional Review Board (which is a group of people who review the research to protect your rights) at *TELEPHONE NUMBER*. *[And, if available, list patient representative (or other individual who is not on the research team or IRB).]*

WHERE CAN I GET MORE INFORMATION?

[To IRB/Investigators: Attach information materials and checklist of attachments. Signature page should be at the end of package. You may also wish to include the following informational resources]

You may call the NCI's Cancer Information Service at 1–800–4–CANCER (1–800–422–6237) or TTY: 1–800–332–8615

Visit the NCI's Web sites…
cancerTrials: comprehensive clinical trials information http://cancertrials.nci.nih.gov.

CancerNet™: accurate cancer information including PDQ http://cancernet.nci.nih.gov.

You will get a copy of this form. You may also request a copy of the protocol (full study plan).

SIGNATURE

You are deciding whether or not to take part in this study. If you sign, it means that you have decided to volunteer to take part in this study, and that you have read and understood all the information on this form.

Participant ________________________________ Date ___________________

CLOSED EFFECTIVE 09/15/2008

Consent for use of excess diagnostic tissue for research purposes:

Preserved tissue from your tumor must be submitted for this study in order to confirm your type of non-Hodgkin's lymphoma. There may be some tissue remaining once your diagnosis has been confirmed.

If you are willing to allow this excess tissue to be used for unspecified, future research studies, please specify your consent below.

SWOG Authorized Document

Consent Form for Use of Tissue For Research

About Using Tissue for Research

You have had a biopsy (or surgery) to identify your cancer. Your doctor will remove some body tissue to do some tests. The results of these tests will be given to you by your doctor and will be used to plan your care.

We would like to keep some of the tissue that is left over for future research. If you agree, this tissue will be kept and may be used in research to learn more about cancer and other diseases. Please read the question and answer sheet (attached) called "How is Tissue Used for Research" to learn more about tissue research.

Your tissue may be helpful for research whether you do or do not have cancer. The research that may be done with your tissue probably will not help you. It might help people who have cancer and other diseases in the future.

Reports about research done with your tissue will not be given to you or your doctor. These reports will not be put in your health record. The research will not have an effect on your care.

Things to Think About

The choice to let us keep the left over tissue for future research is up to you. No matter what you decide to do, it will not affect your care.

If you decide now that your tissue can be kept for research, you can change your mind at any time. Just contact us and let us know that you do not want us to use your tissue. Then the tissue will no longer be used for research.

In the future, people who do research may need to know more about your health. When the Southwest Oncology Group gives them reports about your health, it will not give them your name, address, or phone number.

Sometimes tissue is used for genetic research (about diseases that are passed on in families). Even if your tissue is used for this kind of research, the results will not be put in your health records.

Your tissue will be used only for research and will not be sold. The research done with your tissue may help to develop new products in the future.

Benefits

The benefits of research using tissue include learning more about what causes cancer and other diseases, how to prevent them, how to treat them, and how to cure them.

CLOSED EFFECTIVE 09/15/2008

SWOG Authorized Document

Risks

There are very few risks to you. The greatest risk is the release of information from your health records. The Southwest Oncology Group will protect your records so that your name, address, and phone number will be kept private. The chance that this information will be given to someone else is very small.

Please read each sentence below and think about your choice. After reading each sentence, check "Yes" or "No." **No matter what you decide to do, it will not affect your care.** If you have any questions, please talk to your doctor or nurse. For questions about your rights as a research participant, contact the *name of center* Institutional Review Board (which is a group of people who review the research to protect your rights) at *telephone number*.

The results of tissue research may help find new ways to learn about, prevent, or treat cancer and other diseases. Please read each sentence below and think about your choice. After reading each sentence, circle the answer that is right for you. If you have any questions, please talk to your doctor or nurse, or call the National Cancer Institute's Cancer Information Service at 1/800-422-6237 (1/800-4-CANCER).

---

1. **My tissue may be kept for use in research to learn about, prevent, treat, or cure cancer**.

   Yes ____________ No ____________

---

2. **My tissue may be kept for research about other health problems (for example: causes of diabetes, Alzheimer's disease, and heart disease).**

   Yes ____________ No ____________

---

3. **Someone from the Southwest Oncology Group may contact me in the future to ask me to take part in more research**.

   Yes ____________ No ____________

Please sign your name here after you check your answers.

Participant ________________________________ Date ________________________

CLOSED EFFECTIVE 09/15/2008

SWOG Authorized Document

FOR SOUTHWEST ONCOLOGY GROUP INSTITUTIONS ONLY *(12/15/01)*

If you are willing to submit additional frozen and preserved tissue for unspecified future research purposes, you will be registered to the Central Lymphoma Repository Tissue Procurement Protocol, **SWOG-8819**. Please specify your consent below.

1) My tissue (specimen) may be kept for use in research to learn about, prevent or treat cancer.

Yes__________ No.________ Initial________

3) My tissue (specimen) may be kept for use in research to learn about, prevent, or treat other health problems (for example: diabetes, Alzheimer's disease, or heart disease).

Yes__________ No.________ Initial________

2) Someone from the Southwest Oncology Group may contact me in the future to ask me to take part in more research.

Yes__________ No.________ Initial________

Please sign your name here after you check your answers.

Participant ________________________ Date _________________________________

CLOSED EFFECTIVE 09/15/2008

SWOG Authorized Document

Tissue and Specimen Consent Supplemental Sheets

How are Tissue and Specimens Used for Research?

Where do tissue and specimens come from?

After a person has had a biopsy (or surgery) and all tests have been done, there may be some left over tissue. Sometimes, this tissue is thrown away because it is not needed for the patient's care. Instead, a patient can choose to have the tissue kept for future research. A specimen may be from a blood sample or from bone marrow, skin, toenails or other body materials. People who are trained to handle tissue and protect donors' rights make sure that the highest standards of quality control are followed by the Southwest Oncology Group. Your doctor does not work for the Southwest Oncology Group, but has agreed to help collect tissue from many patients. Many doctors across the country are helping in the same way. If you agree, only left over tissue will be saved for research. Your doctor will not take more tissue during surgery than needed for your care.

Why do people do research with tissue and specimens?

Research with tissue and specimens can help to find out more about what causes cancer, how to prevent it, how to treat it, and how to cure it. Research using specimens can also answer other health questions. Some of these include finding the causes of diabetes and heart disease, or finding genetic links to Alzheimer's.

What type of research will be done with my tissue and specimens?

Many different kinds of studies use tissue and specimens. Some researchers may develop new tests to find diseases. Others may develop new ways to treat or even cure diseases. In the future, some of the research may help to develop new products, such as tests and drugs. Some research looks at diseases that are passed on in families (called genetic research). Research done with your tissue may look for genetic causes and signs of disease.

How do researchers get the tissue and specimens?

Researchers from universities, hospitals, and other health organizations conduct research using tissue and specimens. They contact the Southwest Oncology Group and request samples for their studies. The Southwest Oncology Group reviews the way that these studies will be done, and decides if any of the samples can be used. The Southwest Oncology Group gets the tissue and specimens and information about you from your hospital, and sends the tissue samples and specimens and some information about you to the researcher. The Southwest Oncology Group will not send your name, address, phone number, social security number or any other identifying information to the researcher.

Will I find out the results of the research using my tissue and specimens?

You will receive the results of your biopsy, but you will not receive the results of research done with your tissue. You will not receive the results of research done with your specimens. This is because research can take a long time and must use tissue samples from many people before results are known. Results from research using your tissue may not be ready for many years and will not affect your care right now, but they may be helpful to people like you in the future.

Why do you need information from my health records?

In order to do research with your tissue and specimens, researchers may need to know some things about you. (For example: Are you male or female? What is your race or ethnic group? How old are you? Have you ever smoked?) This helps researchers answer questions about diseases. The information that will be given to the researcher may include your age, sex, race, diagnosis, treatments and family history. This information is collected by your hospital from your health record and sent to the Southwest Oncology Group. If more information is needed, the Southwest Oncology Group will send it to the researcher.

CLOSED EFFECTIVE 09/15/2008

SWOG Authorized Document

Will my name be attached to the records that are given to the researcher?

No. Your name, address, phone number and anything else that could identify you will be removed before they go the researcher. The researcher will not know who you are.

How could the records be used in ways that might be harmful to me?

Sometimes, health records have been used against patients and their families. For example, insurance companies may deny a patient insurance or employers may not hire someone with a certain illness (such as AIDS or cancer). The results of genetic research may not apply only to you, but to your family members too. For disease caused by gene changes, the information in one person's health record could be used against family members.

How am I protected?

The Southwest Oncology Group is in charge of making sure that information about you is kept private. The Southwest Oncology Group will take careful steps to prevent misuse of records. Your name, address, phone number and any other identifying information will be taken off anything associated with your specimen before it is given to the researcher. This would make it very difficult for any research results to be linked to you or your family. Also, people outside the research process will not have access to results about any one person which will help to protect your privacy.

What if I have more questions?

If you have any questions, please talk to your doctor or nurse, or call our research review board at (Insert IRB's Phone Number).

CLOSED EFFECTIVE 09/15/2008

SWOG Authorized Document

Southwest Oncology Group Statistical Center
1100 Fairview Avenue North, MP557
PO Box 19024
Seattle, WA 98109-1024
Patient Registration (206) 667-4623
CCOP Patient Registration (206) 652-2267

Southwest Oncology Group Operations Office
14980 Omicron Drive
San Antonio, TX 78245-3217
(210) 677-8808

Southwest Oncology Group Registration Form

SWOG Study No. S0016 **Registration Step** 1 **Assigned Treatment Arm** ☐

Activation Date: March 1, 2001

Last Amended Date: December 15, 2001

A Phase III Trial of CHOP Vs CHOP + Rituximab Vs CHOP + Iodine-131-Labeled Monoclonal Anti-B1 Antibody (Tositumomab) for Treatment of Newly Diagnosed Follicular Non-Hodgkin's Lymphomas

Patient's Name ______________________

SWOG Patient ID ☐☐☐☐☐☐

Other Group Patient Number ☐☐☐☐☐☐☐☐☐☐

Other Group Name/ Protocol Number ______________________

INSTRUCTIONS: All of the information on this Registration Form and the Protocol Eligibility Section must be answered appropriately for a patient to be considered eligible for registration. This Registration Form must be entirely filled out and referred to during the registration. Do NOT submit this form as part of the patient data.

Caller's SWOG Roster ID ☐☐☐☐☐

IRB Approval Date ☐☐ / ☐☐ / ☐☐☐☐

Date Informed Consent Signed ☐☐ / ☐☐ / ☐☐☐☐

SWOG Investigator Number ☐☐☐☐☐ **OR** **Other Group Investigator Name and Number** ______________ / ______

SWOG Treating Institution Number ☐☐☐☐☐ **OR** **Other Group Institution Name and Number** ______________ / ______

Projected Start Date of Treatment ☐☐ / ☐☐ / ☐☐☐☐

Patient Consent for Specimen Use:

Patient's tissue may be kept for use in research to learn about, prevent, treat, or cure cancer.
☐ Yes ☐ No

Patient's tissue may be kept for research about other health problems (for example: causes of diabetes, Alzheimer's disease, and heart disease).
☐ Yes ☐ No

Someone from the Southwest Oncology Group may contact the patient in the future to ask him or her to take part in more research.
☐ Yes ☐ No

Patient's Date of Birth: ☐☐ / ☐☐ / ☐☐☐☐

Patient's Race / Ethnicity: ☐☐ / ☐☐

Patient's Sex: ☐ Female ☐ Male

Method of Payment: ☐☐

If a U.S. resident:

Patient's Social Security Number: ☐☐☐ - ☐☐ - ☐☐☐☐

Patient's ZIP Code: ☐☐☐☐☐

Country of Residence, if not USA: ______________________

If a resident of Canada:

Social Insurance Number: ☐☐☐ - ☐☐☐ - ☐☐☐

Postal Code: ☐☐☐ - ☐☐☐

Stratification Factor: Beta-2 microglobulin > IULN: ☐ Yes ☐ No

Southwest Oncology Group Registration Form Code Sheet

Patient's race definitions:

White or Caucasian: a person having origins in any of the original peoples of Europe, Middle East, or North Africa.

Black or African American: a person having origins in any of the black racial groups of Africa.

Native Hawaiian or Other Pacific Islander: a person having origins in any of the original peoples of Hawaii, Guam, Samoa and other Pacific islands.

Asian: a person having origins in any of the original peoples of the Far East, Southeast Asia, or the Indian subcontinent. Including, for example, Cambodia, China, India, Japan, Korea, Malaysia, Pakistan, the Philippine Islands, Thailand and Vietnam.

American Indian or Alaskan Native: a person having origins in any of the original peoples of North, Central or South America, and who maintains tribal affiliations or community attachment.

Patient's ethnicity (Spanish/Hispanic Origin) options:

Unknown
No (not Spanish)
Yes, Mexican
Yes, Puerto Rican
Yes, Cuban
Yes, Central American
Yes, South American
Yes, Other
Yes, NOS

Method of Payment codes:

Private
Medicare
Medicare and Private
Medicaid
Medicaid and Medicare
No insurance (self-pay)
No insurance (no means)
Other, specify at registration
Unknown
Veterans Admin
Military

Other Group codes for use in the Web Registration program:

9977 – ACOSOG
9982 – CALGB
9976 – CTSU
9995 – ECOG
9984 – GOG
9987 – MDACC
9996 – NCCTG
9981 – NCIC
9983 – NSABP
9997 – RTOG

SOUTHWEST ONCOLOGY GROUP
S0016 NON-HODGKIN'S LYMPHOMA (FOLLICULAR) PRESTUDY FORM

SWOG Patient ID ☐☐☐☐☐☐ **SWOG Study No.** S 0 0 1 6 **Registration Step** 1

Patient Initials ______________(L, F M)

Institution/Affiliate ___________________________________ Physician ___________________________________

Participating Group: Group Name/Study No./Patient ID ______________ / ______________/ ______________

Instructions: All dates are **MONTH, DAY, YEAR**. Explain any blank fields or blank dates in the **Comments** section.
Place an [X] in appropriate boxes. Circle **AMENDED** items in red and write **AMENDED** across the top of the form.

PATIENT AND DISEASE DESCRIPTION

Date of First Pathologic Diagnosis: ☐☐ / ☐☐ / ☐☐☐☐

Height (cm): ☐☐☐ **Weight (kg):** ☐☐☐.☐ **BSA (m^2):** ☐.☐☐ **Performance Status:** ☐

REAL Classification Histology - B Cell: ☐ Follicular Grade I ☐ Follicular Grade II ☐ Follicular Grade III

Current Stage of Disease: ☐ II ☐ III ☐ IV

Does the patient have bulky disease (any mass ≥ 10 cm in diameter or a mediastinal mass > 1/3 chest diameter)? ☐ No ☐ Yes

Symptoms: ☐ A (No Symptoms) ☐ B (Fever, Weight Loss, and/or Night Sweats)

CURRENT LABORATORY VALUES

LDH (U/l): ☐☐☐☐ **Serum-ß2 Microglobulin (mg/L):** ☐☐.☐ **Hemoglobin:** ☐☐☐ g/dL

LDH ULN: ☐☐☐☐ **Serum-ß2 IULN (mg/L):** ☐☐.☐ **Hemoglobin ILLN:** ☐☐☐ g/dL

Albumin (g/dl): ☐☐.☐☐

CURRENT LYMPHATIC TISSUE INVOLVEMENT

Is there current nodal involvement above the diaphragm? ☐ No ☐ Yes

If yes, number of sites involved above the diaphragm*: ☐☐

Is there current nodal involvement below the diaphragm? ☐ No ☐ Yes

If yes, number of sites involved below the diaphragm*: ☐☐

Is there current splenic involvement? ☐ No ☐ Yes

** Instructions for counting the number of involved nodal sites are on page 2 of this form.*

CURRENT EXTRANODAL INVOLVEMENT

Is there current extranodal involvement? ☐ No ☐ Yes

If Yes, select a box for each involved: ☐ Bone Marrow ☐ Lung ☐ Liver ☐ CNS/Brain ☐ GI Tract ☐ Other:____________________

continued on next page

SOUTHWEST ONCOLOGY GROUP
S0016 NON-HODGKIN'S LYMPHOMA (FOLLICULAR) PRESTUDY FORM

SWOG Patient ID | | | | | | | **SWOG Study No.** S 0 0 1 6 **Registration Step** 1

Patient Initials ______________(L, F M)

Instructions: The figure below indicates how to count the number of involved nodal sites according to Solal-Celigny et al., Blood, 2004. Each box represents a nodal area. Multiple involved nodes within a single nodal area (or box) are counted as 1 nodal site. Bilateral disease is counted as 2 nodal sites. Bilateral nodal areas include axillary, cervical, inguinal, epitrochlear, and popliteal. The mediastinal, mesenteric, and para aortic nodal areas are not considered to be bilateral; in these cases multiple involved nodes from each are counted as at most only 1 nodal site. Iliac nodes are a component of the para aortic nodal area and are also NOT considered to be bilateral. *(Note: Other non-bilateral sites include preaortic and subcarinal; these are counted as 1 nodal site.)*

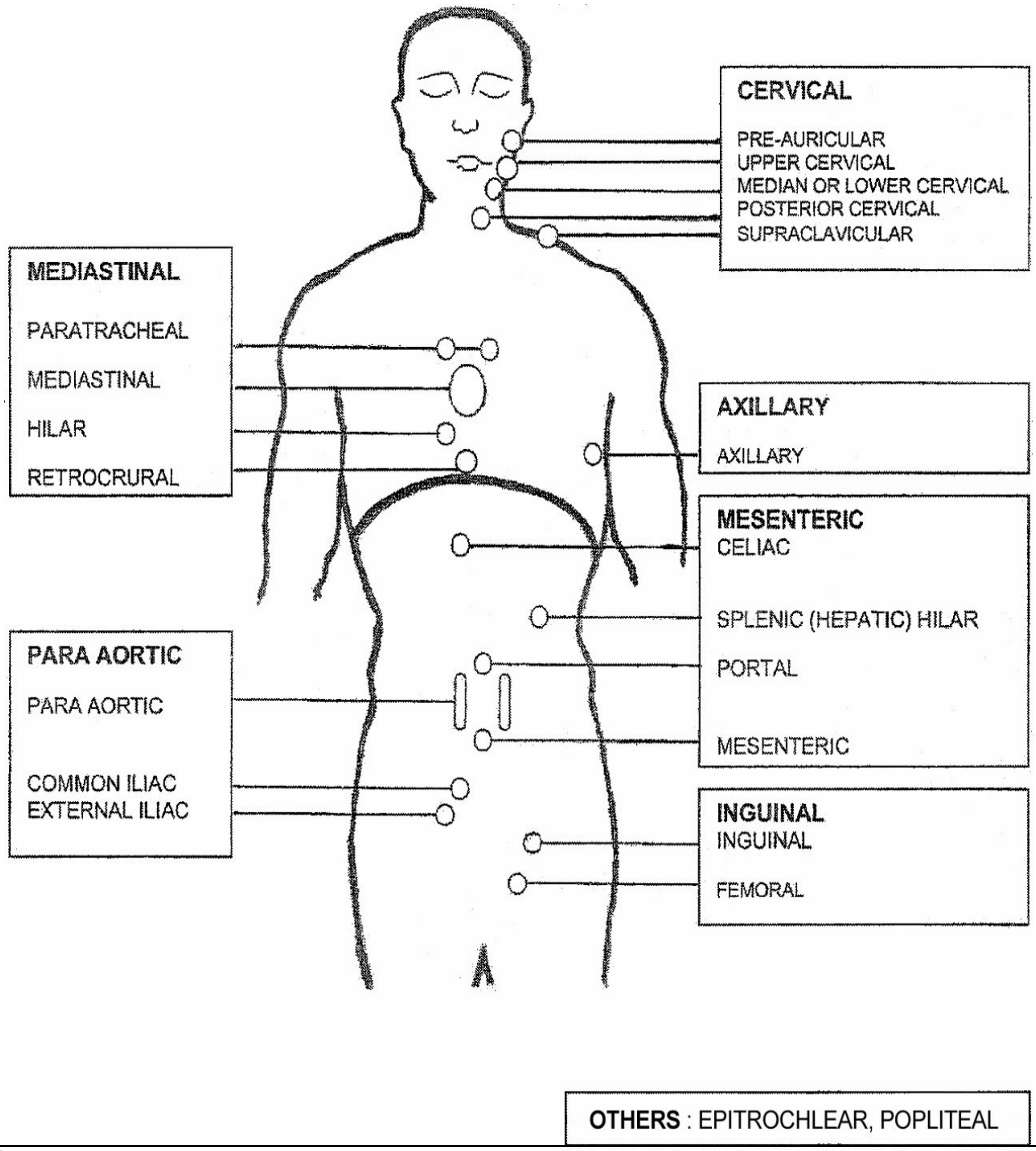

Comments:

7/1/2007

32770

SOUTHWEST ONCOLOGY GROUP
LYMPHOMA BASELINE TUMOR ASSESSMENT FORM

SWOG Patient No. ☐☐☐☐☐☐ SWOG Study No. S☐☐☐☐ Protocol Step: 1

Patient Initials ______________(L,F,M)

Institution/Member ______________________________ Physician ______________________________

Groups other than SWOG: Group Name/Study No./Patient No. __________ / __________ / __________

Instructions: Please use black ink. Circle **AMENDED** items in red. Record the requested information for all measurable lesions and all sites of evaluable and non-evaluable disease. Please refer to section 10.1 of the protocol for definitions. If an organ or site has too many measurable lesions to measure at each evaluation, choose three to follow as measurable disease and record the rest as evaluable disease. For measurable lesions, check the RT-No Progression box if the lesion was previously irradiated and has not progressed since.
The same test procedures used for baseline disease assessment must be used for all required subsequent disease assessments.

Site of Target Lesions

	Site of Measurable Lesions	RT-No Progr.	Tumor Measurement (cm)	Assessment Code*	Date of Assessment
**L1**	________	☐	☐☐.☐ x ☐☐.☐	☐☐	☐☐/☐☐/☐☐☐☐
**L2**	________	☐	☐☐.☐ x ☐☐.☐	☐☐	☐☐/☐☐/☐☐☐☐
**L3**	________	☐	☐☐.☐ x ☐☐.☐	☐☐	☐☐/☐☐/☐☐☐☐
**L4**	________	☐	☐☐.☐ x ☐☐.☐	☐☐	☐☐/☐☐/☐☐☐☐
**L5**	________	☐	☐☐.☐ x ☐☐.☐	☐☐	☐☐/☐☐/☐☐☐☐
**L6**	________	☐	☐☐.☐ x ☐☐.☐	☐☐	☐☐/☐☐/☐☐☐☐

	Other Sites of Disease	Extent	Assessment Code*	Date of Assessment
**S1**	________	________	☐☐	☐☐/☐☐/☐☐☐☐
**S2**	________	________	☐☐	☐☐/☐☐/☐☐☐☐
**S3**	________	________	☐☐	☐☐/☐☐/☐☐☐☐
**S4**	________	________	☐☐	☐☐/☐☐/☐☐☐☐
**S5**	________	________	☐☐	☐☐/☐☐/☐☐☐☐

List all **negative** diagnostic tests/studies used to evaluate patient for malignancy.

**Tests/studies**	**Date**	**Tests/studies**	**Date**
________	☐☐/☐☐/☐☐☐☐	________	☐☐/☐☐/☐☐☐☐
________	☐☐/☐☐/☐☐☐☐	________	☐☐/☐☐/☐☐☐☐

* **Assessment Codes**:
01-Palpation
02-Visualization
03-Colposcopy
04-CA-125 assay
05-Endoscopy
10-Plain film/X-ray without contrast
11-Plain film/X-ray with contrast
12-CT scan
13-MRI scan
14-Radioisotope scan
15-Ultrasound
20-Histologic confirmation
21-Cytologic confirmation
99-Other (specify below and indicate lesion number)
____
____

Notes:

SOUTHWEST ONCOLOGY GROUP
S0016 TOSITUMOMAB TREATMENT FORM

SWOG Patient ID ☐☐☐☐☐☐ **SWOG Study No.** S 0 0 1 6 **Registration Step** 1

Patient Initials ________ (L, F M)

Institution/Affiliate ______________________________ Physician ____________________

Participating Group: Group Name/Study No./Patient ID ____________ / ____________ / ____________

Instructions: Please complete and submit this form after completion of I-131 treatment. This form should only be completed for patients randomized to Arm 3 (CHOP + I-131). All dates are **MONTH, DAY, YEAR**. Explain any blank dates or fields in a **Comments** section. Place an ☒ in appropriate boxes. Circle **AMENDED** items in red and write **AMENDED** across the top of the form.

STATUS

Date of Last Contact or Death: ☐☐ / ☐☐ / ☐☐☐☐ **Vital Status:** ☐ Alive ☐ Dead *(submit Notice of Death)*

Has the patient progressed per the definition in Section 10.0 of the protocol? ☐ No ☐ Yes *(submit Follow-up Form)*

TOSITUMOMAB TREATMENT

Start date: ☐☐ / ☐☐ / ☐☐☐☐ **Weight (first day this cycle):** ☐☐☐ . ☐ kg

Date of last treatment: ☐☐ / ☐☐ / ☐☐☐☐ **BSA (first day this cycle):** ☐ . ☐☐ m^2

Were there any dose modifications or additions/omissions to protocol treatment?

- ☐ No
- ☐ Yes, planned (per protocol guidelines), specify in comments
- ☐ Yes, unplanned (not per protocol guidelines), specify in comments

Report total dose for reporting period

Agent	Dose
Dosimetric: Anti-B1 antibody predose	☐☐☐ mg
Dosimetric: Anti-B1 antibody hot dose	☐☐☐ mg
Dosimetric: Iodine-131 dose	☐☐☐ mCi
Therapeutic: Anti-B1 antibody predose	☐☐☐ mg
Therapeutic: Anti-B1 antibody hot dose	☐☐☐ mg
Therapeutic: Iodine-131 dose	☐☐☐ mCi

Comments:

SOUTHWEST ONCOLOGY GROUP
S0016 CHOP/R-CHOP TREATMENT FORM

SWOG Patient ID ☐☐☐☐☐☐ **SWOG Study No.** S 0 0 1 6 **Registration Step** 1

Patient Initials ________ (L, F M)

Cycle Submission: ☐ End of cycle 1-6 of CHOP/R-CHOP (enter cycle number): ☐

Institution/Affiliate ______________________ Physician ______________

Participating Group: Group Name/Study No./Patient ID ________ / ________ / ________

Instructions: Please complete and submit this form after each cycle of R-CHOP (for patients randomized to Arm 2, CHOP + Rituxumab) or CHOP (for patients randomized to Arm 3, CHOP + I-131). All dates are **MONTH, DAY, YEAR**. Explain any blank dates or fields in a **Comments** section. Place an X in appropriate boxes. Circle **AMENDED** items in red and write **AMENDED** across the top of the form.

STATUS

Date of Last Contact or Death: ☐☐ / ☐☐ / ☐☐☐☐ **Vital Status:** ☐ Alive ☐ Dead *(submit Notice of Death)*

Has the patient progressed per the definition in Section 10.0 of the protocol? ☐ No ☐ Yes *(submit Follow-up Form)*

TREATMENT FOR THIS CYCLE

Cycle start date: ☐☐ / ☐☐ / ☐☐☐☐ **Weight (first day this cycle):** ☐☐☐.☐ kg

Date of last treatment for this cycle: ☐☐ / ☐☐ / ☐☐☐☐ **BSA (first day this cycle):** ☐.☐☐ m^2

Were there any dose modifications or additions/omissions to protocol treatment?

☐ No
☐ Yes, planned (per protocol guidelines), specify in comments
☐ Yes, unplanned (not per protocol guidelines), specify in comments

Report total dose for reporting period

**Agent**	**Dose**
Cyclophosphamide	☐☐☐☐ mg
Doxorubicin	☐☐☐ mg
Vincristine	☐.☐ mg
Prednisone	☐☐☐ mg
Rituximab	☐☐☐☐ mg

Comments:

SOUTHWEST ONCOLOGY GROUP
S0016 ADVERSE EVENT FORM

SWOG Patient ID [ ] **SWOG Study No.** S0016 **Registration Step** 1

Patient Initials ________ (L, F M)

Cycle submission:
- ☐ End of cycle 1-6 of CHOP or R-CHOP (enter cycle number): ☐
- ☐ 2 weeks after completion of I-131 treatment (for patients on the CHOP I-131 arm)
- ☐ 3 months after removal from protocol treament (either arm)

Institution/Affiliate ____________________________ Physician ____________________

Participating Group: Group Name/Study No./Patient ID ____________ / ____________/ ____________

Instructions: For patients on the R-CHOP arm (Arm 2), please complete and submit this form at the end of every cycle of R-CHOP (cycles 1-6). For patients on the CHOP I-131 Tositumomab arm (Arm 3), please complete and submit this form at the end of every cycle of CHOP (cycles 1-6) and once 2 weeks after the completion of I-131 treatment. For either arm, please also complete once 3 months after removal from protocol treatment. Report adverse events occurring up until the next cycle of treatment begins. Document the worst Grade seen during the reporting period. Do not code a condition existing prior to registration as an adverse event unless it worsens. Category lists may not include all adverse events from that category. Record any observed adverse events not listed on the blank lines at the end. All dates are MONTH, DAY, YEAR. Explain any blank dates or fields in the **Comments** section. Place an X in appropriate boxes. Circle **AMENDED** items in red and write **AMENDED** across top of form.

TOXICITY **Were toxicities assessed during this time period?**

☐ **No** ☐ **Yes** **Date of most recent toxicity assessment:** __ __ / __ __ / __ __ __ __

☐ Mark box if toxicities were assessed but none were seen. Otherwise indicate grades below.

CTC 2.0 Code	Toxicity	Grade (1 - 5)	Treatment Relation*	CTC 2.0 Code	Toxicity	Grade (1 - 5)	Treatment Relation*
CA20	Cardiac ischemia/infarction			GI30	Constipation bowel obstruction		
CA50	Hypertension			GI60	Stomatitis/pharyngitis		
CA51	Hypotension			GU03	Urinary frequency/urgency		
CA53	Edema			GU50	Creatinine increase		
CA99	Cardiovascular - other			GU53	Renal failure		
CL20	PTT			HE00	Leukopenia		
FL01	Fever without neutropenia			HE10	Thrombocytopenia		
FL10	Rigors/chills			HE20	Anemia		
FL30	Sweating			HE30	Neutropenia/granulocytopenia		
FL40	Fatigue/malaise/lethargy			HE40	Lymphopenia		
GI00	Nausea			IM00	Allergic/hypersensitivity		
GI01	Anorexia			IN00	Infection without 3-4 neutropenia		
GI10	Vomiting			IN05	Infection with 3-4 neutropenia		
GI20	Diarrhea without colostomy			IN30	Febrile neutropenia		
GI21	Diarrhea with colostomy			IN99	Infection with unknown ANC		
GI23	Dehydration			LI00	Bilirubin increase		

*** Treatment Relation codes:** 1-unrelated 2-unlikely 3-possible 4-probable 5-definite

SOUTHWEST ONCOLOGY GROUP
S0016 ADVERSE EVENT FORM

SWOG Patient ID ☐☐☐☐☐☐ **SWOG Study No.** S 0 0 1 6 **Registration Step** 1

Patient Initials ________ (L, F M)

Cycle submission:
- ☐ End of cycle 1-6 of CHOP or R-CHOP (enter cycle number): ☐
- ☐ 2 weeks after completion of I-131 treatment (for patients on the CHOP I-131 arm)
- ☐ 3 months after removal from protocol treament (either arm)

TOXICITY, *continued*

CTC 2.0 Code	Toxicity	Grade (1 - 5)	Treatment Relation*	CTC 2.0 Code	Toxicity	Grade (1 - 5)	Treatment Relation*
LI11	SGOT (AST) increase	☐	☐	PA00	Headache	☐	☐
LI12	SGPT (ALT) increase	☐	☐	PA21	Myalgia	☐	☐
LU00	Dyspnea	☐	☐	PA22	Arthralgia	☐	☐
LU10	Hypoxia	☐	☐	PA99	Pain - other	☐	☐
LU60	Cough	☐	☐	SK11	Rash/desquamation	☐	☐
LY00	Lymphedema	☐	☐	SK90	Alopecia	☐	☐
ME30	Hypoglycemia	☐	☐	SY10	Tumor lysis syndrome	☐	☐
ME31	Hyperglycemia	☐	☐				
ME90	Hypophosphatemia	☐	☐		Other Toxicities (specify):		
MS99	Joint, muscle, bone - other	☐	☐		________	☐	☐
NR50	Weakness (motor neuropathy)	☐	☐		________	☐	☐
NR60	Sensory - neuropathy	☐	☐		________	☐	☐
NR92	Insomnia	☐	☐		________	☐	☐

*** Treatment Relation codes:** 1-unrelated 2-unlikely 3-possible 4-probable 5-definite

Comments:

SOUTHWEST ONCOLOGY GROUP
S0016 TSH REPORTING FORM

SWOG Patient ID ☐☐☐☐☐☐ **SWOG Study No.** S 0 0 1 6 **Registration Step** 1

Patient Initials ________ (L, F M)

Assessment timepoint:

☐ Prestudy ☐ Day 133 ☐ Day 200 ☐ Day 365 ☐ 2 years ☐ 3 years ☐ 4 years ☐ 5 years

Institution / Affiliate ____________________ Physician ____________________

Participating Group: Group Name/Study No./Patient ID ________ / ________ / ________

Instructions: All dates are **MONTH, DAY, YEAR**. Explain any blank fields or blank dates in the **Comments** section. Place an X in appropriate boxes. Circle **AMENDED** items in red and write **AMENDED** across top of form.

Date of assessment: ☐☐ / ☐☐ / ☐☐☐☐

Has the patient received thyroid hormone replacement therapy?

☐ Currently receiving and/or received in past year ☐ Not receiving now and none in past year

Current TSH value: ☐☐.☐ mIU/ml

TSH ULN: ☐☐.☐ mIU/ml

Comments:

OFF TREATMENT NOTICE

SWOG Patient No. ☐☐☐☐☐☐ SWOG Study No. S☐☐☐☐ Protocol Step ☐

Disease Committee ____________________ Patient Initials (L, F, M) __________

Institution / Member ____________________ Physician ____________________

Groups other than SWOG: Group Name/Study No./Pt. No. __________ /__________ /__________

INSTRUCTIONS: For each protocol step, submit this form within 2 weeks after completion (or discontinuation) of treatment. List protocol-directed treatments that the patient received.

Chemotherapy: List regimens, start and stop dates. For multidrug regimens, do not list individual drugs separately; stop date would be the date all drugs in the regimen were discontinued.

Surgery: List type of surgery and in the "stop" column the date of surgery.

Radiation: List sites, start and stop dates (inclusive of boosts and implants).

Indicate an unknown part of a date with a horizontal line drawn across the appropriate boxes.

Start Date (mm,dd,yyyy)	Stop Date (mm,dd,yyyy)	REGIMEN or PROCEDURE or SITE(S)
☐☐/☐☐/☐☐☐☐	☐☐/☐☐/☐☐☐☐	____________________
☐☐/☐☐/☐☐☐☐	☐☐/☐☐/☐☐☐☐	____________________
☐☐/☐☐/☐☐☐☐	☐☐/☐☐/☐☐☐☐	____________________
☐☐/☐☐/☐☐☐☐	☐☐/☐☐/☐☐☐☐	____________________
☐☐/☐☐/☐☐☐☐	☐☐/☐☐/☐☐☐☐	____________________

(If more room is needed, please continue on a separate page)

Reason OFF TREATMENT (select one)

- ☐ Treatment completed per protocol
- ☐ Toxicity, medically required, specify:
- ☐ Patient refused, due to toxicity, specify:
- ☐ Patient refused, other than toxicity, specify:
- ☐ Progression or relapse. Sites:
- ☐ Death (attach Notice of Death form)
- ☐ Other, specify: ____________________

Date OFF TREATMENT

Date of completion, progression, death or decision to discontinue therapy ☐☐/☐☐/☐☐☐☐

Will patient receive FURTHER TREATMENT?

☐ No ☐ Yes, specify: ____________________ ☐ Unknown

Date of Last Contact (or death): ☐☐/☐☐/☐☐☐☐

VITAL STATUS: ☐ Alive ☐ Dead (attach Notice of Death form)

Notes:

BY: ____________________ DATE: ____________________

2/1/2000

22204

NOTICE OF DEATH

SWOG Patient No. ☐☐☐☐☐☐

Most Recent SWOG Study No. S☐☐☐☐

Disease Committee ____________________

Patient Initials (L, F, M) ____________

Institution / Member ____________________________

Physician ____

Groups other than SWOG: Group Name/Study No./Pt. No. ________/________/________

INSTRUCTIONS: Submit within 4 weeks of knowledge of death. AMENDED data: ☐ Yes, mark amended items in red.

Date of Death ☐☐ / ☐☐ / ☐☐☐☐ (month, day, year)

Causes of Death

Any cancer (check one)

☐ No ☐ Primary Cause ☐ Contributory ☐ Possible ☐ Unknown

If patient has had multiple tumor types, specify those which were causes of death:

____

____

____

Toxicity from disease related treatment (check one)

☐ No ☐ Primary cause ☐ Contributory ☐ Possible ☐ Unknown

If Primary Cause, Contributory or Possible, specify treatment and toxicity: ____________

____

Non-cancer and non-treatment related causes (check one)

☐ No ☐ Primary cause ☐ Contributory ☐ Possible ☐ Unknown

If Primary Cause, Contributory or Possible, specify ____________

____

Autopsy done? ☐ No ☐ Yes ☐ Unknown

Death information obtained from (check all that apply)

☐ Autopsy report

☐ Medical record / death certificate

☐ Physician

☐ Relative or friend

☐ Other, specify ____

Notes:

BY: ________________________ DATE: __________________

SOUTHWEST ONCOLOGY GROUP
S0016 FOLLOW UP FORM

SWOG Patient ID [| | | | |] SWOG Study No. [S|0|0|1|6] Registration Step [1]

Patient Initials ____________ (L, F M)

Institution / Affiliate ______________________________ Physician ______________________________

Participating Group: Group Name/Study No./Patient ID ______________ / ______________ / ______________

Instructions: Please submit at each follow up after completion of treatment until relapse or progression, at time of relapse or progression, and at protocol-specified intervals after relapse or progression. Also submit at time of diagnosis of second primary. All dates are **MONTH, DAY, YEAR**. Explain any blank fields or blank dates in the **Comments** section. Place an [X] in appropriate boxes. Circle **AMENDED** items in red and write **AMENDED** across the top of the form.

VITAL STATUS

Vital Status: ☐ Alive ☐ Dead Date of last contact or death: __ __ / __ __ / __ __ __ __

If vital status is Dead, complete and submit Notice of Death form.

DISEASE FOLLOW UP STATUS

Has the patient had a documented clinical assessment for this cancer (since submission of the previous follow-up form)?

☐ No ☐ Yes If Yes, Date of Last Clinical Assessment: __ __ / __ __ / __ __ __ __

NOTICE OF FIRST RELAPSE OR PROGRESSION

Has the patient developed a first relapse or progression that has not been previously reported?

☐ No ☐ Yes If Yes, Date of Relapse or Progression: __ __ / __ __ / __ __ __ __

Site(s) of Relapse or Progression: ______________________________

NOTICE OF NEW PRIMARY

Has a new primary cancer or MDS (myelodysplastic syndrome) been diagnosed that has not been previously reported?

☐ No ☐ Yes If Yes, Date of Diagnosis: __ __ / __ __ / __ __ __ __

New Primary Site: ______________________________

NON-PROTOCOL TREATMENT

Has the patient received any non-protocol cancer therapy not previously reported?

☐ No ☐ Yes If Yes, Beginning Date of Non-Protocol Therapy: __ __ / __ __ / __ __ __ __

Agent Name(s): ______________________________

LONG TERM ADVERSE EVENT

Has the patient experienced (prior to treatment for progression or relapse or a second primary, and prior to non-protocol treatment) any severe (grade $\geq$ 3) long term toxicity that has not been previously reported?

☐ No ☐ Yes If Yes, Adverse Events and Grades: ______________________________

Comments:

SOUTHWEST ONCOLOGY GROUP
LYMPHOMA FOLLOW-UP TUMOR ASSESSMENT FORM

SWOG Patient ID ☐☐☐☐☐☐ **SWOG Study No.** ☐☐☐☐☐ **Registration Step** ☐

(L, F M)
Patient Initials ________ Institution/Affiliate ________________________ Physician ____________________

Groups other than SWOG: Group Name/Study No./Patient No. __________ / __________ / __________

Instructions: Please use black ink. Circle **AMENDED** items in red. Record the requested information for all measurable lesions and all sites of evaluable and non-evaluable disease. Please refer to section 10.1 of the protocol for definitions. If an organ or site has too many measurable lesions to measure at each evaluation, choose three to follow as measurable disease and record the rest as evaluable disease. For measurable lesions, check the RT-No Progression box if the lesion was previously irradiated and has not progressed since.
The same test procedures used for baseline disease assessment must be used for all required subsequent disease assessments.

Site of Target Lesions

	Site of Measurable Lesions	RT-No Progr.	Tumor Measurement (cm)	Assessment Code*	Date of Assessment
**L1**	________	☐	☐☐.☐ x ☐☐.☐	☐☐	☐☐ / ☐☐ / ☐☐☐☐
**L2**	________	☐	☐☐.☐ x ☐☐.☐	☐☐	☐☐ / ☐☐ / ☐☐☐☐
**L3**	________	☐	☐☐.☐ x ☐☐.☐	☐☐	☐☐ / ☐☐ / ☐☐☐☐
**L4**	________	☐	☐☐.☐ x ☐☐.☐	☐☐	☐☐ / ☐☐ / ☐☐☐☐
**L5**	________	☐	☐☐.☐ x ☐☐.☐	☐☐	☐☐ / ☐☐ / ☐☐☐☐
**L6**	________	☐	☐☐.☐ x ☐☐.☐	☐☐	☐☐ / ☐☐ / ☐☐☐☐

	Other Sites of Disease	Extent	Assessment Code*	Date of Assessment
**S1**	________	________	☐☐	☐☐ / ☐☐ / ☐☐☐☐
**S2**	________	________	☐☐	☐☐ / ☐☐ / ☐☐☐☐
**S3**	________	________	☐☐	☐☐ / ☐☐ / ☐☐☐☐
**S4**	________	________	☐☐	☐☐ / ☐☐ / ☐☐☐☐
**S5**	________	________	☐☐	☐☐ / ☐☐ / ☐☐☐☐

	New Lesions (Specify Site)	Assessment Code*	Date of Assessment
**S1**	________	☐☐	☐☐ / ☐☐ / ☐☐☐☐
**S2**	________	☐☐	☐☐ / ☐☐ / ☐☐☐☐

List all **negative** diagnostic tests/studies used to evaluate patient for malignancy.

**Tests/studies**	**Date**	**Tests/studies**	**Date**
________	☐☐ / ☐☐ / ☐☐☐☐	________	☐☐ / ☐☐ / ☐☐☐☐
________	☐☐ / ☐☐ / ☐☐☐☐	________	☐☐ / ☐☐ / ☐☐☐☐

* **Assessment Codes**:
01-Palpation
02-Visualization
03-Colposcopy
04-CA-125 assay
05-Endoscopy
10-Plain film/X-ray without contrast
11-Plain film/X-ray with contrast
12-CT scan
13-MRI scan
14-Radioisotope scan
15-Ultrasound
20-Histologic confirmation
21-Cytologic confirmation
99-Other (specify below and indicate lesion number)
____
____

SOUTHWEST ONCOLOGY GROUP
S0016 LABORATORY AND IMAGING DATA FORM

SWOG Patient ID ☐☐☐☐☐☐ **SWOG Study No.** S 0 0 1 6 **Registration Step** 1

Patient Initials__________(L, F M) **Assessment date:** ☐☐ // ☐☐ // ☐☐☐☐

Visit (select one):

CHOP and Concurrent Rituximab Arm:

- ☐ Baseline
- ☐ Day 29
- ☐ Day 50
- ☐ Day 71
- ☐ Day 92
- ☐ Day 113
- ☐ Day 141
- ☐ Day 200
- ☐ Day 365

CHOP Followed by Tositumomab and I-131 Tositumomab:

- ☐ Baseline
- ☐ Day 22
- ☐ Day 43
- ☐ Day 64
- ☐ Day 85
- ☐ Day 106
- ☐ Day 133
- ☐ Day 200
- ☐ Day 365

For patients on either arm:

☐ Year 1.5 ☐ Year 2 ☐ Year 2.5 ☐ Year 3 ☐ Year 4

☐ Unscheduled visit

Institution / Affiliate ____________________ Physician ____________________

Instructions: Please complete and submit this form based on data available at the designated timepoints for patients registered to S0016. For patients on the CHOP/Rituximab arm, complete the form at baseline, Days 29, 50, 71, 92, 113, 141, 200, and 365. For patients on the CHOP/I-131 arm, complete the form at baseline, Days 22, 43, 64, 85, 106, 133, 200, and 365. The form should also be completed for patients on either arm at 1.5 Years after registration, and at Years 2, 2.5, 3 and 4 after registration. All dates are **MONTH, DAY, YEAR**. Explain any blank fields or blank dates in the **Comments** section. Place an X in appropriate boxes. Circle **AMENDED** items in red and write **AMENDED** across top of form.

NON-CENTRAL LABORATORY HEMATOLOGY

Lab name: ____________________

Assay	Result	Units	Low range	High range	Mark if Data Not Available Or Test Not Done
WBC	☐☐☐.☐	1000/UL	☐☐☐.☐	☐☐☐.☐	☐
Hemoglobin	☐☐.☐	g/dL	☐☐.☐	☐☐.☐	☐
Hematocrit	☐☐.☐	%	☐☐.☐	☐☐.☐	☐
Platelets	☐☐☐	1000/UL	☐☐☐	☐☐☐	☐
ANC	☐☐.☐☐	1000/UL	☐☐.☐☐	☐☐.☐☐	☐
Lymphocytes	☐☐.☐	%	☐☐.☐	☐☐.☐	☐

____

(total of any cells not specified in the differential above)

continued on next page

SOUTHWEST ONCOLOGY GROUP
S0016 LABORATORY AND IMAGING DATA FORM

SWOG Patient ID ☐☐☐☐☐☐ **SWOG Study No.** S 0 0 1 6 **Registration Step** 1

Patient Initials____________(L, F M) **Assessment date:** ☐☐ / ☐☐ / ☐☐☐☐

NON-CENTRAL LABORATORY CHEMISTRY

Assay	Result	Units	Low range	High range	Mark if Data Not Available Or Test Not Done
Potassium	☐.☐	mmol/L	☐.☐	☐.☐	☐
BUN	☐☐.☐	mg/dL	☐☐.☐	☐☐.☐	☐
Creatinine	☐.☐	mg/dL	☐.☐	☐.☐	☐
Calcium	☐☐.☐	mg/dL	☐☐.☐	☐☐.☐	☐
AST/SGOT	☐☐☐.☐	U/L	☐☐☐.☐	☐☐☐.☐	☐
ALT/SGPT	☐☐☐.☐	U/L	☐☐☐.☐	☐☐☐.☐	☐
Alkaline phosphatase	☐☐☐	U/L	☐☐☐	☐☐☐	☐
LDH	☐☐☐☐	U/L	☐☐☐☐	☐☐☐☐	☐
Total bilirubin	☐☐.☐	mg/dL	☐☐.☐	☐☐.☐	☐
TSH	☐☐.☐☐	U/L	☐☐.☐☐	☐☐.☐☐	☐
HAMA	☐☐.☐☐	ng/mL	☐☐.☐☐	☐☐.☐☐	☐
Uric acid	☐☐.☐	mg/dL	☐☐.☐	☐☐.☐	☐
CD20	☐ Negative	☐ Positive			☐
HBV screening	☐ Negative	☐ Positive			☐

continued on next page

SOUTHWEST ONCOLOGY GROUP
S0016 LABORATORY AND IMAGING DATA FORM

SWOG Patient ID ☐☐☐☐☐☐ **SWOG Study No.** S 0 0 1 6 **Registration Step** 1

Patient Initials____________(L, F M) **Assessment date:** ☐☐ / ☐☐ / ☐☐☐☐

CT OR OTHER RADIOLOGICAL IMAGING RESULTS

Head/neck ☐ Not done **Date:** ☐☐ / ☐☐ / ☐☐☐☐

Method (select one): ☐ CT ☐ MRI ☐ X-ray ☐ Other, specify: ____________

Results: ☐ Normal ☐ Abnormal, specify: ____________

Chest ☐ Not done **Date:** ☐☐ / ☐☐ / ☐☐☐☐

Method (select one): ☐ CT ☐ MRI ☐ X-ray ☐ Other, specify: ____________

Results: ☐ Normal ☐ Abnormal, specify: ____________

Abdomen ☐ Not done **Date:** ☐☐ / ☐☐ / ☐☐☐☐

Method (select one): ☐ CT ☐ MRI ☐ X-ray ☐ Other, specify: ____________

Results: ☐ Normal ☐ Abnormal, specify: ____________

Pelvis ☐ Not done **Date:** ☐☐ / ☐☐ / ☐☐☐☐

Method (select one): ☐ CT ☐ MRI ☐ X-ray ☐ Other, specify: ____________

Results: ☐ Normal ☐ Abnormal, specify: ____________

Other ☐ Not done **Date:** ☐☐ / ☐☐ / ☐☐☐☐

Method (select one): ☐ CT ☐ MRI ☐ X-ray ☐ Other, specify: ____________

Results: ☐ Normal ☐ Abnormal, specify: ____________

NHL THERAPY

Any new NHL Therapy since the last visit/contact? ☐ Yes ☐ No

Type of Therapy	Number of Cycles	Start Date	End Date
____________	☐☐	☐☐ / ☐☐ / ☐☐☐☐	☐☐ / ☐☐ / ☐☐☐☐
____________	☐☐	☐☐ / ☐☐ / ☐☐☐☐	☐☐ / ☐☐ / ☐☐☐☐
____________	☐☐	☐☐ / ☐☐ / ☐☐☐☐	☐☐ / ☐☐ / ☐☐☐☐
____________	☐☐	☐☐ / ☐☐ / ☐☐☐☐	☐☐ / ☐☐ / ☐☐☐☐
____________	☐☐	☐☐ / ☐☐ / ☐☐☐☐	☐☐ / ☐☐ / ☐☐☐☐

Comments:

19.0 APPENDIX

19.1 Dosimetry Instructions and Worksheets

a. Dosimetry Instructions

b. Worksheet #1 - Gamma Camera Daily Quality Control

c. Worksheet #2 - Gamma Camera Whole Body Dosimetry

d. Table 1 - Maximum Effective Mass

e. Table 2 - Activity Hours

f. Graph 1 - Total Body Residence Time Estimation

19.2 Assessment of Bone Marrow Involvement

19.3 a. Ordering Instructions for Tositumomab and Iodine I-131 Tositumomab

b. Study Drug Order Form for **S0016**

19.4 **S0016** Site Contact Information Form

19.5 Determination of Expedited Adverse Event Reporting Requirements

19.6 Cancer Trials Support Unit (CTSU) Participation Procedures

19.7 HAMA assay - Methods

CLOSED EFFECTIVE 09/15/2008

SWOG Authorized Document

19.1 DOSIMETRY INSTRUCTIONS AND WORKSHEETS

a DOSIMETRY INSTRUCTIONS

1. INTRODUCTION

The dosimetric methodology for determining the therapeutic Iodine-131 activity (dose) to be administered to the patient as Iodine-131 anti-B1 antibody involves the following two steps: (a) following administration of the dosimetric dose, sequentially measuring the whole-body elimination kinetics of the radiolabeled antibody with an appropriately collimated and calibrated gamma camera operated in whole body scanning mode [serial anterior whole body scans on Day 1 (within 1 hour of completion of Iodine-131 anti-B1 antibody infusion), Day 3, 4 or 5, and Day 7 or 8], and (b) calculating the Iodine-131 activity (therapeutic dose) to be administered to the patient. The following sections describe the required techniques for gamma camera validation, background determination, whole body counts, and calculating the Iodine-131 activity to be administered for therapy as Iodine-131 anti-B1 antibody.

2. GAMMA CAMERA PROCEDURES, QUALITY CONTROL, AND COUNTS

a. Gamma Camera and Dose Calibrator Procedures

The gamma camera can be either a single- or dual-head camera with a large or extra large field of view and can be equipped with either a high-energy or medium-energy parallel hole collimator suitable for performing whole body scans and whole body counts with Iodine-131. A 5 x 10^6 count ^{99m}Tc or ^{57}Co extrinsic flood image using the selected collimator should be obtained before the gamma camera/collimator system is used for dose calculations.

Institution- and/or manufacturer-specific quality control procedures for the gamma camera/computer system and the dose calibrator should be followed each day patient imaging is to be performed. The performance of the gamma camera and dose calibrator must be within performance guidelines. Steps must be taken to ascertain reasons for variation and corrective measures must be taken before injecting or imaging the patient.

b. Gamma Camera Quality Control

Camera sensitivity must be determined each day the patient is to be imaged by scanning a calibrated activity of Iodine-131. This is accomplished by obtaining an Iodine-131 source (approximately 200 - 250 µCi) at the time the first scanning is to be initiated per patient, placing it in a glass or plastic 10cc or 20-cc normal saline vial (as used for dilutions), adding 10 - cc of water or saline, and sealing the vial. This 200 - 250 µCi source can be Iodine-131 anti-B1 antibody or other soluble Iodine-131. Note that by the final day, this source will contain approximately 100-150 µCi due to physical decay.

Check the source activity immediately before the scanning is to be done with an NIST-traceable-calibrated dose calibrator at the Iodine-131 setting. Record the activity.

CLOSED EFFECTIVE 09/15/2008

SWOG Authorized Document

Set up computer and camera as follows:

High-energy or medium-energy parallel hole collimator
Symmetric window centered on the 364 keV photopeak of Iodine-131 (314-414 keV)

Matrix: minimum 128 x 128

Scanning speed: 30-100 cm/minute

The same computer and camera setup, collimator, ROI, and scanning speed must be used for the patient and background scans. The scan should be obtained with the camera at a distance of 30 cm above the table to simulate patient imaging parameters and should be of the same length as the patient scans.
Draw a rectangular ROI around the entire field of view to obtain the source counts. Record the counts. Only anterior counts are used in calculations. The same ROI should be used for source, background, and whole body patient counts on each day. The counts should be done just before scanning the patient.

The collection of background counts is described in the next section. Following the collection of the background counts on each day, the background-corrected source count (defined as the source count minus the background count) is calculated. The counts per µCi are calculated by dividing the background-corrected source count by the calibrated activity of that day. For a specific camera and collimator, the counts per µCi should be relatively constant. If the ratio on a post-infusion day is within ±10% of the ratio from the infusion day (Day 1), the quality control is adequate and the patient can be scanned. When values vary more than 10% from the established ratio, the reason for the discrepancy must be ascertained and corrected before treating the patient.

c. <u>Background counts</u>
The background in the patient imaging room must be determined before the patient is scanned, as outlined below:

Maintain the same camera and computer setup, collimator, ROI, and scanning speed used for the quality control scanning of the calibrated source. The scan should be obtained with the camera at a distance of 30 cm above the table to simulate patient imaging parameters.

Draw a rectangular ROI around the entire field of view of the image to obtain the background counts (C_B). Record the background counts. Only anterior counts are used in calculations. The same ROI should be used for the background counts on each day. This should be done just before imaging the patient. The same ROI that is used for the background scans must be used for the patient whole body dosimetry.

If abnormally high background counts are measured, the source should be identified and, if possible, removed. Verify that the same scanning speed, the camera window setting, and collimator are being used for each scan before the background radioactivity level is re-measured. If abnormally low background counts are measured, it must be assured that the ROI is correctly delineated. Similarly, the camera energy window setting and collimator should be verified before repeating the background counts.

CLOSED EFFECTIVE 09/15/2008

SWOG Authorized Document

The dosimetry worksheets for the first 3 patients at each clinical site must be submitted to GlaxoSmithKline by fax to confirm that the calculations were performed correctly (fax: 877/279-1512). A dosimetry hotline will be maintained by GlaxoSmithKline to assist in calculation of the proper therapeutic dose (Service Center, toll free 877/423-9927).

d. Gamma Camera Whole Body Counts for Dosimetry

Anterior whole body counts should be performed immediately after the background counts on each of the 3 days (Day 1; Day 3, 4, or 5; and Day 7 or 8).

The following procedures must be used to determine the kinetics of Iodine-131 from the whole body. The whole body counts will be obtained at three timepoints (Day 1; Day 3, 4, or 5; and Day 7 or 8 post-infusion). On Day 1 (≤ 1 hour from end of infusion), the whole body counts must be obtained before any bladder emptying has occurred. For the other two timepoints, the counts will all be obtained immediately following patient voiding. The patient scans are to be obtained immediately following the background determinations. Details are as follows:

Maintain the camera and computer set up from quality control and background determination.

Scan the anterior whole body. For any particular patient, the same gamma camera, collimator and scan speed must be used for all scans. Extremities should be included in the scans. To obtain proper counts, the arms should not cross over the body. Bring the camera head(s) as close to the patient as possible. The scans should be centered on the midline of the patient. Record the time of day that the counts are obtained. Draw a rectangular ROI around the entire field of view to obtain the anterior counts (C_A). Record the patient anterior counts. The same ROI should be used for the patient counts on each day. The patient scanning distance must always be the same.

3. CALCULATION OF IODINE-131 ACTIVITY (THERAPEUTIC DOSE) AND SAMPLE CALCULATION

a. Calculation of Iodine-131 Activity

The following equation is used to calculate the activity of Iodine-131 to administer to the patient as Iodine-131 anti-B1 antibody to achieve the desired total body dose of radiation (cGy):

Iodine-131 Activity (mCi) =

$$\frac{\text{Activity Hours (mCi h)}}{\text{Residence Time (h)}} \times \frac{\text{Desired Total Body dose (cGy)}}{\text{75 cGy}}$$

The methods for determining the activity hours (mCi h), residence time (h), and desired total body dose (cGy) are described below:

1. Activity Hours (mCi h)

In order to determine the activity hours (mCi h), look up the patient's maximum effective mass derived from the patient's sex and height in Table 1. Then, use either the patient's weight in kg or the maximum effective mass, whichever is less, to look up the value for activity hours (mCi h) in Table 2.

CLOSED EFFECTIVE 09/15/2008
SWOG Authorized Document

2. Residence Time (h)

In order to determine the residence time (h), the % injected activity remaining at the two post-dosimetric dose-imaging timepoints [calculated from the start of the infusion to the time of image acquisition on Day 3, 4, or 5, and Day 7 or 8] must be calculated. [For each timepoint, calculate the total body count by subtracting the background count from the anterior patient count.]

Once the background-corrected total body counts have been calculated for the two timepoints, the % injected activity remaining at each timepoint is calculated by dividing the total body counts from that timepoint by the total body counts from Day 1 and multiplying by 100.

The residence time can now be calculated by using the graphical method:

Graphical Method

The residence time (h) is determined by plotting the time from the start of the infusion and the % injected activity values for the last 2 imaging timepoints on Graph 1. A best-fit line is then drawn from 100% (the pre-plotted Day 0 value) through the 2 plotted points (if the line does not intersect the two points, one point must lie above the best-fit line and one point must lie below the best-fit line). The residence time (h) is read from the x-axis of the graph at the point where the fitted line intersects the horizontal 37% injected activity line.

3. Desired Total Body Dose (cGy)

The desired total body dose is 75 cGy for patients with a baseline platelet count of ≥ 150,000 cells/mm^3. The desired total body dose (cGy) is 65 cGy for patients with a baseline platelet count of 100,000 to 149,999 cells/mm^3.

b. Sample Calculation

RH is a 63-year-old, 5'6" male who weighs 90 kg. His baseline platelet count is 121,000 cells/mm^3 and his % injected activities from 1 h, 72 h, and 168 h were 100%, 50%, and 20%, respectively. From Table 1, his maximum effective mass is determined to be 88.5 kg. As his maximum effective mass is less than his weight, it is used to look up the value for activity hours from Table 2; the activity hours is determined to be 9490 mCi h. By plotting the % injected activity values on Graph 1, the residence time is determined to be 103 hours. As the patient's platelet count is between 100,000 and 149,999 cells/mm^3, the desired total body dose is 65 cGy. The equation for Iodine-131 activity is then solved as follows:

$$\textbf{Iodine-131 Activity (mCi)} = \frac{\textbf{9490 mCi h}}{\textbf{103 h}} \times \frac{\textbf{65 cGy}}{\textbf{75 cGy}} = \textbf{80 mCi}$$

CLOSED EFFECTIVE 03/14/2008

SWOG Authorized Document

GlaxoSmithKline

Site Number (*GSK will complete*): ___________________
Patient Identification Number: _________________
Patient Initials (LFM): _________________

DOSIMETRY CALCULATION WORKSHEETS FOR SWOG S0016 PROTOCOL

Instructions for completing worksheets:

- Enter all dates as MM/DD/YYYY and times as HH:MM based on a 24-hour clock (i.e. 13:30)
- Gamma Camera and settings should be consistent for all scans done (i.e. I-131 source, background, and patient counts)
- A preliminary dose estimate must be done after the second scan. The estimated dose must be communicated to the Bexxar Service Center to determine if an additional vial may be required for the therapeutic dose.
- When patient dose calculation is complete, fax all Worksheets to GSK at (877) 279-1512 or via email to BEXXARServiceCenter@GSK.com for confirmation of calculations (for the first 3 patients enrolled at the study site or until GSK determines it is no longer necessary).
- If using a commercial radiopharmacy, fax Worksheet #2 to radiopharmacy, *after receiving confirmation of calculations*.

Worksheet #1 — Gamma Camera Daily Quality Control

**STUDY DAY**	**Day 1**	**Day 3, 4, or 5**	**Day 7 or 8**
**DATE**	________	________	________
**DOSE CALIBRATOR ACTIVITY**			
Time Measured	________	________	________
I-131 source activity ($\mu Ci_{I\text{-}131}$)	________	________	________
**GAMMA CAMERA COUNTS**			
CAMERA SETTINGS			
Camera Name: _______________			
Collimator: ☐ Medium Energy ☐ High Energy			
Scanning Speed (30 – 100 cm/min)	________	________	________
Whole Body Scan Field of View	________	________	________
Camera Height from Table (anterior head; cm)	________	________	________
BACKGROUND COUNTS			
Time Started	________	________	________
TOTAL COUNTS: Anterior (C_{BA})	________	________	________
I-131 SOURCE (200-250µCi on Day 1)			
Time Started (t)	________	________	________
TOTAL COUNTS: Anterior (C_{SA})	________	________	________
**CALCULATIONS**			
1. Background Corrected Source Counts $C_S = C_{SA} - C_{BA}$	________	________	________
2. Percent Initial Count $\%ISC = C_{S(2 \text{ or } 3)} / C_{S1} \times 100$	________	________	________
3. Counts per µCi $= C_S / \mu Ci_{I\text{-}131}$	________	________	________

Date Recorded/Initials ________ ________ ________

GlaxoSmithKline

Site Number (*GSK will complete*): ____________

Patient Identification Number: ____________

Patient Initials (LFM): ____________

Worksheet #2 — Gamma Camera Whole Body Dosimetry

A. Dosimetric Dose Infusion

Date of Infusion: ____________

Start Time (t_{INF}): ____________

End Time: ____________ (not including flush)

Residual after infusion: ________ mCi ________ time

Net activity to patient: ________ mCi

B. Determination of Activity Hours

Patient gender: ☐ Male ☐ Female

Patient height: ________ cm Patient weight: ________ kg

Patient maximum effective mass (Table 1) ________ kg

Is patient weight above maximum effective mass?

☐ Yes - Use maximum effective mass (from Table 1) to determine Activity Hours

☐ No - Use patient's actual weight to determine Activity Hours

Activity Hours (Table 2): ____________ mCi-hour

D. Determination of Total Body Dose (cGy)

Platelet count ____________ cells/ mm^3

Date: ________

Prescribed Total Body Dose:

☐ 65 cGy (for PLT count of 100,000 to 149,999 cells/mm^3)

☐ 75 cGy (for PLT count of ≥150,000 cells/mm^3)

A. Determination of Residence Time (h) using whole body gamma camera counts

STUDY DAY	Day 1	Day 3, 4, or 5	Day 7 or 8
**DATE**	______	______	______
**GAMMA CAMERA COUNTS**			
BACKGROUND COUNTS			
Time Started	______	______	______
TOTAL COUNTS: Anterior (C_{BA})	______	______	______
PATIENT TOTAL BODY COUNTS			
Time Started (t)	______	______	______
TOTAL COUNTS: Anterior (C_{PA})	______	______	______
**CALCULATIONS**			
1. Background Corrected Patient Counts $C_P = C_{PA} - C_{BA}$	C_{P1}=	C_{P2} =	C_{P3} =
2. Time from start of ^{131}I Anti-B1 Infusion to start of Patient Counts $t - t_{INF}$	t_1 = hr	t_2 = hr	t_3 = hr
3. Percent Injected Activity $\%IA = C_{P(2\text{ or }3)} / C_{P1} \times 100$	$\%IA_1$ = 100%	$\%IA_2$ =	$\%IA_3$ =
Date Recorded/Initials	______	______	______

Residence Time (from Graph 1) = ________ hours

Site Number (*GSK will complete*): ____________________
Patient Identification Number: ____________________
Patient Initials (LFM): ____________________

E. Calculation of Iodine-131 Anti-B1 Antibody Activity (*Date Calculated/Initials:* ______________________**)**

$$^{131}\text{Iodine Activity (mCi)} = \frac{\text{Activity Hours (mCi h)}}{\text{Residence Time (h)}} \times \frac{\text{Prescribed Total Body Dose (cGy)}}{75\ \text{cGy}}$$

$$= \frac{\_\_\_\_\_\_\_\_\_\_\ \text{mCi h}}{\_\_\_\_\_\_\_\_\_\_\ \text{h}} \times \frac{\_\_\_\_\_\_\ \text{cGy}}{75\ \text{cGy}} = \boxed{\_\_\_\_\_\_\_\_\_\_\ \text{mCi}}$$

DATE AND TIME OF PLANNED ADMINISTRATION: ____

F. Therapeutic Dose Iodine I 131 Tositumomab Infusion

Start Time (t_{INF}):____________ End Time:______________(not including flush)

Residual after infusion: ________mCi __________time

Net activity to patient: ____________mCi

Site Number (*GSK will complete*): ___________________
Patient Identification Number: _________________
Patient Initials (LFM): _________________

Table 1
Maximum Effective Mass

MEN		
Height (ft—inches)	Height (cm)	**Maximum Effective Mass (kg)**
4'-5"	134.5	**40.5**
4'-6"	137.0	**44.2**
4'-7"	140.0	**47.9**
4'-8"	142.0	**51.6**
4'-9"	145.0	**55.3**
4'-10"	147.5	**59.0**
4'-11"	150.0	**62.7**
5'-0"	152.5	**66.3**
5'-1"	155.0	**70.0**
5'-2"	157.5	**73.7**
5'-3"	160.0	**77.4**
5’-4"	162.5	**81.1**
5'-5"	165.0	**84.8**
5'-6"	167.5	**88.5**
5'-7"	170.0	**92.2**
5’-8"	172.5	**95.8**
5'-9"	175.5	**99.5**
5'-10"	178.0	**103.2**
5'-11"	180.5	**106.9**
6'-0"	183.0	**110.6**
6'-1"	185.5	**114.3**
6'-2"	188.0	**118.0**
6'-3"	190.5	**121.7**
6'-4"	193.0	**125.4**
6'-5"	195.5	**129.0**
6'-6"	198.0	**132.7**
6'-7"	200.5	**136.4**
6'-8"	203.0	**140.0**
6'-9"	205.5	**143.8**
6'-10"	208.5	**147.5**
6'-11"	211.0	**151.2**
7'-0"	213.5	**154.9**

WOMEN		
Height (ft—inches	Height (cm)	**Maximum Effective Mass (kg)**
4'-5"	134.5	**40.7**
4'-6"	137.0	**43.8**
4'-7"	140.0	**47.0**
4'-8"	142.0	**50.2**
4'-9"	145.0	**53.3**
4'-10"	147.5	**56.5**
4'-11"	150.0	**59.7**
5’-0"	152.5	**62.8**
5’-1"	155.0	**66.0**
5’-2"	157.5	**69.2**
5’-3"	160.0	**72.3**
5’-4"	162.5	**75.5**
5’-5"	165.0	**78.7**
5’-6"	167.5	**81.8**
5’-7"	170.0	**85.0**
5’-8"	172.5	**88.2**
5’-9"	175.5	**91.3**
5’-10"	178.0	**94.5**
5’-11"	180.5	**97.7**
6’-0"	183.0	**100.8**
6'-1"	185.5	**104.0**
6'-2"	188.0	**107.2**
6'-3"	190.5	**110.3**
6'-4"	193.0	**113.5**
6'-5"	195.5	**116.7**
6'-6"	198.0	**119.8**
6'-7"	200.5	**123.0**
6'-8"	203.0	**126.2**
6'-9"	205.5	**129.3**
6'-10"	208.5	**132.5**
6'-11"	211.0	**135.7**
7'-0"	213.5	**138.8**

Multiply pounds by 0.454 to obtain kilograms. Multiply inches by 2.54 to obtain centimeters. To calculate the maximum effective mass for patient heights not included in above table, use the following formulas:

Males: **Maximum Effective Mass (kg) = 65.76 + 1.452 (Ht. in cm - 152)**
Females: **Maximum Effective Mass (kg) = 62.34 + 1.247 (Ht. in cm - 152)**

Adapted from (K. Zasadny, R. Wahl, *et al.*, J Nuc Med 1995; 36(5):214. “Total Body Mass Lean”).

GlaxoSmithKline

Site Number (*GSK will complete*): ___________________
Patient Identification Number: _________________
Patient Initials (LFM): _________________

Table 2
Activity Hours

Mass[1] (kg)	Activity Hours (mCi h)	Mass[1] (kg)	Activity Hours (mCi h)	Mass[1] (kg)	Activity Hours (mCi h)	Mass[1] (kg)	Activity Hours (mCi h)	Mass[1] (kg)	Activity Hours (mCi h)
40.0	**4638**	60.0	**6686**	80.0	**8670**	100.0	**10595**	120.0	**12463**
40.5	**4690**	60.5	**6737**	80.5	**8718**	100.5	**10643**	120.5	**12509**
41.0	**4743**	61.0	**6787**	81.0	**8767**	101.0	**10690**	121.0	**12556**
41.5	**4796**	61.5	**6838**	81.5	**8816**	101.5	**10738**	121.5	**12602**
42.0	**4848**	62.0	**6888**	82.0	**8864**	102.0	**10785**	122.0	**12648**
42.5	**4901**	62.5	**6938**	82.5	**8913**	102.5	**10833**	122.5	**12694**
43.0	**4953**	63.0	**6989**	83.0	**8961**	103.0	**10880**	123.0	**12741**
43.5	**5005**	63.5	**7039**	83.5	**9010**	103.5	**10927**	123.5	**12787**
44.0	**5057**	64.0	**7089**	84.0	**9058**	104.0	**10975**	124.0	**12833**
44.5	**5109**	64.5	**7139**	84.5	**9106**	104.5	**11022**	124.5	**12879**
45.0	**5160**	65.0	**7189**	85.0	**9154**	105.0	**11069**	125.0	**12925**
45.5	**5212**	65.5	**7238**	85.5	**9202**	105.5	**11116**	125.5	**12971**
46.0	**5264**	66.0	**7288**	86.0	**9251**	106.0	**11163**	126.0	**13017**
46.5	**5315**	66.5	**7338**	86.5	**9299**	106.5	**11210**	126.5	**13063**
47.0	**5366**	67.0	**7387**	87.0	**9347**	107.0	**11257**	127.0	**13109**
47.5	**5418**	67.5	**7437**	87.5	**9394**	107.5	**11304**	127.5	**13155**
48.0	**5469**	68.0	**7486**	88.0	**9442**	108.0	**11351**	128.0	**13200**
48.5	**5520**	68.5	**7536**	88.5	**9490**	108.5	**11398**	128.5	**13246**
49.0	**5571**	69.0	**7585**	89.0	**9538**	109.0	**11445**	129.0	**13292**
49.5	**5621**	69.5	**7634**	89.5	**9585**	109.5	**11492**	129.5	**13337**
50.0	**5672**	70.0	**7683**	90.0	**9633**	110.0	**11538**	130.0	**13383**
50.5	**5724**	70.5	**7733**	90.5	**9682**	110.5	**11585**	130.5	**13429**
51.0	**5775**	71.0	**7783**	91.0	**9730**	111.0	**11632**	131.0	**13474**
51.5	**5826**	71.5	**7833**	91.5	**9779**	111.5	**11678**	131.5	**13520**
52.0	**5878**	72.0	**7883**	92.0	**9827**	112.0	**11725**	132.0	**13565**
52.5	**5929**	72.5	**7932**	92.5	**9875**	112.5	**11771**	132.5	**13611**
53.0	**5980**	73.0	**7982**	93.0	**9924**	113.0	**11818**	133.0	**13656**
53.5	**6031**	73.5	**8031**	93.5	**9972**	113.5	**11864**	133.5	**13701**
54.0	**6082**	74.0	**8081**	94.0	**10020**	114.0	**11910**	134.0	**13747**
54.5	**6133**	74.5	**8130**	94.5	**10068**	114.5	**11957**	134.5	**13792**
55.0	**6184**	75.0	**8180**	95.0	**10117**	115.0	**12003**	135.0	**13837**
55.5	**6234**	75.5	**8229**	95.5	**10165**	115.5	**12049**	135.5	**13882**
56.0	**6285**	76.0	**8278**	96.0	**10213**	116.0	**12095**	136.0	**13928**
56.5	**6335**	76.5	**8327**	96.5	**10261**	116.5	**12141**	136.5	**13973**
57.0	**6386**	77.0	**8376**	97.0	**10309**	117.0	**12187**	137.0	**14018**
57.5	**6436**	77.5	**8425**	97.5	**10357**	117.5	**12233**	137.5	**14063**
58.0	**6486**	78.0	**8474**	98.0	**10404**	118.0	**12279**	138.0	**14108**
58.5	**6536**	78.5	**8523**	98.5	**10452**	118.5	**12325**	138.5	**14153**
59.0	**6586**	79.0	**8572**	99.0	**10500**	119.0	**12371**	139.0	**14198**
59.5	**6636**	79.5	**8621**	99.5	**10548**	119.5	**12417**	139.5	**14242**

[1] The minimum of the patient's actual weight (kg) or maximum effective mass (kg) from Table 1. For values between 140 kg and 160 kg, use the following formula:

Activity Hours (mCi h) = 14287 + (88.74) (Wt in kg - 140)

Site Number (*GSK will complete*): ____________________
Patient Identification Number: ____________________
Patient Initials (LFM): ____________________

Graph 1
Total Body Residence Time Estimation

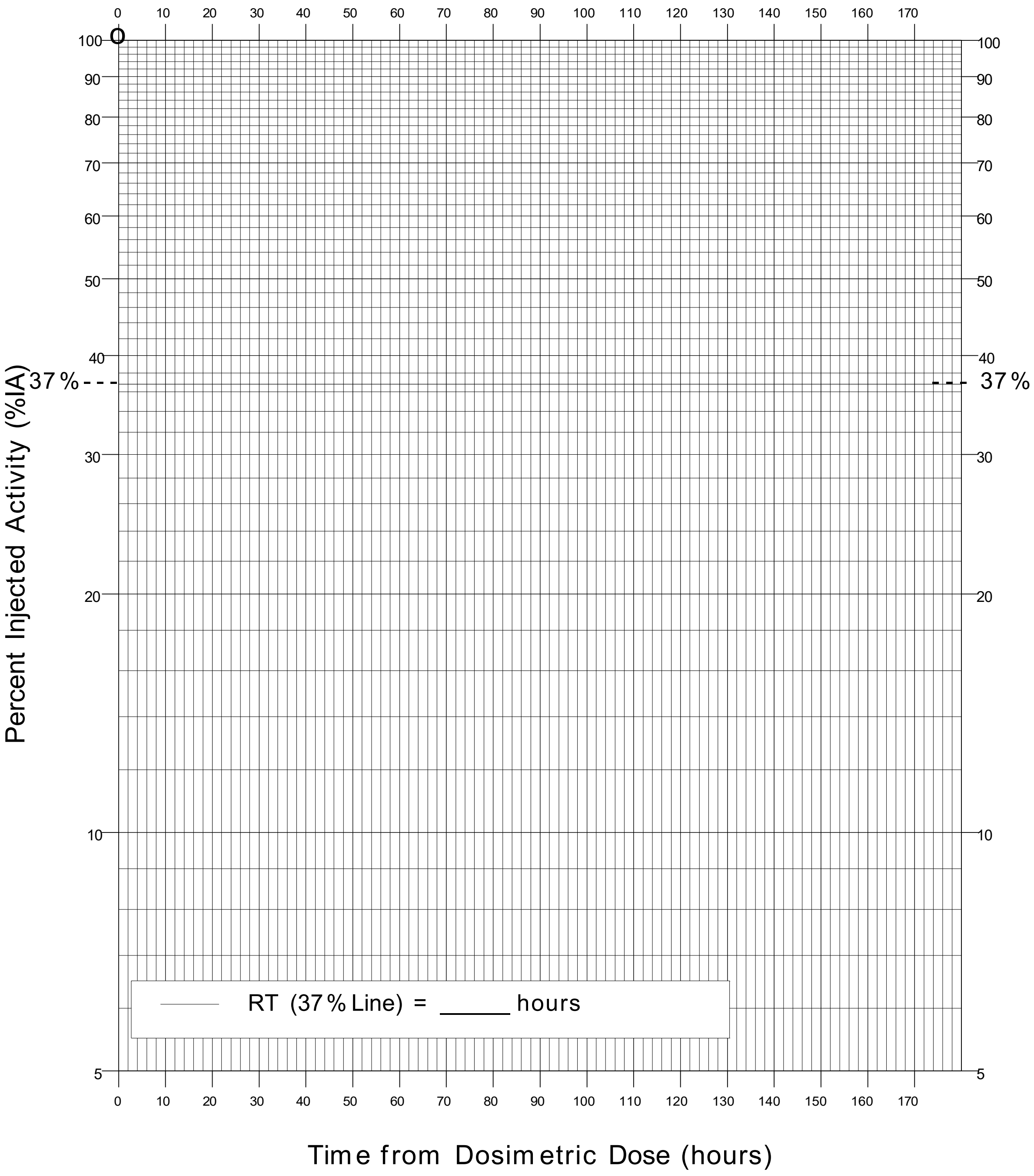

19.2 ASSESSMENT OF BONE MARROW INVOLVEMENT

Pathology procedure for determining percentage of bone marrow involvement with lymphoma by bilateral biopsy.

1. Bone marrow core biopsies are collected bilaterally. Results of a unilateral biopsy are acceptable if that result indicates that less than 10% of the intratrabecular space is lymphoma. The results of reading the two biopsies are averaged and extrapolated to the entire bone marrow. The core biopsy specimens are sectioned longitudinally if they are longer than 0.5 cm in length. Three or 4 of these sections for each biopsy specimen can be positioned on a slide, stained, and read. One slide is prepared per biopsy sample. If a biopsy specimen is 0.5 cm or less in length, then it is sectioned sagittally and about 10 to 12 sections of each biopsy are positioned on a slide, stained, and read. Again one slide is prepared from each specimen.

2. The sections for each patient are generally fixed, embedded, and stained using hematoxylin and eosin. Where the histopathology of the specimen is not clear with H & E, sections of the specimen will be analyzed for cell surface markers by immunohistopathology using antibodies, such as L26, which binds to the CD20 antigen. The monoclonal nature of lymphoma cells cannot be established on fixed specimens, as the kappa and lambda antigens are denatured by fixation.

3. The lymphoma disease of almost all patients occurs as foci of disease cells within the bone marrow. The percentage of the total field areas that are composed of these foci are estimated visually over each section. A few patients have diffuse disease which is more difficult to quantitate, but again the percentage of bone marrow area on sections which is involved with lymphoma is visually estimated. The mean percentage of the total fields consisting of lymphoma cells is calculated for the two biopsy specimens. It is assumed that all bone marrow has a similar level of infiltration with disease as the core biopsies.

CLOSED EFFECTIVE 09/15/2006

SWOG Authorized Document

19.3 a. Ordering Instructions for Tositumomab and Iodine I-131 Tositumomab

b. Study Drug Order Form For **S0016**

SWOG Authorized Document

19.3 a Drug Ordering Instructions for Tositumomab and Iodine I-131 Tositumomab

Step 1: Determining scheduling dates for treatment

- Availability of study drug varies according to the manufacturing schedule. Please contact the Bexxar Service Center at 1/877-423-9927 as far in advance as possible to confirm drug availability on the potential treatment dates.
  - The dosimetric and therapeutic doses are administered 7 to 14 days apart.
  - **Alternate** dates for the dosimetric and therapeutic doses should also be considered.

Step 2: Ordering Product

After the treatment dates have been scheduled, the study/site coordinator completes the first section of the study drug order form and faxes it to **GlaxoSmithKline (fax #: 1-610-917-6119) no later than Wednesday 4pm EST the week prior to scheduled dosimetric dose**.

- GlaxoSmithKline will fax the study drug order form back to the study/site coordinator to confirm initiation of the order.
- The study/site Coordinator then faxes the returned study drug order form, along with the written physician order, to the radiopharmacy.
- GlaxoSmithKline will forward radiopharmacy contact information to the Bexxar Service Center. The Bexxar Service Center will contact the radiopharmacy to complete the order.
-
  - When completing an order, a Bexxar Service Center Consultant will ask the radiopharmacy for the information provided on the Study Drug Order Form.
  - The order must be completed by Thursday 4pm the week prior to the dosimetric dose.
  - The Bexxar Service Center is open 5 days per week (Monday-Friday) from 8am - 6pm (EST). A recording will provide you with alternatives if you contact the Service Center outside of these hours.

Radiopharmacy is the department that will be preparing the radioactive doses for administration. This may be an in-house radiopharmacy, commercial radiopharmacy or someone in the nuclear medicine department.

CLOSED EFFECTIVE 09/15/2008

Step 3: Shipment of Prepared Doses

The nonradiolabeled and radiolabeled components of study drug are supplied from two distinct locations.

- All study drug (both nonradiolabeled and radiolabeled) is shipped to the radiopharmacy.
- The radiopharmacy prepares the dosimetric and therapeutic doses and delivers the doses to the nuclear medicine facility within the treatment center.
- The radiopharmacy also delivers the nonradiolabeled components of the study drug to the pharmacy at the treatment center.

Step 4: Treating the Patient

The patient should be treated with the dosimetric and therapeutic doses of study drug according to the scheduled treatment dates.

- The estimated mCi requirements for the therapeutic dose should be calculated based on results of the **first and second** gamma-camera whole-body counts.

  The radiopharmacy or study/site coordinator must contact the Bexxar Service Center with the estimated mCi requirements to determine if a second vial of study drug may be necessary.

- The actual mCi requirements for the therapeutic dose should be calculated based on results of **all three** gamma-camera whole-body counts and forwarded to the radiopharmacy, along with the written physician order, so that the dose may be prepared.
- Check the product specification sheet, provided with the drug shipment, for expiration date. **DO NOT USE STUDY DRUG AFTER EXPIRATION DATE.**

For answers to any questions, please contact the Service Center at 1-877-423-9927

Study Drug Order Form

For Southwest Oncology Group Protocol **S0016**
(GSK Protocol # 393229/002)

Study/Site Name: ____________________ **Date:** ____________
CCOP Name (if applicable): ____________________

Study/Site Coordinator
(Name, Phone # and Fax #): ____________________

Radiopharmacy Name: ____________________
Address, City, State:
Phone #: ____________________ Fax #: ____________________

Prescribing Physician Name: ____________________
Address, City, State: ____________________

SWOG Patient Identification Number: ____________________

Site Name Where Nonradiolabeled Drug will be administered:
____

Dosimetric: Date: Time: Therapeutic: Date: Time:
Dx Alternate Date(s): Tx Alternate Date(s):

To complete order placement:

1. **Place order by faxing form to GSK R & D at (610) 917-6119.**
2. **GSK R & D will fax order confirmation back to the site and will forward the order onto the Bexxar Service Center (BSC).**
3. **The study/site coordinator then faxes the returned study drug order form, along with the written physician order, to the radiopharmacy.**
4. **The BSC will follow-up with the radiopharmacy.**
5. **Your radiopharmacy can call the BEXXAR Service Center (877) 423-9927 if additional scheduling requirements are needed.**

****

Study Drug Order Form received by GSK: Date: ____________________

- -

Estimated Tx mCi requirement (based on Gamma-Scan Results of Count #2): ____________
This is only an estimate for ordering the study drug. Contact Radiopharmacy with this mCi estimate as soon as available. They will relay the mCi requirement to the BEXXAR Service Center for confirming the order. **These estimates should not be used for the final therapeutic preparation.**

Stop here for order confirmation. Contact radiopharmacy with mCi requirements.

- -

Actual Tx mCi requirement (based on Gamma-Scan results of Count #3): ____________

Prescribing Physician Signature (optional): ____________________

SWOG Authorized Document

S0016 SITE CONTACT INFORMATION FORM

(GSK Protocol # 393229/002)

Medical Oncology Investigator Information	
**Name:**	
**Institution:**	
**CCOP affiliate if applicable:**	
**Address:**	
**Address:**	
**City:**	**State:** **Zip:**
**Telephone:**	**Fax:**
**Email:**	

Research Nurse/Study Coordinator (for this study):
Tel: ______________________________ **Fax:** ___________________ **Email:** ___________________

Nuclear Medicine/Radiation Oncology/Radiopharmacy Information

1) Which department will administer the radiolabeled component of Bexxar®?

□ **Nuclear Medicine** □ **Radiation Oncology**

**Contact Name:**	
**Phone:**	**Fax:**
**E-mail**	

2) Does your current radioactive materials license allow you to have up to 300 mCi of Liquid Iodine 131 (not capsules)? □ **Yes** □ **No**

If no, please explain:

3) Who will prepare the Bexxar® dose?
□ **Commercial Radiopharmacy** _____________________________ □ **Onsite Radiopharmacy**
Name of Radiopharmacy

(Provide name of appropriate contact person in Commercial or Onsite Radiopharmacy)

Contact Name: _________________________
Phone: ________________________________ Fax: __________________________
E-mail: ________________________________

4) Does the Gamma Camera(s) have whole body scanning capability?
□ **Yes** □ **No**

Collimator: □ **High Energy** □ **Medium Energy**
Manufacturer ________________ **Model Number** ___________________

5) Has your institution implemented the new Nuclear Regulatory Commission (NRC) guidelines for release of patients administered radioactive materials?

□ **Yes** □ **No** □ **Would like to implement, need assistance**

PLEASE FAX THIS FORM AND THE RADIOACTIVE MATERIALS LICENCE FOR YOUR INSTITUTION AND FOR THE COMMERCIAL RADIOPHARMACY (IF APPLICABLE) TO GSK AT 610-917-6119

SWOG Authorized Document

19.5 Determination of Expedited Adverse Event Reporting Requirements

Adverse event data collection and reporting, which are required as part of every clinical trial, are done to ensure the safety of patients enrolled in the studies as well as those who will enroll in future studies using similar agents. Adverse events are reported in a routine manner at scheduled times during a trial. (Directions for routine reporting are provided in Section 14.0.) Additionally, certain adverse events must be reported in an expedited manner to allow for more timely monitoring of patient safety and care. Expedited adverse event reporting principles and general guidelines follow; specific guidelines for expedited adverse event reporting on this protocol are found in Section 16.0.

Reporting requirements may include the following considerations: 1) whether the patient has received an investigational or commercial agent; 2) the characteristics of the adverse event including the *grade* (severity), the *relationship to the study therapy* (attribution), and the *prior experience* (expectedness) of the adverse event; 3) the Phase (1, 2, or 3) of the trial; and 4) whether or not hospitalization or prolongation of hospitalization was associated with the event.

An investigational agent is a protocol drug administered under an Investigational New Drug Submission (IND). In some instances, the investigational agent may be available commercially, but is actually being tested for indications not included in the approved package label.

Commercial agents are those agents not provided under an IND but obtained instead from a commercial source. The NCI, rather than a commercial distributor, may on some occasions distribute commercial agents for a trial.

When a study includes both investigational and commercial agents, the following rules apply.

- *Concurrent administration*: When an investigational agent(s) is used in combination with a commercial agent(s), the combination is considered to be investigational and expedited reporting of adverse events would follow the guidelines for investigational agents.
- *Sequential administration*: When a study includes an investigational agent(s) and a commercial agent(s) on the same study arm, but the commercial agent(s) is given for a period of time prior to starting the investigational agent(s), expedited reporting of adverse events that occur prior to starting the investigational agent(s) would follow the guidelines for commercial agents. Once therapy with the investigational agent(s) is initiated, all expedited reporting of adverse events should follow the investigational guidelines.

Steps to determine if an adverse event is to be reported in an expedited manner

Step 1: *Identify the type of event using the NCI Common Terminology Criteria for Adverse Events (CTCAE).* The CTCAE provides descriptive terminology and a grading scale for each adverse event listed. A copy of the CTCAE can be downloaded from the CTEP home page (http://ctep.cancer.gov). Additionally, if assistance is needed, the NCI has an Index to the CTCAE that provides help for classifying and locating terms. All appropriate treatment locations should have access to a copy of the CTCAE.

Step 2: *Grade the event using the NCI CTCAE version specified.*

CLOSED EFFECTIVE 09/15/2008

SWOG Authorized Document

Step 3: *Determine whether the adverse event is related to the protocol therapy (investigational or commercial).* Attribution categories are as follows: Unrelated, Unlikely, Possible, Probable, and Definite.

Step 4: *Determine the prior experience of the adverse event.* Expected events are those that have been previously identified as resulting from administration of the agent. An adverse event is considered *unexpected,* for expedited reporting purposes only, when either the type of event or the severity of the event is not listed in

- the current NCI Agent-Specific Adverse Event List (for treatments using agents provided under an NCI-held IND);
- the drug package insert (for treatments with commercial agents only);
- Section 3.0 of this protocol.

Step 5: *Review Tables 16.1 and 16.2 in the protocol to determine if there are any protocol-specific requirements for expedited reporting of specific adverse events that require special monitoring.*

Step 6: *Determine if the protocol treatment given prior to the adverse event included an investigational agent(s), a commercial agent(s), or a combination of investigational and commercial agents.*

Note: If the patient received at least one dose of investigational agent, follow the guidelines in Table 16.1. If no investigational agent was administered, follow the guidelines in Table 16.2.

Note: This includes all events that occur within 30 days of the last dose of protocol treatment. Any event that occurs more than 30 days after the last dose of treatment and is attributed possibly, probably, or definitely to the agent(s) must be reported accordin

CLOSED EFFECTIVE 09/15/2008

SWOG Authorized Document

19.6 Cancer Trials Support Unit (CTSU) Participation Procedures

Registration/Randomization

Prior to the recruitment of a patient for this study, investigators must be registered members of the CTSU. Each investigator must have an NCI investigator number and must maintain an 'active' investigator registration status through the annual submission of a complete investigator registration packet (FDA Form 1572 with original signature, current CV, Supplemental Investigator Data Form with signature, and Financial Disclosure Form with original signature) to the Pharmaceutical Management Branch, CTEP, DCTD, NCI. These forms are available on the CTSU registered member website or by calling the PMB at 301/496-5725 Monday through Friday between 8:30 a.m. and 4:30 p.m. EST.

Each CTSU investigator or group of investigators at a clinical site must obtain IRB approval for this protocol and submit IRB approval and supporting documentation to the CTSU Regulatory Office before they can enroll patients. Study centers can check the status of their registration packets by querying the Regulatory Support System (RSS) site registration status page of the CTSU member web site at https://www.ctsu.org

All forms and documents associated with this study can be downloaded from the **S0016** web page on the CTSU registered member web site (https://www.ctsu.org). Patients can be registered only after pre-treatment evaluation is complete, all eligibility criteria have been met, and the study site is listed as 'approved' in the CTSU RSS.

Requirements for **S0016 site registration**:
- CTSU IRB Certification
- CTSU IRB/Regulatory Approval Transmittal Sheet

Prior to patient registration, sites must be approved by GlaxoSmithKline for the delivery of I-131 therapy. The site must fax the S0016 Site Contact Information Form and a radioactive materials license to GlaxoSmithKline (see Section 19.4). If approved, GlaxoSmithKline will send the site an approval notice.

Prestudy requirements for **patient enrollment** on **S0016**:
- Patient must meet all inclusion criteria, and no exclusion criteria should apply.
- Patient has signed and dated all applicable consents and authorization forms.
- All baseline laboratory tests and prestudy evaluations performed.
- Approval notice from GlaxoSmithKline for delivery of I-131 delivery.

CTSU Procedures for Patient Enrollment

1. Contact the CTSU Patient Registration Office by calling 1-888/462-3009. Leave a voicemail to alert the CTSU Patient Registrar that an enrollment is forthcoming. For immediate registration needs, e.g. within one hour, call the registrar cell phone at 301/704-2376.

2. Complete the following forms:
   - CTSU Patient Enrollment Transmittal Form
   - Eligibility Criteria Checklist (Section 5.0 of the protocol)
   - SWOG Registration Form (Complete all sections of form except for SWOG-specific data fields)

3. Fax these forms to the CTSU Patient Registrar at 1-888-691-8039 between the hours of 8:00 a.m. and 8:00 p.m., Mon-Fri, Eastern Time (excluding holidays). The CTSU registrar will check the investigator and site information to ensure that all regulatory requirements have been met. The registrar will also check that forms are complete and follow-up with the site to resolve any discrepancies.

CLOSED EFFECTIVE 09/15/2008

SWOG Authorized Document

4. Once investigator eligibility is confirmed and enrollment documents deemed complete, the CTSU registrar will contact the Southwest Oncology Group to obtain assignment of a treatment arm and assignment of a unique patient ID (to be used on all future forms and correspondence). The CTSU registrar will convey this information to the enrolling site and follow up with a confirmation via e-mail or fax.

   If a patient is randomized to Arm 3, GlaxoSmithKline will contact the site shortly after randomization to provide assistance and to arrange for an on-site training session for Iodine I-131 therapy. Drug orders will **not** be processed without the required site training (see protocol Section 15.0 for further details).

Patients must be registered prior to initiation of treatment (no more than one working day prior to planned start of treatment.)

Data Submission and Reconciliation

1. All case report forms (CRFs) associated with this study must be downloaded from the **S0016** web page located on the CTSU registered member website (https://www.ctsu.org). Sites must use the current form versions and adhere to the instructions and submission schedule outlined in the protocol.

2. Submit all completed CRFs (with the exception of patient enrollment forms), clinical reports, and transmittals directly to the SWOG Data Operations Center. The preferred method of sending data is via fax at 800/892-4007, (large volumes of data may be sent via post, see contacts table for mailing address). Do NOT include a cover sheet for faxed data.

3. The SWOG Data Operations Center will send query notices and delinquency reports directly to the site for reconciliation. Please fax query responses and delinquent data to the SWOG Data Operations Center and do not copy the CTSU Data Operations. When faxing data, include the query sheet that was originally sent from SWOG.

4. Each site should have a designated CTSU Administrator and Data Administrator and **must keep their CTEP AMS account contact information current.** This will ensure timely communication between the clinical site and the SWOG data center.

Special Materials or Substudies

Sites enrolling through the CTSU will not be participating in SWOG-8947 or SWOG-8819.

1. All specimens submitted for this study must be entered and tracked using the SWOG on-line Specimen Tracking System, as specified in protocol Sections 12.0 and 15.0.

2. You can also access the Tracking System from the CTSU Member Web Site. Go to the **S0016** protocol page and click on the link provided under the Case Report Forms header.

3. Pathology Review
   - Collect, prepare, and submit specimens as outlined in protocol Section 12.0.
   - Do not send specimens, supporting clinical reports, or transmittals to the CTSU.

4. Specimen Submission
   - Submit bone marrow as outlined in Section 15.4.
   - Submit serum for HAMA testing as outlined in Section 15.5.

CLOSED EFFECTIVE 08/15/2008

SWOG Authorized Document

Serious Adverse (AE) Reporting (Section 16.0)

1. CTSU sites must comply with the expectations of their local Institutional Review Board (IRB) regarding documentation and submission of adverse events. Local IRBs must be informed of all reportable serious adverse reactions.

2. CTSU sites will assess and report adverse events according to the guidelines and timelines specified in the protocol. You may navigate to the CTEP Adverse Event Expedited Report System (AdEERS) from either the Adverse Events tab of the CTSU member homepage (https://members.ctsu.org) or by selecting Adverse Event Reporting Forms from the document center drop down list on the **S0016** web page.

3. Do not send adverse event reports to the CTSU.

4. Secondary AML/MDS/ALL reporting: Report occurrence of secondary AML, MDS, or ALL via the NCI/CTEP AML-MDS Report Form in lieu of AdEERS. Submit the completed form and supporting documentation as outlined in the protocol.

Drug Procurement (Section 3.0)

Information on drug formulation, procurement, storage and accountability, administration, and potential toxicities are outlined in Section 3.0 of the protocol.

Investigational agents: Iodine I-131 labeled monoclonal anti-B1 antibody (tositumomab)

- Complete the Study Drug Order Form for S0016 (see Appendix 19.3) and fax it to GlaxoSmithKline Clinical Research Department. The order form must be received by Wednesday 4:00 p.m. EST prior to the treatment week. See protocol Section 3.5c for complete details.

Commercial agents: Cyclophosphamide, Doxorubicin, Prednisone, Vincristine, Rituximab chimeric anti-CD20 monoclonal antibody

- These drugs are commercially available and will not be supplied free of charge.

Regulatory and Monitoring

Study Audit

To assure compliance with Federal regulatory requirements [CFR 21 parts 50, 54, 56, 312, 314 and HHS 45 CFR 46] and National Cancer Institute (NCI)/Cancer Therapy Evaluation Program (CTEP) Clinical Trials Monitoring Branch (CTMB) guidelines for the conduct of clinical trials and study data validity, all protocols approved by NCI/CTEP that have patient enrollment through the CTSU are subject to audit.

Responsibility for assignment of the audit will be determined by the site's primary affiliation with a Cooperative Group or CTSU. For Group-aligned sites, the audit of a patient registered through CTSU will become the responsibility of the Group receiving credit for the enrollment. For CTSU Independent Clinical Research Sites (CICRS), the CTSU will coordinate the entire audit process.

For patients enrolled through the CTSU, you may request the accrual be credited to any Group for which you have an affiliation provided that Group has an active clinical trials program for the primary disease type being addressed by the protocol. (e.g., NSABP members may only request credit for protocols pertaining to breast or colorectal cancers). Registrations to protocols for other disease sites may still take place through CTSU without receiving credit for your NSABP activities. Per capita reimbursement will be issued directly from CTSU.

CLOSED EFFECTIVE 09/15/2008

SWOG Authorized Document

Details on audit evaluation components, site selection, patient case selection, materials to be reviewed, site preparation, on-site procedures for review and assessment, and results reporting and follow-up are available for download from the CTSU Operations Manual located on the CTSU Member website.

Health Insurance Portability and Accountability Act of 1996 (HIPAA)

The HIPAA Privacy Rule establishes the conditions under which protected health information may be used or disclosed by covered entities for research purposes. Research is defined in the privacy rule referenced in HHS 45 CFR 164.501. Templated language addressing NCI-U.S. HIPAA guidelines are provided in the HIPAA Authorization Form located on the CTSU website.

The HIPAA Privacy Rule does not affect participants from outside the United States. Authorization to release protected health information is NOT required from patients enrolled in clinical trials at non-US sites.

Clinical Data Update System (CDUS) Monitoring

This study will be monitored by the Clinical Data Update System (CDUS) Version 3.0. Cumulative CDUS data will be submitted quarterly to CTEP by electronic means. The sponsoring Group fulfills this reporting obligation by electronically transmitting to CTEP the CDUS data collected from the study-specific case report forms.

CLOSED EFFECTIVE 09/15/2008

SWOG Authorized Document

19.7 HAMA assay - Methods

Human antibodies to mouse immunoglobulins are known as HAMA (Human Anti-Mouse Antibody). We will use the UBI MAGIWEL™ HAMA kit, which is an enzyme immunoassay that provides for the sensitive detection and quantitation of HAMA (United Biotech Inc., Mountain View, CA, www.unitedbiotech.com, cat.no. RR-101). This HAMA kit comes with all reagents, ready to use, including standards and a positive control. Test samples are diluted and quantified in mcg HAMA/mL of sample from the standard curve. Test samples and an enzyme-linked mouse IgG are added to coated wells of a 96-well microplate. A chromogen-substrate is added and the enzyme reaction stopped with the absorbance read at 450 nm. We will use the Bioteck Synergy HT Multi-Detection Microplate Reader (Biotek Instruments, Inc. Winooski, Vermont).

The assays will be performed in the SWOG Lymphoma Translational Medicine lab of Dr. Lisa Rimsza (University of Arizona). Dr. Rimsza is a clinical pathologist, whose laboratory currently uses enzyme immunoassays for several research projects. A list of published references that involve the quantification of basic fibroblastic growth factor, vascular endothelial growth factor, and endostatin using similar technology is provided below.

1. Rimsza L, Ahrens, K, Mainwaring M, et al. Both freshly isolated hematologic tumor cells and cell lines stimulate endothelial growth: this effect correlates with VEGF levels in the cell lines only. Blood 2000; 96(11):58B.

2. Rimsza L, Pastos K, Lynch J, et al. Conditioned media from CLL and SLL stimulates *in vitro* endothelial cell proliferation and decreases endostatin generation by endothelial cells, both likely mediated by b-FGF. Blood 2001; 98(11):361A.

3. Rimsza L, Pastos K, Massey J, et al. Endothelial stimulation by small lymphocytic lymphoma correlates with secreted levels of basic fibroblastic growth factor. British J Haem 2003; 120(5):753-8.

4. Stopeck A, Iannone M, Rimsza L, et al. Expression of VEGF, VEGF receptors, and other angiogenic markers in relapsed aggressive NHL: correlative studies from SWOG S0108 Trial. Blood 2004; 104(11):629A.

CLOSED EFFECTIVE 09/15/2008

SWOG Authorized Document

Summary of Protocol Amendments

Amendment 1; November 1, 2001
Amendment 2; November 15, 2004
Amendment 3; February 15, 2006
Amendment 4; December 15, 2006
Amendment 5; April 15, 2007

AMENDMENT #1
November 1, 2001
The study protocol has been amended as follows:
1. Dr. Press's contact information has been updated on the title page.
2. The supplier information for tositumomab has been updated in Section 3.5c and new drug ordering instructions inserted. A new section titled, "Drug Return" has been added to this section.
3. The Nuclear Medicine Physician/Radiation Oncology Investigator Questionnaire has been replaced with the **S0016** Site Contact Information Form.
4. "TSH" has been inserted into Section 7.1 as item "e" and an "X ß" placed in the prestudy column for TSH on the study calendar, Section 9.3.
5. The first bolded paragraph in Sections 7.2 and 7.5 have been rewritten to indicate that Corixa will contact the institution soon after their first patient is randomized to Arm 3 to provide assistance and to make arrangements for on-site training, if required.
6. The word, "therapeutic" replaces the word, "radioimmunotherapy" throughout Section 7.5 for clarification. A statement referencing Section 3.5c has been added to the end of Section 7.5c.1 regarding return of unused drug.
7. The phrase, "upon request (contact the Service Center at 877/423-9927)" has been inserted into the third sentence of Section 7.5c.2iv and into the fifth sentence of Section 7.5c.3ii.
8. The phrase, "(Service Center, toll free 877/423-9927)" replaces the phone number for Teresa White in the last paragraph of Section 7.5c.2v and in Appendix 19.1a.2b. The numbering of Section 19.1d has been corrected to read 19.1c.
9. The word "therapeutic" replaces the word "radioimmunotherapeutic" in Section 8.4b and the word "tachycardia" has been inserted into the second sentence of this section.
10. In the study calendar, Section 9.3, the "ß" footnote has been removed from the Day 133 column (urinalysis and uric acid) as it is not applicable. Additionally, the name "Coulter" has been replaced with "Corixa" in the required studies column. The word "therapeutic" replaces "radioimmunotherapy" in the "¥" footnote. Also, the "¶" footnote has been reworded for clarification.
11. The statistical section has been updated as follows:
    a. The phrase, "one-sided level .024" has been replaced with "one-sided level .012" in the first paragraph of Section 11.2. Also, the phrase, "power of .92" has been replaced with "power of .87".
    b. A new second paragraph has been added to Section 11.2 providing information and clarification about the overall survival endpoint of the study. Additionally, a new third paragraph has been added to provide clarification regarding stratification on the study.
    c. The phrase, "rejected at the .0024 level" has been replaced with "rejected at the .0025 level" in Section 11.3. Additionally, the last sentence of this section has been revised to state, "If the study is not terminated early or the hypothesis tests at the time of closure are not rejected, the primary analysis on progression-free survival will be completed after approximately 2 years of follow-up after closing randomization at the .01 one-sided level for the control vs. experimental comparisons."
12. Section 15.1 has been revised to indicate that Corixa will contact the institution shortly after their first patient is randomized to Arm 3 to provide assistance and arrange for on-site training, if required. Also, the former questionnaire has been replaced with the **S0016** Site Contact Information Form in this section.
13. The phrase "through your vein" has been inserted into the 4th sentence of the 5th paragraph of the "What Is Involved In This Study?" section of the consent form for clarification. The phrase "treatment dose of the antibody" replaces the phrase "antibody treatment" in the last sentence of this paragraph. The word "treatment" replaces the

word "therapeutic" in the first sentence of the 6th paragraph of this section. Also, the second sentence of the 6th paragraph has been reworded to clarify when the potassium iodide is received.
14. To be consistent with the updated investigator's brochure for tositumomab, six bulleted items have been added to the "Less Likely" risks section for tositumomab and 4 bulleted items have been added to the "Less Likely, but Serious" section.
15. New "Ordering Instructions for Tositumomab and Iodine I-131 Tositumomab" and a new "Study Drug Order Form for **S0016**" have been inserted into Section 19.3 as Sections 19.3a and 19.3b.
16. The **S0016** Site Contact Information Form replaces the old Nuclear Medicine Physician Questionnaire in Section 19.4 and the title has been updated on the appendix page, page 78.

AMENDMENT #2
November 15, 2004
The protocol has been revised as follows:
1. (Title page): The version date has been updated.
2. (Pages 5, Section 2.0): The rituximab background section has been amended to incorporate language regarding Hepatitis B virus (HBV) reactivation.
3. (Page 20, Section 5.20): The sentence "Patients at high risk of Hepatitis B virus infection should be screened before initiation of rituximab" has been added to this eligibility section.
4. (Page 22, Sections 7.1 and 7.2): In Section 7.1, Hepatitis B virus screening has been added to the Good Medical Practice section as item "f". In the first bolded paragraph of Section 7.2, the underlined sentences regarding instructions for institutions that have patients randomized to Arm 3 have been deleted and replaced with the following information:
"A protocol specific training is required for all institutions with patients randomized to Arm 3. In addition, members of the treatment team are required to participate in the general Bexxar training program. Drug orders can not be processed without this requirement being met." The second bolded paragraph has been deleted in its entirety.
5. (Page 28, Sections 7.5c2.iv): The second sentence has been edited to read, "The unlabeled antibody must be administered through a 0.22 micron in-line filter." All references to an Abbott lab filter set, etc., have been deleted from this sentence. In the following sentence, "these filters will" has been changed to "these filters can".
6. (Page 29, Section 7.5c3.ii): The fourth sentence has been edited to read, "The unlabeled antibody must be administered through a 0.22 micron in-line filter." All references to an Abbott lab filter set, etc., have been deleted from this sentence. In the following sentence, "these filters will" has been changed to "these filters can".
7. (Page 32, Section 8.3b): A new paragraph was added at the end of the rituximab dose modification section. This new paragraph contains information about HBV precautions.
8. (Page 35, Section 9.2): Under laboratory evaluations, "HBV screening" has been added to the list of tests suggested for prestudy and follow-up as part of Good Medical Practice. The "◊" footnote corresponding to this test specifies that this test is "recommended for patients at high risk of HBV infection."
9. (Page 44, Section 15.1): The bolded NOTE section (fourth paragraph) has been updated with new information regarding Corixa approval and training prior to I- 131 treatment.
10. (Page 47a, Section 16.1g): Revised guidelines for reporting secondary AML and MDS have replaced the original section.
11. (Page 55 and 59a, Model Consent Form, WHAT IS INVOLVED IN THE STUDY?) have been updated to describe precautions and procedures for HBV testing.

AMENDMENT #3
Protocol Distribution Date: February 15, 2006
CTEP Submission Date: February 1, 2006
The study has been revised as follows:
1. The following cooperative group has endorsed this trial via CTSU: Eastern Cooperative Oncology Group (ECOG), Study Chair: Sandra Horning, M.D.
2. (Title page): The following changes have been made to this page:
• The version date has been updated (Version Date 02/15/06).
• The page numbers in the table of contents have been updated.
• CTSU has been added to the participant list.

• Under the participant list, the following text was added: "Patient enrollments from institutions that are not aligned with SWOG or CALGB will be conducted via the NCI Cancer Trials Support Unit (CTSU) and all data should be sent to CTSU Data Operations unless otherwise specified in the CTSU logistical appendix (see Section 19.6)."

3. (Page 1a): The following changes have been made to this page:

• The contact information for the ECOG study coordinator, Sandra Horning, M.D. has been added.

• The CTSU contact information table has been added.

4. (Pages 19 - 20, Sections 5.8 - 5.9): In Section 5.8 the abdomen and pelvis CT scan date blank has been relocated under Section 5.9.

5. (Page 31, Section 8.2a): The following changes have been made to this section:

• First paragraph, last sentence: "In this study, G-CSF or GM-CSF will not be administered to prevent neutropenia" has been changed to "In this study, *growth factors* will not be administered..."

• Second paragraph, first sentence: "...G-CSF or GM-CSF may be added..." has been changed to "...*growth factors may be added...*"

• Third paragraph: The third paragraph has been revised to the following:

"Pegfilgrastim (pegylated G-CSF), filgrastim (G-CSF), and sargramostim (GMCSF) are acceptable growth factors. These growth factors are commercially available and should be purchased through third party mechanisms. The Southwest Oncology Group will not provide growth factors for this study."

• Fourth paragraph: The bolded paragraph that begins "The use of G-CSF or GM-CSF must be documented..." has been removed.

6. (Page 35, Section 9.2): The following changes have been made to this page:

• History & Physical Exam: Day 48, Day 90, Day 134, and Day 141 have been removed. The number of visits has been decreased as physical exams should be performed once per cycle.

• Bone marrow aspirate/biopsy: Day 365 biopsy at restaging has been removed (only required at Day 200 if initially involved with lymphoma).

• HBV screening: Follow-up screening has been removed.

7. (Page 36, Section 9.3): Under bone marrow aspirate/biopsy, the Day 365 biopsy at restaging has been removed (only required at Day 200 if initially involved with lymphoma).

8. (Page 40, Sections 12.0): The pathology review section has been replaced with updated instructions. Highlights of this section include the following: (1) Dr. Lisa Rimsza replacing Dr. Thomas Grogan throughout this section; and (2) Removal of the Pathology Submission Form as all specimen submissions should be executed using the SWOG Online Specimen Tracking System.

9. (Page 43, Section 14.0): The following changes have been made to this page:

• Section 14.4a: The Non-Hodgkin's Lymphoma (Follicular) Prestudy Form has been updated to a study specific form (Form #55363).

• Section 14.4d: This section has been removed. The study specific flow sheets have been removed from this study. This section has been replaced with the following: "Submit bone marrow for bcl2 assessment to Dr. Rita Braziel per Section 15.4."

• Section 14.5: This section has been updated to the following: "Submit histopathologic materials along with a copy of the pathology reports to Dr. Rimsza (see Section 12.0)."

• Section 14.6: This section has been replaced with the following:

"FOR PATIENTS ON THE I-131 TOSITUMOMAB ARM (ARM 3), AFTER EVERY CYCLE OF CHOP AND AFTER COMPLETION OF I-131 TOSITUMOMAB TREATMENT: Submit the **S0016** Tositumomab Treatment Form (Form #57993) and the **S0016** Adverse Event Form (Form #41405)."

• Section 14.7: A new section has been added:

"FOR PATIENTS ON THE R-CHOP ARM (ARM 2), AFTER EVERY CYCLE OF RCHOP: Submit the **S0016** CHOP/R-CHOP Treatment Form (Form #30359) and the **S0016** Adverse Event Form (Form #41405). "

• Section 14.8: A new section has been added:

"FOR EITHER ARM, 3 MONTHS AFTER REMOVAL FROM PROTOCOL TREATMENT: Submit the **S0016** Adverse Event Form (Form #41405)."

• Subsequent sections have been renumbered and final form numbers will be inserted upon CTEP approval of these changes.

• Section 14.10: "Submit a copy of the Southwest Oncology Group Follow-Up Form..." has been changed to "Submit a copy of the Follow-Up Form (Form #1512)."

• Section 14.11: This section has been updated to the following: "Submit bone marrow for bcl2 assessment to Dr. Rita Braziel per Section 15.4."
• Section 14.12: This section has been revised as follows: "Submit a copy of the Follow-Up Form (Form #1512)." The study specific flow sheets have been removed from this study.
• Section 14.13: This section has been revised as follows: "Submit a copy of the Off Treatment Notice (Form #22204)." The study specific flow sheets have been removed from this study.
• Section 14.14: This section has been revised as follows: "Submit a copy of the Follow-Up Form (Form #1512) (if death occurs after off treatment) and a copy of the Notice of Death (Form #1821)."
10. (Pages 44-45b, Sections 15.2-15.5): Updated instructions for specimen collection and shipping (Section 15.2-15.5) have replaced the original Sections 15.2-15.3. Page 45b was added to prevent extensive repagination.
11. (Page 47, Table 16.1): Formatting changes have been made to this page, but the contents remain unchanged.
12. (Page 47a, Section 16.1g): The address for the Investigational Drug Branch has been removed. Institutions should continue to fax documentation.
13. (Page 51, Section 18.2): The following changes have been made to this page:
• Section 18.2b: The Non-Hodgkin's Lymphoma (Follicular) Prestudy Form has been updated to a study specific form (Form #55363).
• Section 18.2d: The study specific flow sheet has been removed from this study. This has been replaced with the **S0016** Tositumomab Treatment Form (Form #57993*).*
• Section 18.2e: The **S0016** CHOP/R-CHOP Treatment Form (Form #30359*)* has been added.
• Section 18.2f: The **S0016** Adverse Event Form (Form #41405*)* has been added.
• Section 18.2g: The Study Specific Pathology Submission Form has been removed.
• Section 18.2h: The Southwest Oncology Group Specimen Submission Form has been removed. Subsequent sections have been reorganized.
• Section 18.2i: "Southwest Oncology Group Follow-Up Form (Form #1512)" has been changed to "Follow-Up Form (Form #1512)".
• Version dates have been included where appropriate.
14. (Page 53, Model Informed Consent): The accrual goal of 775 has been changed to 500 to reflect the two open arms of this study.
15. (Pages 55, Model Consent Form): The following change has been made to this page:
• Last paragraph, first sentence: "You will also have your bone marrow examined (called "bone marrow aspiration and biopsy") at the start of the study, four to eight weeks after completion of therapy, one year after treatment, and annually thereafter as long as your lymphoma does not get worse" has been changed to the following:
"You will also have your bone marrow examined (called "bone marrow aspiration and biopsy") at the start of the study, four to eight weeks after completion of *CHOP* therapy *(if you are assigned to Arm 3)*, *and 200 days after beginning treatment (this is required only if lymphoma cells are present in your bone marrow at prestudy)***.**"
16. (Page 56, Model Consent Form): In the first paragraph, second sentence, "In addition, a portion of the bone marrow taken at the start of the study and again at one year after you finish treatment will be sent to a special laboratory..." has been changed to "In addition, a portion of the bone marrow *will be* taken at the start of the study and again at one year after *beginning* treatment*, and* will be sent to a special laboratory..."
17. (Page 60, Model Consent Form): Under "What about confidentiality", the Cancer Trials Support Group (CTSU) has been to the list of organizations that may inspect and/or copy research records for quality assurance and data analysis.
18. (Pages 81-99, Section 19.0): Section 19.6 "Cancer Trials Support Unit (CTSU) Participation Procedures" was added to Section 19.0. Also, due to the addition of new forms and removal of the study specific flow sheets (as outlined in #7 above) repagination was required (pages 81-99). The CTSU participation procedures were appended as pages 100-103.

AMENDMENT #4
Distributed: December 15, 2006
Submitted to CTEP: November 21, 2006
The study protocol has been amended as follows:
1. (Fast Fact Sheet): Under the eligibility column, first row, seventh line, the following sentence has been added: "*Must agree to the serum sample submission schedule for HAMA testing as outlined in Section 15.5.*"

2. (Title page): The version date has been updated (11/21/06). Also, the table of contents has been updated.
3. (Page 3, Section 1.5, Objectives): The following new objective has been added:
"*To determine the incidence and time to development of human anti-mouse antibody (HAMA) positivity.*"
4. (Pages 6 - 7a, Section 2.0, Background): A new background section titled "*HAMA with Tositumomab/Iodine I-131 Tositumomab (BEXXAR®)"* has been added (Page
6). Due to the inclusion of new references (citations 24-26; see also bibliography changes), subsequent citations have been renumbered. Page 7a has been added to prevent extensive repagination.
5. (Page 7, Section 2.0, Background): The following changes have been made to his page:
• The paragraph that begins with "Other groups have also investigated..." has been titled "*High Dose Therapy with I-131 Tositumomab*".
• The paragraph that begins with "A similar series of trials have been conducted..." has been titled "*Studies With Other Radioimmunoconjugates*".
• The paragraph that begins "Witzig has recently presented an interim analysis..." has been changed to "Witzig has presented an interim analysis..." In the above paragraph, the second sentence that ends with "...but only 90 patients were evaluable at the time of the interim analysis in December, 1999" has been changed to ""...but only 90 patients were evaluable at the time of the interim analysis." *("...in December, 1999" has been removed.)*
6. (Page 13, Section 3.5c, Drug Information): Under the formulation information for anti-B1 antibody, the second sentence "...Anti-B1 antibody is a sterile, clear, colorless liquid supplied in a 3 ml or 20 ml glass vial stoppered with a gray silicone coated butyl rubber stopper..." has been revised. The term "*stoppered*" has been removed from this sentence.
7. (Page 14, Section 3.5c, Drug Information): Under the formulation information for iodine I-131 anti-B1 antibody, the first sentence "The Iodine-131 Anti-B1 antibody is a sterile, colorless liquid in a glass vial stoppered with a gray silicone-coated butyl rubber stopper..." has been revised. The term "*stoppered*" has been removed
from this sentence.
8. (Page 19, Section 5.0, Eligibility Criteria): In the first paragraph under eligibility criteria, the third sentence "For each patient, this section may be photocopied, completed and submitted to the Statistical Center (see Section 14.4e) in lieu of comprehensive documentation of these items on the initial flow sheet" has been changed to "For each patient, this section *must* be photocopied, completed, and submitted to the Statistical Center (see Section 14.4e)." ("...*in lieu of comprehensive documentation of these items on the initial flow sheet*" has been removed).
9. (Page 20, Section 5.9, Eligibility Criteria): The following new eligibility criterion has been added: "Patients must agree to the serum sample submission schedule for HAMA testing as outlined in Section 15.5." Subsequent eligibility criteria have been renumbered.
10. (Page 20, Section 5.20, Eligibility Criteria): The second sentence of Section 5.20 has been revised. "If the patient's history is questionable, a MUGA scan must be obtained..." has been changed to "If the patient's *cardiac* history is questionable, a MUGA scan *or 2-d ECHO* must be obtained..." The result and date blanks in the Section 5.20 criterion have been updated for "*MUGA scan or 2-d ECHO*".
11. (Page 24, Sections 7.4, Treatment Plan): The second sentence of the "*" footnote, "The reason for omitting this drug must be noted on flow sheet", has been removed. *(Flow sheets are no longer used in this study.)*
12. (Page 25, Section 7.5, Treatment Plan): In the bolded paragraph, the second and third sentences, "The training session must occur prior to the dosimetric infusion of I-131-tositumomab, and the date of the training session must be noted on the Flow Sheet. For subsequent patients, the original training session date must still be noted on the Flow Sheets", have been replaced with the following updated information: "*The training session must occur prior to the dosimetric infusion of I-131 tositumomab, and the date of the training session must be noted on the **S0016** Tositumomab Treatment Form (Form #57993). For subsequent patients, the original training session date must still be noted on the **S0016** Tositumomab Treatment Form (Form #57993).*"
13. (Page 26, Section 7.5c, Treatment Plan): The second sentence of the "*" footnote,
"The reason for omitting this drug must be noted on flow sheet", has been removed. *(Flow sheets are no longer used in this study.)*
14. (Page 27, Section 7.5c.2iii, Treatment Plan): The bolded statement, "All concomitant medications must be recorded on the Flow Sheets" has been changed to "All concomitant medications *given in conjunction with the administration of tositumomab treatment must be recorded in the comments section of the **S0016** Tositumomab Treatment Form (Form #57993).*"

15. (Page 28, Section 7.5c.2iv, Treatment Plan): The third sentence, "These filters can be provided by GlaxoSmithKline upon request...", has been removed. *(GSK does not provide 0.22 micron in-line filters*).
16. (Page 29, Section 7.5c.3ii, Treatment Plan): The fifth sentence, "These filters can be provided by GlaxoSmithKline upon request...", has been removed. *(GSK does not provide 0.22 micron in-line filters*).
17. (Page 30, Section 7.10, Treatment Plan): "All reasons for discontinuation of treatment must be documented in the flow sheets" has been changed to "All reasons for discontinuation of treatment must be documented *on the Off Treatment Notice (Form #22204)*."
18. (Page 33, Section 8.4c, Toxicities to be Monitored and Dosage Modifications): The following changes have been made to this section:
• First paragraph: In the first sentence, the reference citation has been updated. Also, the first sentence, "Colony Stimulating Factors (CSF) should be administered..." has been changed to "Colony Stimulating Factors (CSF)*, such as pegfilgrastim (pegylated G-CSF), filgrastim (G-CSF) and sargramostim (GM-CSF)*, should be administered..."
• First and second paragraph: In the second sentence, "...but must be recorded on the flow sheets" has been changed to "...but must be recorded *in the comments section of the appropriate treatment form*."
19. (Page 35, Section 9.2, Study Calendar): The following changes have been made to this page:
• Laboratory: Added thyroid stimulating hormone testing (TSH) checkboxes to the following columns: prestudy, Day 29 (Cycle 2), Day 50 (Cycle 3), Day 71 (Cycle 4), Day 92 (Cycle 5), and Day 113 (Cycle 6). (*These columns were previously unchecked. TSH testing is recommended at prestudy for Good Medical Practice (See Section 7.1); TSH testing is required during Cycles 2-6 as hypothyroidism is monitored on the **S0016** CHOP/CHOP-R Treatment Form [Form #30359].)*
• Laboratory: "*Serum for HAMA testing*" has been added to the laboratory column. A "Δ" footnote has been added that indicates the following: "*See Section 15.5 for collection and submission instructions*." Timepoints for serum collection for HAMA testing have been checked for Days 133 (located under the Day 134 column), 200, 365, and 596 (located under the follow-up column).
• X-rays and scans: Under the x-rays and scans column, "MUGA" has been changed to "MUGA *or 2-d ECHO*".
Revised 12/8/06 20. (Page 36, Section 9.3, Study Calendar): The following changes have been made to this page:
• Laboratory: Added thyroid stimulating hormone testing (TSH) checkboxes to the following columns: Day 22 (Cycle 2), Day 43 (Cycle 3), Day 64 (Cycle 4), Day 85 (Cycle 5), and Day 106 (Cycle 6). (*These columns were previously unchecked. TSH testing is required during Cycles 2-6 as hypothyroidism is monitored on the **S0016** CHOP/CHOP-R Treatment Form [Form #30359].)*
• Laboratory: "*Serum for HAMA testing*" has been added to the laboratory column. A "Δ" footnote has been added that indicates the following: "*See Section 15.5 for collection and submission instructions*." Timepoints for serum collection for HAMA testing have been checked for Days 133, 200, 365, and 596 (located under the follow-up column).
• X-rays and scans: Under the x-rays and scans column, "MUGA" has been changed to "MUGA *or 2-d ECHO*".
21. (Page 39, Section 11.0, Statistical Considerations): The following changes have been made to this section:
• Section 11.2: Formatting changes have been made to this section, but the contents remain unchanged.
• A new Section 11.3 has been added outlining statistical considerations regarding HAMA testing. The subsequent section has been renumbered.
22. (Page 40, Section 12.2, Discipline Review): The fourth paragraph, "For any questions or problems regarding the Specimen Tracking System please send an email to technicalquestion@crab.org", has been replaced with the following paragraph:
"*To report technical problems with Specimen tracking, such as database errors or connectivity issues, please send an email to technicalquestion@crab.org. For procedural help with logging and shipping specimens, there is an introduction to the system on the Specimen Tracking main page http://dnet.crab.org/SpecTrack/Documents/SpecTPrimer-Insts.pdf); or contact the Data Operations Center at 206/652-2267 to be routed to the Data Coordinator for further assistance*."
23. (Page 40, Section 12.3, Discipline Review): The SWOG Lymphoma Repository address has been updated.
24. (Page 41, Section 13.3a, Registration Guidelines): In the first sentence of the paragraph that begins "For assistance with points 1 and 2..." the phone number for the Southwest Oncology Group Operations Office has been changed from 210/677-8808 to 210/*450*-8808.
25. (Page 42, Sections 14.2-14.3, Data Submission Schedule): The SWOG data submission procedures have been replaced with updated sections. Page 42a has been removed.

26. (Page 43, Section 14.5, Data Submission Schedule): "Completed Section 5.0 of the protocol" has been added as item "e". (*This item must be submitted within 14 days of registration.*)
27. (Page 43, Section 14.6, Data Submission Schedule): "Submit the **S0016** Tositumomab Treatment Form (Form #57993) and the **S0016** Adverse Event Form (Form #41405)" has been changed to "Submit the **S0016** Tositumomab Treatment Form (Form #57993)*, the **S0016** CHOP/CHOP-R Treatment Form (Form #30359),* and the **S0016** Adverse Event Form (Form #41405)."
28. (Page 43, Section 14.7, Data Submission Schedule): A new Section 14.7 has been created (subsequent sections have been renumbered): *"AT DAYS 133, 200, 365, AND 59: Submit serum specimens for HAMA testing as specified in Section 15.5a. For patients enrolled on this study before HAMA testing became mandatory, please see Section 15.5b for timing of serum collection."*
29. (Page 44, Section 15.2, Special Instructions): The paragraph that begins "Institutions are **required** to submit bone marrow specimens..." has been replaced with the following:
"*Institutions are **required** to submit the following specimens:*
a. Bone marrow (all patients): These specimens are being collected in order to compare the molecular remission rates by measuring clonal t(14:18)/bcl-2 rearrangements in the bone marrow at baseline and at one year posttreatment. Samples will be collected and stored. Testing will be done when funding is obtained.
b. Serum (only for patients on Arm 3): These specimens are being collected in order to determine the incidence and time to development of human antimouse antibody (HAMA) positivity."
30. (Page 45, Section 15.3c, Special Instructions): The third paragraph that begins "For any questions or problems regarding the Specimen Tracking program..." has been updated to provide additional contact information.
31. (Page 45a, Section 15.5, Special Instructions): A new Section 15.5 has been created outlining serum collection and submission instructions for HAMA testing. The subsequent section has been renumbered.
32. (Page 45b, Section 15.6, Special Instructions): "Instructions for submission of tissue..." has been changed to "Instructions for *snap frozen* tissue..."
33. (Pages 46a & 47a, Sections 16.1e & 16.1f, respectively; Adverse Event Reporting Requirements): In the last sentence, the phone number for the Southwest Oncology Group Operations Office has been changed from 210/677-8808 to 210/*450*-8808. Page 46a has been added to prevent repagination.
34. (Pages 49-50, Section 17.0, Bibliography): Bibliography listing #34 has been removed. *(This was listed in error.)* New bibliography items #24-26 have been added. Subsequent bibliography listings have been renumbered.
35. (Page 51, Section 18.2, Master Forms Set): The following changes have been made to this page:
• Section 18.2b: The **S0016** Non-Hodgkin's Lymphoma (Follicular) Prestudy Form has been updated.
• Section 18.2i: The Follow-Up Form has been updated.
• Final form numbers and version dates will be inserted into the protocol upon CTEP approval.
36. (Page 55, Model Consent Form, What is involved in this study): In the last paragraph, the following sentence was added as the second sentence: "*Your bone marrow will be looked at to find out if any lymphoma cells are present.*"
37. (Page 56, Model Consent Form, What is involved in this study): The first paragraph that begins "The bone marrow will be looked at..." has been updated to better describe bone marrow collection and submission. Page 56a was added to prevent extensive repagination.
38. (Page 81, Section 19.0, Appendix): A new Section 19.7 "HAMA assay – Methods" has been added. Corresponding section is included as page 104.
39. (Pages 94-95, Section 19.3a, Drug Ordering Instructions for Tositumomab and Iodine I-131 Tositumomab): The instructions for ordering tositumomab and iodine I-131 tositumomab have been replaced with updated guidelines.
40. (Page 96, Section 19.3b, Study Drug Order Form for S0016): The study drug order form for S0016 has been replaced with an updated document.
41. (Page 100, Section 19.6, CTSU participation procedures): Under "CTSU Procedures for Patient Enrollment", the CTSU patient registrar hours have been changed from "9:00 a.m. and 7:00 p.m." to "*8:00* a.m. and *8:00* p.m."
42. (Page 101, Section 19.6, CTSU participation procedures): Formatting changes have been made to this page, but the contents remain unchanged.
43. (Page 43, Section 19.6, CTSU participation procedures): Under "Specimen Submission", the following new paragraph related to serum submission for HAMA testing has been added: "*Serum for HAMA testing must be submitted to the SWOG Lymphoma Repository. Instructions for obtaining, handling, and shipping specimens are outlined in Section 15.5. Do not send specimens to the CTSU.*"

AMENDMENT #5
Distribution Date: April 15, 2007
CTEP Submission Date: April 2, 2007
The protocol has been amended as follows:
1. (Title page): The version date has been updated (version 03/28/07). The contact information for Drs. O. Press, D. Maloney, and R. Braziel have been updated.
2. (Pages 16-16f, Section 3.6b, Drug Information): The rituximab toxicology section has been replaced with an updated toxicology discussion including addition of the rituximab comprehensive adverse event and potential risk (CAEPR) list. (*Reason: Added to match the current rituximab adverse event list and post-marketing reports*.) Pages 16a-f have been added to prevent extensive repagination.
3. (Pages 32-33, Sections 8.3c-h, Toxicities to be Monitored and Dosage Modifications): Updated toxicity monitoring sections for hepatitis b reactivation with related fulminant hepatitis and other viral infections, severe mucocutaneous reactions, cardiovascular events, bowel obstruction and perforation, and renal complications have been added. (*Reason: Added per the current boxed warnings in the rituximab prescribing information*.) Page 33a has been added to prevent extensive repagination.
4. (Page 33a, Section 8.4c, Toxicities to be Monitored and Dosage Modifications): In the second paragraph, the misspelling of "transfusions" has been corrected.
5. (Page 42, Section 14.3d, Data Submission Schedule): The location for CTSU logistical information has been corrected from "Appendix 19.1" to "Appendix *19.6*". (*Reason: Change made to provide clarification.*)
6. (Page 45a, Section 15.5, Special Instructions): The following changes have been made to this section:
• Section 15.5a: "Institutions are required to submit serum for HAMA testing" has been changed to "Institutions are required to submit serum *for all patients* for HAMA testing".
• Section 15.5b.1: The timing of the serum collection for HAMA testing has been replaced with an updated section describing collection for both of the open arms of the trial [Arm 2 and Arm 3]). (*Reason: These changes were made to provide clarification that HAMA testing is required for* ***all*** *patients randomized to the open arms of the trial.*)
7. (Page 45b, Section 15.5d.1, Special Instructions): In the serum shipping instructions, the sentence "Please also enclose a copy of the eligibility checklist with each specimen (See Section 18.1)" has been removed. (*Reason: The eligibility checklist is not required for submission with each specimen per the SWOG Lymphoma Repository.*)
8. (Page 52, Model Informed Consent, Readability Statistics): The readability statistics have been updated.
9. (Pages 56-56a, Model Consent Form, What is Involved in this Study?): The second bullet relating to serum specimen submission for HAMA testing has been replaced with the following two bullet items:
• *Serum specimen submission (if you are assigned to Arm 2): You will have 10 mL (about 2-3 teaspoons) of serum collected on Day 133 (prior to receiving the 5th dose of rituximab), Day 200 (about 9 weeks after the final rituximab dose), Day 365 (about 8 months after the final rituximab dose) and Day 596 (about 15 months after the final rituximab dose). These serum specimens are being collected in order to find out if your body is producing antibodies to the rituximab treatment (called human anti-mouse antibodies or "HAMA".) If you registered to the study prior toHAMA testing becoming mandatory, your serum will be drawn at the next scheduled clinic visit (if applicable). If you are already beyond Day 596 in your protocol treatment, you will have a single serum specimen submitted at the time of your next scheduled doctor's visit.*
• *Serum specimen submission (if you are assigned to Arm 3): You will have 10 mL (about 2-3 teaspoons) of serum collected on Day 133 (prior to receiving the Iodine- 131 Anti-B1 dosimetric dose), Day 200 (about 9 weeks after the dosimetric dose), Day 365 (about 8 months after the dosimetric dose) and Day 596 (about 15 months after the dosimetric dose). These serum specimens are being collected in order to find out if your body is producing antibodies to the Iodine-131 anti-B1 treatment (called human anti-mouse antibodies or "HAMA".) If you registered to the study prior to HAMA testing becoming mandatory, your serum will be drawn at the next scheduled clinic visit (if applicable). If you are already beyond Day 596 in your protocol treatment, you will have a single serum specimen submitted at the time of your next scheduled doctor's visit.* (*Reason: These changes were made to provide clarification that HAMA testing is required for* ***all*** *patients randomized to the open arms of the trial.*)
10. (Pages 57-58a, Model Consent Form, What are the risks of the study?): The list of risks and side effects related to the rituximab regimen has been replaced with an updated section. (*Reason: This section was updated to match the current rituximab adverse event list and post-marketing reports*.) Page 58a has been added to prevent extensive repagination. Changes to adverse event frequency categories.

11. (Page 59a, Model Consent Form, What are the risks of the study?): The hepatitis B virus reaction paragraph (related to the rituximab regimen) has been moved to page 58a as all risk and side effects related to the rituximab regimen have been grouped together. *(Reason: This change was made for editorial purposes.)*
