# Supplementary material for: Treatment of Follicular Lymphoma With CHOP and Anti-CD20 Therapy: 15-Year Follow-Up of the SWOG S0016 Trial
Source: JAMA Oncol. 2026 Feb 26;12(4):394–401. doi: 10.1001/jamaoncol.2026.0042 (PMC12947078; doi:10.1001/jamaoncol.2026.0042)
Supplement: Supplement 2. — eFigure 1. CONSORT Diagram eAppendix. Sensitivity Analysis eTable 1. Sensitivity Analysis of Simulated Late Events and Estimated Cure Fraction eTable 2. Secondary Malignancies eTable 3. Cause of Death by Treatment Arm eTable 4. Cause of Death due to Secondary Malignancies eFigure 2. Cause of Death due to Secondary Malignancies eTable 5. Cause of Death due to AML/MDS [file jamaoncol-e260042-s002.pdf]

Supplemental Online Content

Shadman M, LeBlanc M, Rimsza L, et al. Treatment of follicular lymphoma with CHOP and anti-CD20 therapy: a secondary analysis of the S0016 trial. *JAMA Oncol*. Published online February 26, 2026. doi:10.1001/jamaoncol.2026.0042

eFigure 1. CONSORT Diagram

eAppendix. Sensitivity Analysis

eTable 1. Sensitivity Analysis of Simulated Late Events and Estimated Cure Fraction

eTable 2. Secondary Malignancies

eTable 3. Cause of Death by Treatment Arm

eTable 4. Cause of Death due to Secondary Malignancies

eFigure 2. Cause of Death due to Secondary Malignancies

eTable 5. Cause of Death due to AML/MDS

eFigure 3. Cause of Death due to AML/MDS

This supplemental material has been provided by the authors to give readers additional information about their work.

© 2026 Shadman M et al. *JAMA Oncology*.

eFigure 1 – CONSORT Diagram

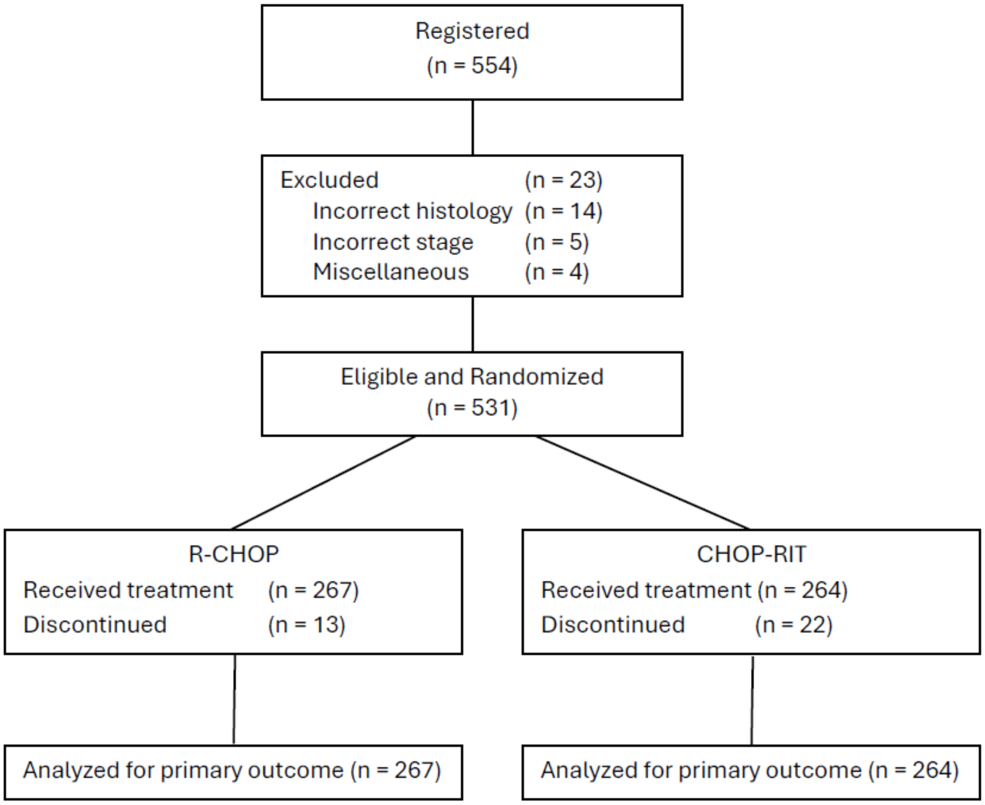

© 2026 Shadman M et al. *JAMA Oncology*.

eAppendix – Sensitivity Analysis for Long-Term Event Ascertainment

To assess the potential impact of late under-ascertainment of disease-specific progression events on the estimated cure fraction, a sensitivity analysis was conducted. The analysis focused on the period beginning 10 years after registration, when surveillance becomes less frequent and some events may be missed.

Observed cause-specific event rates during the 10–15 year and 15–20 year intervals were used as the baseline for generating additional pseudo-events. Within each interval, additional pseudo-events were simulated to represent potentially missed lymphoma-related progressions. These pseudo-events were then added to the observed data.

The models allowed for differential rates of under-ascertainment across time intervals, with the assumption that the rate of missed events may increase in the later (15–20 year) period. The table below provides the percentage of additional cause-specific events assumed for each interval. For example, the first row corresponds to a scenario in which the number of disease-specific events is inflated by 25% in the 10–15 year window and by 50% in the 15–20 year window. The cure fraction estimated from the observed data without added events was 42%.

© 2026 Shadman M et al. *JAMA Oncology*.

eTable 1 – Sensitivity Analysis of Simulated Late Events and Estimated Cure Fraction

Values in the first two columns represent the proportional increase in lymphoma-specific events assumed to have been missed.

Additional events (10–15 y)	Additional events (15–20 y)	Estimated cure (%)
25%	50%	38.0
50%	50%	33.8
50%	75%	32.8
50%	100%	31.5
75%	100%	27.2
100%	100%	22.9

© 2026 Shadman M et al. *JAMA Oncology*.

eTable 2 – Secondary Malignancies

The incidence of second malignancies was not different between the two arms. Fifty-nine patients (22.1%) on the R-CHOP arm and 52 patients (19.7%) on CHOP-RIT arm developed secondary malignancies (P=0.52). Six patients (2.2%) who were treated on the R-CHOP arm and 14 patients (5.3%) who received CHOP-RIT developed myeloid malignancies (AML, acute myeloid leukemia, or MDS, myelodysplastic syndrome) (P=0.08).

Table S2a: Secondary Malignancies (per patient *)

	R-CHOP (N=267)		CHOP-RIT (N=264)		Two-sided P-value*
All Second Malignancies	59	22.1%	52	19.7%	0.52
AML/MDS	6	2.2%	14	5.3%	0.08

*two-sided Fisher's exact test

© 2026 Shadman M et al. *JAMA Oncology*.

eTable S2b: Secondary Malignancies (per event *)

	R-CHOP (N=267)	CHOP-RIT (N=264)
AML/MDS	6	16
Hematologic (other than AML/MDS)	4	3
Gastrointestinal	12	2
Genitourinary	10	8
Gynecologic	1	2
Lung	5	3
Breast	5	0
Head and Neck	0	3
Thyroid	2	0
Melanoma	4	5
Skin (other than melanoma)	12	14
Unknown primary	1	0

*7 patients on R-CHOP and 5 patients on CHOP-RIT arm developed more than one secondary malignancy.

© 2026 Shadman M et al. *JAMA Oncology*.

eTable 3 – Cause of Death by Treatment Arm

There were 171 deaths (84 in R-CHOP and 87 in CHOP-RIT arms) during follow-up. There was no overall difference in causes of death between the two arms (2-sided fisher's exact P=0.27).

Cause of Death	R-CHOP (N=267)		CHOP-RIT (N=264)	
Medical issues other than cancer	23	8.6%	17	6.4%
Transplant complications	3	1.1%	0	0
Lymphoma	31	11.6%	40	15.2%
Second malignancies	15	5.6%	19	7.2%
Unknown	12	4.5%	10	3.8%
**Total**	84	31.5%	87	32.9%

© 2026 Shadman M et al. *JAMA Oncology*.

eTable 4 – Cause of Death due to Secondary Malignancies

Fifteen patients (5.6%) in the R-CHOP arm and 19 patients (7.2%) in the CHOP-RIT died from secondary malignancies during follow-up. This translated to an estimated 15-year cumulative incidence (Aalen Johansen estimator) of death of 5.4% (95% CI, 3.3%-8.9%) in the R-CHOP arm and 7.0% (95% CI, 4.6%-10.8%) in the CHOP-RIT arm.

	Total	Event	Competing Event	Censored	P-value	Estimate of 15-year Cumulative Incidence of Deaths due to Secondary Malignancy
Treatment Arm	531	34	137	360	0.45	sHR=1.3 (95% CI, 0.66–2.6)
R-CHOP	267	15	69	183		5.4% (95% CI, 3.3%-8.9%)
CHOP-RIT	264	19	68	177		7.0% (95% CI, 4.6%-10.8%)

© 2026 Shadman M et al. *JAMA Oncology*.

eFigure 2 – Cause of Death due to Secondary Malignancies

Gray’s test was used to test equality of cumulative incidence function (CIF) across treatment groups. Cumulative incidences for R-CHOP and CHOP-RIT are not statistically different for death due to secondary malignancy (P=0.45). The estimated 15-year CIF for death due to secondary malignancy is 5.4% for the R-CHOP arm and 7.0% for CHOP-RIT arm.

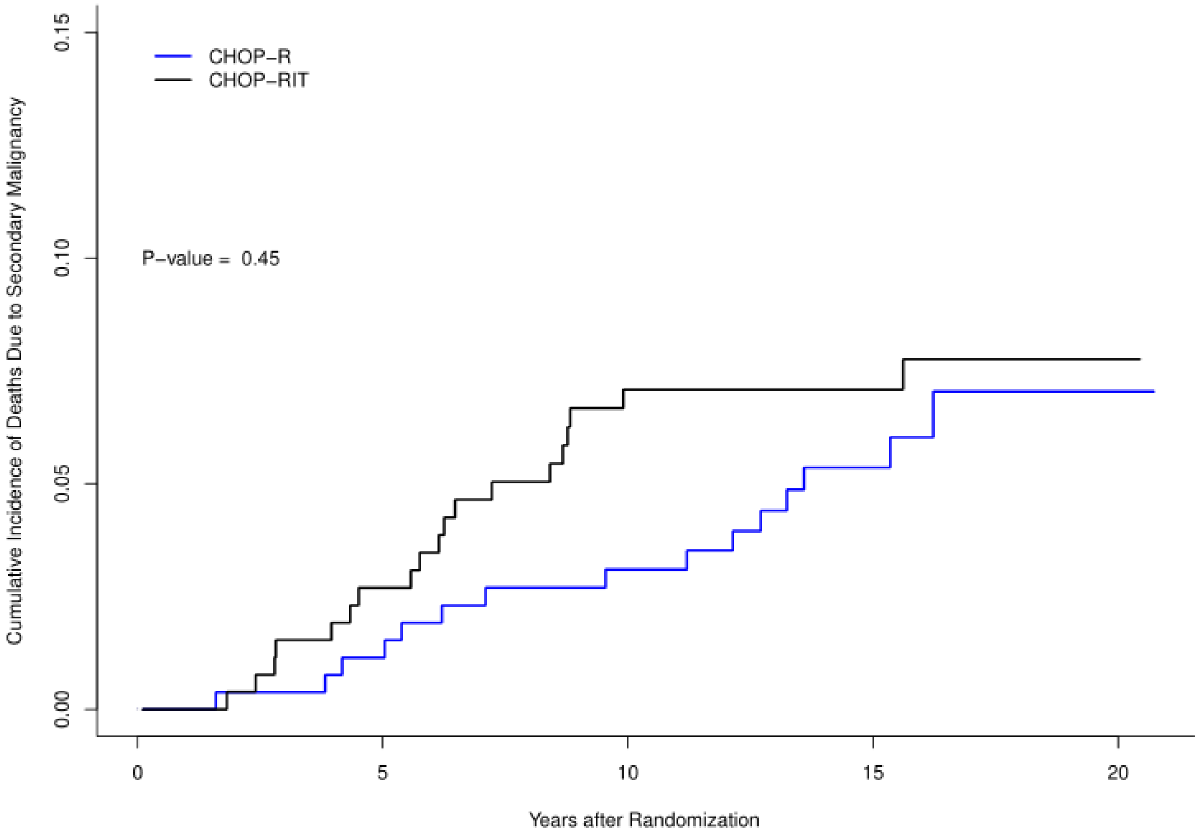

© 2026 Shadman M et al. *JAMA Oncology*.

eTable 5 – Cause of Death due to AML/MDS

AML/MDS caused death in 3 (1.1%) and 11 (4.2%) patients in the R-CHOP and CHOP-RIT arms, respectively. Estimated 15-year cumulative incidences of death were 1.2% (95% CI 0.4%-3.7%) in R-CHOP arm and 4.4% (95% CI, 2.4%-8.0%) in the CHOP-RIT arm (P=0.03).

	Total	Event	Competing Event	Censored	P-value	Estimate of 15-year Cumulative Incidence of Deaths due to AML/MDS
Treatment Arm	531	14	157	360	0.03	sHR=3.8 (95% CI, 1.1–13.5
R-CHOP	267	3	81	183		1.2% (95% CI, 0.4%-3.7%)
CHOP-RIT	264	11	76	177		4.4% (95% CI, 2.4%-8.0%)

© 2026 Shadman M et al. *JAMA Oncology*.

eFigure 3 – Cause of Death due to AML/MDS

Cumulative incidences of death resulting from acute myeloid leukemia (AML) or myelodysplastic syndrome (MDS). Gray's test was used to test equality of cumulative incidence function (CIF) across treatment groups.

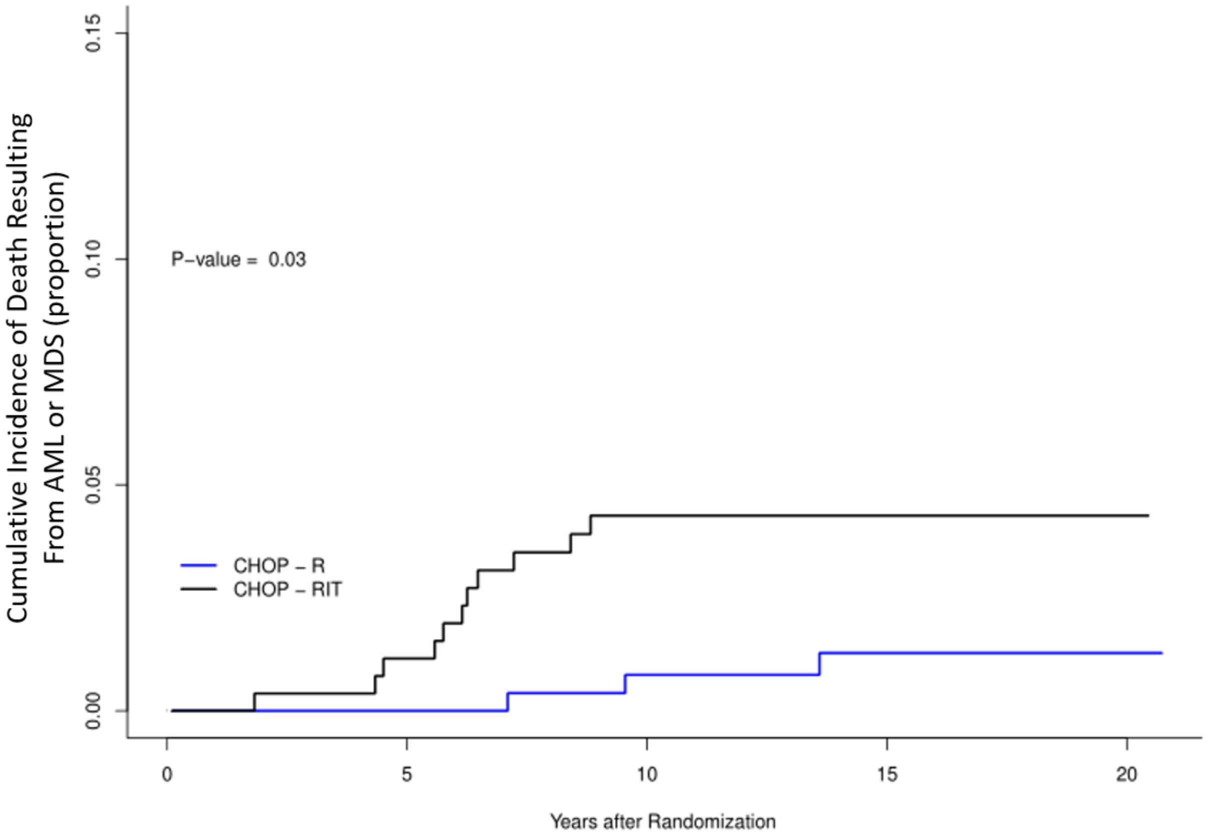

© 2026 Shadman M et al. *JAMA Oncology*.
