# Supplementary material for: Treatment of Follicular Lymphoma With CHOP and Anti-CD20 Therapy: 15-Year Follow-Up of the SWOG S0016 Trial
Source: JAMA Oncol. 2026 Feb 26;12(4):394–401. doi: 10.1001/jamaoncol.2026.0042 (PMC12947078; doi:10.1001/jamaoncol.2026.0042)
Supplement: Supplement 3. — Data sharing statement [file jamaoncol-e260042-s003.pdf]

Data Sharing Statement

Shadman. Treatment of Follicular Lymphoma With CHOP and Anti-CD20 Therapy. *JAMA Oncol*. Published February 26, 2026. doi:10.1001/jamaoncol.2026.0042

Data
Additional Information: ClinicalTrials.gov; NCT00006721; https://clinicaltrials.gov/study/NCT00006721
Data available: Yes
Data types: Deidentified participant data, Participant data with identifiers
How to access data: Requests for SWOG data to be released and analyzed by an outside investigator are governed by the SWOG Data Sharing Policy/Agreement. Only data from studies that have been previously published in a peer-reviewed publication may be released. Furthermore, single institution-data use and publication should occur only after a formal data request has been submitted to SWOG and, if approved, a DUA executed. SWOG recognition in the acknowledgements, including appropriate grant acknowledgement, is required. Prior to journal submission, manuscript draft must be sent to SWOG publications office (pubs@swog.org) to insure compliance with Group policy.
When available: With publication

Supporting Documents
Document types: None

Additional Information
Who can access the data: The data may be shared with outside investigators after formal request and if in alignment with the SWOG Data Sharing Policy.
Types of analyses: The data may be shared with outside investigators after formal request, the purpose of the research should be discussed with SWOG at the time of request.
Mechanisms of data availability: The data may be shared with outside investigators after formal data request has been submitted to SWOG and, if approved, a DUA executed.
